# A robust host-response-based signature distinguishes bacterial and viral infections across diverse global populations

**DOI:** 10.1016/j.xcrm.2022.100842

**Published:** 2022-12-20

**Authors:** Aditya M. Rao, Stephen J. Popper, Sanjana Gupta, Viengmon Davong, Krista Vaidya, Anisone Chanthongthip, Sabine Dittrich, Matthew T. Robinson, Manivanh Vongsouvath, Mayfong Mayxay, Pruksa Nawtaisong, Biraj Karmacharya, Simone A. Thair, Isaac Bogoch, Timothy E. Sweeney, Paul N. Newton, Jason R. Andrews, David A. Relman, Purvesh Khatri

**Affiliations:** 1 Institute for Immunity, Transplantation, and Infection, Stanford University School of Medicine, 240 Pasteur Dr., Biomedical Innovation Building, Room 1553, Stanford, CA, USA; 2 Immunology Graduate Program, Department of Medicine, Stanford University, Stanford, CA, USA; 3 Division of Infectious Diseases and Geographic Medicine, Department of Medicine, Stanford University, Stanford, CA, USA; 4 Center for Biomedical Informatics Research, Department of Medicine, Stanford University, Stanford, CA, USA; 5 Lao-Oxford-Mahosot Hospital-Wellcome Trust Research Unit, Microbiology Laboratory, Mahosot Hospital, Vientiane, Lao PDR; 6 Dhulikhel Hospital, Kathmandu University Hospital, Kavrepalanchok, Nepal; 7 Centre for Tropical Medicine and Global Health, Nuffield Department of Medicine, University of Oxford, Oxford, UK; 8 Institute of Research and Education Development (IRED), University of Health Sciences, Ministry of Health, Vientiane, Lao PDR; 9 Inflammatix Inc., Sunnyvale, CA, USA; 10 Department of Medicine, University of Toronto, Toronto, ON, Canada; 11 Department of Microbiology and Immunology, Stanford University, Stanford, CA, USA; 12 Infectious Diseases Section, Veterans Affairs Palo Alto Health Care System, Palo Alto, CA, USA

**Keywords:** host response, antimicrobial resistance, global health, public health, gene expression, point-of-care diagnostics, infectious disease diagnosis, bacterial vs. viral diagnosis

## Abstract

Limited sensitivity and specificity of current diagnostics lead to the erroneous prescription of antibiotics. Host-response-based diagnostics could address these challenges. However, using 4,200 samples across 69 blood transcriptome datasets from 20 countries from patients with bacterial or viral infections representing a broad spectrum of biological, clinical, and technical heterogeneity, we show current host-response-based gene signatures have lower accuracy to distinguish intracellular bacterial infections from viral infections than extracellular bacterial infections. Using these 69 datasets, we identify an 8-gene signature to distinguish intracellular or extracellular bacterial infections from viral infections with an area under the receiver operating characteristic curve (AUROC) > 0.91 (85.9% specificity and 90.2% sensitivity). In prospective cohorts from Nepal and Laos, the 8-gene classifier distinguished bacterial infections from viral infections with an AUROC of 0.94 (87.9% specificity and 91% sensitivity). The 8-gene signature meets the target product profile proposed by the World Health Organization and others for distinguishing bacterial and viral infections.

## Highlights

• Intracellular and extracellular bacterial infection prevalence differ across countries
• Host-response-based signatures have low accuracy for intracellular bacterial infections
• 8-gene signature distinguishes bacterial versus viral infection despite broad heterogeneity
• 8 genes diagnose intra- and extracellular bacterial infection with similar accuracy

## Graphical abstract

**Figure:**
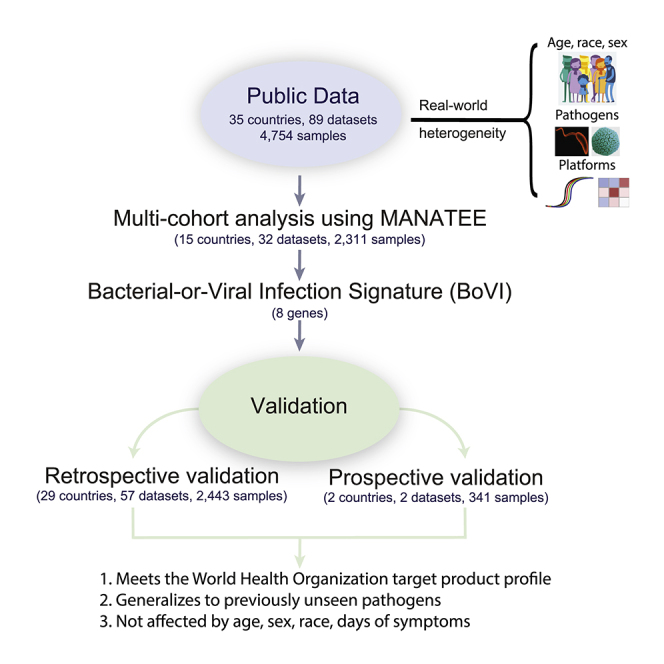

## Teaser

Current host-response-based diagnostics have lower accuracy for distinguishing intracellular bacterial infections from viral infections. Rao et al. identified an 8-gene signature to address this challenge. Using multiple validation cohorts, they show that the 8-gene signature generalizes to global patient population with similar accuracy for extra- and intracellular bacterial infections.

## Introduction

Early and accurate diagnosis of acute infection has important consequences for clinical outcome, antibiotic stewardship, and allocation of health care resources. However, the majority of patients with acute infection never receive a positive microbiological diagnosis.^1^^,^^2^ Acute systemic infections typically present with non-specific signs and symptoms, and yet decisions about clinical management and treatment are often made on the basis of clinical presentation, local epidemiology, and local clinical standards of care, leading to the erroneous prescription of empiric antibiotics in 30%–75% of patients with viral infections in the US, Canada, or the UK.^3^^,^^4^ This number is even higher in low- and middle-income countries (LMICs), with up to 95% of patients with non-bacterial infections inappropriately prescribed antibiotics.^5^^,^^6^ With resistance to antimicrobial treatment rising, the health and economic burden of these misdiagnoses is, and will continue to be, very large.^7^^,^^8^ There is a critical unmet need to develop diagnostics that can safely rule out bacterial infection.

To address this challenge the World Health Organization (WHO) and the Foundation for Innovative New Diagnostics (FIND), together with several others, have proposed diagnostic performance requirements of >90% sensitivity and >80% specificity for distinguishing bacterial from non-bacterial infections.^9^ None of the current pathogen-detecting diagnostic solutions meet this target product profile (TPP). Despite the recent popularity of metagenomic sequencing, the sensitivity and interpretability of this approach remain in question.^10^^,^^11^ In fact, there have been calls for establishing next-generation sequencing (NGS) stewardship boards to restrict its use in intensive care units (ICUs) to only patients who truly need it.^12^ In contrast, a host-response-based diagnostic test for infectious diseases can be sensitive, more easily interpreted, and cheaper with faster turnaround time (TAT). However, host-response-based single-biomarker tests such as C-reactive protein (CRP), pro-calcitonin (PCT), and lactate are often non-specific for the type of infection and can be elevated in the inflammatory response to non-infection settings such as trauma.^9^^,^^10^ The information gained from these tests has limited clinical utility, as negative results have poor predictive value and often are not sufficient to rule out infection.^13^

Recently, several studies have demonstrated the ability of host-response-based gene signatures to diagnose the presence and type of infection even when the pathogen itself is not detected.^14^^,^^15^^,^^16^ However, these signatures were identified and validated exclusively in populations from Western Europe and North America, whereas the greatest burden of systemic infection exists elsewhere.^17^^,^^18^ In fact, a recent study suggested that the vast majority of sepsis cases occur in countries with low socio-demographic indices.^19^ Further, the epidemiology of febrile illness in these countries includes a distinct group of bacterial and viral pathogens. For instance, important infectious causes of fever in LMICs, particularly in Asia, are either not endemic or more rarely acquired in Europe or North America, including typhoid, dengue, and scrub typhus (*Orientia tsutsugamushi*).^20^^,^^21^^,^^22^ In addition, bacterial pathogens that are common in LMICs are often obligate or facultative intracellular pathogens that, similar to viruses, generate an interferon-driven host response that leads to different modulations of host cell metabolism and gene expression than what is observed in response to extracellular bacterial infections.^23^^,^^24^ Note that this dichotomous classification of bacterial pathogens as intracellular or extracellular pathogens is simplistic because certain pathogens (e.g., *Staphylococcus aureus*) can occupy both niches. The qualifier terms, intracellular and extracellular, are sometimes used to indicate bacterial infections that are differentially enriched in studies from LMIC and non-LIMC countries, respectively.

These differences in both demographics and causative infectious agents have underscored the importance of accounting for real-world biological, clinical, and technical heterogeneity in developing diagnostics generalizable to global populations. For example, a host-response-based signature for tuberculosis (TB) identified using samples from one country failed to replicate in samples from other countries on the same continent.^25^ On the other hand, a host-response-based diagnostic signature for TB that was discovered using multiple independent cohorts across several countries has been independently validated in several follow-up prospective studies and translated into a point-of-care test,^26^^,^^27^ and has also been validated in additional prospective cohorts.^28^ Similar results have also been observed in host-response-based diagnostic signatures for sepsis where an 11-gene signature derived using multiple independent cohorts has outperformed those derived using a single cohort.^29^^,^^30^

Here, we hypothesized that recently described host-response-based mRNA signatures for distinguishing bacterial and viral infections do not account for biological heterogeneity in the immune response to intracellular bacterial infections, and therefore will not distinguish intracellular infections from viral infections with clinically useful accuracy. Our comparison of 4 host-response-based gene signatures, ranging from 2 to 120 genes, demonstrated, as hypothesized, their lower accuracy in distinguishing intracellular bacterial infections from viral infections. Next, we developed a multi-cohort analysis framework to enable integration of a large number of independent heterogeneous datasets with bacterial infections (extracellular or intracellular) and viral infections, and used it to derive an 8-gene host-response-based signature that is able to distinguish extracellular and intracellular bacterial infections from viral infections with comparable accuracy. Finally, we validated the 8-gene signature and observed increased accuracy in multiple independent retrospective datasets and two prospective cohorts from Nepal and the Lao People’s Democratic Republic (Laos).

## Results

### Current host-response-based gene signatures have lower accuracy for distinguishing intracellular bacterial infections from viral infections

We hypothesized that a host-response-based gene signature identified using only blood samples from patients with viral or extracellular bacterial infections would have lower accuracy in distinguishing intracellular bacterial infections from viral infections. To test this hypothesis, we performed a search of public data repositories to identify blood transcriptome profiles from febrile patients with extracellular bacterial, intracellular bacterial, or viral infections.^31^^,^^32^ We identified 64 independent datasets including 55 from whole-blood (WB) samples and nine from peripheral blood mononuclear cell (PBMC) samples that met our inclusion and exclusion criteria (STAR Methods; Tables S1 and S2).^14^^,^^15^^,^^16^^,^^23^^,^^24^^,^^30^^,^^33^^,^^34^^,^^35^^,^^36^^,^^37^^,^^38^^,^^39^^,^^40^^,^^41^^,^^42^^,^^43^^,^^44^^,^^45^^,^^46^^,^^47^^,^^48^^,^^49^^,^^50^^,^^51^^,^^52^^,^^53^^,^^54^^,^^55^^,^^56^^,^^57^^,^^58^^,^^59^^,^^60^^,^^61^^,^^62^^,^^63^^,^^64^^,^^65^^,^^66^^,^^67^^,^^68^^,^^69^^,^^70^^,^^71^^,^^72^^,^^73^ These 64 datasets included transcriptome profiles of 3,708 blood samples from 1,186 healthy controls and 2,522 patients with a microbiologically confirmed infection (728 extracellular bacterial infections, 301 intracellular bacterial infections, 191 infections with an unspecified bacterial pathogen, 1,302 viral infections) collected from 20 countries across six continents, with similar representation of both sexes and a wide range of ages (Table S3). These samples also include a wide range of extracellular bacteria (e.g., *S. aureus*, *Escherichia coli*, *Streptococcus pneumoniae*, *Leptospira* spp., *Streptococcus pyogenes*, *Klebsiella pneumoniae*), intracellular bacteria (e.g., *Burkholderia pseudomallei*, *Salmonella enterica* Typhi, *Rickettsia typhi*, *Orientia tsutsugamushi*, *Brucella* spp.), and viruses (e.g., influenza, dengue, cytomegalovirus [CMV], parainfluenza, adenovirus, human rhinovirus, respiratory syncytial virus) (Table S4). Finally, the patients were enrolled in diverse clinical settings, including outpatient clinics, emergency departments, general wards, and intensive care units (Tables S1 and S2).

After co-normalizing the 64 datasets using Combat Co-normalization Using Controls (COCONUT) (STAR Methods), we compared four previously described host-response-based gene signatures for distinguishing bacterial and viral infections.^74^ The number of genes in these signatures ranged from two to 120, and they were each derived using between 240 and 599 samples. The Sampson4^75^ and Sweeney7^74^ signatures were derived using multiple independent cohorts, whereas the Herberg2^14^ and Tsalik120^76^ signatures were each derived from a single cohort. Importantly, none of these signatures were derived using samples from patients with intracellular bacterial infections.

**Figure 1:**
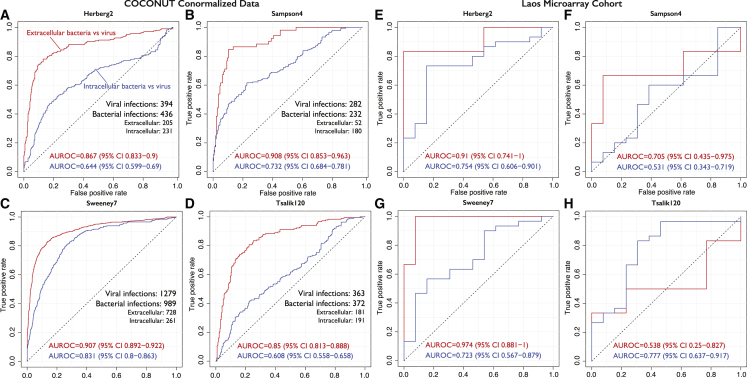
Existing signatures derived by comparing only extracellular bacterial infections and viral infections have lower accuracy in distinguishing intracellular bacterial infections from viral infections ROC curves comparing the performance of 4 published signatures at separating extracellular bacterial and viral infections versus separating intracellular bacterial and viral infections. (A–D) Receiver operating characteristic (ROC) curves from testing the signatures in up to 64 COCONUT co-normalized datasets. (E–H) ROC curves from testing the signatures in the Laos Microarray cohort. See also Figures S1 and S2, and Tables S1–S5 and S7.

Each of the four signatures had high accuracy in distinguishing extracellular bacterial infections from viral infections in the 64 co-normalized datasets, with the AUROC ranging from 0.85 for Tsalik120 to 0.91 for Sampson4 and Sweeney7 (Figures 1A–1D). However, each signature had significantly lower accuracy in distinguishing intracellular bacterial from viral infections, with AUROCs ranging from 0.61 for Tsalik120 to 0.83 for Sweeney7 (Figures 1A–1D). The difference in AUROCs for distinguishing extracellular or intracellular bacterial infections from viral infections ranged from 7.6% (Sweeney7) to 24.2% (Tsalik120) (Figures 1A–1D). To ensure that the higher accuracy for extracellular bacterial infection was not an artifact of COCONUT co-normalization, we also profiled each of the four signatures in an independent cohort of 61 WB samples from Laos, including healthy controls (n = 12) and patients with extracellular bacterial (n = 6), intracellular bacterial (n = 30), and viral infections (n = 13) using a custom cDNA array (Tables S1 and S5). In the Laos Microarray cohort, three of the four signatures had higher accuracy for extracellular bacterial infections than intracellular bacterial infections (difference in AUROC >15%), and no signature demonstrated clinically useful accuracy in distinguishing intracellular bacterial from viral infections, with AUROCs ranging from 0.531 to 0.777 (Figures 1E–1H).

Arguably, these estimates may be confounded as we could not use all datasets to evaluate every signature, since not all genes for each of the four signatures were measured across every dataset. Therefore, we performed an extensive random sampling of our datasets and analyzed each signature’s performance across 1,396,677 unique combinations (STAR Methods). Irrespective of the number of samples and datasets used, each signature had consistently higher accuracy for extracellular bacterial infections. The difference between the AUROC for distinguishing extracellular bacterial infections and that for distinguishing intracellular bacterial infections ranged from approximately 10% for Sweeney7 to just under 20% for Herberg2 (Figure S1). Interestingly, the difference in AUROCs had higher variability for the Sampson4 and Tsalik120 signatures than the Herberg2 and Sweeney7 signatures. One explanation for this is that some datasets did not measure all of the genes in Sampson4 and Tsalik120. However, we found that, even when fewer genes were missing, the Tsalik120 signature had higher AUROC for distinguishing extracellular bacterial infections from viral infections than for distinguishing intracellular bacterial infections from viral infections (Figure S2).

Collectively, these results strongly supported our hypothesis that a host-response-based gene signature derived using only samples from patients with extracellular bacterial or viral infections will have lower discriminatory power for distinguishing intracellular bacterial infections from viral infections, resulting in severely restricted generalizability in the global fight against antimicrobial resistance. They were also in concordance with previous observations that signatures developed by leveraging heterogeneity across multiple datasets are more robust than those developed using single cohorts.^25^^,^^29^^,^^77^^,^^78^ Importantly, these results demonstrated that virtually none of the host-response-based diagnostic signatures described to date are generalizable to LMICs where the incidence of intracellular bacterial infections is as high or higher than extracellular bacterial infections.

### Deriving an 8-gene signature for distinguishing bacterial and viral infections with MANATEE

**Figure 2:**
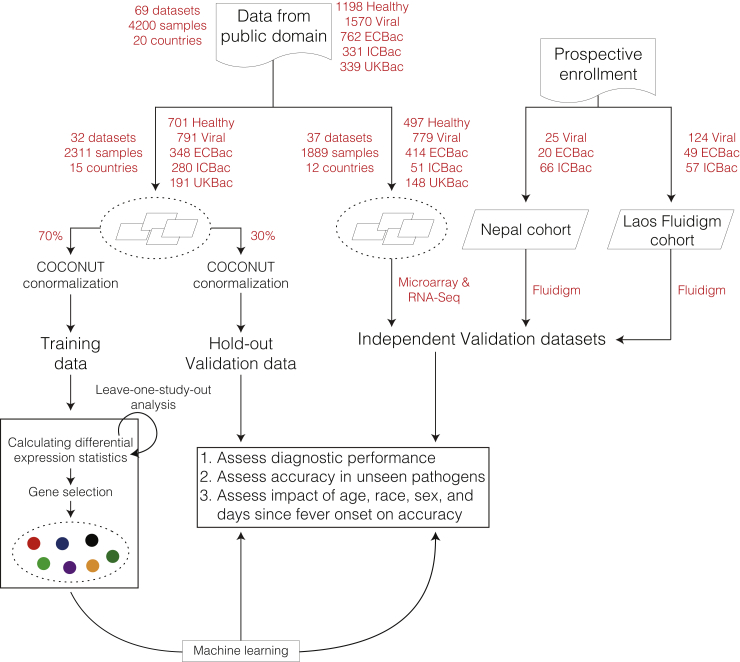
MANATEE framework diagram Schematic of the multi-cohort analysis work flow that was used for training and validation of the BoVI signature. The Laos Microarray cohort was combined with 31 datasets from the public domain, for a total of 32 datasets used in signature discovery. ECBac, extracellular bacterial infection; ICBac, intracellular bacterial infection; UKB, unknown or unspecified bacterial infection. See also Figure S3 and Tables S3 and S4.

We set out to identify a globally generalizable host-response-based gene signature for distinguishing viral infections from either extracellular or intracellular bacterial infections with comparable accuracy. However, despite the large amount of blood transcriptome data available from patients with either extracellular bacterial, intracellular bacterial, or viral infections, only 3 datasets included transcriptome profiles from all 3 classes of infections. Therefore, we devised a machine learning framework, referred to as Multi-cohort Analysis of Aggregated Gene Expression (MANATEE), to aggregate and analyze data from a large number of independent studies while reducing the risk of overfitting (Figure 2 and STAR Methods).

Prior to applying MANATEE, we aggregated the 64 datasets with the Laos Microarray dataset and 5 additional publicly available datasets (3 WB and 2 PBMC) composed of 431 samples.^14^^,^^73^^,^^76^ These five datasets did not include healthy subjects and thus could not be co-normalized using COCONUT, but, because each dataset included both patients with bacterial and viral infections, they could still be used to validate any signatures we discovered. Overall, we identified 69 datasets composed of 4,200 samples (Figure 2). We divided these 69 datasets into 3 groups: training, hold-out validation, and independent validation (Tables S1–S3 and S6). In order to ensure that no samples from the datasets used in independent validation were included in either training or hold-out validation, we chose 32 WB datasets (2,311 samples) and *a priori* randomly divided them into the training set (70%) and the hold-out validation set (30%). We used the remaining 37 WB or PBMC datasets (1,889 samples) as an independent validation set to ensure the generalizability of a signature derived using the training and hold-out validation sets.

We selected three sets of genes using stringent selection criteria in the training set: (1) 417 genes with FDR ≤ 1% and absolute effect size (|ES|) ≥ 0.6, (2) 76 genes with FDR ≤ 1% and |ES| ≥ 0.8, and (3) 100 genes with the highest scores from a significance analysis of microarrays (SAM) analysis (Table S7). We further applied different strategies to identify a parsimonious set of genes used for distinguishing extracellular and intracellular bacterial infections from viral infections (STAR Methods). There were two signatures with fewer than 10 genes, making them suitable for clinical translation. Of these, an 8-gene signature had consistently higher AUROCs for distinguishing viral infection from bacterial infection in both training and hold-out validation sets (Table S7). Therefore, we chose the 8-gene signature for further investigation.

**Figure 3:**
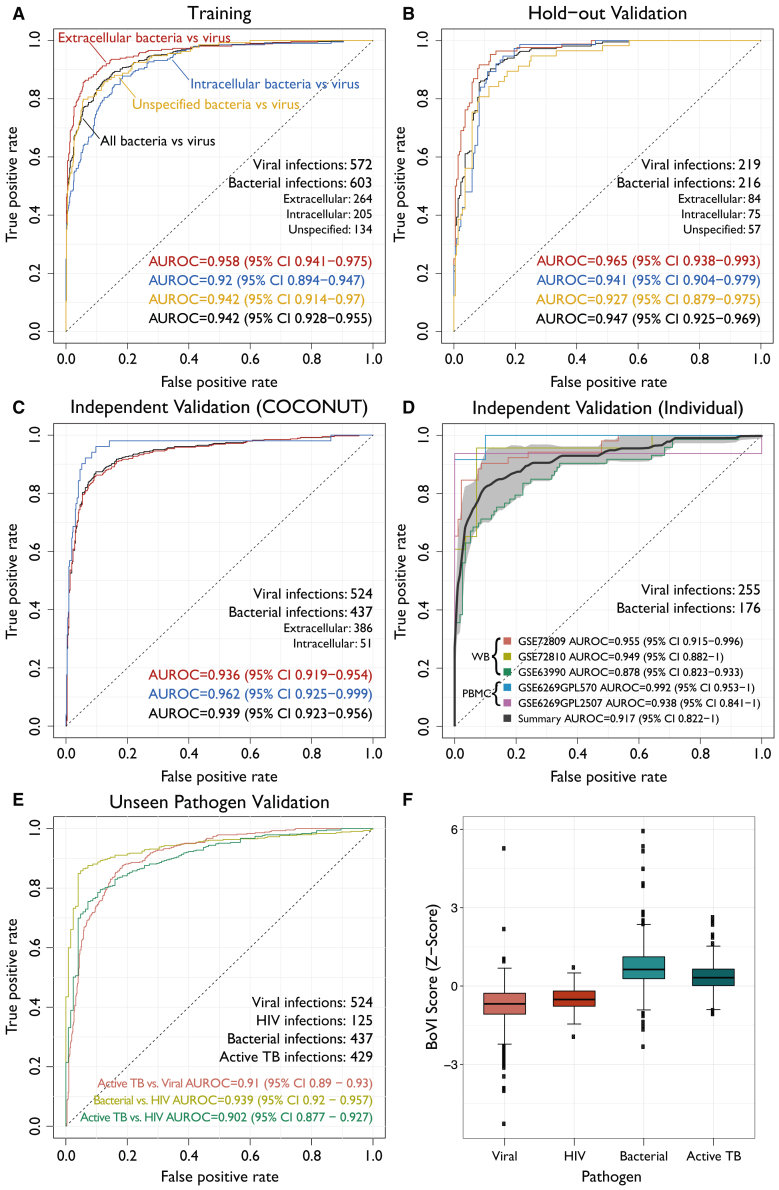
Performance of the BoVI signature in training, hold-out validation, and independent validation data ROC curves were generated for training, hold-out validation, and both independent validation sets. The number of samples in each infection category is shown for each stage. (A–C) Across the co-normalized data, performance was assessed for four comparisons: extracellular bacterial infections versus viral infections (red), intracellular bacterial infections versus viral infections (blue), bacterial infections with an unknown or unspecified pathogen versus viral infections (yellow), and all bacterial infections versus viral infections (black). (D) For individual datasets, performance was assessed by pooling the bacterial versus viral AUROCs for each dataset in order to generate a summary ROC curve (black) (STAR Methods). The weighted SD for the summary ROC curve is shown in gray. Each dataset was designated as containing whole blood (WB) or peripheral blood mononuclear cell (PBMC) samples. GSE6269 was measured on two non-overlapping platforms (GPL570 and GPL2507), so each platform was assessed separately. (E) Performance was assessed for HIV and active TB infections. (F) Boxplots of the BoVI score across unseen pathogens. The BoVI score was centered and scaled. See also Figures S4–S6, and Tables S6 and S8.

The 8-gene signature included three genes over-expressed in bacterial infections (*SMARCD3*, *ICAM1*, *EBI3*; “bacterial genes”) and five over-expressed in viral infections (*JUP*, *SUCLG2*, *IFI27*, *FCER1A*, *HESX1*; “viral genes”) (Figures S3A and S3B). We defined a bacterial or viral infection (BoVI) score for each sample as the difference between the geometric mean of the expression of the three bacterial genes and the geometric mean of the five viral genes (STAR Methods). The BoVI score distinguished viral infections from extracellular and intracellular bacterial infections with high accuracy in the training (AUROC = 0.942; 95% confidence interval [CI], 0.928–0.955) and hold-out validation (AUROC = 0.947; 95% CI, 0.925–0.969) sets (Figures 3A and 3B). Importantly, the high accuracy of the BoVI score in training and hold-out validation was not dependent on any single pathogen (Figures S4A and S4B). At >90% sensitivity, the BoVI score had specificities of 84.4% and 84% in the training and the hold-out validation sets (Table 1), respectively, which met the diagnostic TPP for distinguishing bacterial infections from viral infections.^9^ These high accuracies translated to positive predictive values (PPVs) >85%, negative predictive values (NPVs) ≥89%, and diagnostic odds ratios (DORs) >49 at 51.3% prevalence in the training set and 49.7% prevalence in the hold-out validation set, respectively (Table 1).

**Table 1:** Diagnostic statistics for the BoVI signature across all analysis stages

Dataset group	Dataset	Prevalence of bacterial infection (%)	AUROC	Sensitivity (%)	Specificity (%)	PPV (%)	NPV (%)	LR+	LR−	DOR
Discovery datasets	training	51.3	0.94 (0.93–0.96)	90.0	84.4	85.9	89.0	5.79	0.118	49.1
	hold-out validation	49.7	0.95 (0.93–0.97)	91.7	84.0	85.0	91.1	5.74	0.099	58
Retrospective validation datasets	independent validation (COCONUT)	45.5	0.94 (0.92–0.96)	90.2	85.1	83.5	91.2	6.06	0.116	52.4
	independent validation (individual)	40.8	0.92 (0.82–1)	90.3 (85.1–93.9)	87 (71.1–94.8)	82.7 (67–92.6)	92.9 (87.4–95.8)	7.38 (2.8–19.4)	0.123 (0.078–0.192)	62 (19.9–193.6)
summary (retrospective datasets)		44.0	0.93 (0.88–0.99)	90.1 (87.5–92.3)	85.9 (74.3–92.8)	83.4 (72.8–91)	91.7 (88.3–93.9)	6.33 (3.46–11.6)	0.118 (0.093–0.151)	51.3 (26.1–100.9)
Unseen pathogen datasets	active TB versus viral independent validation	45.0	0.91 (0.89–0.93)	90.2	74	74	90.2	3.48	0.13	26.3
	bacterial versus HIV independent validation	77.8	0.94 (0.92–0.96)	90.4	85.6	95.6	71.8	6.28	0.11	55.9
	active TB versus HIV independent validation	77.4	0.9 (0.88–0.93)	90.2	64.8	89.8	65.9	2.56	0.15	17
Prospective validation datasets	Laos Fluidigm Cohort	46.1	0.96 (0.93–0.99)	96.2	87.9	87.2	96.5	7.95	0.043	184.9
	Nepal Cohort	77.5	0.91 (0.86–0.97)	90.7	76.0	92.9	70.4	3.78	0.122	31
summary (prospective datasets)		56.3	0.94 (0.89–1)	91.6 (69.4–98.1)	87.9 (81.7–92.3)	90.7 (83–94.3)	89 (67.5–97.4)	7.78 (5.04–12)	0.095 (0.02-0.4)	87 (18.3–413.4)

The AUROC and prevalence of bacterial infections were computed for each dataset, and other diagnostic statistics were computed for two different BoVI score cutoffs in each dataset. The cutoff was selected by eliminating all cutoffs with a sensitivity below 90% and then choosing the cutoff that maximized the sum of sensitivity and specificity. For independent validation (individual), summary (retrospective datasets), and summary (prospective datasets), the summary AUROC was calculated by pooling the bacterial versus viral AUROCs for each dataset (STAR Methods), the summary sensitivity, specificity, likelihood ratios, and DOR were calculated using the R package *mada*, the summary prevalence was calculated as the proportion of samples with bacterial infection across all the constituent datasets, and the NPV and PPV were calculated using the summary sensitivity/specificity and the summary prevalence. When combining statistics with *mada*, each individual cutoff was selected to maximize the sum of summary sensitivity and summary specificity. The 95% CI is included in parentheses for all relevant statistics. AUROC, area under the receiver operating characteristics curve; PPV, positive predictive value; NPV, negative predictive value; LR+, positive likelihood ratio; LR−, negative likelihood ratio; DOR, diagnostic odds ratio.

### Validation of the BoVI score in independent retrospective datasets

We further investigated the robustness and generalizability of the BoVI score in an additional 32 independent validation datasets (23 WB and 9 PBMC) consisting of 1,889 blood samples, none of which were used in either the training or hold-out validation sets (Figure 2). In these 32 COCONUT co-normalized datasets, each of the eight genes were significantly differentially expressed (Figure S3C). The BoVI score distinguished viral infections from extracellular and intracellular bacterial infections with AUROCs ranging from 0.936 to 0.962 (Figure 3C) and was similarly accurate across a variety of pathogens (Figure S4C). The BoVI score had equal accuracy in distinguishing extracellular or intracellular bacterial infections from viral infections irrespective of which and how many datasets and samples were used (Figure S5). At >90% sensitivity, the BoVI score had 85.1% specificity (Table 1), which again met the proposed diagnostic TPP. At 45.5% prevalence of bacterial infections in the 32 COCONUT-co-normalized validation datasets, the BoVI score had 83.5% PPV and 91.2% NPV (Table 1).

Next, we validated the BoVI score in five additional datasets composed of 431 samples that profiled patients with bacterial or viral infection but did not include any healthy controls (Table S6). In these five datasets, each of the 8 genes was significantly differentially expressed (Figure S3D). Across the datasets, the BoVI score distinguished bacterial and viral infections with high accuracy (summary AUROC = 0.917; 95% CI, 0.822–1.0; range, 0.878–0.955) (Figure 3D). At >90% sensitivity, the BoVI score had 87% specificity (Table 1).^79^ At 40.8% prevalence of bacterial infections in these five datasets, the BoVI score had 82.7% PPV and 92.9% NPV (Table 1).

In addition, to assess the ability of the BoVI score to generalize to pathogens that were not included in the discovery process, we tested the BoVI score in 14 datasets containing 429 samples with active TB (ATB) infection and 6 datasets containing 125 samples with HIV infection. Distinguishing TB from viral infections has been challenging to date. At 90% sensitivity, the BoVI score distinguished patients with ATB from other viral infections with ∼85% specificity (Figure 3E) but did not differentiate it from other bacterial infections (Figure 3F). The BoVI score distinguished these “unseen” pathogens from each other and from the other bacterial and viral pathogens with AUROCs ranging from 0.902 to 0.939 (Figures 3E and 3F; Table S8). Importantly, when we applied the 8-gene signature to only healthy controls from the studies that included patients with either TB or viral infection, it did not distinguish control cases (AUROC = 0.51; 95% CI, 0.453–0.568; Figure S6), demonstrating that, after COCONUT normalization, the discovery of the 8-gene signature is not affected by batch effects. These results suggest that the 8-gene signature could be integrated with a host-response signature for diagnosis of TB (e.g., a 3-gene signature for diagnosis of TB^26^ that has been translated and validated as a point-of-care test^80^^,^^81^^,^^82^^,^^83^) to further improve diagnosis of ATB.

Overall, across the 37 independent validation datasets composed of 2,320 samples, the BoVI score had a summary AUROC of 0.932 (95% CI, 0.875–0.989). At >90% sensitivity, the BoVI score had 85.9% specificity, a DOR of 51.3, and 83.4% PPV and 91.7% NPV at 44% prevalence of bacterial infections (Table 1). Hence, the BoVI score accurately distinguished patients with viral infection from those with bacterial infection, irrespective of extracellular or intracellular bacterial infection, and met the proposed diagnostic TPP.

### The BoVI score is robust to differences in age, sex, race, and days of fever

**Figure 4:**
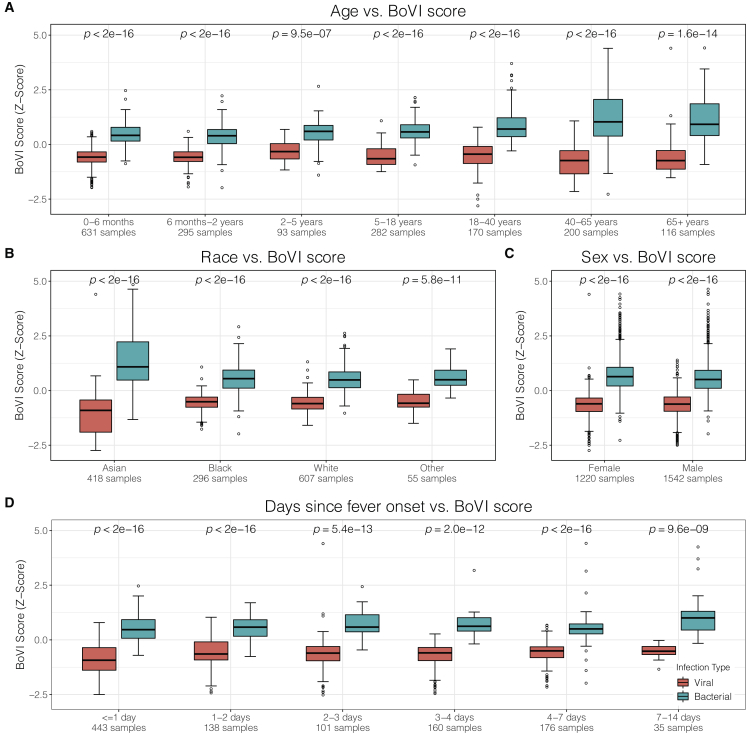
The BoVI signature maintains high accuracy regardless of age, race, sex, or days since fever onset Performance of the BoVI signature was calculated for each category across training, hold-out validation, and COCONUT co-normalized independent validation samples. Scores were centered at 0 and then the *Z* score was calculated. Centering and scaling was done separately for training/hold-out validation and for independent validation. The p values were calculated using the Mann-Whitney U test. (A) Boxplots of the BoVI score across each age category. Samples were used only if (1) the exact age was listed, (2) a range of ages was provided that fit completely into a single age window, or (3) the mean and SD of the ages was provided and all ages that were within one SD of the mean fit into a single age window. Overall, 1,787 samples had usable age information. (B) Boxplots of the BoVI score across each race category. Overall, there were 1,376 samples with information on race. (C) Boxplots of the BoVI score for either female or male patients. Overall, there were 2,762 samples with sex listed or with sex imputed using a set of Y-linked genes and a set of known X-escape genes, as described in Bongen et al.^84^ (D) Boxplots of the BoVI score across each day since fever onset category. Overall, there were 1,053 samples with information on day since fever onset. See also Figures S7–S10 and Table S9.

Several datasets provided detailed demographic information for individual patients including race, sex, age (or age range), and days since fever onset. We note that these datasets reported sex, not environmentally defined gender. In datasets where sex was not explicitly recorded, we imputed it using expression of a set of genes located on the Y chromosome and a set of known X-escape genes.^84^^,^^85^ Overall, we had data on age, race, sex, and days since fever onset for 1,787, 1,376, 2,762, and 1,053 samples, respectively, across the 69 datasets used in our analysis. Across a wide range of ages, from less than 6 months to more than 80 years, the BoVI score was consistently higher in patients with bacterial infection than in those with viral infections and distinguished them with high accuracy (AUROC range: 0.844–0.956 in training/hold-out validation, 0.878–0.97 in independent validation; Figures 4A and S7). The BoVI score also diagnosed whether a patient had a bacterial or viral infection with high accuracy irrespective of race (AUROC range: 0.911–0.970 in training/hold-out validation, 0.954–0.963 in independent validation; Figures 4B and S8) and sex (AUROC range: 0.927–0.947 in training/hold-out validation, 0.943–0.955 in independent validation; Figures 4C and S9). Finally, the BoVI score maintained high discriminative power irrespective of the number of days since fever onset (AUROC range: 0.872–1 in training/hold-out validation, 0.88–0.992 in independent validation; Figures 4D and S10). Across 1,053 samples for which age, race, and sex information was available, a multivariable logistic regression found that the BoVI score, age, and black patients had significantly positive associations with bacterial infection (Table S9).

### Validation of the BoVI score in two independent prospective cohorts using Fluidigm RT-PCR

**Figure 5:**
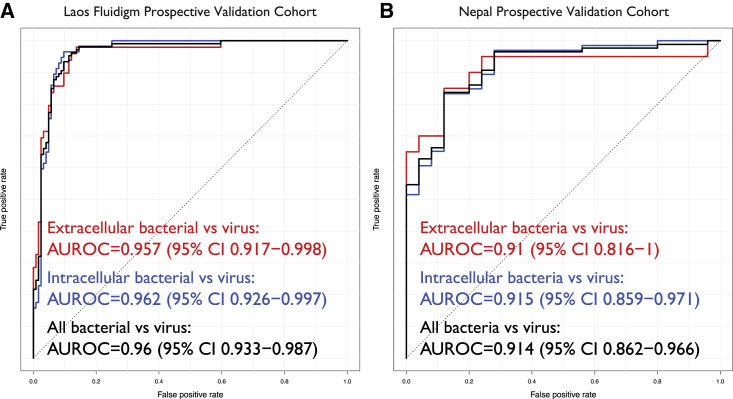
Performance of the BoVI signature in the Laos Fluidigm and Nepal prospective validation cohorts (A) AUROC using the BoVI signature in the Laos cohort. (B) AUROC using the BoVI signature in the Nepal cohort. Performance was assessed for three comparisons: extracellular bacterial infections versus viral infections (red), intracellular bacterial infections versus viral infections (blue), and all bacterial infections versus viral infections (black). See also Figure S11 and Tables S10–S12.

Finally, we validated the BoVI score in two cohorts of febrile patients in two countries, Laos (Table S10) and Nepal (Table S11), using Fluidigm RT-PCR. The main intracellular bacterial pathogens in these two cohorts included *O. tsutsugamushi*, *R. typhi*, *B. pseudomallei*, and *S.* Typhi. The Laos Fluidigm cohort consisted of 230 WB samples (49 extracellular bacterial infections, 57 intracellular bacterial infections, 124 viral infections) from febrile inpatients over the age of 15 years. We excluded any patients that had been enrolled in the Laos Microarray cohort. The BoVI score had an overall AUROC of 0.96 (95% CI, 0.933–0.987) for distinguishing bacterial and viral infections with virtually identical accuracy for distinguishing extracellular bacterial (AUROC = 0.957; 95% CI, 0.917–0.998) and intracellular bacterial (AUROC = 0.962; 95% CI, 0.926–0.997) infections from viral infections (Figure 5A), irrespective of the pathogen (Figure S11A). At 96.2% sensitivity, the BoVI score had 87.9% specificity and a DOR of 184.9. At 46.1% prevalence of bacterial infections in the Laos Fluidigm cohort, the BoVI score had 87.2% PPV and 96.5% NPV (Table 1).

The Nepal cohort consisted of 111 WB samples (15 extracellular bacterial, 71 intracellular bacterial, 25 viral) from individuals >12 months of age presenting to the hospital with a self-reported >72-h history of fever. The BoVI score distinguished bacterial and viral infections with an overall AUROC of 0.914 (95% CI, 0.862–0.966) with almost the same accuracy for extracellular bacterial (AUROC = 0.91; 95% CI, 0.816–1.0) and intracellular bacterial (AUROC = 0.915; 95% CI, 0.859–1.0) infections (Figure 5B), irrespective of the pathogen (Figure S11B). At >90% sensitivity, the BoVI score had 76% specificity and a DOR of 31 (Table 1). At 77.5% prevalence of bacterial infections in the Nepal cohort, the BoVI score had 92.9% PPV and 70.4% NPV (Table 1).

Together, across 341 samples in two prospective cohorts from two countries, the BoVI score had 91.6% specificity at 87.9% sensitivity and a DOR of 87, which met the diagnostic TPP. At 56.3% prevalence of bacterial infections in both cohorts combined, the BoVI score had 90.7% PPV and 89% NPV (Table 1).

## Discussion

Overuse and misuse of antimicrobial agents continues to drive antimicrobial resistance on a global scale. Antimicrobial agents are overused or misused for several reasons, including inappropriate broad coverage due to an incomplete understanding of local epidemiology and inadequate diagnostics. Our comparison of four host-response gene signatures for distinguishing bacterial and viral infections found that current signatures have significantly lower accuracy for distinguishing intracellular bacterial infections from viral infections, which limits their clinical utility to settings where these infections are not prevalent.^86^

Arguably, we could have grouped our datasets as from LMICs and non-LMICs to investigate generalizability of the four previously described host response-based signatures. However, datasets from non-LMICs had an over-representation of mild viral infections (e.g., rhinovirus), whereas datasets from LMICs had severe viral infections over-represented. In other words, grouping datasets by LMIC and non-LMIC settings was confounded by disease severity.

We applied MANATEE, a multi-cohort analysis framework, to address this challenge, and performed the largest multi-cohort analysis to date of blood transcriptome profiles from patients with bacterial or viral infections. Our analysis of 4,200 transcriptome profiles represented biological, clinical, and technical heterogeneity observed in the worldwide population by including substantial diversity of pathogens, host genetics, ethnic and racial backgrounds, and environmental exposures from different geographic regions, ages, sex, and clinical severity. We identified and extensively validated an 8-gene signature that distinguished both extracellular and intracellular bacterial infections from viral infections with high accuracy and met the proposed diagnostic TPP, despite broad heterogeneity in age, sex, race, days since fever onset, and severity of infection, demonstrating its robustness across heterogeneous clinical populations. The 8-gene signature had slightly lower accuracy in the Nepal cohort, which could be due to the large proportion of identified human herpesvirus 6 (HHV6)- and CMV-infected patients, viruses for which testing was not performed in the Lao cohorts. These viruses may be detected and have disease attributed to them even when they are not the causative agent of acute illness.^87^^,^^88^

Across 37 retrospective and 2 prospective cohorts used for validation, the BoVI score has low negative likelihood ratios (<0.12), high positive likelihood ratios (≥6.33), and high PPV and NPV (≥83%). These test characteristics are substantially higher than other host-response-based diagnostics,^89^ which suggest that it would be potentially effective as a rule-out and a rule-in test. Moreover, these test characteristics assume no knowledge of the patient and thus are only estimates of the real-world clinical utility of such a test, since patient history, physical examination, vital signs, and laboratory values would all assist in a diagnosis as well. Ultimately, only interventional trials will be able to establish the cost-effectiveness and clinical utility of a diagnostic test.

We validated the BoVI score in two prospective cohorts using Fluidigm RT-PCR, demonstrating that the score is robust. The parsimony of our 8-gene signature makes it an ideal candidate for translation to a point-of-care test as several commercial platforms capable of measuring multiple mRNAs in a short time are available.^90^ For example, the GeneXpert platform from Cepheid has successfully translated a 3-gene signature for diagnosis of active tuberculosis^26^ into a point-of-care diagnostic cartridge with high accuracy in a prospective cohort.^27^^,^^28^

The identity of the genes in our signature is consistent with the known biology of the response to infection. *ICAM1* is a well-studied cell surface glycoprotein that is expressed by many immune cells and is upregulated in response to lipopolysaccharide (LPS) exposure.^91^ *SMARCD3* and *EBI3*, two genes over-expressed in bacterial infection, are associated with tuberculosis infection and bacterial burden.^92^^,^^93^ Of the viral genes, *IFI27* and *JUP* are known to be upregulated in response to viral infection.^42^^,^^94^ Furthermore, *FCER1A* encodes a subunit of the immunoglobulin (Ig) E receptor and is thought to be involved in the immune response to viral infections.^95^ Hence, selection of these transcripts as biomarkers for distinguishing bacterial and viral infections makes sense biologically based on known features of host immune response to the type of pathogens.

### Limitations of the study

Our study has a few limitations. First, COCONUT co-normalization may have removed some biological variation between the immune responses measured in different patient groups. However, our results demonstrate that the effects of acute bacterial or viral infection on the host immune response are robustly detectable across cohorts, even in datasets that were separately co-normalized or datasets that were not co-normalized. Second, we validated the 8-gene signature in two independent cohorts that used only microbiologically confirmed patients in case-control design and did not establish accuracy of the 8-gene signature against current biomarkers of bacterial infection such as CRP and PCT. The performance characteristics of the 8-gene signature will differ in patient cohorts that rely on non-microbiological adjudication methods, which are known to suffer from high inter-rater variations, leading to a variable standard. We were also not able to assess the positive predictive value for subsequent microbiological confirmation of either bacterial or viral infections. The 8-gene signature should be validated in additional prospective cohorts from other populations and where microbiological diagnosis is not available. Third, although we demonstrated that the 8-gene signature can distinguish TB from viral infections, it was dependent on merging datasets that included either viral or TB cases. The 8-gene signature should be validated in future studies that include both classes within the same study. Fourth, there were no TB, malaria, or other protozoal/fungal infection cases in the two prospective cohorts, although we did not systematically exclude them. However, these infections can present as an acute febrile illness. Hence, the 8-gene signature should be validated in the future studies that include these infections.

In summary, by leveraging the large amount of heterogeneity present across tens of independent datasets composed of thousands of blood samples, we ensured that our data were representative of real-world global patient populations. In turn, we utilized biological, clinical, and technical heterogeneity in these cohorts to identify a parsimonious and robust host-response-based 8-gene signature for distinguishing patients with bacterial infections from those with viral infections that generalizes to global populations. The signature consistently met the TPP for distinguishing bacterial and viral infections in both retrospective and prospective cohorts. Its parsimony makes it an ideal candidate for translation to a point-of-care test, which in turn has the potential to improve targeted patient therapy and to help reduce the overuse of antimicrobial drugs.

## STAR★Methods

### Key resources table

**Table:** 

REAGENT or RESOURCE	SOURCE	IDENTIFIER
**Deposited data**		

RNA-seq dataset	Turner et al.^96^	ArrayExpress: E-MTAB-8290
RNA-seq dataset	Eckold et al.^97^	GEO: GSE114192
RNA-seq dataset	Sambarey et al.^92^	GEO: GSE122485
RNA-seq dataset	Singhania et al.^98^	GEO: GSE107995
RNA-seq dataset	N/A (no associated paper)	GEO: GSE69597
Microarray dataset	N/A (no associated paper)	GEO: GSE206829
Microarray dataset	Mahajan et al.^71^	GEO: GSE64456
Microarray dataset	Lindow et al.^44^	GEO: GSE72946
Microarray dataset	Pollard and Blohmke^99^	GEO: E-MTAB-3423
Microarray dataset	Schreiber^100^	ArrayExpress: E-MTAB-852
Microarray dataset	Simmons et al.^63^	GEO: GSE40628
Microarray dataset	de Steenhuijsen Piters et al.^39^	GEO: GSE77087
Microarray dataset	Wong et al.^69^	GEO: GSE4607
Microarray dataset	Hu et al.^54^	GEO: GSE40396
Microarray dataset	Herberg et al.^41^	GEO: GSE42026
Microarray dataset	Bermejo-Martin et al.^50^	GEO: GSE21802
Microarray dataset	Zhai et al.^70^	GEO: GSE68310
Microarray dataset	Parnell et al.^72^	GEO: GSE20346
Microarray dataset	Parnell et al.^36^	GEO: GSE40012
Microarray dataset	Sweeney et al.^101^	GEO: GSE66099
Microarray dataset	Suarez et al.^15^	GEO: GSE60244
Microarray dataset	N/A (no associated paper)	GEO: GSE11755
Microarray dataset	Sutherland et al.^65^	GEO: GSE28750
Microarray dataset	Thuny et al.^67^	GEO: GSE29161
Microarray dataset	Ahn et al.^49^	GEO: GSE33341
Microarray dataset	Lill et al.^59^	GEO: GSE40586
Microarray dataset	Pankla et al.^61^	GEO: GSE69528
Microarray dataset	van den Ham^102^	ArrayExpress: E-MTAB-3162
Microarray dataset	Smith et al.^64^	GEO: GSE25504
Microarray dataset	Pankla et al.^61^	GEO: GSE13015
Microarray dataset	Mejias et al.^46^	GEO: GSE38900
Microarray dataset	Heinonen et al.^40^	GEO: GSE67059
Microarray dataset	Thompson et al.^24^	GEO: GSE7000
Microarray dataset	Kwissa et al.^58^	GEO: GSE51808
Microarray dataset	Irwin et al.^55^	ArrayExpress: E-MEXP-3567
Microarray dataset	Zaas et al.^16^	GEO: GSE17156
Microarray dataset	Rodriguez-Fernandez et al.^47^	GEO: GSE103842
Microarray dataset	Banchereau et al.^37^	GEO: GSE30119
Microarray dataset	Popper et al.^62^	GEO: GSE96656
Microarray dataset	Kulohoma et al.^57^	GEO: GSE47172
Microarray dataset	Herberg et al.^14^	GEO: GSE80496
Microarray dataset	Tang et al.^48^	GEO: GSE82050
Microarray dataset	Andeweg^103^	ArrayExpress: E-MTAB-5195
Microarray dataset	Liu et al.^45^	GEO: GSE73072
Microarray dataset	Cvijanovich et al.^53^	GEO: GSE9692
Microarray dataset	Berry et al.^51^	GEO: GSE19491
Microarray dataset	Jaggi et al.^56^	GEO: GSE68004
Microarray dataset	Berdal et al.^38^	GEO: GSE27131
Microarray dataset	Popper et al.^33^	GEO: GSE38246
Microarray dataset	Nascimento et al.^60^	GEO: GSE18090
Microarray dataset	Ramilo et al.^73^	GEO: GSE6269
Microarray dataset	Ioannidis et al.^42^	GEO: GSE34205
Microarray dataset	Liu et al.^104^	GEO: GSE23140
Microarray dataset	Ardura et al.^35^	GEO: GSE16129
Microarray dataset	Tantibhedhyangkul et al.^66^	GEO: GSE16463
Microarray dataset	Chaussabel et al.^52^	GEO: GSE11907
Microarray dataset	Herberg et al.^14^	GEO: GSE72809
Microarray dataset	Herberg et al.^14^	GEO: GSE72810
Microarray dataset	Tsalik et al.^76^	GEO: GSE63990
Microarray dataset	Bloom et al.^105^	GEO: GSE42834
Microarray dataset	Maertzdorf et al.^106^	GEO: GSE28623
Microarray dataset	Maertzdorf et al.^107^	GEO: GSE34608
Microarray dataset	Verhagen et al.^108^	GEO: GSE41055
Microarray dataset	Blankley et al.^109^	GEO: GSE83456
Microarray dataset	N/A (no associated paper)	GEO: GSE81746
Microarray dataset	Maertzdorf et al.^110^	ArrayExpress: E-GEOD-25534
Microarray dataset	Noursadeghi et al.^111^	ArrayExpress: E-MTAB-3260
Microarray dataset	Rinchai et al.^112^	GEO: GSE100150
Microarray dataset	N/A (no associated paper)	GEO: GSE29429
Microarray dataset	Park et al.^113^	GEO: GSE29536
Microarray dataset	Montano et al.^114^	GEO: GSE4124
Microarray dataset	Singh et al.^115^	GEO: GSE77939

**Oligonucleotides**		

Taqman gene expression assays	See Table S12	See Table S12

**Software and algorithms**		

R	R Core Team^116^	https://www.r-project.org/
gcRMA	Wu and Irizarry^117^	https://www.bioconductor.org/packages/release/bioc/html/gcrma.html
DESeq2	Love et al.^118^	https://bioconductor.org/packages/release/bioc/html/DESeq2.html
MetaIntegrator	Haynes et al.^119^	https://cran.r-project.org/web/packages/MetaIntegrator/index.html
COCONUT	Sweeney et al.^74^	https://cran.r-project.org/web/packages/COCONUT/index.html
ROCR	Sing et al.^120^	https://cran.r-project.org/web/packages/ROCR/index.html
rmeta	Lumley^121^	https://cran.r-project.org/web/packages/rmeta/index.html
OptimalCutpoints	López-Ratón et al.^122^	https://cran.r-project.org/web/packages/OptimalCutpoints/index.html
glmnet	Friedman et al.^123^	https://cran.r-project.org/web/packages/glmnet/index.html
samr	Tusher et al.^124^	https://cran.r-project.org/web/packages/samr/index.html

### Resource availability

#### Lead contact

Further information and requests for resources, software, and data should be directed to and will be fulfilled by the lead contact, Purvesh Khatri (pkhatri@stanford.edu).

#### Materials availability

This study did not generate new unique reagents.

### Experimental model and subject details

#### Search for gene expression datasets

We performed a search in NIH Gene Expression Omnibus (GEO) and European Bioinformatics Institute (EBI) ArrayExpress for public human microarray genome-wide expression studies of bacterial or viral infections.^31^^,^^32^ We excluded datasets that (i) were nonclinical, (ii) were profiled using tissues other than WB or PBMCs, (iii) did not have samples from at least 3 healthy controls, or (iv) did not provide information to identify whether a patient had bacterial or viral infection.

We renormalized Affymetrix arrays using GC robust multiarray average (gcRMA) (on arrays with mismatch probes) or RMA. We renormalized Illumina, Agilent, GE, and other commercial arrays using normal-exponential background correction followed by quantile normalization. We did not renormalize the custom arrays. After log_2_-transformation of normalized expression data, we used a fixed-effect model to summarize probes to genes within each study. Within each study, cohorts assayed with different microarray types were treated as independent datasets.

#### Laos cohorts

Patients were recruited at Mahosot Hospital, Vientiane, Laos between July 2007 and March 2009 as part of a study of the etiology of infectious febrile illness.^125^ All adults (>15 years of age) with tympanic temperature >37.5°C and suspected septicemia were enrolled if they provided written informed consent. Ethical clearance was granted by the former Faculty of Medical Sciences Ethical Review Committee (now University of Health Sciences Ethics Committee), National University of Laos, the Oxford University Tropical Ethics Committee, and the Stanford University Institutional Review Board.

Venous blood samples were drawn for blood culture and for other diagnostic tests. Positive blood cultures were identified as described in Phetsouvanh et al.^125^ Targeted PCR tests were performed for specific pathogens known to be of importance in this hospital population, including *O. tsutsugamushi, Rickettsia* spp., *Leptospira* spp., *dengue virus*, and *Japanese encephalitis virus* (Mayxay et al.^2^). A positive case was defined as CT below 40 (single sample) with appropriate results for controls. Patients without any positive diagnosis were excluded from the analysis. Whole blood for RNA was collected into PAXgene tubes, stored at room temperature for 2 h and then stored at 4°C for up to four days. RNA was extracted using Qiagen PAXgene Blood RNA kit according to manufacturer’s direction, stored at −80°C, and shipped to Stanford University on dry ice.

#### Nepal cohort

We recruited all individuals >12 months of age presenting to Dhulikhel Hospital, Kathmandu University Hospital, Kavrepalanchok, Nepal with a self-reported >72-h history of fever. Participants were recruited from outpatient departments, emergency department, and inpatient wards. After obtaining informed consent, we administered a standardized questionnaire to each participant or adult guardian to ascertain demographic and clinical characteristics. Ethical clearance was granted by the Nepal Health Research Council and the Stanford University Institutional Review Board. A venous blood specimen was collected from each participant and transferred into PAXgene RNA tubes, EDTA tubes, and Bactec blood culture bottles. Blood culture was performed using a Bactec automated culture system, and bacterial identification was performed using biochemical testing.^5^

We performed real-time PCR for Rickettsia, Leptospira, HHV6, CMV, Parvovirus B19, and enteroviruses using previously published protocols.^126^^,^^127^^,^^128^^,^^129^^,^^130^^,^^131^^,^^132^ Patients without any positive diagnosis were excluded from analysis.

### Method details

Methods for signature discovery and validation, including co-normalization using COCONUT, identifying 8-gene signature using a multi-cohort analysis framework, and a classifier are described below.

### Quantification and statistical analysis

#### COCONUT co-normalization

Despite availability of 64 independent datasets that profiled respiratory infections, there were only three cohorts that contained intracellular bacterial, extracellular bacterial, and viral infections. Furthermore, two of these datasets had ≤2 intracellular bacterial infections. This made it virtually impossible to conduct within-dataset analyses, so we instead decided to perform an analysis across all the datasets. However, because of the difference in background measurements for each dataset (owing to the use of different platforms), we were concerned about skewed results due to the batch effects. In order to make use of these data, we used Combat CO-Normalization Using conTrols or COCONUT.^74^ ComBat only assumes that the data have been normalized and expression values have been estimated for all genes and samples. Prior to running COCONUT, we ensured that all datasets were normalized as described above in the subsection search for gene expression datasets. COCONUT allows for co-normalization of expression data without changing the distribution of genes between studies and without any bias towards sample diagnosis. It applies a modified version of the ComBat empirical Bayes normalization method^133^ that assumes an equal distribution only between control samples. Briefly, the healthy controls from each cohort undergo ComBat co-normalization without covariates, and the ComBat estimated parameters are acquired for each dataset’s healthy samples. These parameters are then applied to the disease samples in each dataset, which causes all samples to assume the same background distribution while still retaining within-dataset differences between healthy and disease samples in each dataset. We have previously shown that when COCONUT co-normalization is applied, housekeeping genes remain invariant across diseases and cohorts, and each gene retains the same distribution between diseases and controls within each dataset.^74^

When validating published signatures (see below), we co-normalized all 64 datasets and performed analyses across all datasets. However, for signature discovery the data were randomly assigned to one of three groups (training, hold-out validation, or independent validation; described below) and each group was independently batch-corrected using COCONUT co-normalization. Additional statistical details can be found in Figure 2.

#### Validation of published signatures

We chose 4 signatures to investigate whether they generalize to global patient population or not based on a recently described systematic comparison of 28 host gene expression signatures, following PRISMA guidelines, for discrimination of bacterial and viral infections using 51 datasets by Bodkin et al*.*^134^ Only 7 out of the 51 datasets used by Bodkin et al. were from low-and-middle income countries (LMICs), 3 of which included only patients with viral infections. In other words, Bodkin et al. did not have sufficient datasets (only 4 out of 51 datasets from LMICs) to investigate whether these 28 signatures are generalizable to global population or not.

Given the comprehensive analysis by Bodkin et al., we chose signatures that had high accuracy for diagnosis of bacterial infections according to Bodkin et al. We reasoned that if a signature with higher accuracy for bacterial infection in Western Europe and North America (non-LMICs) does not generalize to LMICs, a signature with lower accuracy will not generalize either (because even if it has higher accuracy in LMICs, it has lower accuracy in non-LMICs). Bodkin et al. further found that signatures with smaller number of genes had lower accuracy than those with higher number of genes. Therefore, we selected a set of signatures with a varying number of genes with high accuracy to test our hypothesis.

Finally, we prioritized signatures that are under active development for translation to clinical practice to demonstrate that none of them will address the global health challenge of discriminating bacterial vs viral infections. To the best of our knowledge, the signatures under active development are Herberg2, Sweeney7, Mayhew29^135^ and Tsalik120. Among these, Mayhew29 includes all genes from Sweeney7 and had only 1% higher AUROC.

We also excluded signatures from comparison if (i) the underlying model could not be recreated, either because the training data were inaccessible or because not enough details about the training procedure were available; (ii) the model was unable to be applied to a new dataset without re-training (e.g., *kNN*-based classifiers); or (iii) the model did not produce a continuous score. For Sweeney7, we used a previously described bacterial/viral metascore to perform classification (Equation 1). For Herberg2 and Sampson4, we used the described Disease Risk Score^136^ to perform classification (Equation 2). For Tsalik120, we recreated their Least Absolute Shrinkage and Selection Operator (LASSO) models^137^ using the provided coefficients and then calculated the score as the ratio of the linear outputs of the bacterial and viral LASSOs (Equation 3). We removed two predictors from the Tsalik120 model because they had no existing mapping to a gene symbol and thus could not be used in any dataset that was not run with the original microarray platform. Since COCONUT requires genes to be measured in all datasets, for each signature we removed any datasets that would cause a significant number of signature genes to be excluded. In the COCONUT co-normalized data, no signatures were missing any genes other than Tsalik120, which was missing 17/120 genes. In the Laos Microarray cohort, Tsalik120 was missing two genes and Sampson4 was missing one gene.

To ensure that the decreased accuracy for intracellular bacterial infections that we observed for all four published signatures was not simply due to the specific subset of datasets that we chose for each signature, we also analyzed the difference in performance for different subsets of 64 datasets. Because there were too many combinations for an exhaustive analysis, we randomly sampled 1,396,677 combinations from all possible combinations. To reduce noise, we only included combinations where each infection group had at least five samples, at least one of the two AUROCs was greater than 0.6, and where at least half of the signature genes were present. Additionally, to ensure that this phenomenon was not driven by the number of genes present in Tsalik120, we also computed the difference in performance compared to the number of missing genes. Additional statistical details can be found in Figure 1.

(Equation 1) $$S_{i}={\left(\prod_{gene\in pos}x_{i}\left(gene\right)\right)}^{\frac{1}{\left|pos\right|}}-{\left(\prod_{gene\in neg}x_{i}\left(gene\right)\right)}^{\frac{1}{\left|neg\right|}}\cdot \frac{\left|neg\right|}{\left|pos\right|}$$

(Equation 2) $$S_{i}=\left(\sum_{gene\in pos}x_{i}\left(gene\right)\right)-\left(\sum_{gene\in neg}x_{i}\left(gene\right)\right)$$

(Equation 3) $$S_{i}=\frac{lasso_{bac}\left(i\right)}{lasso_{vir}\left(i\right)}$$

#### Derivation of the 8-gene signature with MANATEE

We developed Multi-cohort ANalysis of AggregaTed gEne Expression (MANATEE) to include biological, clinical, and technical heterogeneity representative of the global patient population across heterogeneous independent datasets. After collecting and curating relevant datasets from public repositories through a search, we selected a subset of those datasets for the discovery of a gene signature, referred to as “training data”, and the rest were set aside for validation of the gene signature, referred to as “independent validation datasets.” We randomly divided the training data such that 70% of the data were used for discovery of the gene signature and the remaining 30% were used as hold-out validation data. Both the training and hold-out data were independently batch-corrected using COCONUT.

We calculated four measures of differential expression between cases and controls for each gene using the training data: (1) the Significant Analysis of Microarray (SAM) score from SAM,^124^ (2) the corresponding SAM local FDR, (3) the Benjamini-Hochberg FDR corrected p value from a t-test,^138^ and (4) Hedge’s adjusted *g* that accounts for small sample bias^139^^,^^140^^,^^141^ as the effect size (ES).

To ensure that resulting signatures are generalizable and not affected by unknown confounders that may be present in individual datasets, we performed a leave-one-study-out (LOSO) analysis, wherein we iteratively removed each study that accounted for at least 5% of the training samples from the training set and re-calculated each of the four differential expression statistics for each iteration of the training set. This LOSO iterative analysis ensured that any gene selected to be a part of the signature met the selection criteria irrespective of which study was removed. This strategy prevented a single study from exerting too strong of an effect on the selection of genes.^142^^,^^143^ We have recently showed that classification signatures identified using LOSO tend to be less overfit than signatures identified using k-fold cross validation.^135^

Next, we applied three different differential expression thresholds: 1) ES ≥ 0.6 & FDR ≤1%; 2) ES ≥ 0.8 & FDR ≤1%; and 3) top 100 SAM scores. For the SAM score threshold, all genes with a local SAM FDR ≤1% were ranked by their SAM score, and the top 100 genes from that list were selected. After applying the differential expression filters, several feature selection methods could be used to further narrow down which genes were selected. Here, we used three methods: 1) greedy forward search; 2) greedy backward search; and 3) abridged best subset selection (abridged BSS; described below). We selected two signatures from each abridged BSS: (1) the signature with the maximum AUROC in the training data, and (2) the signature with the fewest number of genes but with AUROC within the 95% confidence interval of the maximum AUROC signature. Additional statistical details can be found in Figure 2.

**Abridged Best Subset Selection** method combines a greedy backward search with an exhaustive search to perform as exhaustive a search as possible within a reasonable computational time. Performing a greedy search alone would be computationally feasible, but because of the nature of the greedy algorithm it does not ensure that one finds the best possible combination of genes for diagnostic purposes. In contrast, best subset selection is an exhaustive search that will always select the optimal combination of genes; however, the computational cost of best subset selection increases exponentially with the number of genes, which makes it infeasible to execute on more than ∼20 genes. The Abridged Best Subset Selection (Abridged BSS) combines the strengths of both methods.

First, we ran a greedy backward search on the initial gene list. Briefly, we took the starting gene set and calculated the AUROC after individually removing each of the genes. We identified the gene whose removal leads to the largest increase in AUROC, and then permanently remove that gene from the set. We then applied this same strategy to the new gene set, once again removing the gene whose exclusion resulted in the largest increase in AUROC. In a normal greedy backward search, we would continue repeating this step until we reach a point such that removing any gene resulted in a loss of AUROC that is greater than some pre-defined threshold. However, in this case, the greedy backward search was simply run until 20 genes remained.

Once we had a smaller set of genes, best subset selection could be run on the abridged gene list. Briefly, we assessed the diagnostic power of every possible combination of the genes by calculating the signature scores for each combination and reporting the corresponding AUROC. Next, for every unique number of total genes, the subset of genes that produced the best AUROC was reported. This resulted in a list of the best signatures for each number of total genes, from which the final gene signature was selected.

We tested each signature in the hold-out validation data. While the samples in the hold-out validation data came from the same datasets as the training data, the samples themselves were different and were co-normalized independently. This allowed us to acquire a semi-independent assessment of each signature’s diagnostic accuracy. In addition, the drop-off in performance of each signature between training and hold-out validation could be used as a proxy for estimating the degree to which each signature is overfit.

We selected an 8-gene signature that was derived by filtering out the top 100 genes from SAM, running an abridged BSS, and then selecting the cutoff with the minimum number of genes while still being within the 95% confidence interval of the max AUROC. We then assessed the accuracy of this 8-gene signature in the 37 independent validation datasets, which were set aside at the beginning of the analysis. Here, out of the 37 datasets, 32 were COCONUT co-normalized; the remaining 5 datasets, which did not contain healthy samples, were not COCONUT co-normalized. The validation datasets were completely independent from those included in the training and hold-out validation sets, which allowed assessment of a signature in more heterogeneous and independent cohorts to ensure that it was not confounded by and is generalizable across a broad spectrum of patient populations, even when applied to samples containing previously unseen clinical, biological, and technical heterogeneity. Finally, we validated the 8-gene signature in two prospective cohorts from two countries (Nepal and Laos; described below) to directly assess its real-world diagnostic accuracy. Additional statistical details can be found in Table S7.

#### Calculation of the BoVI score

In order to perform disease classification, we defined a BoVI score (*S*_*i*_) for a sample based on our previously described signature score.^26^^,^^74^ The BoVI score is defined as the geometric mean of the genes over-expressed in bacterial infection minus the geometric mean of the genes over-expressed in viral infection (Equation 4).

(Equation 4) $$S_{i}={\left(\prod_{gene\in pos}x_{i}\left(gene\right)\right)}^{\frac{1}{\left|pos\right|}}-{\left(\prod_{gene\in neg}x_{i}\left(gene\right)\right)}^{\frac{1}{\left|neg\right|}}$$

#### Summary ROC curves

Summary ROC curves represent a weighted average of multiple independent ROC curves. When fewer than 5 curves were present, we generated unsmoothed summary ROC curves as follows. We used linear interpolation to approximate the true positive rate (TPR) values for each curve, so that TPR values could be aligned without changing the corresponding false positive rate (FPR) values. For each curve, we assigned weights based on the number of samples used to create the corresponding curve and calculated a summary ROC curve by taking the weighted mean of the corresponding TPR values. In addition, we calculated the weighted SD for each TPR, which was represented by a gray area on the plot. Finally, we calculated the summary AUROC using the trapezoid rule and the 95% confidence interval using the pooled SE of the individual curves. When greater than 5 curves were present, we generated a smoothed summary ROC curve using a modified version of the method of Kester and Buntinx^144^ as previously described.^74^ Summary ROC curve is used in Figure 3D.

#### Selecting optimal cutoffs with the Youden Index

When a discrete signature score cutoff needed to be selected (e.g., for calculating sensitivity/specificity), the Youden Index was sometimes used to identify an optimal cutoff. The Youden Index is defined as the cutoff which maximizes the sum of sensitivity and specificity.^145^^,^^146^

#### Whole genome transcript profiling

We performed human whole genome transcript profiling in the Laos Microarray cohort. We amplified total RNA using the TargetAmp Aminoallyl aRNA amplification kit (Epicentre, Madison, CA). We labeled eight ug of amplified RNA and 5 ug of amplified Universal Human Reference RNA (Agilent, Santa Clara, CA) using Cy5 and Cy3 dyes, respectively, and hybridized to Human Exonic Evidence Based Oligonucleotide (HEEBO) microarrays. Details of the protocol have been described previously.^33^ HEEBO microarrays, containing 44,544 probes, were printed by the Stanford Functional Genomics Core Facility.

We filtered data to exclude control probes, calculated background measurements using the Edwards correction, and then normalized data using a 2D spatial loess estimation.^147^ For replicate probes, we averaged the expression values.

#### Fluidigm RT-PCR validation

We validated the 8-gene signature Fluidigm RT-PCR using samples from the prospective cohorts from Laos and Nepal. There was no overlap between the patients enrolled in the Laos Microarray and Laos Fluidigm cohorts. AROS Applied Biotechnology (Aarhus, Denmark) carried out cDNA synthesis and qPCR using the Fluidigm platform. TaqMan assays and the corresponding genes are listed in Table S12. TaqMan assays were normalized using the geometric mean of three housekeeping genes: *18S, ACTB*, and *KPNA6*. We calculated the BoVI score using the geometric mean of the Ct values, as described above.

## Data Availability

• All datasets, software, and algorithms used in this study are publicly available and listed in the Key resources table. • This paper did not generate any unique datasets or code. • Any additional information required to reanalyze the data reported in this paper is available from the lead contact upon request.

## Supplemental information

Document S1. Figures S1–S11 and Tables S1–S12

Document S2. Article plus supplemental information

## References

[1] Crump J.A., Morrissey A.B., Nicholson W.L., Massung R.F., Stoddard R.A., Galloway R.L., Ooi E.E., Maro V.P., Saganda W., Kinabo G.D. (2013). Etiology of severe non-malaria febrile illness in Northern Tanzania: a prospective cohort study. PLoS Negl. Trop. Dis..

[2] Mayxay M., Castonguay-Vanier J., Chansamouth V., Dubot-Pérès A., Paris D.H., Phetsouvanh R., Tangkhabuanbutra J., Douangdala P., Inthalath S., Souvannasing P. (2013). Causes of non-malarial fever in Laos: a prospective study. Lancet. Glob. Health.

[3] Fleming-Dutra K.E., Hersh A.L., Shapiro D.J., Bartoces M., Enns E.A., File T.M., Finkelstein J.A., Gerber J.S., Hyun D.Y., Linder J.A. (2016). Prevalence of inappropriate antibiotic prescriptions among US Ambulatory care Visits, 2010-2011. JAMA.

[4] Silverman M., Povitz M., Sontrop J.M., Shariff S.Z. (2017). Antibiotic prescribing for Nonbacterial acute Upper respiratory infections in Elderly Persons. Ann. Intern. Med..

[5] Andrews J.R., Vaidya K., Bern C., Tamrakar D., Wen S., Madhup S., Shrestha R., Karmacharya B., Amatya B., Koju R. (2018). High rates of enteric fever diagnosis and lower burden of culture-confirmed disease in Peri-urban and Rural Nepal. J. Infect. Dis..

[6] Gwimile J.J., Shekalaghe S.A., Kapanda G.N., Kisanga E.R. (2012). Antibiotic prescribing practice in management of cough and/or diarrhoea in Moshi Municipality, Northern Tanzania: cross-sectional descriptive study. Pan Afr. Med. J..

[7] Laxminarayan R., Duse A., Wattal C., Zaidi A.K.M., Wertheim H.F.L., Sumpradit N., Vlieghe E., Hara G.L., Gould I.M., Goossens H. (2013). Antibiotic resistance-the need for global solutions. Lancet Infect. Dis..

[8] Andrews J.R., Qamar F.N., Charles R.C., Ryan E.T. (2018). Extensively Drug-Resistant typhoid — are Conjugate Vaccines Arriving just in time?. N. Engl. J. Med..

[9] Dittrich S., Tadesse B.T., Moussy F., Chua A., Zorzet A., Tängdén T., Dolinger D.L., Page A.L., Crump J.A., D'Acremont V. (2016). Target product profile for a diagnostic assay to differentiate between bacterial and non-bacterial infections and reduce antimicrobial overuse in Resource-limited settings: an Expert Consensus. PLoS One.

[10] Hogan C.A., Yang S., Garner O.B., Green D.A., Gomez C.A., Dien Bard J., Pinsky B.A., Banaei N. (2021). Clinical Impact of metagenomic next-generation sequencing of plasma cell-Free DNA for the diagnosis of infectious diseases: a Multicenter retrospective cohort study. Clin. Infect. Dis..

[11] Wilson M.R., Naccache S.N., Samayoa E., Biagtan M., Bashir H., Yu G., Salamat S.M., Somasekar S., Federman S., Miller S. (2014). Actionable diagnosis of neuroleptospirosis by next-generation sequencing. N. Engl. J. Med..

[12] Babady N.E. (2021). Clinical metagenomics for Bloodstream infections: is the Juice Worth the Squeeze?. Clin. Infect. Dis..

[13] Watson J., Salisbury C., Whiting P., Banks J., Pyne Y., Hamilton W. (2019). Added value and cascade effects of inflammatory marker tests in UK primary care: a cohort study from the Clinical Practice Research Datalink. Br. J. Gen. Pract..

[14] Herberg J.A., Kaforou M., Wright V.J., Shailes H., Eleftherohorinou H., Hoggart C.J., Cebey-López M., Carter M.J., Janes V.A., Gormley S. (2016). Diagnostic test accuracy of a 2-transcript host RNA signature for discriminating bacterial vs viral infection in febrile children. JAMA.

[15] Suarez N.M., Bunsow E., Falsey A.R., Walsh E.E., Mejias A., Ramilo O. (2015). Superiority of transcriptional profiling over procalcitonin for distinguishing bacterial from viral lower respiratory tract infections in hospitalized adults. JID (J. Infect. Dis.).

[16] Zaas A.K., Chen M., Varkey J., Veldman T., Hero A.O., Lucas J., Huang Y., Turner R., Gilbert A., Lambkin-Williams R. (2009). Gene expression signatures diagnose influenza and other symptomatic respiratory viral infections in humans. Cell Host Microbe.

[17] Lubell Y., Althaus T., Blacksell S.D., Paris D.H., Mayxay M., Pan-Ngum W., White L.J., Day N.P.J., Newton P.N. (2016). Modelling the Impact and cost-effectiveness of biomarker tests as compared with pathogen-specific diagnostics in the management of Undifferentiated fever in Remote Tropical settings. PLoS One.

[18] Prasad N., Murdoch D.R., Reyburn H., Crump J.A. (2015). Etiology of severe febrile illness in low- and middle-income countries: a systematic review. PLoS One.

[19] Rudd K.E., Johnson S.C., Agesa K.M., Shackelford K.A., Tsoi D., Kievlan D.R., Colombara D.V., Ikuta K.S., Kissoon N., Finfer S. (2020). Global, regional, and national sepsis incidence and mortality, 1990-2017: analysis for the Global Burden of Disease Study. Lancet.

[20] GBD 2017 Typhoid and Paratyphoid Collaborators (2019). The global burden of typhoid and paratyphoid fevers: a systematic analysis for the Global Burden of Disease Study 2017. Lancet Infect. Dis..

[21] Bhatt S., Gething P.W., Brady O.J., Messina J.P., Farlow A.W., Moyes C.L., Drake J.M., Brownstein J.S., Hoen A.G., Sankoh O. (2013). The global distribution and burden of dengue. Nature.

[22] Bonell A., Lubell Y., Newton P.N., Crump J.A., Paris D.H. (2017). Estimating the burden of scrub typhus: a systematic review. PLoS Negl. Trop. Dis..

[23] Blohmke C.J., Darton T.C., Jones C., Suarez N.M., Waddington C.S., Angus B., Zhou L., Hill J., Clare S., Kane L. (2016). Interferon-driven alterations of the host’s amino acid metabolism in the pathogenesis of typhoid fever. J. Exp. Med..

[24] Thompson L.J., Dunstan S.J., Dolecek C., Perkins T., House D., Dougan G., Nguyen T.H., Tran T.P.L., Doan C.D., Le T.P. (2009). Transcriptional response in the peripheral blood of patients infected with Salmonella enterica serovar Typhi. Proc. Natl. Acad. Sci. USA.

[25] Suliman S., Thompson E.G., Sutherland J., Weiner J., Ota M.O.C., Shankar S., Penn-Nicholson A., Thiel B., Erasmus M., Maertzdorf J. (2018). Four-gene pan-African blood signature predicts progression to tuberculosis. Am. J. Respir. Crit. Care Med..

[26] Sweeney T.E., Braviak L., Tato C.M., Khatri P. (2016). Genome-wide expression for diagnosis of pulmonary tuberculosis: a multicohort analysis. Lancet Respir. Med..

[27] Södersten E., Ongarello S., Mantsoki A., Wyss R., Persing D.H., Banderby S. (2020). Diagnostic accuracy study of a novel blood-based assay for identification of TB in people living with HIV. J. Clin. Microbiol..

[28] Sutherland J.S., van der Spuy G., Gindeh A., Thuong N.T.T., Namuganga A., Owolabi O., Mayanja-Kizza H., Nsereko M., Thwaites G., Winter J. (2022). Diagnostic accuracy of the Cepheid 3-gene host response fingerstick blood test in a prospective, multi-site study: interim results. Clin. Infect. Dis..

[29] Maslove D.M., Shapira T., Tyryshkin K., Veldhoen R.A., Marshall J.C., Muscedere J. (2019). Validation of diagnostic gene sets to identify critically ill patients with sepsis. J. Crit. Care.

[30] Thair S., Mewes C., Hinz J., Bergmann I., Büttner B., Sehmisch S., Meissner K., Quintel M., Sweeney T.E., Khatri P., Mansur A. (2021). Gene expression–based diagnosis of infections in critically ill patients—prospective validation of the SepsisMetaScore in a Longitudinal severe Trauma cohort. Crit. Care Med..

[31] Edgar R., Domrachev M., Lash A.E. (2002). Gene Expression Omnibus: NCBI gene expression and hybridization array data repository. Nucleic Acids Res..

[32] Kolesnikov N., Hastings E., Keays M., Melnichuk O., Tang Y.A., Williams E., Dylag M., Kurbatova N., Brandizi M., Burdett T. (2015). ArrayExpress update-simplifying data submissions. Nucleic Acids Res..

[33] Popper S.J., Gordon A., Liu M., Balmaseda A., Harris E., Relman D.A. (2012). Temporal dynamics of the transcriptional response to dengue virus infection in Nicaraguan children. PLoS Negl. Trop. Dis..

[34] Koh G.C.K.W., Schreiber M.F., Bautista R., Maude R.R., Dunachie S., Limmathurotsakul D., Day N.P.J., Dougan G., Peacock S.J. (2013). Host responses to melioidosis and tuberculosis are both dominated by interferon-mediated signaling. PLoS One.

[35] Ardura M.I., Banchereau R., Mejias A., Di Pucchio T., Glaser C., Allantaz F., Pascual V., Banchereau J., Chaussabel D., Ramilo O. (2009). Enhanced monocyte response and decreased central memory T cells in children with invasive Staphylococcus aureus infections. PLoS One.

[36] Parnell G.P., McLean A.S., Booth D.R., Armstrong N.J., Nalos M., Huang S.J., Manak J., Tang W., Tam O.Y., Chan S., Tang B.M. (2012). A distinct influenza infection signature in the blood transcriptome of patients with severe community-acquired pneumonia. Crit. Care.

[37] Banchereau R., Jordan-Villegas A., Ardura M., Mejias A., Baldwin N., Xu H., Saye E., Rossello-Urgell J., Nguyen P., Blankenship D. (2012). Host immune transcriptional profiles reflect the variability in clinical disease manifestations in patients with staphylococcus aureus infections. PLoS One.

[38] Berdal J.E., Mollnes T.E., Wæhre T., Olstad O.K., Halvorsen B., Ueland T., Laake J.H., Furuseth M.T., Maagaard A., Kjekshus H. (2011). Excessive innate immune response and mutant D222G/N in severe A (H1N1) pandemic influenza. J. Infect..

[39] de Steenhuijsen Piters W.A.A., Heinonen S., Hasrat R., Bunsow E., Smith B., Suarez-Arrabal M.C., Chaussabel D., Cohen D.M., Sanders E.A.M., Ramilo O. (2016). Nasopharyngeal Microbiota, host transcriptome, and disease severity in children with respiratory syncytial virus infection. Am. J. Respir. Crit. Care Med..

[40] Heinonen S., Jartti T., Garcia C., Oliva S., Smitherman C., Anguiano E., de Steenhuijsen Piters W.A.A., Vuorinen T., Ruuskanen O., Dimo B. (2016). Rhinovirus detection in symptomatic and asymptomatic children value of host transcriptome analysis. Am. J. Respir. Crit. Care Med..

[41] Herberg J.A., Kaforou M., Gormley S., Sumner E.R., Patel S., Jones K.D.J., Paulus S., Fink C., Martinon-Torres F., Montana G. (2013). Transcriptomic profiling in childhood H1N1/09 influenza reveals reduced expression of protein synthesis genes. J. Infect. Dis..

[42] Ioannidis I., McNally B., Willette M., Peeples M.E., Chaussabel D., Durbin J.E., Ramilo O., Mejias A., Flaño E. (2012). Plasticity and virus specificity of the airway epithelial cell immune response during respiratory virus infection. J. Virol..

[43] Jong V.L., Ahout I.M.L., van den Ham H.J., Jans J., Zaaraoui-Boutahar F., Zomer A., Simonetti E., Bijl M.A., Brand H.K., van IJcken W.F.J. (2016). Transcriptome assists prognosis of disease severity in respiratory syncytial virus infected infants. Sci. Rep..

[44] Lindow J.C., Wunder E.A., Popper S.J., Min J.N., Mannam P., Srivastava A., Yao Y., Hacker K.P., Raddassi K., Lee P.J. (2016). Cathelicidin Insufficiency in patients with Fatal leptospirosis. PLoS Pathog..

[45] Liu T.Y., Burke T., Park L.P., Woods C.W., Zaas A.K., Ginsburg G.S., Hero A.O. (2016). An individualized predictor of health and disease using paired reference and target samples. BMC Bioinf..

[46] Mejias A., Dimo B., Suarez N.M., Garcia C., Suarez-Arrabal M.C., Jartti T., Blankenship D., Jordan-Villegas A., Ardura M.I., Xu Z. (2013). Whole blood gene expression profiles to assess pathogenesis and disease severity in infants with respiratory syncytial virus infection. PLoS Med..

[47] Rodriguez-Fernandez R., Tapia L.I., Yang C.F., Torres J.P., Chavez-Bueno S., Garcia C., Jaramillo L.M., Moore-Clingenpeel M., Jafri H.S., Peeples M.E. (2017). Respiratory syncytial virus Genotypes, host immune profiles, and disease severity in young children hospitalized with Bronchiolitis. J. Infect. Dis..

[48] Tang B.M., Shojaei M., Parnell G.P., Huang S., Nalos M., Teoh S., O'Connor K., Schibeci S., Phu A.L., Kumar A. (2017). A novel immune biomarker IFI27 discriminates between influenza and bacteria in patients with suspected respiratory infection. Eur. Respir. J..

[49] Ahn S.H., Tsalik E.L., Cyr D.D., Zhang Y., van Velkinburgh J.C., Langley R.J., Glickman S.W., Cairns C.B., Zaas A.K., Rivers E.P. (2013). Gene expression-based classifiers identify Staphylococcus aureus infection in mice and humans. PLoS One.

[50] Bermejo-Martin J.F., Martin-Loeches I., Rello J., Antón A., Almansa R., Xu L., Lopez-Campos G., Pumarola T., Ran L., Ramirez P. (2010). Host adaptive immunity deficiency in severe pandemic influenza. Crit. Care.

[51] Berry M.P.R., Graham C.M., McNab F.W., Xu Z., Bloch S.A.A., Oni T., Wilkinson K.A., Banchereau R., Skinner J., Wilkinson R.J. (2010). An interferon-inducible neutrophil-driven blood transcriptional signature in human tuberculosis. Nature.

[52] Chaussabel D., Quinn C., Shen J., Patel P., Glaser C., Baldwin N., Stichweh D., Blankenship D., Li L., Munagala I. (2008). A modular analysis framework for blood genomics studies: application to systemic lupus erythematosus. Immunity.

[53] Cvijanovich N., Shanley T.P., Lin R., Allen G.L., Thomas N.J., Checchia P., Anas N., Freishtat R.J., Monaco M., Odoms K. (2008). Validating the genomic signature of pediatric septic shock. Physiol. Genomics.

[54] Hu X., Yu J., Crosby S.D., Storch G.A. (2013). Gene expression profiles in febrile children with defined viral and bacterial infection. Proc. Natl. Acad. Sci. USA.

[55] Irwin A.D., Marriage F., Mankhambo L.A., Jeffers G., Kolamunnage-Dona R., Guiver M., Denis B., Molyneux E.M., Molyneux M.E., IPD Group Study (2012). Novel biomarker combination improves the diagnosis of serious bacterial infections in Malawian children. BMC Med. Genomics.

[56] Jaggi P., Mejias A., Xu Z., Yin H., Moore-Clingenpeel M., Smith B., Burns J.C., Tremoulet A.H., Jordan-Villegas A., Chaussabel D. (2018). Whole blood transcriptional profiles as a prognostic tool in complete and incomplete Kawasaki Disease. PLoS One.

[57] Kulohoma B.W., Marriage F., Vasieva O., Mankhambo L., Nguyen K., Molyneux M.E., Molyneux E.M., Day P.J.R., Carrol E.D. (2017). Peripheral blood RNA gene expression in children with pneumococcal meningitis: a prospective case-control study. BMJ Paediatr. Open.

[58] Kwissa M., Nakaya H.I., Onlamoon N., Wrammert J., Villinger F., Perng G.C., Yoksan S., Pattanapanyasat K., Chokephaibulkit K., Ahmed R., Pulendran B. (2014). Dengue virus infection induces expansion of a CD14(+)CD16(+) monocyte population that stimulates plasmablast differentiation. Cell Host Microbe.

[59] Lill M., Kõks S., Soomets U., Schalkwyk L.C., Fernandes C., Lutsar I., Taba P. (2013). Peripheral blood RNA gene expression profiling in patients with bacterial meningitis. Front. Neurosci..

[60] Nascimento E.J.M., Braga-Neto U., Calzavara-Silva C.E., Gomes A.L.V., Abath F.G.C., Brito C.A.A., Cordeiro M.T., Silva A.M., Magalhães C., Andrade R. (2009). Gene expression profiling during early acute febrile stage of dengue infection can predict the disease outcome. PLoS One.

[61] Pankla R., Buddhisa S., Berry M., Blankenship D.M., Bancroft G.J., Banchereau J., Lertmemongkolchai G., Chaussabel D. (2009). Genomic transcriptional profiling identifies a candidate blood biomarker signature for the diagnosis of septicemic melioidosis. Genome Biol..

[62] Popper S.J., Strouts F.R., Lindow J.C., Cheng H.K., Montoya M., Balmaseda A., Durbin A.P., Whitehead S.S., Harris E., Kirkpatrick B.D., Relman D.A. (2018). Early transcriptional responses after dengue Vaccination Mirror the response to natural infection and predict Neutralizing Antibody Titers. J. Infect. Dis..

[63] Simmons C.P., Popper S., Dolocek C., Chau T.N.B., Griffiths M., Dung N.T.P., Long T.H., Hoang D.M., Chau N.V., Thao L.T.T. (2007). Patterns of host genome-wide gene transcript abundance in the peripheral blood of patients with acute dengue hemorrhagic fever. J. Infect. Dis..

[64] Smith C.L., Dickinson P., Forster T., Craigon M., Ross A., Khondoker M.R., France R., Ivens A., Lynn D.J., Orme J. (2014). Identification of a human neonatal immune-metabolic network associated with bacterial infection. Nat. Commun..

[65] Sutherland A., Thomas M., Brandon R.A., Brandon R.B., Lipman J., Tang B., McLean A., Pascoe R., Price G., Nguyen T. (2011). Development and validation of a novel molecular biomarker diagnostic test for the early detection of sepsis. Crit. Care.

[66] Tantibhedhyangkul W., Prachason T., Waywa D., El Filali A., Ghigo E., Thongnoppakhun W., Raoult D., Suputtamongkol Y., Capo C., Limwongse C., Mege J.L. (2011). Orientia tsutsugamushi stimulates an original gene expression program in monocytes: relationship with gene expression in patients with scrub typhus. PLoS Negl. Trop. Dis..

[67] Thuny F., Textoris J., Amara A.B., Filali A.E., Capo C., Habib G., Raoult D., Mege J.L. (2012). The gene expression analysis of blood reveals S100A11 and AQP9 as potential biomarkers of infective endocarditis. PLoS One.

[68] van de Weg C.A.M., van den Ham H.J., Bijl M.A., Anfasa F., Zaaraoui-Boutahar F., Dewi B.E., Nainggolan L., van IJcken W.F.J., Osterhaus A.D.M.E., Martina B.E.E. (2015). Time since onset of disease and individual clinical markers associate with transcriptional changes in uncomplicated dengue. PLoS Negl. Trop. Dis..

[69] Wong H.R., Shanley T.P., Sakthivel B., Cvijanovich N., Lin R., Allen G.L., Thomas N.J., Doctor A., Kalyanaraman M., Tofil N.M. (2007). Genome-level expression profiles in pediatric septic shock indicate a role for altered zinc homeostasis in poor outcome. Physiol. Genomics.

[70] Zhai Y., Franco L.M., Atmar R.L., Quarles J.M., Arden N., Bucasas K.L., Wells J.M., Niño D., Wang X., Zapata G.E. (2015). Host transcriptional response to influenza and other acute respiratory viral infections – a prospective cohort study. PLoS Pathog..

[71] Mahajan P., Kuppermann N., Mejias A., Suarez N., Chaussabel D., Casper T.C., Smith B., Alpern E.R., Anders J., Atabaki S.M. (2016). Association of RNA Biosignatures with bacterial infections in febrile infants aged 60 Days or younger. JAMA.

[72] Parnell G., McLean A., Booth D., Huang S., Nalos M., Tang B. (2011). Aberrant cell cycle and apoptotic changes characterise severe influenza a infection - a meta-analysis of genomic signatures in circulating leukocytes. PLoS One.

[73] Ramilo O., Allman W., Chung W., Mejias A., Ardura M., Glaser C., Wittkowski K.M., Piqueras B., Banchereau J., Palucka A.K., Chaussabel D. (2007). Gene expression patterns in blood leukocytes discriminate patients with acute infections. Blood.

[74] Sweeney T.E., Wong H.R., Khatri P. (2016). Robust classification of bacterial and viral infections via integrated host gene expression diagnostics. Sci. Transl. Med..

[75] Sampson D.L., Fox B.A., Yager T.D., Bhide S., Cermelli S., McHugh L.C., Seldon T.A., Brandon R.A., Sullivan E., Zimmerman J.J. (2017). A four-biomarker blood signature discriminates systemic inflammation due to viral infection versus other Etiologies. Sci. Rep..

[76] Tsalik E.L., Henao R., Nichols M., Burke T., Ko E.R., McClain M.T., Hudson L.L., Mazur A., Freeman D.H., Veldman T. (2016). Host gene expression classifiers diagnose acute respiratory illness etiology. Sci. Transl. Med..

[77] Warsinske H., Vashisht R., Khatri P. (2019). Host-response-based gene signatures for tuberculosis diagnosis: a systematic comparison of 16 signatures. PLoS Med..

[78] Warsinske H.C., Rao A.M., Moreira F.M.F., Santos P.C.P., Liu A.B., Scott M., Malherbe S.T., Ronacher K., Walzl G., Winter J. (2018). Assessment of Validity of a blood-based 3-gene signature score for progression and diagnosis of tuberculosis, disease severity, and treatment response. JAMA Netw. Open.

[79] Philipp Doebler H.H., Holling H., Sousa-Pinto B. (2013).

[80] Södersten E., Ongarello S., Mantsoki A., Wyss R., Persing D.H., Banderby S. (2020). Diagnostic accuracy study of a novel blood-based assay for identification of tuberculosis in people living with HIV. J. Clin. Microbiol..

[81] Zimmer A.J., Schumacher S.G., Södersten E., Mantsoki A., Wyss R., Persing D.H., Banderby S., Strömqvist Meuzelaar L., Prieto J., Gnanashanmugam D. (2021). A novel blood-based assay for treatment monitoring of tuberculosis. BMC Res. Notes.

[82] Moreira F.M.F., Verma R., Pereira Dos Santos P.C., Leite A., da Silva Santos A., de Araujo R.C.P., da Silva B.O., de Sá Queiroz J.H.F., Persing D.H., Södersten E. (2021). Blood-based host biomarker diagnostics in active case finding for pulmonary tuberculosis: a diagnostic case-control study. EClinicalMedicine.

[83] Sutherland J.S., Spuy G van der, Gindeh A., Thuong N.T., Namuganga A.R., Owolabi O. (2021). Diagnostic accuracy of the Cepheid 3-gene host response fingerstick blood test in a prospective, multi-site study: interim results. Clin. Infect. Dis..

[84] Bongen E., Lucian H., Khatri A., Fragiadakis G.K., Bjornson Z.B., Nolan G.P., Utz P.J., Khatri P. (2019). Sex differences in the blood transcriptome identify robust changes in immune cell proportions with aging and influenza infection. Cell Rep..

[85] Tukiainen T., Villani A.C., Yen A., Rivas M.A., Marshall J.L., Satija R., Aguirre M., Gauthier L., Fleharty M., Kirby A. (2017). Landscape of X chromosome inactivation across human tissues. Nature.

[86] GBD 2019 Diseases and Injuries Collaborators (2020). Global burden of 369 diseases and injuries in 204 countries and territories, 1990–2019: a systematic analysis for the Global Burden of Disease Study 2019. Lancet.

[87] Plosa E.J., Esbenshade J.C., Fuller M.P., Weitkamp J.H. (2012). Cytomegalovirus infection. Pediatr. Rev..

[88] Zhou W., Lin F., Teng L., Li H., Hou J., Tong R., Zheng C., Lou Y., Tan W. (2013). Prevalence of herpes and respiratory viruses in induced sputum among hospitalized children with non typical bacterial community-acquired pneumonia. PLoS One.

[89] Wacker C., Prkno A., Brunkhorst F.M., Schlattmann P. (2013). Procalcitonin as a diagnostic marker for sepsis: a systematic review and meta-analysis. Lancet Infect. Dis..

[90] Ducharme J., Self W.H., Osborn T.M., Ledeboer N.A., Romanowsky J., Sweeney T.E., Liesenfeld O., Rothman R.E. (2020). A multi-mRNA host-response molecular blood test for the diagnosis and prognosis of acute infections and sepsis: Proceedings from a clinical advisory Panel. J. Pers. Med..

[91] Suzuki K., Ohkuma M., Nagaoka I. (2019). Bacterial lipopolysaccharide and antimicrobial LL-37 enhance ICAM-1 expression and NF-kappaB p65 phosphorylation in senescent endothelial cells. Int. J. Mol. Med..

[92] Sambarey A., Devaprasad A., Mohan A., Ahmed A., Nayak S., Swaminathan S., D'Souza G., Jesuraj A., Dhar C., Babu S. (2017). Unbiased identification of blood-based biomarkers for pulmonary tuberculosis by modeling and mining molecular Interaction networks. EBioMedicine.

[93] Zheng R., Liu H., Song P., Feng Y., Qin L., Huang X., Chen J., Yang H., Liu Z., Cui Z. (2015). Epstein-Barr virus-induced gene 3 (EBI3) polymorphisms and expression are associated with susceptibility to pulmonary tuberculosis. Tuberculosis.

[94] Tolfvenstam T., Lindblom A., Schreiber M.J., Ling L., Chow A., Ooi E.E., Hibberd M.L. (2011). Characterization of early host responses in adults with dengue disease. BMC Infect. Dis..

[95] Li L., Ni Y.A., Song Z., Yi Z., Wang F. (2021). Identification of pathogenic genes and transcription factors in respiratory syncytial virus. BMC Pediatr..

[96] Turner C.T. (2020). Blood transcriptional profiling in a prospective observational cohort of South African adults presenting for investigation of possible pulmonary tuberculosis. BioStudies.

[97] Eckold C., Kumar V., Weiner J., Alisjahbana B., Riza A.L., Ronacher K., Coronel J., Kerry-Barnard S., Malherbe S.T., Kleynhans L. (2021). Impact of Intermediate Hyperglycemia and Diabetes on immune Dysfunction in tuberculosis. Clin. Infect. Dis..

[98] Singhania A., Verma R., Graham C.M., Lee J., Tran T., Richardson M., Lecine P., Leissner P., Berry M.P.R., Wilkinson R.J. (2018). A modular transcriptional signature identifies phenotypic heterogeneity of human tuberculosis infection. Nat. Commun..

[99] Pollard A., Blohmke C. (2015). Whole blood transcriptome in humans experimentally infected with Salmonella Typhi. BioStudies.

[100] Schreiber F. (2010). Transcription profiling of diabetic vs non-diabetic human blood cells infected with Burkholderia pseudomallei. BioStudies.

[101] Sweeney T.E., Shidham A., Wong H.R., Khatri P. (2015). A comprehensive time-course-based multicohort analysis of sepsis and sterile inflammation reveals a robust diagnostic gene set. Sci. Transl. Med..

[102] van den Ham H.J. (2015). Time since onset of disease and individual clinical markers associate with transcriptional changes in uncomplicated dengue. BioStudies.

[103] Andeweg A. (2016). Blood mRNA profiling to support prognosis of disease severity in respiratory syncytial virus infected infants. BioStudies.

[104] Liu K., Chen L., Kaur R., Pichichero M. (2012). Transcriptome signature in young children with acute otitis media due to Streptococcus pneumoniae. Microbes Infect..

[105] Bloom C.I., Graham C.M., Berry M.P.R., Rozakeas F., Redford P.S., Wang Y., Xu Z., Wilkinson K.A., Wilkinson R.J., Kendrick Y. (2013). Transcriptional blood signatures distinguish pulmonary tuberculosis, pulmonary sarcoidosis, pneumonias and lung cancers. PLoS One.

[106] Maertzdorf J., Ota M., Repsilber D., Mollenkopf H.J., Weiner J., Hill P.C., Kaufmann S.H.E. (2011). Functional correlations of pathogenesis-driven gene expression signatures in tuberculosis. PLoS One.

[107] Maertzdorf J., Weiner J., Mollenkopf H.J., Bauer T., Prasse A., Müller-Quernheim J., Kaufmann S.H.E., TBornotTB Network (2012). Common patterns and disease-related signatures in tuberculosis and sarcoidosis. Proc. Natl. Acad. Sci. USA.

[108] Verhagen L.M., Zomer A., Maes M., Villalba J.A., Del Nogal B., Eleveld M., van Hijum S.A., de Waard J.H., Hermans P.W. (2013). A predictive signature gene set for discriminating active from latent tuberculosis in Warao Amerindian children. BMC Genom..

[109] Blankley S., Graham C.M., Turner J., Berry M.P.R., Bloom C.I., Xu Z., Pascual V., Banchereau J., Chaussabel D., Breen R. (2016). The transcriptional signature of active tuberculosis reflects symptom Status in extra-pulmonary and pulmonary tuberculosis. PLoS One.

[110] Maertzdorf J., Repsilber D., Walzl G., Mollenkopf H., Kaufmann S. (2010). Tuberculosis patients, latent and uninfected controls; whole blood. BioStudies.

[111] Noursadeghi M. (2016). Whole blood transcriptional profiling of healthy volunteers and patients with active tuberculosis. BioStudies.

[112] Rinchai D., Altman M.C., Konza O., Hässler S., Martina F., Toufiq M., Garand M., Kabeer B.S.A., Palucka K., Mejias A. (2020). Definition of erythroid cell-positive blood transcriptome phenotypes associated with severe respiratory syncytial virus infection. Clin. Transl. Med..

[113] Park J., Munagala I., Xu H., Blankenship D., Maffucci P., Chaussabel D., Banchereau J., Pascual V., Cunningham-Rundles C. (2013). Interferon signature in the blood in inflammatory common variable immune deficiency. PLoS One.

[114] Montano M., Rarick M., Sebastiani P., Brinkmann P., Russell M., Navis A., Wester C., Thior I., Essex M. (2006). Gene-expression profiling of HIV-1 infection and perinatal transmission in Botswana. Genes Immun..

[115] Singh S., Toor J.S., Sharma A., Arora S.K. (2020). Signature genes associated with immunological non-responsiveness to anti-retroviral therapy in HIV-1 subtype-c infection. PLoS One.

[116] R Core Team (2020).

[117] Wu J., Irizarry R. (2020).

[118] Love M.I., Huber W., Anders S. (2014). Moderated estimation of fold change and dispersion for RNA-seq data with DESeq2. Genome Biol..

[119] Haynes W.A., Vallania F., Liu C., Bongen E., Tomczak A., Andres-Terrè M., Lofgren S., Tam A., Deisseroth C.A., Li M.D. (2017). Empowering multi-cohort gene expression analysis to increase reproducibility. Pac. Symp. Biocomput..

[120] Sing T., Sander O., Beerenwinkel N., Lengauer T. (2005). ROCR: visualizing classifier performance in R. Bioinformatics.

[121] Lumley T. (2003).

[122] López-Ratón M., Rodríguez-Álvarez M.X., Suárez C.C., Sampedro F.G. (2014). OptimalCutpoints: an R package for selecting optimal Cutpoints in diagnostic tests. J. Stat. Software.

[123] Friedman J., Hastie T., Tibshirani R. (2010). Regularization Paths for generalized linear models via Coordinate descent. J. Stat. Softw..

[124] Tusher V.G., Tibshirani R., Chu G. (2001). Significance analysis of microarrays applied to the ionizing radiation response. Proc. Natl. Acad. Sci. USA.

[125] Phetsouvanh R., Phongmany S., Soukaloun D., Rasachak B., Soukhaseum V., Soukhaseum S., Frichithavong K., Khounnorath S., Pengdee B., Phiasakha K. (2006). Causes of community-acquired bacteremia and patterns of antimicrobial resistance in Vientiane, Laos. Am. J. Trop. Med. Hyg..

[126] Nielsen A.C.Y., Böttiger B., Midgley S.E., Nielsen L.P. (2013). A novel enterovirus and parechovirus multiplex one-step real-time PCR-validation and clinical experience. J. Virol. Methods.

[127] Jia J., Ma Y., Zhao X., Guo Y., Huangfu C., Fang C., Fan R., Lv M., Yin H., Zhang J. (2015). Prevalence of human parvovirus B19 in Chinese plasma pools for manufacturing plasma derivatives. Virol. J..

[128] Binnicker M.J., Espy M.E. (2013). Comparison of six real-time PCR assays for qualitative detection of cytomegalovirus in clinical specimens. J. Clin. Microbiol..

[129] Riediger I.N., Stoddard R.A., Ribeiro G.S., Nakatani S.M., Moreira S.D.R., Skraba I., Biondo A.W., Reis M.G., Hoffmaster A.R., Vinetz J.M. (2017). Rapid, actionable diagnosis of urban epidemic leptospirosis using a pathogenic Leptospira lipL32-based real-time PCR assay. PLoS Negl. Trop. Dis..

[130] Henry K.M., Jiang J., Rozmajzl P.J., Azad A.F., Macaluso K.R., Richards A.L. (2007). Development of quantitative real-time PCR assays to detect Rickettsia typhi and Rickettsia felis, the causative agents of murine typhus and flea-borne spotted fever. Mol. Cell. Probes.

[131] Jiang J., Chan T.C., Temenak J.J., Dasch G.A., Ching W.M., Richards A.L. (2004). Development of a quantitative real-time polymerase chain reaction assay specific for Orientia tsutsugamushi. Am. J. Trop. Med. Hyg..

[132] Locatelli G., Santoro F., Veglia F., Gobbi A., Lusso P., Malnati M.S. (2000). Real-time quantitative PCR for human herpesvirus 6 DNA. J. Clin. Microbiol..

[133] Johnson W.E., Fau L.C., Rabinovic A. (2006). Adjusting batch effects in microarray expression data using empirical Bayes methods. Biostat. TA - Biostat.

[134] Bodkin N., Ross M., McClain M.T., Ko E.R., Woods C.W., Ginsburg G.S., Henao R., Tsalik E.L. (2022). Systematic comparison of published host gene expression signatures for bacterial/viral discrimination. Genome Med..

[135] Mayhew M.B., Buturovic L., Luethy R., Midic U., Moore A.R., Roque J.A., Shaller B.D., Asuni T., Rawling D., Remmel M. (2020). A generalizable 29-mRNA neural-network classifier for acute bacterial and viral infections. Nat. Commun..

[136] Kaforou M., Wright V.J., Oni T., French N., Anderson S.T., Bangani N., Banwell C.M., Brent A.J., Crampin A.C., Dockrell H.M. (2013). Detection of tuberculosis in HIV-infected and -uninfected African adults using whole blood RNA expression signatures: a case-control study. PLoS Med..

[137] Tibshirani R. (1996). Regression shrinkage and selection via the Lasso. J. Roy. Stat. Soc. B.

[138] Benjamini Y., Hochberg Y. (1995). Controlling the false discovery rate: a practical and powerful approach to multiple testing. J. Roy. Stat. Soc. B.

[139] Doncaster C.P., Spake R. (2018). Correction for bias in meta-analysis of little-replicated studies. Methods Ecol. Evol..

[140] Hedges L.V. (1982). Estimation of effect size from a series of independent experiments. Psychol. Bull..

[141] Hedges L.V. (1983). A random effects model for effect sizes. Psychol. Bull..

[142] Arlot S., Celisse A. (2010). A survey of cross-validation procedures for model selection. Stat. Surv..

[143] Stone M. (1974). Cross-validatory Choice and assessment of statistical Predictions. J. Roy. Stat. Soc. B.

[144] Kester A.D., Buntinx F. (2000). Meta-analysis of ROC curves. Med. Decis. Making.

[145] Shapiro D.E. (1999). The interpretation of diagnostic tests. Stat. Methods Med. Res..

[146] Youden W.J. (1950). Index for rating diagnostic tests. Cancer.

[147] Edwards D. (2003). Non-linear normalization and background correction in one-channel cDNA microarray studies. Bioinformatics.

