# Supplementary material for: A robust host-response-based signature distinguishes bacterial and viral infections across diverse global populations
Source: Cell Rep Med. 2022 Dec 20;3(12):100842. doi: 10.1016/j.xcrm.2022.100842 (PMC9797950; doi:10.1016/j.xcrm.2022.100842)
Supplement: Document S1. Figures S1–S11 and Tables S1–S12 [file mmc1.pdf]

Cell Reports Medicine, Volume *3*

Supplemental information

A robust host-response-based signature distinguishes bacterial and viral infections across diverse global populations

Aditya M. Rao, Stephen J. Popper, Sanjana Gupta, Viengmon Davong, Krista Vaidya, Anisone Chanthongthip, Sabine Dittrich, Matthew T. Robinson, Manivanh Vongsouvath, Mayfong Mayxay, Pruksa Nawtaisong, Biraj Karmacharya, Simone A. Thair, Isaac Bogoch, Timothy E. Sweeney, Paul N. Newton, Jason R. Andrews, David A. Relman, and Purvesh Khatri

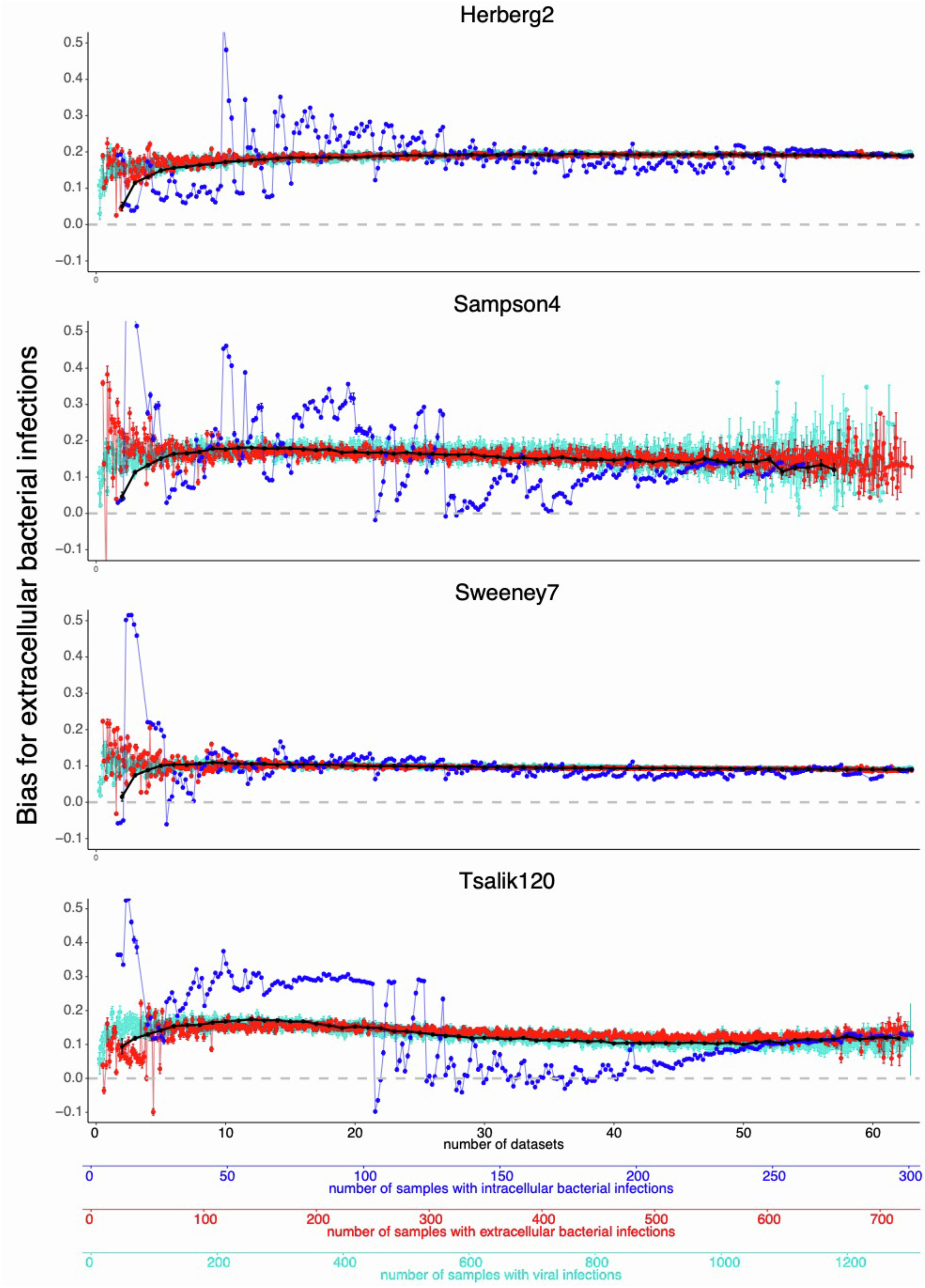

Herberg2
Sampson4
Sweeney7
Tsalik120
Bias for extracellular bacterial infections
0.5
0.4
0.3
0.2
0.1
0.0
−0.1
0
10
20
30
40
50
60
number of datasets
0
50
100
150
200
250
300
number of samples with intracellular bacterial infections
0
100
200
300
400
500
600
700
number of samples with extracellular bacterial infections
0
200
400
600
800
1000
1200
number of samples with viral infections

Figure S1. **Lower accuracy in intracellular bacterial infections is not dependent on the number of datasets or samples used.** Summary of the difference in performance between extracellular and intracellular bacterial infections for 1,396,677 unique, randomly sampled subsets of our 64 datasets. Bias for extracellular bacterial infections was calculated by taking the extracellular bacterial vs. viral infection AUROC and subtracting the intracellular bacterial vs. viral infection AUROC. The dotted line at 0 indicates no bias, and anything above the dotted line indicates positive bias (i.e., higher performance for extracellular vs. viral infection). The bias was averaged for each unique number of datasets (black) or samples in each of the three categories (intracellular bacterial infection = blue; extracellular bacterial infections = red; viral infections = cyan). The x-axes were scaled to match each other and then curves were overlaid. The error bar indicates the standard error of each measurement. **Related to Figure 1**.

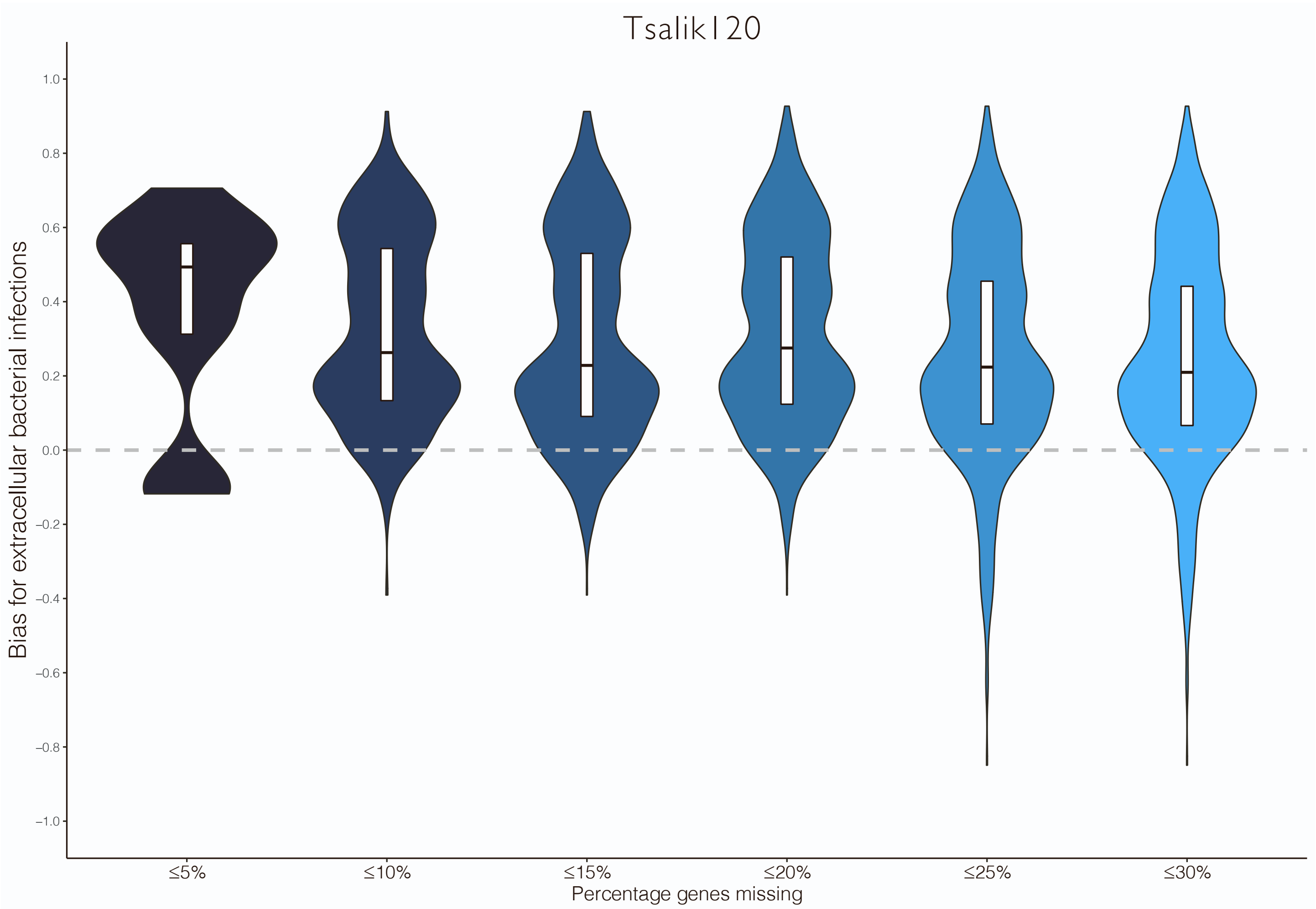

Figure S2. **The AUROC differential in the Tsalik120 signature is less biased when fewer genes are missing.** Summary of the difference in performance between extracellular and intracellular bacterial infections for 1,396,677 unique, randomly sampled subsets of our 64 datasets. The difference in performance was averaged for each unique number of missing genes in the Tsalik120 signature. The violin plots represent the distribution of AUROCs for all combinations where at most the given percentage of genes are missing. The horizontal grey dotted line at 0 indicates no difference between extracellular vs. viral and intracellular vs. viral performance, and anything above the dotted line indicates higher performance for extracellular vs. viral performance. **Related to Figure 1**.

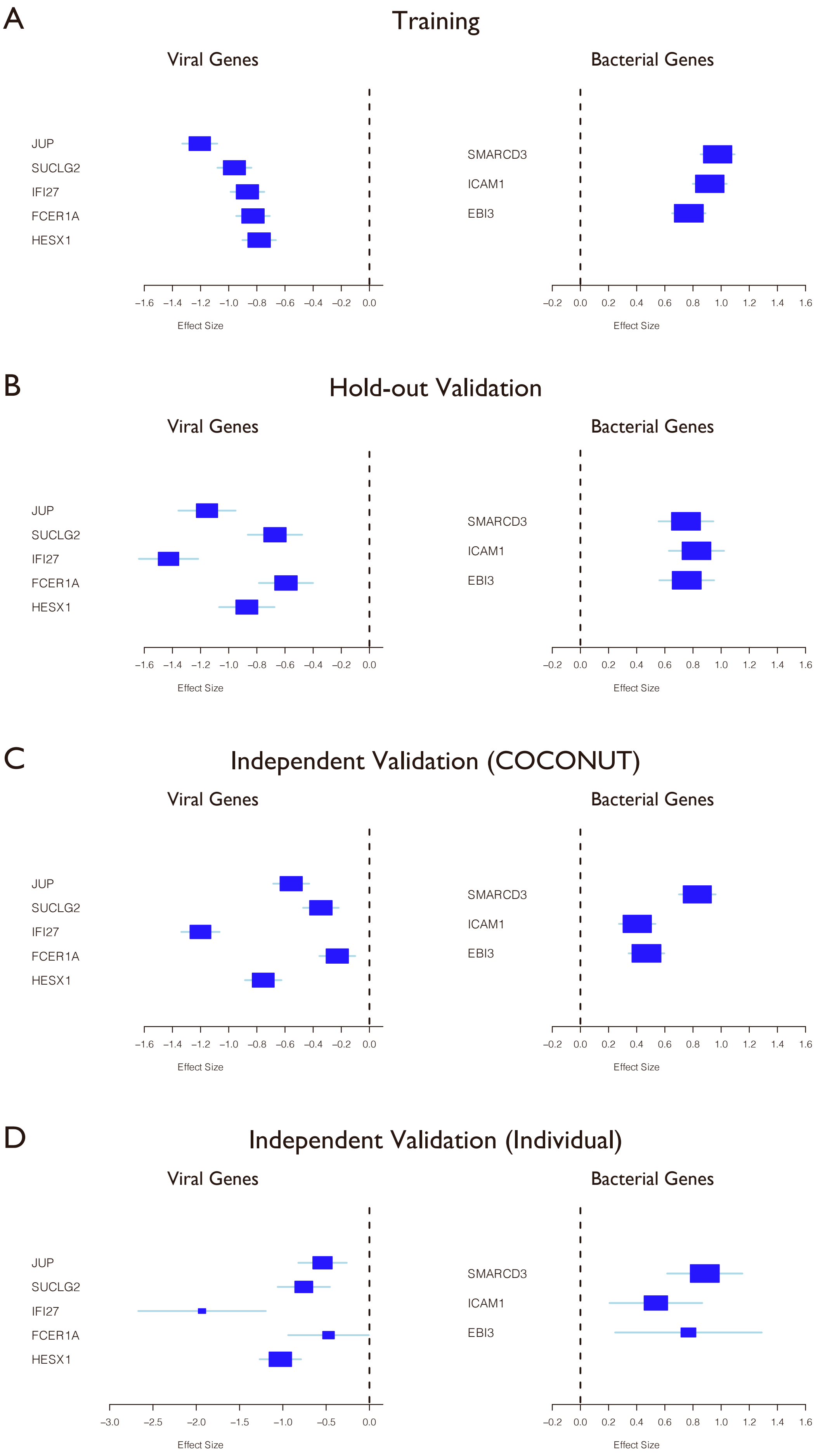
A
Training
Viral Genes
JUP
SUCLG2
IFI27
FCER1A
HESX1
−1.6 −1.4 −1.2 −1.0 −0.8 −0.6 −0.4 −0.2 0.0
Effect Size
Bacterial Genes
SMARCD3
ICAM1
EBI3
−0.2 0.0 0.2 0.4 0.6 0.8 1.0 1.2 1.4 1.6
Effect Size
B
Hold-out Validation
Viral Genes
JUP
SUCLG2
IFI27
FCER1A
HESX1
−1.6 −1.4 −1.2 −1.0 −0.8 −0.6 −0.4 −0.2 0.0
Effect Size
Bacterial Genes
SMARCD3
ICAM1
EBI3
−0.2 0.0 0.2 0.4 0.6 0.8 1.0 1.2 1.4 1.6
Effect Size
C
Independent Validation (COCONUT)
Viral Genes
JUP
SUCLG2
IFI27
FCER1A
HESX1
−1.6 −1.4 −1.2 −1.0 −0.8 −0.6 −0.4 −0.2 0.0
Effect Size
Bacterial Genes
SMARCD3
ICAM1
EBI3
−0.2 0.0 0.2 0.4 0.6 0.8 1.0 1.2 1.4 1.6
Effect Size
D
Independent Validation (Individual)
Viral Genes
JUP
SUCLG2
IFI27
FCER1A
HESX1
−3.0 −2.5 −2.0 −1.5 −1.0 −0.5 0.0
Effect Size
Bacterial Genes
SMARCD3
ICAM1
EBI3
−0.2 0.0 0.2 0.4 0.6 0.8 1.0 1.2 1.4 1.6
Effect Size

Figure S3. **The 8 signature genes exhibit consistent differential expression patterns across all analysis stages.** Forest plots of the 8 signature genes in the BoVI signature, with bacterial samples as the positive class and viral samples as the negative class. These forest plots display the effect sizes for each signature gene, computed as Hedges' *g*. The size of the blue rectangles is proportional to the standard error of the effect size. Whiskers represent the 95% confidence interval. **(A-C)** Effect sizes were computed across all samples. **(D)** Summary effect sizes were computed for each gene by performing a meta-analysis across all 5 datasets. Meta-analysis was done using the R package *MetaIntegrator*. **Related to Figure 2**.

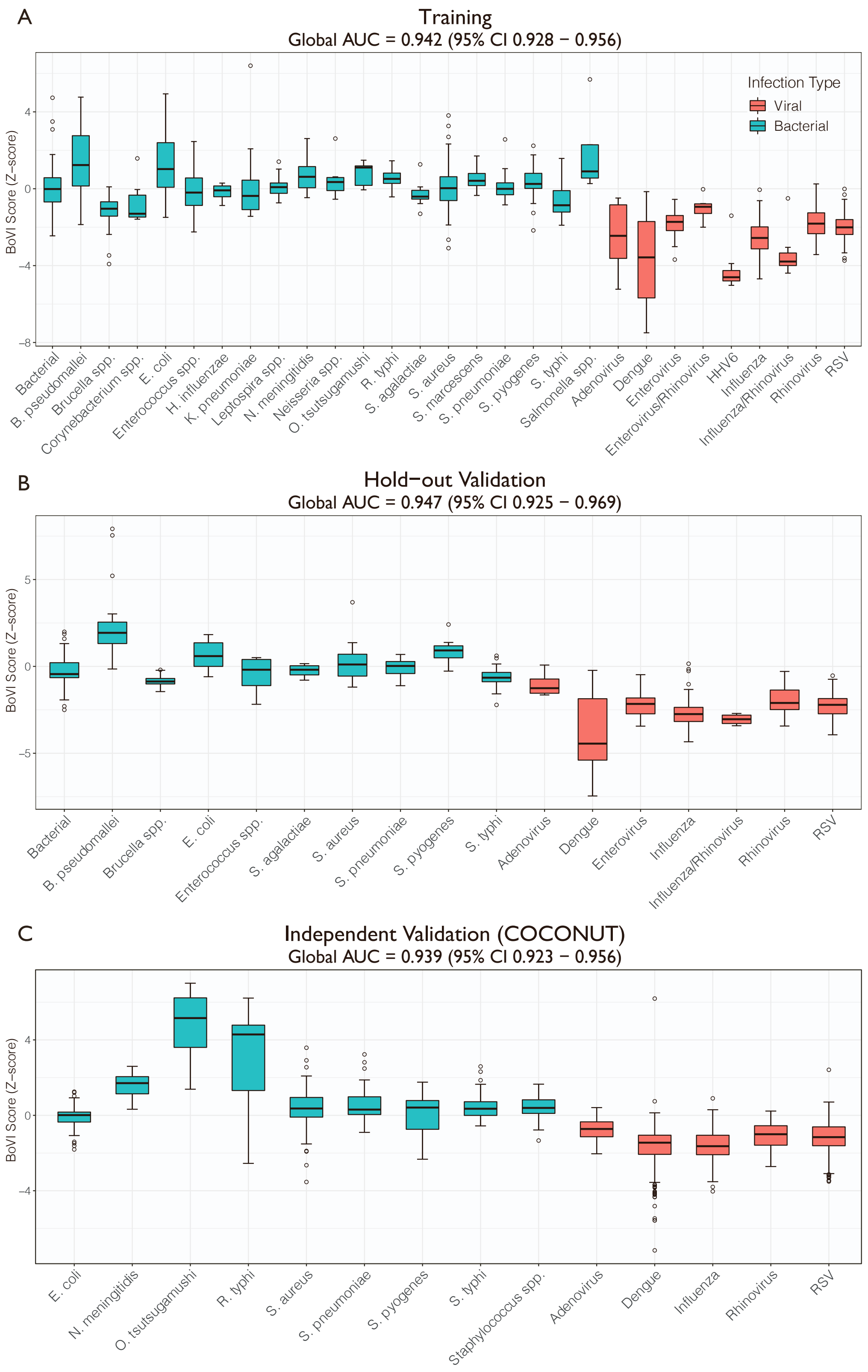

Figure S4. The BoVI signature works across a wide variety of pathogens. Boxplots of the BoVI score across different pathogens, for each in-silico analysis stage. Scores were centered at 0 and then the Z-score was calculated. Any bacterial infection with a non-specific diagnosis was listed as "Bacterial". Only infecting pathogens that were present in at least 4 samples were displayed. **Related to Figure 3**.

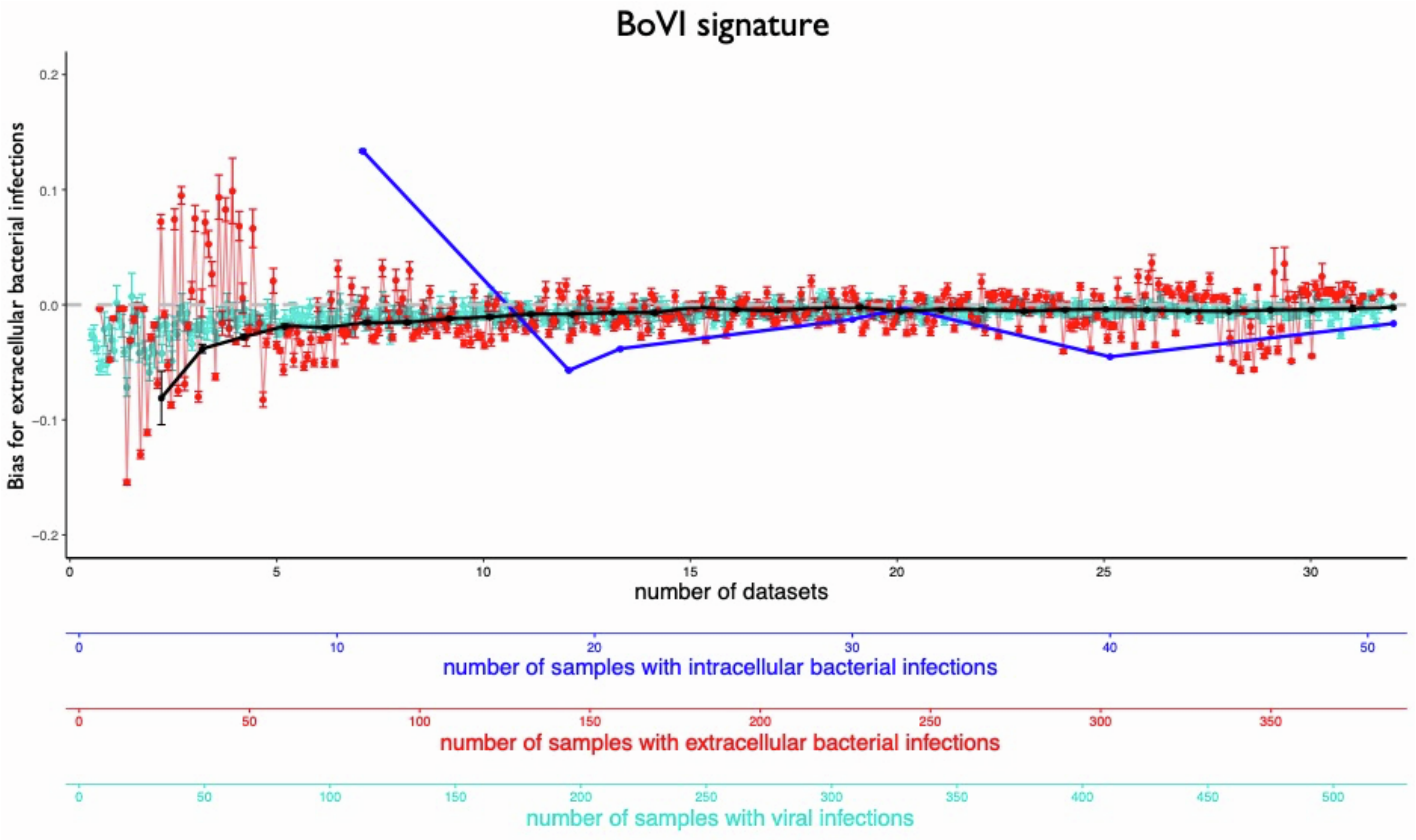

Figure S5. **The BoVI score is not biased toward extracellular bacterial infections, regardless of the number of datasets or samples used.** Summary of the difference in performance between extracellular and intracellular bacterial infections for 435,902 unique, randomly sampled subsets of our 32 independent validation datasets. Bias for extracellular bacterial infections was calculated by taking the extracellular bacterial vs. viral infection AUROC and subtracting the intracellular bacterial vs. viral infection AUROC. Bias was averaged for each unique number of datasets (black) or samples in each of the three categories (intracellular bacterial infection = blue; extracellular bacterial infections = red; viral infections = cyan). The dotted line at 0 indicates no bias, and anything above the dotted line indicates positive bias (i.e., higher performance for extracellular vs. viral infection). The x-axes were scaled to match each other and then curves were overlaid. The error bar indicates the standard error of each measurement. **Related to Figure 3**.

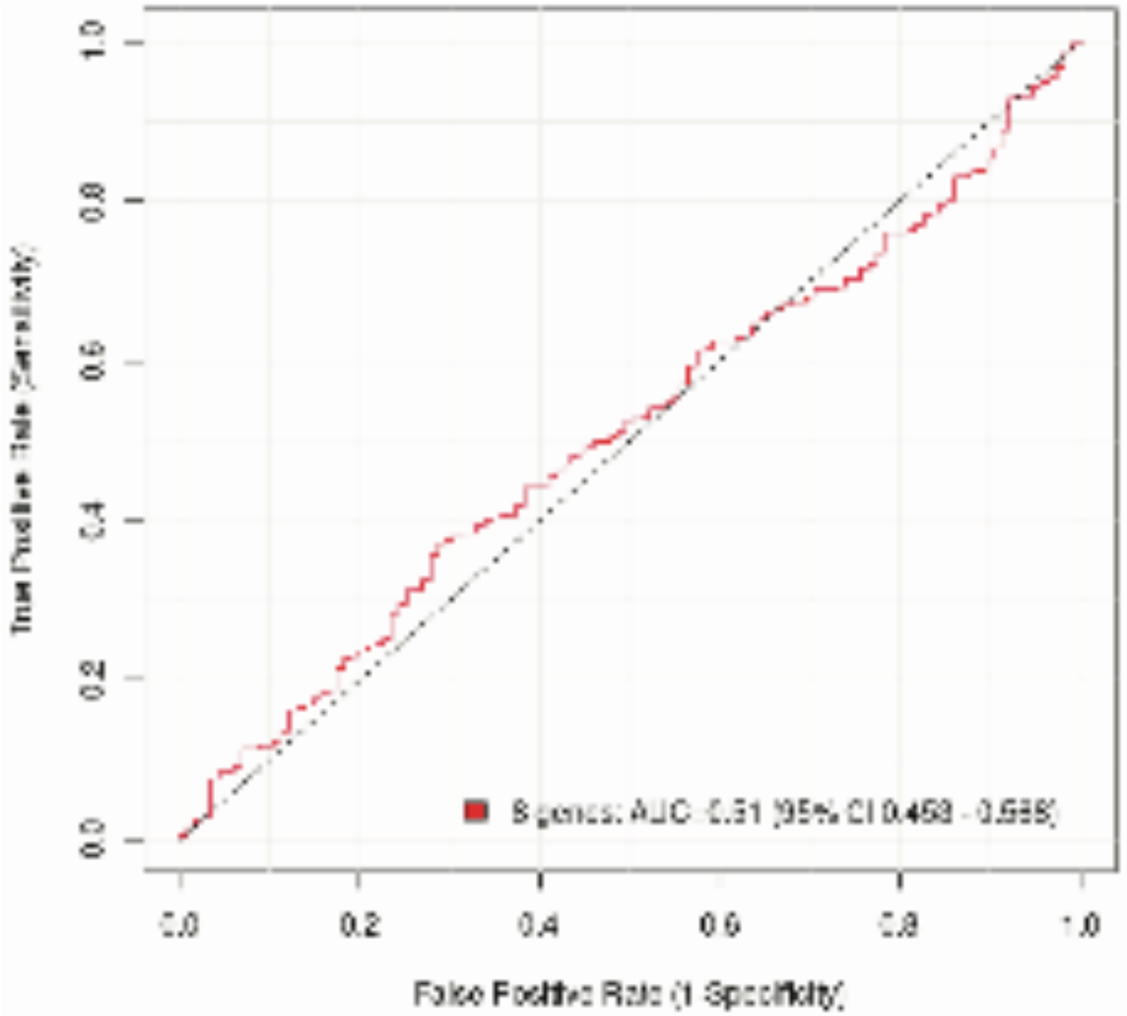

Figure S6. The 8-gene signature does not distinguish healthy controls from studies that profiled either patients with TB or with HIV, demonstrating it is not affected by batch effects between these studies. Related to Figure 3.

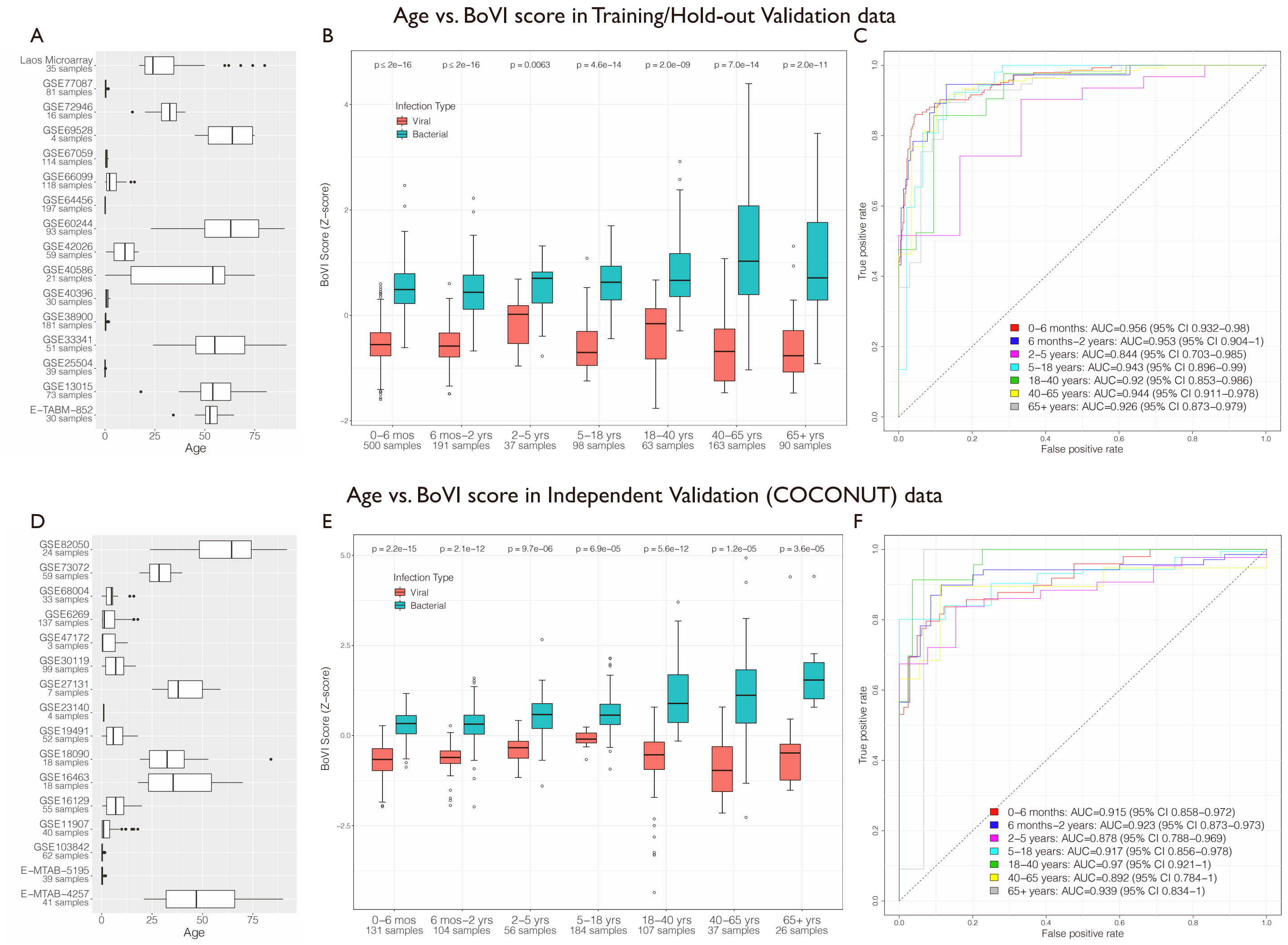

Figure S7. **The BoVI signature maintains high accuracy across all ages.** Performance of the BoVI signature was calculated for different age windows across training, hold-out validation, and independent validation samples. Samples were used only if (i) the exact age was listed, (ii) a range of ages was provided that fit completely into a single age window, or (iii) the mean and standard deviation of the ages was provided and all ages that were within one standard deviation of the mean fit into a single age window. In training/hold-out validation, 1,035 samples had the exact age listed and 107 samples had a usable age range. In independent validation, 600 samples had the exact age listed and 45 samples had a usable age range. Overall, there were 1,142 samples in training/hold-out validation and 645 samples in independent validation that were used. **(A,D)** All samples with usable age information were plotted to show the distribution of ages within each dataset. Ages were randomly generated for the 107 samples in training/hold-out validation and 45 samples in independent validation which only had age range info available. For samples with an exact range provided, ages were randomly sampled from a uniform distribution. For samples with the mean and standard deviation provided, ages were randomly sampled from a normal distribution. **(B,E)** Scores were centered at 0 and then the Z-score was calculated. P-values were calculated using the Mann-Whitney U test. **(C,F)** ROC curves comparing the performance of the BoVI signature across each age window. mos, months; yrs, years. **Related to Figure 4**.

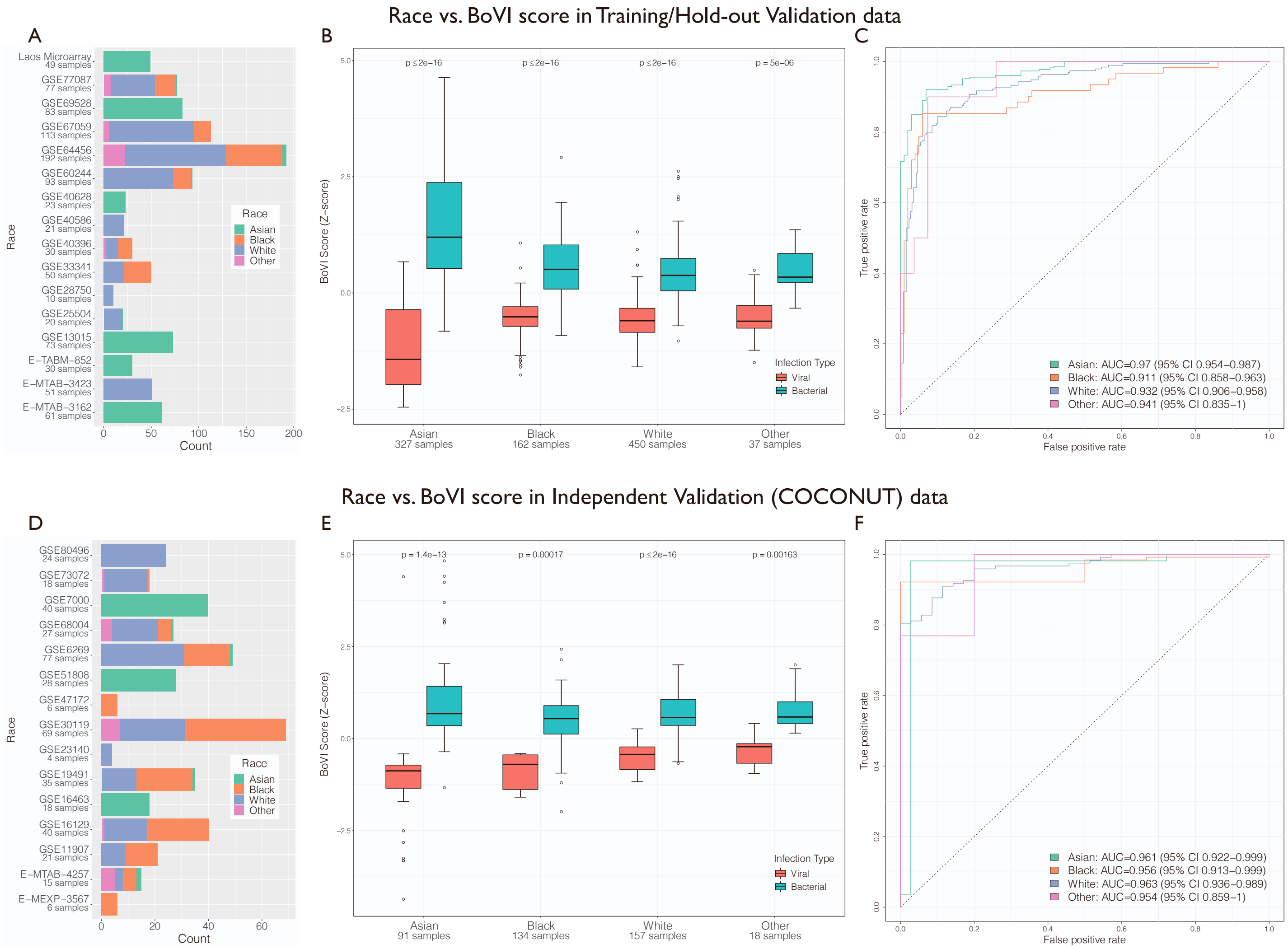

Figure S8. **The BoVI signature retains high accuracy regardless of race.** Performance of the BoVI signature was calculated for each race category across training, hold-out validation, and independent validation samples. Overall, there were 976 samples in training/hold-out validation and 400 samples in independent validation that were used. **(A,D)** All samples with information on race were plotted to show the distribution within each dataset. **(B,E)** Scores were centered at 0 and then the Z-score was calculated. P-values were calculated using the Mann-Whitney U test. **(C,F)** ROC curves comparing the performance of the BoVI signature across each race category. **Related to Figure 4**.

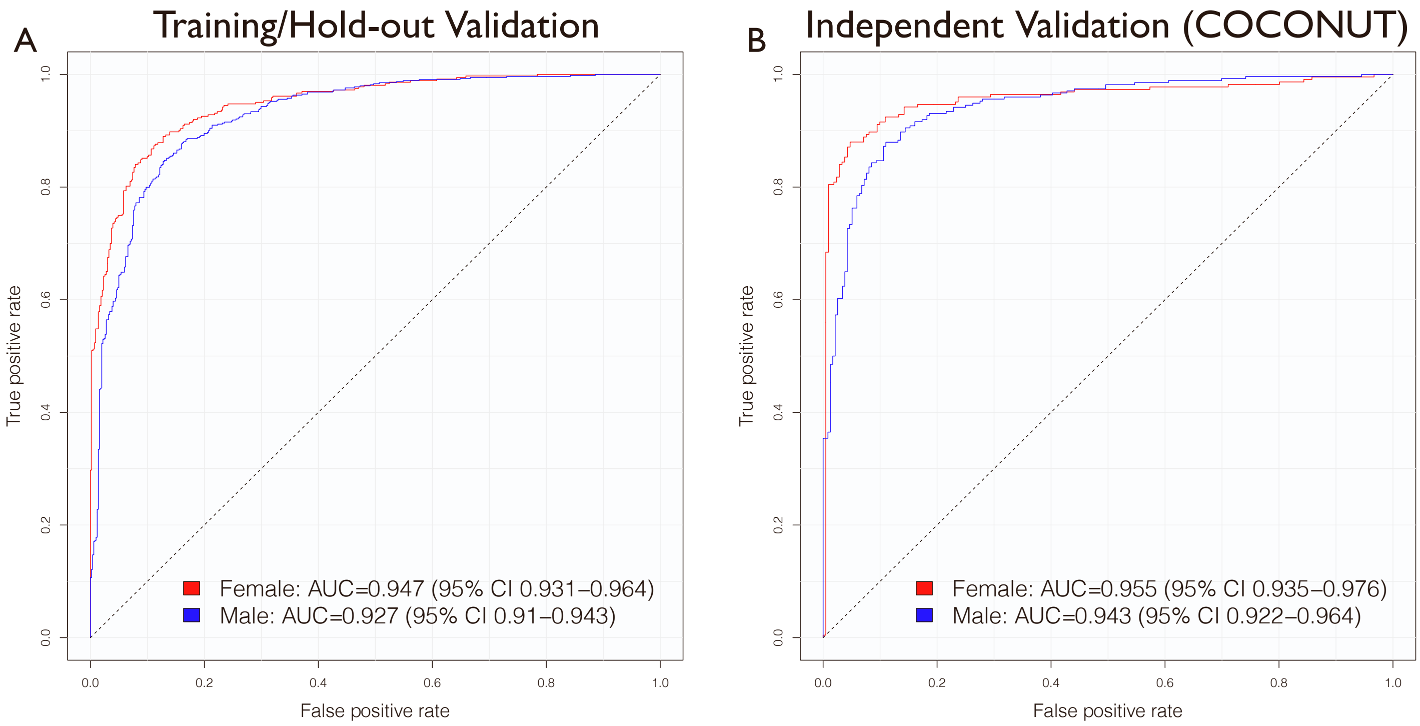

Figure S9. **The BoVI signature's performance is unaffected by sex.** ROC curves comparing the performance of the BoVI signature for all male or all female samples. Overall, there were 1,839 samples in training/hold-out validation and 923 samples in independent validation that were used. **(A)** Performance across training and hold-out validation samples. **(B)** Performance across independent validation samples. **Related to Figure 4**.

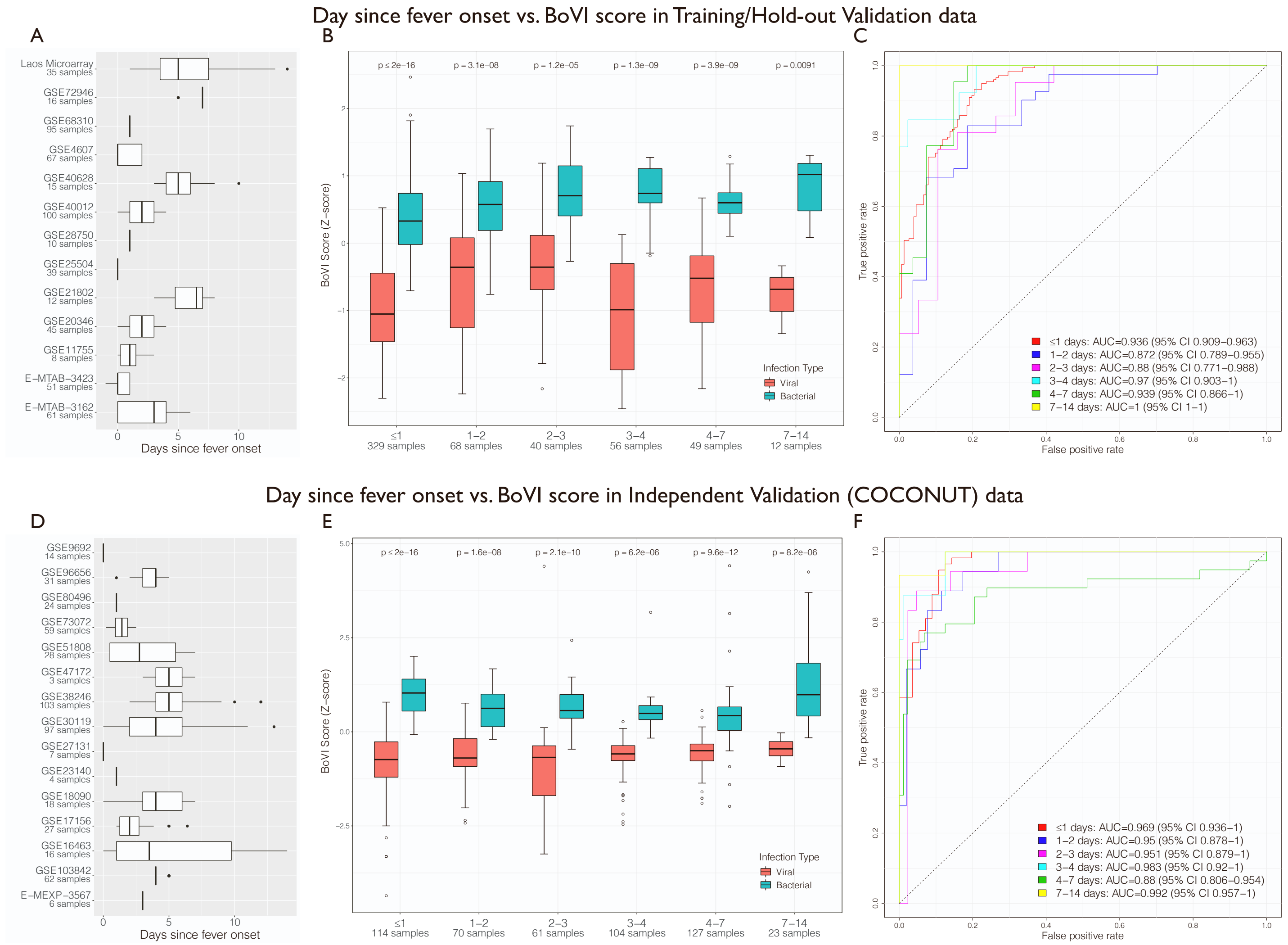

Figure S10. The BoVI signature effectively distinguishes between bacterial and viral infection up to two weeks after the first incidence of febrile symptoms. Performance of the BoVI signature was calculated for different windows of the number of days since fever onset across training, hold-out validation, and independent validation samples. Only samples that were taken within 14 days of fever onset were used. Overall, there were 554 samples in training/hold-out validation and 499 samples in independent validation that were used. **(A,D)** All samples with information on the number of days since fever onset were plotted to show the distribution within each dataset. **(B,E)** Scores were centered at 0 and then the Z-score was calculated. P-values were calculated using the Mann-Whitney U test. (**C,F**) ROC curves comparing the performance of the BoVI signature across each window of days since fever onset. **Related to Figure 4**.

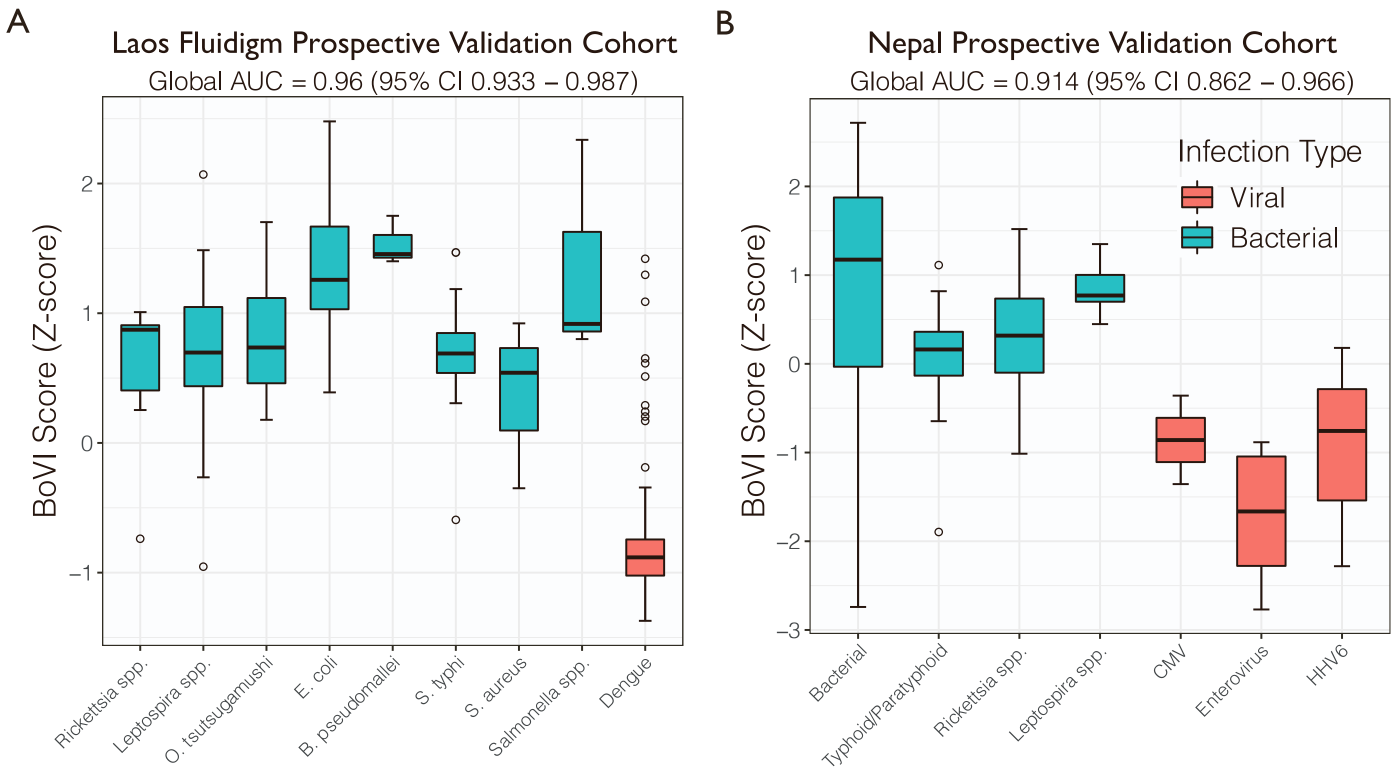

Figure S11. **Performance of the BoVI signature in the prospective validation cohorts.** Scores were centered at 0 and then the Z-score was calculated. Any bacterial infection with a non-specific diagnosis was listed as "Bacterial". Only infecting pathogens that were present in at least 3 samples were displayed. **Related to Figure 5.**

Table S1: Datasets used in the training and hold-out validation cohorts, related to Figure 1 and 2.
The Laos Microarray cohort was used separately for the analysis of existing bacterial vs. viral signatures, but was combined with the other datasets during signature discovery. WB, whole blood; PBMC, peripheral blood mononuclear cells; HRV, human rhinovirus; RSV, respiratory syncytial virus; HSV, herpes simplex virus; CMV, cytomegalovirus; HHV6, human herpesvirus 6; HMPV, human metapneumovirus; HPIV, human parainfluenza virus; BKV, BK virus; PICU, pediatric intensive care unit; ICU, intensive care unit; NICU; neonatal intensive care unit; ARI, acute respiratory infection; CAP, community acquired pneumonia; LRTI, lower respiratory tract infection.

Accession	Author	Platform	Tissue	Location	Demographic	Clinical setting(s)	Bacteria	Viruses	# of healthy samples	# of extracellular bacterial samples	# of intracellular bacterial samples	# of unknown bacterial	# of viral samples
Laos Microarray	Popper	GPL20858	WB	Laos	Adults with systemic infections	Inpatient	*S. typhi*, *R. typhi*, *O. tsutsugamushi*, *E. coli*	Dengue	12	6	30	0	13
GSE64456	Mahajan	GPL10558	WB	USA	Febrile infants ≤ 60 days old	Emergency department	Unknown	Influenza, Enterovirus, HRV, RSV	19	0	0	89	108
GSE72946	Lindow	GPL17077	WB	Brazil	Adults with acute leptospirosis	Inpatient, ICU	*Leptospira* spp.		4	16	0	0	0
E-MTAB-3423	Blohmke	GPL10558	WB	UK	Adults challenged with infective dose of S. typhi	Outpatient	*S. typhi*		40	0	47	0	0
E-TABM-852	Koh	GPL6884	WB	Thailand	Adults with melioidosis	Inpatient	*B. pseudomallei*		10	0	30	0	0
GSE40628	Simmons	GPL16021	WB	Vietnam	Dengue patients at hospital admission	Inpatient		Dengue	4	0	0	0	23
GSE69597	Khaenam	GPL16791	WB	Macedonia, Spain	Acute brucellosis patients	Unknown	*Brucella* spp.		40	0	103	0	0
GSE77087	de Steenhuijsen Piters	GPL10558	WB	USA	Young children with mild and severe RSV disease	Outpatient, Pediatric ward, PICU		RSV	23	0	0	0	81
GSE4607	Wong	GPL570	WB	USA	Septic children in the PICU	PICU	Multiple	Influenza, Rotavirus,	15	48	2	4	13
GSE40396	Hu	GPL10558	WB	USA	Febrile children in the emergency department	Emergency department	*S. aureus*, *E. coli*, *Salmonella* spp.	Adenovirus, Enterovirus, HHV6	22	6	1	0	22
GSE42026	Herberg	GPL6947	WB	UK	Children admitted with febrile infections	Inpatient, PICU	Gram-positive	Influenza, RSV	33	0	0	18	41
GSE21802	Bermejo-Martin	GPL6102	WB	Spain	Adults with septic influenza	ICU		Influenza	4	0	0	0	12

GSE68310	Zhai	GPL10558	WB	USA	Adults with ARIs	Outpatient		Influenza, HRV, RSV, Enterovirus, Coronavirus	98	0	0	0	95
GSE20346	Parnell	GPL6947	WB	Australia	Adults with CAP	ICU	Unknown	Influenza	18	0	0	6	4
GSE40012	Parnell	GPL6947	WB	Australia, Hong Kong	Adults with CAP	ICU	Unknown	Influenza	18	0	0	16	8
GSE66099	Sweeney	GPL570	WB	USA	Septic children in the PICU	PICU	Multiple	Influenza, HSV, HMPV, HPIV, BKV, Adenovirus, CMV	47	103	0	4	11
GSE60244	Suarez	GPL10558	WB	USA	Adults hospitalized with LRTI	Inpatient	Unknown		40	0	0	22	0
GSE11755	Emonts	GPL570	WB	Netherlands	Children in PICU with meningococcal sepsis	PICU	Unknown		3	0	0	6	0
GSE28750	Sutherland	GPL570	WB	Australia	Adults with community-acquired bacterial sepsis	ICU	Unknown		20	0	0	10	0
GSE29161	Thuny	GPL6480	WB	France	Adults with native valve-infected endocarditis	Inpatient	Unknown		5	0	0	5	0
GSE33341	Ahn	GPL571	WB	USA	Adults with septic bloodstream infections	Inpatient	*E. coli* , *S. aureus*		43	51	0	0	0
GSE40586	Lill	GPL6244	WB	Estonia	Bacterial meningitis patients	Inpatient	Multiple		18	19	1	1	0
GSE69528	Khaenam	GPL10558	WB	Thailand	Adults with bacterial sepsis	Unknown	Multiple		28	45	32	5	0
E-MTAB-3162	van de Weg	GPL570	WB	Indonesia	Indonesian patients > 14 years old with uncomplicated and severe dengue	Outpatient, Inpatient		Dengue	15	0	0	0	61
GSE25504 GPL13667	Dickinson	GPL13667	WB	UK	Septic neonates	Neonatal ward, NICU	Multiple	HRV, CMV	6	7	0	2	3
GSE25504 GPL6947	Dickinson	GPL6947	WB	UK	Septic neonates	Neonatal ward, NICU	Multiple	CMV	32	22	0	2	1
GSE13015 GPL6106	Pankla	GPL6106	WB	Thailand	Adults with bacterial sepsis	Unknown	Multiple		3	18	26	1	0
GSE13015 GPL6947	Pankla	GPL6947	WB	Thailand	Adults with bacterial sepsis	Unknown	Multiple		5	7	8	0	0

GSE38900 GPL10558	Mejias	GPL10558	WB	USA	Children with acute LRTI	Outpatient, Inpatient	RSV	8	0	0	0	28
GSE38900 GPL6884	Mejias	GPL6884	WB	USA, Finland	Children with acute LRTI	Outpatient, Inpatient	Influenza, HRV, RSV	31	0	0	0	153
GSE67059 GPL6947	Heinonen	GPL6947	WB	USA, Spain, Finland	Previously healthy children with asymptomatic or symptomatic HRV	Outpatient, Inpatient	HRV	21	0	0	0	82
GSE67059 GPL10558	Heinonen	GPL10558	WB	USA, Spain, Finland	Previously healthy children with asymptomatic or symptomatic HRV	Outpatient, Inpatient	HRV	16	0	0	0	32

Table S2: COCONUT co-normalized datasets used for independent validation of the BoVI signature, related to Figure 1 and 2.

WB, whole blood; PBMC, peripheral blood mononuclear cells; HRV, human rhinovirus; RSV, respiratory syncytial virus; ARI, acute respiratory infection; ICU, intensive care unit; ED, emergency department; PICU, pediatric intensive care unit.

Accession	Author	Platform	Tissue	Location	Demographic	Clinical setting(s)	Bacteria	Viruses	# of healthy samples	# of extracellular bacterial samples	# of intracellular bacterial samples	# of unknown bacterial	# of viral samples
GSE7000 GPL4857	Thompson	GPL4857	WB	Vietnam	Patients with typhoid fever	Inpatient	*S. typhi* , *S. enterica*		15	0	21	0	0
GSE7000 GPL4858	Thompson	GPL4858	WB	Vietnam	Patients with typhoid fever	Inpatient	*S. typhi* , *S. enterica*		3	0	19	0	0
GSE51808	Kwissa	GPL13158	WB	Thailand	Patients with dengue fever or dengue hemorrhagic fever	Inpatient		Dengue	9	0	0	0	28
E-MEXP-3567	Irwin	GPL96	WB	Malawi	Children with bacterial meningitis or pneumonia	ED	*S. pneumoniae*		3	6	0	0	0
GSE17156	Zaas	GPL571	WB	USA, UK	Adults with respiratory viral infection	Outpatient		Influenza, HRV, RSV	56	0	0	0	27
GSE103842	Rodriguez-Fernandez	GPL10558	WB	USA	Young children hospitalized with bronchiolitis	Inpatient		RSV	12	0	0	0	62
GSE30119	Banchereau	GPL6947	WB	USA	Children with community acquired Staph infection	Inpatient, PICU	*S. aureus*		44	99	0	0	0
GSE96656	Popper	GPL20858	WB	Nicaragua	Dengue patients	Inpatient, ICU		Dengue	9	0	0	0	31
GSE47172	Kulohoma	GPL96	WB	Malawi	Children with pneumococcal meningitis	Inpatient	*S. pneumoniae*		3	6	0	0	0
GSE80496	Herberg	GPL6883	WB	UK, Spain, Netherland, USA	Children < 17 years old with meningococcal disease	Inpatient, PICU	*N. meningitidis*		21	24	0	0	0

GSE82050	Tang	GPL21185	WB	Germany	Adults with influenza	Inpatient, ICU		Influenza	15	0	0	0	24
E-MTAB-5195	Jong	GPL570	WB	Netherlands	Infants with RSV	Inpatient, ICU		RSV	4	0	0	0	39
GSE73072 (RSV DEE1)	Liu	GPL14604	WB	USA	Patients in the acute phase of a viral challenge study	Outpatient		RSV	20	0	0	0	9
GSE73072 (H3N2 DEE2)	Liu	GPL14604	WB	USA	Patients in the acute phase of a viral challenge study	Outpatient		Influenza	17	0	0	0	9
GSE73072 (H1N1 DEE3)	Liu	GPL14604	WB	USA	Patients in the acute phase of a viral challenge study	Outpatient		Influenza	22	0	0	0	9
GSE73072 (H1N1 DEE4)	Liu	GPL14604	WB	USA	Patients in the acute phase of a viral challenge study	Outpatient		Influenza	19	0	0	0	5
GSE73072 (H3N2 DEE5)	Liu	GPL14604	WB	USA	Patients in the acute phase of a viral challenge study	Outpatient		Influenza	21	0	0	0	8
GSE73072 (HRV UVA)	Liu	GPL14604	WB	USA	Patients in the acute phase of a viral challenge study	Outpatient		HRV	20	0	0	0	8
GSE73072 (HRV DUKE)	Liu	GPL14604	WB	USA	Patients in the acute phase of a viral challenge study	Outpatient		HRV	26	0	0	0	11
GSE9692	Cvijanovich	GPL570	WB	USA	Children with septic shock	PICU	*S. aureus*, *Streptococcus* spp., *Neisseria* spp.	Adenovirus	15	13	0	0	1
GSE19491	Berry	GPL6947	WB	UK, South Africa	Patients with febrile bacterial infection	Unknown	*S. pyogenes*, *Staphylococcus* spp.		18	52	0	0	0
GSE68004	Jaggi	GPL10558	WB	USA	Children with bacterial or viral infection	Inpatient	*S. pyogenes*	Adenovirus	37	14	0	0	19
GSE27131	Berdal	GPL6244	WB	Norway	Adults with influenza	ICU		Influenza	7	0	0	0	7

GSE38246	Popper	GPL15615	PBMC	Nicaragua	Children hospitalized with dengue infection	Inpatient		Dengue	8	0	0	0	105
GSE18090	Nascimento	GPL570	PBMC	Brazil	Adults with early acute febrile dengue infection	Inpatient		Dengue	8	0	0	0	18
GSE6269 GPL96	Ramilo	GPL96	PBMC	USA	Children with bacterial or viral sepsis	ED	*E. coli* , *S. aureus* , *S. pneumoniae*	Influenza	6	73	0	0	18
GSE34205	Ioannidis	GPL570	PBMC	USA	Children with ARIs	Inpatient		Influenza, RSV	22	0	0	0	79
GSE23140	Liu	GPL6254	PBMC	USA	Children with acute otitis media	Inpatient	*S. pneumoniae*		4	4	0	0	0
GSE16129 GPL6106	Ardura	GPL6106	PBMC	USA	Children with invasive staph infections	Inpatient	*S. aureus*		9	9	0	0	0
GSE16129 GPL96	Ardura	GPL96	PBMC	USA	Children with invasive staph infections	Inpatient	*S. aureus*		10	46	0	0	0
GSE16463	Tantibhedhy angkul	GPL6102	PBMC	Thailand	Patients with rickettsial or dengue infection	Inpatient	*R. typhi* , *O. tsutsugamushi*	Dengue	2	0	11	0	7
GSE11907 GPL96	Chaussabel	GPL96	PBMC	USA	Patients with bacterial infection	Unknown	*E. coli* , *S. aureus*		12	40	0	0	0

Table S3: Demographic characteristics of the subjects used for signature training, hold-out validation, and independent validation, related to Figure 1 and 2.

Age and sex characteristics across datasets, for each analysis phase. The first table summarizes both healthy samples and samples with infection, and the second table summarizes just the samples with infection. WB, whole blood; PBMC, peripheral blood mononuclear cells.

All samples (includes healthy controls)		Training	Hold-out Validation	Independent Validation (COCONUT)	Independent Validation (Individual)	Total
	Number of Datasets	28	22	24	4	
	Number of Samples	1678	633	1458	431	4200
	≤5 years old	684	271	332	30	1317
	>5 and ≤18 years old	39	14	202	16	271
	>18 and ≤65 years old	164	45	100	0	309
	>65 years old	48	12	15	0	75
	Unknown age	743	291	809	385	2228
	Female	699	274	675	214	1862
	Male	979	359	783	217	2338

Only samples with infection		Training	Hold-out Validation	Independent Validation (COCONUT)	Independent Validation (Individual)	Total
	Number of Datasets	28	22	24	4	
	Number of Samples	1175	435	961	431	3002
	≤5 years old	530	213	284	30	1057
	>5 and ≤18 years old	32	8	154	16	210
	>18 and ≤65 years old	107	25	53	0	185
	>65 years old	39	8	14	0	61
	Unknown age	467	181	456	385	1489
	Female	490	176	444	214	1324
	Male	685	259	517	217	1678

Table S4: Infecting pathogen reported for the subjects used in training, hold-out validation, and independent validation, related to Figure 2.

RSV, respiratory syncytial virus; CoNS, coagulase-negative staphylococci; HHV6, human herpesvirus 6; CMV, cytomegalovirus; HSV, herpes simplex virus; BKV, BK virus; HMPV, human

Pathogen	Pathogen Category	Training	Hold-out Validation	Independent Validation (COCONUT)	Independent Validation (Individual)
B. pseudomallei	Intracellular bacteria	74	17		
***Brucella* spp.**	Intracellular bacteria	72	31		
S. typhi	Intracellular bacteria	35	19	38	
O. tsutsugamushi	Intracellular bacteria	9	3	4	
R. typhi	Intracellular bacteria	9	2	7	
***Salmonella* spp.**	Intracellular bacteria	4	2		
L. monocytogenes	Intracellular bacteria	1			
***Rickettsia* spp.**	Intracellular bacteria	1	1		
S. enterica	Intracellular bacteria			2	
S. aureus	Extracellular bacteria	53	23	208	19
E. coli	Extracellular bacteria	37	10	51	
S. pneumoniae	Extracellular bacteria	23	12	31	9
**CoNS**	Extracellular bacteria	17	2		
***Leptospira* spp.**	Extracellular bacteria	16			
S. pyogenes	Extracellular bacteria	15	7	28	
K. pneumoniae	Extracellular bacteria	14	3		
S. agalactiae	Extracellular bacteria	13	5	2	
***Enterococcus* spp.**	Extracellular bacteria	11	4		
N. meningitidis	Extracellular bacteria	9	2	24	
**Viridans Streptococci**	Extracellular bacteria	8			
***Corynebacterium* spp.**	Extracellular bacteria	6	1		
**Enterovirus/Rhinovirus**	Extracellular bacteria	6	2		

***Neisseria* spp.**	Extracellular bacteria	6	1	2	
H. Influenzae	Extracellular bacteria	4	2		
S. marcescens	Extracellular bacteria	4			
A. baumannii	Extracellular bacteria	3	1		
P. aeruginosa	Extracellular bacteria	3			
S. epidermidis	Extracellular bacteria	3			
***Aeromonas* spp.**	Extracellular bacteria	2			
E. faecalis	Extracellular bacteria	2			
E. faecium	Extracellular bacteria	2			
***Micrococcus* spp.**	Extracellular bacteria	2	2		
A. hydrophila	Extracellular bacteria	1			
A. lwoffii	Extracellular bacteria	1	1		
***Acinetobacter* spp.**	Extracellular bacteria	1			
C. difficile	Extracellular bacteria	1	1		
C. freundii	Extracellular bacteria	1			
E. corrodens	Extracellular bacteria	1	1		
***Enterobacter* spp.**	Extracellular bacteria	1	2		
M. catarrhalis	Extracellular bacteria	1			
***Pseudomonas* spp.**	Extracellular bacteria	1	1		
S. anginosus	Extracellular bacteria	1			
S. suis	Extracellular bacteria	1			
E. cloacae	Extracellular bacteria		1		
K. oxytoca	Extracellular bacteria		1		
***Sphingomonas* spp.**	Extracellular bacteria		1		
***Staphylococcus* spp.**	Extracellular bacteria			40	
**Unspecified bacteria**	Unspecified bacteria	116	49		148
**Gram Positive Bacteria**	Unspecified bacteria	14	4		
**Gram Negative Bacteria**	Unspecified bacteria	4	4		
**RSV**	Virus	178	63	170	
**Rhinovirus**	Virus	118	53	29	
**Influenza**	Virus	115	46	116	18

**Dengue**	Virus	74	23	189	
**Enterovirus**	Virus	39	19		
**Influenza/Rhinovirus**	Virus	15	4		
**HHV6**	Virus	8			
**Adenovirus**	Virus	5	4	20	
**CMV**	Virus	3	2		
**HSV**	Virus	3	2		
**Rotavirus**	Virus	2			
**Varicella**	Virus	2			
**BKV**	Virus	1			
**Coronavirus**	Virus	1	1		
**HMPV**	Virus	1			
**Parainfluenza**	Virus	1			
**Unspecified virus**	Virus				237

Table S5: Demographic and clinical characteristics of the subjects from the Laos Microarray cohort, related to Figure 1.

Slide	ID	Diagnosis	Age	Sex	Days since fever onset
HOAD086	10205	Dengue	20	Male	5
HOAD119	10262	Dengue	24	Male	5
HOAD159	10307	Dengue	32	Female	3
HOAD167	10371	Dengue	19	Female	5
HOAD100	10424	Dengue	17	Male	6
HOAD093	10528	Dengue	20	Male	8
HOAD124	10607	Dengue	20	Female	4
HOAD084	10608	Dengue	21	Male	6
HOAD162	10637	Dengue	23	Female	5
HOAD117	10639	Dengue	19	Male	4
HOAD082	10663	Dengue	24	Female	5
HOAD128	10669	Dengue	23	Female	6
HOAD127	10672	Dengue	23	Female	7
HOAD135	10122	*E. coli*	74	Female	4
HOAD090	10240	*E. coli*	80	Female	2
HOAD160	10374	*E. coli*	68	Female	4
HOAD165	10437	*E. coli*	62	Female	6
HOAD122	11066	*E. coli*	20	Female	4
HOAD106	11474	*E. coli*	25	Female	4
HOAD140	BDC22	Healthy	NA	NA	NA
HOAD169	BDC23	Healthy	NA	NA	NA
HOAD110	BDC26A	Healthy	NA	NA	NA
HOAD112	BDC27	Healthy	NA	NA	NA
HOAD144	BDC29	Healthy	NA	NA	NA
HOAD173	BDC31	Healthy	NA	NA	NA
HOAD147	BDC33	Healthy	NA	NA	NA
HOAD107	BDC35	Healthy	NA	NA	NA
HOAD101	BDC37	Healthy	NA	NA	NA

HOAD172	BDC38	Healthy	NA	NA	NA
HOAD109	BDC39A	Healthy	NA	NA	NA
HOAD168	BDC40	Healthy	NA	NA	NA
HOAD102	10039	*Orientia tsutsugamushi*	60	Female	5
HOAD095	10375	*Orientia tsutsugamushi*	28	Male	7
HOAD158	10532	*Orientia tsutsugamushi*	30	Male	10
HOAD149	10584	*Orientia tsutsugamushi*	27	Male	12
HOAD096	10653	*Orientia tsutsugamushi*	18	Female	11
HOAD089	11106	*Orientia tsutsugamushi*	50	Female	14
HOAD164	11114	*Orientia tsutsugamushi*	31	Female	11
HOAD091	10654A	*Orientia tsutsugamushi*	23	Female	9
HOAD138	N058	*Orientia tsutsugamushi*	NA	NA	NA
HOAD103	N248	*Orientia tsutsugamushi*	NA	NA	NA
HOAD145	N250	*Orientia tsutsugamushi*	NA	NA	NA
HOAD139	N363	*Orientia tsutsugamushi*	NA	NA	NA
HOAD161	10191	*Rickettsia typhi*	44	Female	7
HOAD130	10551	*Rickettsia typhi*	46	Male	6
HOAD081	11184	*Rickettsia typhi*	25	Male	12
HOAD132	11357	*Rickettsia typhi*	18	Female	6
HOAD136	11452	*Rickettsia typhi*	22	Female	9
HOAD088	11379A	*Rickettsia typhi*	30	Female	4
HOAD171	N120	*Rickettsia typhi*	NA	NA	NA
HOAD137	N240	*Rickettsia typhi*	NA	NA	NA
HOAD143	N298	*Rickettsia typhi*	NA	NA	NA
HOAD166	N303	*Rickettsia typhi*	NA	NA	NA
HOAD108	N397	*Rickettsia typhi*	NA	NA	NA
HOAD129	9774	*S. typhi*	20	Male	6
HOAD131	10259	*S. typhi*	37	Male	15
HOAD142	N102	*S. typhi*	NA	NA	NA
HOAD146	N259	*S. typhi*	NA	NA	NA
HOAD163	N367	*S. typhi*	NA	NA	NA
HOAD111	N372	*S. typhi*	NA	NA	NA
HOAD104	N387	*S. typhi*	NA	NA	NA

Table S6: Individual datasets used for independent validation of the BoVI signature, related to Figure 3.

WB, whole blood; PBMC, peripheral blood mononuclear cells; ARI, acute respiratory infection; PICU, pediatric intensive care unit; ED, emergency department

Accession	Author	Platform	Tissue	Location	Demographic	Clinical setting(s)	Bacteria	Viruses	# of healthy samples	# of extracellular bacterial samples	# of intracellular bacterial samples	# of unknown bacterial	# of viral samples
GSE72809	Herberg	GPL10558	WB	UK, Spain, Netherland, USA	Children <17 years old with bacterial or viral infections	Inpatient, PICU	Unknown	Unknown	0	0	0	52	92
GSE72810	Herberg	GPL6947	WB	UK, Spain, Netherland, USA	Children <17 years old with bacterial or viral infections	Inpatient, PICU	Unknown	Unknown	0	0	0	23	28
GSE63990	Tsalik	GPL571	WB	USA	Patients with ARIs	ED, Inpatient	Unknown	Unknown	0	0	0	73	117
GSE6269 GPL570	Ramilo	GPL570	PBMC	USA	Children with bacterial or viral sepsis	ED	*S. aureus, S. pneumoniae*	Influenza	0	12	0	0	10
GSE6269 GPL2507	Ramilo	GPL2507	PBMC	USA	Children with bacterial or viral sepsis	ED	*S. aureus, S. pneumoniae*	Influenza	0	16	0	0	8

Table S7: Assessment of promising signatures in the training and hold-out validation sets, related to Figure 2.
Performance of the differential expression filters and corresponding feature selection methods that were used to identify potential signatures. The signature that was chosen for independent validation is highlighted in green. AUROC, area under the receiving operator characteristics curve; ES, effect size; FDR, false discovery rate; abridged BSS, Abridged Best Subset Selection; min.1ci, minimum AUROC within the confidence interval of the maximum AUROC.

Differential expression thresholds	Feature selection method	Number of genes	Training			Hold-out Validation		
			All bacterial vs. viral	EC bacterial vs. viral	IC bacterial vs. viral	All bacterial vs. viral	EC bacterial vs. viral	IC bacterial vs. viral
ES ≥ 0.6; FDR ≤ 1%	N/A	417	0.864 (0.843-0.885)	0.945 (0.926-0.965)	0.723 (0.679-0.766)	0.845 (0.808-0.882)	0.937 (0.9-0.974)	0.725 (0.654-0.796)
	Forward Search	20	0.962 (0.951-0.973)	0.974 (0.96-0.988)	0.953 (0.933-0.974)	0.94 (0.917-0.963)	0.964 (0.936-0.993)	0.937 (0.898-0.976)
	Backward Search	44	0.968 (0.958-0.978)	0.979 (0.966-0.991)	0.96 (0.941-0.979)	0.953 (0.932-0.973)	0.975 (0.952-0.999)	0.947 (0.911-0.983)
	Backward Search + abridged BSS (max AUROC)	38	0.968 (0.958-0.978)	0.978 (0.966-0.991)	0.961 (0.943-0.98)	0.953 (0.933-0.974)	0.975 (0.952-0.999)	0.952 (0.918-0.986)
	Backward Search + abridged BSS (min.1ci AUROC)	15	0.959 (0.947-0.97)	0.964 (0.948-0.98)	0.956 (0.935-0.976)	0.95 (0.928-0.971)	0.96 (0.93-0.99)	0.957 (0.925-0.99)
ES ≥ 0.8; FDR ≤ 1%	N/A	76	0.89 (0.871-0.909)	0.953 (0.935-0.971)	0.773 (0.733-0.814)	0.904 (0.874-0.933)	0.957 (0.927-0.988)	0.83 (0.769-0.89)
	Forward Search	14	0.942 (0.928-0.955)	0.96 (0.943-0.977)	0.918 (0.891-0.944)	0.93 (0.905-0.955)	0.953 (0.921-0.986)	0.924 (0.882-0.967)
	Backward Search	19	0.943 (0.93-0.957)	0.962 (0.945-0.978)	0.913 (0.886-0.94)	0.941 (0.918-0.964)	0.959 (0.921-0.989)	0.942 (0.904-0.979)
	Backward Search + abridged BSS (max AUROC)	18	0.943 (0.929-0.957)	0.96 (0.943-0.977)	0.913 (0.886-0.943)	0.94 (0.917-0.964)	0.956 (0.925-0.988)	0.942 (0.905-0.98)
	Backward Search + abridged BSS (min.1ci AUROC)	6	0.934 (0.919-0.949)	0.951 (0.932-0.969)	0.914 (0.886-0.941)	0.928 (0.903-0.954)	0.956 (0.925-0.987)	0.919 (0.875-0.963)
Top 100 SAM scores	N/A	100	0.874 (0.854-0.894)	0.944 (0.924-0.964)	0.742 (0.699-0.784)	0.877 (0.844-0.911)	0.948 (0.914-0.982)	0.773 (0.706-0.841)
	Forward Search	15	0.951 (0.938-0.963)	0.968 (0.952-0.983)	0.925 (0.9-0.951)	0.947 (0.925-0.969)	0.965 (0.937-0.993)	0.944 (0.907-0.981)
	Backward Search	16	0.951 (0.939-0.964)	0.966 (0.95-0.981)	0.932 (0.908-0.957)	0.946 (0.924-0.968)	0.967 (0.939-0.994)	0.94 (0.902-0.978)
	Backward Search + abridged BSS (max AUROC)	15	0.951 (0.939-0.964)	0.967 (0.952-0.983)	0.928 (0.903-0.953)	0.948 (0.926-0.969)	0.967 (0.939-0.994)	0.944 (0.907-0.981)
	**Backward Search + abridged BSS (min.1ci AUROC)**	8	**0.942 (0.928-0.955)**	**0.958 (0.941-0.975)**	**0.92 (0.894-0.947)**	**0.947 (0.925-0.969)**	**0.965 (0.938-0.993)**	**0.941 (0.904-0.979)**

Table S8: Active TB and HIV datasets used for validation of the BoVI signature, related to Figure 3.

Active TB samples were excluded if they exhibited an atypical presentation of TB (e.g. extrapulmonary TB), were HIV positive or had an ambiguous HIV status, had any other significant comorbidities, or had been successfully treated for TB. HIV samples were excluded if they were undergoing HIV treatment. Any datasets that did not contain all 8 genes in the BoVI signature were excluded. TB, tuberculosis; HIV, human immunodeficiency virus; WB, whole blood; PBMC, peripheral blood mononuclear cells.

Accession	Author	Platform	Tissue	Location	Demographic	Clinical setting(s)	Pathogen	# of healthy samples	# of bacterial samples	# of viral samples
GSE42834	Bloom	GPL10558	WB	UK, France, South Africa	Adults with TB,	Inpatient	TB	118	40	0
GSE19491	Berry	GPL6947	WB	UK, South Africa	Patients with TB or other febrile conditions	Inpatient, outpatient	TB	18	61	0
GSE28623	Maertzdorf	GPL4133	WB	The Gambia	Adults with TB	Outpatient	TB	37	46	0
GSE34608	Maertzdorf	GPL6480	WB	Germany	Adults with TB	Outpatient	TB	18	8	0
GSE41055	Verhagen	GPL5175	WB	Venezuela	Children with ATB or LTBI	Outpatient	TB	9	2	0
GSE83456	Blankley	GPL10558	WB	UK	Adults with TB	Inpatient	TB	61	45	0
GSE81746	Sambarey	GPL17077	WB	India	Adults with TB	Unknown	TB	2	4	0
E-GEOD-25534	Maertzdorf	GPL1708	WB	South Africa	Patients with TB	Inpatient, outpatient	TB	13	44	0
E-MTAB-3260	Noursadeghi	GPL14550	WB	Unknown	Patients with ATB	Inpatient, outpatient	TB	11	16	0
E-MTAB-8290	Turner	GPL18573	WB	South Africa	Adults presenting with possible pulmonary TB	Outpatient	TB	99	37	0

GSE100150	Altman	GPL6884	WB	USA, UK, Finland, Spain	Patients with a variety of clinical conditions	Unknown	TB	11	23	0
GSE114192	Eckold	GPL18573	WB	South Africa, Romania, Indonesia, Peru	TB patients with diabetes comorbidity	Outpatient	TB	36	46	0
GSE122485	Sambarey	GPL15520	WB	India	Adults with TB	Outpatient	TB	3	4	0
GSE107995	Singhania	GPL20301	WB	India, UK, Kenya, Somalia, Afghanistan, Uganda, Sudan, Tanzania, Pakistan, Poland, etc.	Individuals with ATB and LTBI	Inpatient	TB	119	53	0
GSE100150	Altman	GPL6884	WB	USA	Adult patients diagnosed with HIV infection	Unknown	HIV	35	0	28
GSE29429 GPL10558	Chaussabel	GPL10558	WB	Africa	Adult patients diagnosed with acute HIV infection	Unknown	HIV	17	0	30
GSE29429 GPL6947	Chaussabel	GPL6947	WB	USA, Africa	Adult patients diagnosed with acute HIV infection	Unknown	HIV	38	0	28
GSE29536	Banchereau	GPL6102	WB	USA	Patients diagnosed with acute HIV infection	HIV testing sites	HIV	36	0	9
GSE4124	Montano	GPL571	PBMC	Botswana	HIV+ mothers	Outpatient	HIV	20	0	25

GSE77939	Singh	GPL15207	WB	India	Patients diagnosed with HIV infection	Outpatient	HIV	4	0	5

Table S9: Multivariable logistic regression of BoVI score, age, sex, and race, related to Figure 4 and Figure S7-S9.

*, **, *** indicates significance at p<0.05, p<0.01, and p<0.001, respectively.

	Estimate	Standard error	Z-score	P value
**Intercept**	-0.485	0.414	-1.17	0.242
**BoVI score**	3.703	0.231	16.006	<2e-16***
**Age**	0.011	0.005	2.344	0.019*
**Sex (Male)**	0.067	0.197	0.342	0.732
**Race (Asian)**	0.892	0.552	1.615	0.106
**Race (Black)**	1.344	0.448	3.002	0.003**
**Race (White)**	0.313	0.431	0.728	0.466

Table S10: Demographic and clinical characteristics of the subjects from the Laos Fluidigm prospective validation cohort, related to Figure 5 and Figure S11.
JEV; Japanese encephalitis virus.

ID	Sponsor ID	Diagnosis	Age	Sex	Days since fever onset	Sweeney7 Score	BoVI Score
A3927_047	9718	*Rickettsia* sp.	49	female	9	-0.2842763	1.36086713
A3927_048	9787	Dengue	16	female	3	-4.5055377	-4.135445
A3927_049	9788	Dengue	26	male	3	-3.8083701	-3.633376
A3927_050	9806	JEV	19	male	8	-0.0015691	-0.1778702
A3927_051	9812	Dengue	20	female	3	-5.8282189	-4.462533
A3927_052	9819	Dengue	19	male	4	-4.1586179	-2.7141281
A3927_053	9831	Dengue	21	female	11	-0.6100248	-0.4813855
A3927_054	9850	Dengue	21	female	4	-4.7359659	-3.7685813
A3927_055	9875	Dengue	33	male	4	-4.4376922	-3.3014833
A3927_056	9878	Dengue	20	female	4	-3.7528465	-2.518329
A3927_057	9882	*Leptospira* sp.	32	male	30	0.89918285	1.75503654
A3927_058	9883	Dengue	16	male	14	-4.3279038	-3.7107889
A3927_059	9887	*Leptospira* sp.	75	female	1	0.77921931	0.45155862
A3927_060	9904	*O. tsutsugamushi*	20	male	1	-0.8374049	0.18251192
A3927_061	9905	Dengue	17	female	13	-5.5880167	-3.5077974
A3927_062	9910	Dengue	23	female	5	-3.6568906	-2.9977703
A3927_063	9917	*Leptospira* sp.	26	male	7	-0.7509469	-0.2045696
A3927_064	9922	Dengue	16	male	5	-4.1960872	-3.500152
A3927_065	9923	Dengue	50	female	3	-5.1648068	-4.2582222
A3927_066	9948	*E. coli*	28	female	5	2.5847888	4.02212649
A3927_067	9949	Dengue	19	female	7	-4.1496766	-3.3656053
A3927_068	9954	Dengue	25	male	5	-3.8874533	-2.6515083
A3927_069	9967	Dengue	19	female	2	-4.4789349	-4.2006006
A3927_070	9980	Dengue	19	male	6	-3.5164916	-2.7146446
A3927_071	9989	*B. pseudomallei*	68	male	14	2.57218257	3.53341337
A3927_072	9997	Dengue	36	male	6	-4.9263644	-4.5716664
A3927_073	10040	*O. tsutsugamushi*	65	female	5	-2.0295993	0.07732783
A3927_075	10045	Dengue	22	male	3	-4.2149842	-3.0465148
A3927_076	10046	Dengue	17	female	8	-4.4877463	-2.8077175
A3927_077	10052	Dengue	23	male	6	-1.1849604	0.68127555
A3927_078	10067	Dengue	29	female	4	-5.5081609	-3.983312
A3927_080	10084	Dengue	16	female	5	-4.2470734	-3.5888749
A3927_081	10086	Dengue	31	male	4	-4.0065337	-2.971861
A3927_082	10090	*Leptospira* sp.	30	female	5	-2.0361731	-1.6176782

A3927_083	10092	Dengue	17	female	6	-4.6525075	-3.6791776
A3927_084	10095	Dengue	16	male	6	-4.300987	-2.9086558
A3927_085	10113	Dengue	21	female	4	-4.6290395	-3.3002791
A3927_086	10135	Dengue	19	female	5	-6.301492	-2.8401764
A3927_087	10141	Dengue	22	male	5	-4.0997869	-3.4965484
A3927_088	10151	Dengue	23	female	3	-6.1992554	-4.493835
A3927_089	10153	Dengue	16	male	6	-4.1479984	-3.478402
A3927_090	10179	Dengue	18	female	3	-4.055814	-3.6643872
A3927_091	10183	Dengue	46	female	2	-4.3256663	-4.1770774
A3927_092	10184	Dengue	26	female	7	-4.3524515	-3.1708999
A3927_093	10186	Dengue	35	male	4	-4.0783841	-2.8054071
A3927_094	10195	Dengue	22	male	4	-3.7983135	-3.0740389
A3927_095	10203	Dengue	17	female	8	-4.0067396	-4.1001964
A3927_096	10217	Dengue	56	female	3	-1.4357707	-0.2570482
A3927_097	10227	Dengue	19	female	3	-4.5347737	-4.3002903
A3927_098	10231	Dengue	21	male	6	-3.9751261	-3.0535746
A3927_099	10232	Dengue	19	female	7	-4.1229678	-3.7857059
A3927_100	10233	Dengue	32	male	5	-3.8024054	-2.9473052
A3927_101	10237	Dengue	17	female	6	-4.7077025	-2.9432092
A3927_102	10239	Dengue	16	male	5	-3.6883384	-3.6664561
A3927_103	10241	Group B Streptococcus	65	male	5	1.13771676	3.01747692
A3927_104	10248	Dengue	30	male	4	-5.4214761	-3.480253
A3927_105	10249	Dengue	22	female	5	-4.7045711	-3.8841779
A3927_106	10251	Dengue	18	male	4	-4.1115379	-3.5632562
A3927_107	10273	Dengue	21	male	8	-3.1020946	-2.9902377
A3927_108	10274	Dengue	22	female	4	-4.7045008	-3.7467694
A3927_109	10276	Dengue	26	female	4	-5.5829015	-3.8923438
A3927_110	10279	Dengue	33	male	4	-3.5809608	-2.9977932
A3927_111	10288	Dengue	25	female	1	-4.2751769	-2.9325343
A3927_112	10293	*Leptospira* sp.	25	male	4	-0.1836041	1.1624809
A3927_113	10320	Dengue	23	male	4	-3.0891238	-3.226352
A3927_114	10321	Dengue	24	female	4	-4.4235776	-3.456484
A3927_115	10328	Dengue	17	female	3	-4.1718115	-3.0192062
A3927_116	10329	Dengue	23	male	5	-5.4867136	-4.1919911
A3927_118	10342	Dengue	20	male	4	-4.1309704	-3.2435833
A3927_119	10344	Dengue	23	female	5	-4.7699874	-3.6302574
A3927_120	10347	Dengue	30	male	5	-4.0124122	-3.691171
A3927_121	10348	Dengue	17	male	3	-4.6413426	-4.1914877
A3927_122	10378	Dengue	19	female	4	-4.2840491	-3.1607955
A3927_123	10379	Dengue	18	female	7	-4.0139017	-3.9830861

A3927_124	10394	Dengue	16	female	5	-4.5486072	-3.2067688
A3927_125	10410	Dengue	37	male	0	-4.614831	-3.80356
A3927_126	10423	Dengue	24	female	4	-5.716688	-3.7623149
A3927_127	10426	Dengue	32	male	4	-3.824037	-3.3528182
A3927_128	10438	Dengue	24	male	4	-4.1957231	-3.5803347
A3927_129	10439	Dengue	26	female	5	-3.3921763	-3.0867067
A3927_130	10441	Dengue	19	male	3	-4.3955831	-3.8022082
A3927_131	10444	Dengue	50	male	12	0.49239044	-0.5705381
A3927_132	10447	*Leptospira* sp.	35	female	17	1.19056242	0.79741472
A3927_133	10451	Dengue	21	male	5	-3.1875275	-3.5418447
A3927_134	10465	*Leptospira* sp.	16	female	14	-4.2672681	-3.4863745
A3927_135	10476	Dengue	24	male	4	-3.4447348	-2.2719929
A3927_136	10478	Dengue	17	male	4	-3.7322377	-3.7170191
A3927_137	10479	Dengue	20	male	4	-3.9731906	-3.627635
A3927_138	10480	Dengue	26	female	12	-2.65517	-1.4991074
A3927_139	10481	Dengue	21	male	6	-3.5780821	-2.7416055
A3927_140	10489	Dengue	29	male	9	0.29060053	2.35418291
A3927_141	10503	Dengue	23	male	5	-4.9412412	-3.1984048
A3927_142	10509	Dengue	16	male	6	-5.2735293	-3.1895555
A3927_143	10510	Dengue	24	male	7	-4.063949	-2.8904876
A3927_144	10511	Flavivirus	67	female	5	-2.9067966	-0.9588956
A3927_145	10513	Dengue	28	male	6	0.52094256	2.67524425
A3927_146	10514	Dengue	18	female	6	-4.8199451	-3.7011998
A3927_147	10515	Dengue	19	female	1	-4.3733874	-3.8601736
A3927_148	10516	Dengue	30	female	6	-5.300171	-3.5965722
A3927_149	10526	Dengue	17	female	5	-4.4309173	-3.1514213
A3927_150	10527	Dengue	33	female	5	-2.4505906	-1.9033776
A3927_151	10529	Dengue	27	female	5	-4.4133988	-2.6392044
A3927_152	10536	Dengue	24	male	6	-4.0224486	-2.5519584
A3927_153	10537	Dengue	24	male	3	-4.6853943	-3.6493829
A3927_154	10542	Dengue	16	male	5	-4.3440396	-3.6762824
A3927_155	10543	Dengue	20	female	4	-4.9890436	-4.0315129
A3927_156	10549	Dengue	24	male	5	-4.4501951	-3.5212294
A3927_157	10550	Dengue	21	male	6	-4.3613183	-3.1998903
A3927_158	10557	Dengue	19	female	3	-4.9256874	-3.4687698
A3927_159	10570	Dengue	21	female	5	-4.4409013	-3.6485339
A3927_160	10573	Dengue	22	male	6	-4.7983169	-3.4013833
A3927_161	10581	Dengue	23	female	2	-5.0993293	-3.1842701
A3927_162	10590	Dengue	24	male	5	-3.7984137	-3.5880525
A3927_163	10591	Salmonella Group B	75	female	1	0.7402411	1.68932446

A3927_164	10609	Dengue	17	male	3	-4.8228601	-3.1758097
A3927_165	10630	*E. coli*	67	female	9	0.85883914	1.64054443
A3927_166	10656	Dengue	22	female	7	-5.3498392	-3.6301059
A3927_167	10670	Dengue	18	male	4	-4.4679868	-3.1880643
A3927_169	10950	Dengue	27	male	5	-4.7657594	-3.4352387
A3927_170	10955	Dengue	20	female	3	-5.2901359	-3.5818298
A3927_171	10962	Dengue	30	female	5	-3.9687157	-3.1281607
A3927_172	10967	Dengue	27	female	4	-1.2336534	0.31947886
A3927_173	10979	Dengue	18	male	5	-4.5916658	-3.515949
A3927_174	10984	Dengue	27	male	4	-4.6327415	-2.6331445
A3927_175	11031	Dengue	18	male	4	-4.0892495	-2.9318089
A3927_176	11041	Dengue	23	male	4	-4.7437689	-2.9202957
A3927_177	11051	Dengue	18	male	5	-5.1329932	-3.0226404
A3927_178	11068	Dengue	31	female	4	-2.1190547	-0.3841236
A3927_180	11074	*Leptospira* sp.	21	female	4	0.35185772	1.66150753
A3927_181	11075	Dengue	20	male	6	-4.2496686	-3.0954013
A3927_182	11076	*Leptospira* sp.	68	female	5	0.67988926	1.27938729
A3927_183	11083	Dengue	16	male	3	-4.8285382	-3.270098
A3927_184	11084	Dengue	38	female	5	-4.9039075	-3.3484396
A3927_185	11087	Dengue	24	male	5	-4.856111	-3.1017652
A3927_186	11104	Dengue	26	male	5	-4.1983643	-3.3501246
A3927_187	11119	Dengue	25	male	4	-5.1314106	-3.9308003
A3927_188	11142	*S. typhi*	18	male	15	-1.0659978	0.8381991
A3927_189	11145	*S. aureus*	20	female	1	0.53365817	1.38131038
A3927_190	11147	Dengue	25	female	7	-3.822148	-2.2039227
A3927_191	11212	Dengue	30	female	2	1.92460644	0.58289779
A3927_192	11240	*Leptospira* sp.	52	female	7	-0.9575956	0.54029979
A3927_193	11243	JEV	20	male	7	-1.5907234	0.62872443
A3927_194	11246	*Leptospira* sp.	40	male	7	2.20900389	2.84546327
A3927_195	11304	Dengue	28	male	4	-4.9883793	-3.2050754
A3927_196	11343	*S. aureus*	18	male	4	-0.2085599	0.39270775
A3927_197	11362	Dengue	64	female	2	0.53261846	1.81559899
A3927_198	11368	*Rickettsia* sp.	30	female	10	-0.7027827	1.60631459
A3927_199	11441	*Leptospira* sp.	26	female	2	-3.0345107	-1.69988
A3927_200	11467	*S. typhi*	24	female	1	-1.1531873	0.62502306
A3927_201	11564	*E. coli*	70	female	5	1.12967343	2.28822574
A3927_202	11567	*S. typhi*	33	female	13	-1.0072912	0.92565394
A3927_203	11579	*S. typhi*	24	female	3	-1.6375075	0.21287683
A3927_204	11582	*S. typhi*	36	female	5	-1.2200625	1.92501691
A3927_205	11584	*S. typhi*	27	male	6	-1.5057356	1.20901186

A3927_206	11614	*Salmonella* sp.	26	female	6	-1.3025954	1.37032192
A3927_207	11620	*E. coli*	61	female	3	2.28093658	3.42002081
A3927_208	11633	*E. coli*	76	female	3	-1.6263572	0.75229182
A3927_209	11655	*S. typhi*	26	male	14	-0.0725324	2.06721521
A3927_210	11659	*S. typhi*	38	male	7	-1.4125439	0.44762989
A3927_211	11666	*S. pneumoniae*	24	male	80	-1.3889904	-0.7265846
A3927_212	11670	*S. typhi*	18	male	3	-3.7272969	-2.5497606
A3927_214	11705	*S. typhi*	19	male	5	-1.135101	0.11199716
A3927_215	11712	*S. typhi*	28	female	7	-1.633431	1.48055402
A3927_216	11744	*E. coli*	26	female	3	-0.5867071	0.63865616
A3927_217	11759	*E. coli*	45	male	0	0.8471719	2.12593253
A3927_218	11770	*K. pneumoniae*	77	male	4	3.00157554	3.75193157
A3927_219	12108	*B. pseudomallei*	56	female	3	2.17134576	2.7679059
A3927_222	12123	*S. typhi*	42	male	15	-1.2246822	-0.2164049
A3927_223	12124	*O. tsutsugamushi*	23	male	15	-0.8035127	0.89797054
A3927_224	12136	*Salmonella* sp.	63	female	7	3.51267616	5.05413188
A3927_225	12171	*E. coli*	16	male	5	0.63284021	1.22290046
A3927_226	12173	*O. tsutsugamushi*	50	female	6	-1.382469	-0.2449159
A3927_227	12179	*Moraxella* sp.	42	male	7	-1.4765506	0.43249978
A3927_229	12183	*O. tsutsugamushi*	46	male	4	-1.7997418	-0.4794779
A3927_230	12233	*O. tsutsugamushi*	42	male	7	-1.0591502	0.59564239
A3927_231	12268	*Leptospira* sp.	20	male	2	2.47822385	2.26163316
A3927_232	12283	*S. typhi*	43	male	14	0.30259321	2.803426
A3927_233	12288	Group G Streptococcus	22	male	34	-3.666012	-2.3129063
A3927_234	12291	*W. paucula*	17	male	5	-2.6244924	-0.8999069
A3927_235	12299	*S. typhi*	30	female	10	-1.0689172	0.89164264
A3927_236	12342	*Leptospira* sp.	38	male	2	3.90388738	4.36180575
A3927_237	12353	*E. coli*	55	female	2	3.1884651	2.84828033
A3927_238	12354	*E. coli*	35	female	2	4.3666286	5.42320768
A3927_239	12407	*O. tsutsugamushi*	25	female	6	-1.7210431	1.00187452
A3927_240	12441	*O. tsutsugamushi*	31	male	8	-0.0524469	2.83037616
A3927_241	12443	*O. tsutsugamushi*	25	male	4	-0.393036	2.95342342
A3927_242	12456	*S. typhi*	36	female	4	-1.3189474	0.12037628
A3927_243	12468	*Rickettsia* sp.	20	female	2	-3.6344279	-2.9278346
A3927_244	12646	*E. coli*	61	male	2	2.42024431	4.97150091
A3927_246	12657	*B. pseudomallei*	46	female	5	0.15965792	2.62399401
A3927_247	12674	*K. pneumoniae*	76	male	4	3.06772443	4.39873173
A3927_248	12697	*S. typhi*	19	female	8	-1.2814548	0.83892971
A3927_250	12822	*Salmonella* sp.	42	female	3	-1.1160205	1.06652316
A3927_251	12828	*O. tsutsugamushi*	30	male	7	-0.8884574	0.9742752

A3927_252	12852	*S. typhi*	16	male	13	-1.2056229	1.79651309
A3927_253	12881	*S. typhi*	25	male	9	-1.5602695	0.66348972
A3927_254	12931	*E. coli*	30	male	4	4.11754219	4.49562445
A3927_255	12986	*O. tsutsugamushi*	30	male	11	-0.9617764	3.40731278
A3927_256	12996	*E. coli*	60	female	7	1.86391068	2.41566424
A3927_258	13037	*Rickettsia* sp.	18	female	7	-2.0307133	-0.3530427
A3927_259	13043	*E. coli*	73	male	6	0.39648477	2.45709271
A3927_260	13077	*E. coli*	40	female	7	2.50582929	3.01299696
A3927_261	13095	*O. tsutsugamushi*	18	male	7	-1.5149969	0.41330791
A3927_262	13138	*O. tsutsugamushi*	35	male	7	-1.0789415	0.58250695
A3927_263	13141	*Rickettsia* sp.	53	male	7	-1.3965248	1.2138481
A3927_264	13165	*A. baumanii*	54	male	6	-1.9451865	-0.639857
A3927_265	13168	*E. coli*	21	female	3	-0.3258542	1.86913276
A3927_266	13205	*O. tsutsugamushi*	52	female	7	-0.5715867	1.88856915
A3927_267	13209	*O. tsutsugamushi*	58	female	7	-2.0228829	-0.549114
A3927_268	13210	*S. typhi*	18	male	7	-1.439958	0.134612
A3927_269	13268	*S. typhi*	30	female	8	-1.1609706	1.12376916
A3927_270	13294	*E. coli*	54	female	6	-1.2726039	0.26146321
A3927_271	13306	*E. coli*	53	female	3	0.4312623	1.7334704
A3927_272	13337	*S. oralis*	15	female	5	-1.8670762	0.86444044
A3927_273	13354	*S. aureus*	40	male	10	-3.2123484	-1.9186084
A3927_274	13398	*E. coli*	17	female	4	1.87153828	2.216926
A3927_275	13441	*O. tsutsugamushi*	30	female	6	-0.151075	2.90558887
A3927_276	13457	*O. tsutsugamushi*	25	female	6	-1.5792693	1.56722631
A3927_277	13489	*Rickettsia* sp.	33	female	8	-1.4823442	1.29393368
A3927_278	13573	*S. typhi*	32	male	14	-2.0661619	0.90438807
A3927_279	13578	*E. coli*	62	female	4	0.28045882	1.76966989
A3927_280	13599	*S. typhi*	42	female	17	-2.4143316	0.4527292
A3927_281	13655	*S. typhi*	17	male	18	-1.9979776	0.36928285
A3927_282	13697	*S. typhi*	25	male	4	-1.0771982	0.71887155
A3927_283	13698	*E. coli*	50	female	3	-11.666667	0
A3927_284	13781	*S. typhi*	25	male	7	-1.6000734	0.52132874
A3927_285	13795	*S. pneumoniae*	26	male	5	1.59725183	3.06282355
A3927_286	13803	*E. coli*	78	male	30	3.16054764	3.60446798
A3927_287	13823	*S. typhi*	27	female	30	0.67581219	2.79915108
A3927_288	13836	*Leptospira* sp.	32	female	90	-1.7390053	0.78712755

Table S11: Demographic and clinical characteristics of the subjects from the Nepal prospective validation cohort, related to Figure 5 and Figur
HHV6, Human Herpesvirus 6; CMV, Cytomegalovirus

ID	Age	Sex	Days since fever onset	Sweeney7 Score	BoVI Score	Rickettsia	HHV6	CMV	Lepto-spirosis	Parvovirus B19	Enterovirus	Typhoid/ Paratyphoi	Other Bacteremia
MD-1271	23	Male	5	4.35671117	2.3223715	NA	NA	NA	1	NA	NA	NA	NA
MD-1291	27	Male	4	0.73432697	-0.7710399	NA	NA	NA	NA	NA	NA	1	NA
MD-1305	25	Female	3	1.52998749	0.02202964	NA	NA	NA	NA	NA	NA	1	NA
MD-1307	26	Male	30	1.00187608	-0.5777194	NA	NA	NA	NA	NA	NA	1	NA
MD-1311	18	Male	3	2.42445032	0.98989613	NA	NA	NA	NA	NA	NA	1	NA
MD-1316	25	Male	13	2.08013424	0.50908894	NA	1	NA	NA	NA	NA	NA	NA
MD-1318	20	Female	4	1.0635897	-0.6678065	NA	NA	NA	NA	NA	NA	1	NA
MD-1342	35	Female	4	1.66562992	0.440424	1	NA	NA	NA	NA	NA	NA	NA
MD-1343	19	Male	3	1.30865015	0.15870642	NA	NA	NA	NA	NA	NA	1	NA
MD-1368	31	Female	3	1.58971076	0.50186351	NA	NA	NA	NA	NA	NA	1	NA
MD-1380	55	Female	6	2.34527005	0.89877188	1	NA	NA	NA	NA	NA	NA	NA
MD-1389	15	Female	9	0.5619972	0.18905936	NA	NA	NA	NA	NA	NA	NA	1
MD-1398	20	Female	5	0.88372359	0.28834722	1	NA	NA	NA	NA	NA	NA	NA
MD-1400	36	Male	7	1.20414882	0.03104493	NA	NA	NA	NA	NA	NA	1	NA
MD-1420	31	Male	5	5.00541109	2.05086348	NA	NA	NA	NA	NA	NA	NA	1
MD-1421	65	Female	3	1.74478929	-0.264004	NA	NA	NA	NA	NA	NA	NA	1
MD-1430	19	Male	3	0.54188317	-0.3656634	NA	NA	NA	NA	NA	NA	1	NA
MD-1436	85	Female	4	2.22611925	-0.3602213	NA	NA	NA	NA	NA	NA	NA	1
MD-1447	47	Female	3	2.68513843	1.42418338	NA	NA	NA	1	NA	NA	NA	NA
MD-1459	54	Male	4	1.84798906	0.30013943	1	NA	NA	NA	NA	NA	NA	NA
MD-1461	19	Female	5	0.87076873	0.68479991	1	NA	NA	NA	NA	NA	NA	NA
MD-1498	23	Female	3	2.01938907	0.51344586	NA	NA	NA	NA	NA	NA	1	NA
MD-1501	23	Male	3	-0.0325583	-2.9283768	NA	1	NA	NA	NA	NA	NA	NA
MD-1517	56	Male	10	1.92912896	2.23939672	1	NA	NA	NA	NA	NA	NA	NA
MD-1522	19	Female	4	2.30401256	1.153923	NA	NA	NA	NA	NA	NA	1	NA
MD-1525	72	Male	10	1.40643515	1.11042719	1	NA	NA	NA	NA	NA	NA	NA
MD-1526	27	Male	7	1.39362338	0.12945331	NA	NA	NA	NA	NA	NA	1	NA
MD-1568	55	Female	4	2.49605219	1.31565851	NA	NA	NA	1	NA	NA	NA	NA
MD-1572	28	Female	6	3.76007714	0.35243434	NA	1	NA	NA	NA	NA	NA	NA
MD-1577	22	Female	6	1.42916007	0.76210368	1	NA	NA	NA	NA	NA	NA	NA
MD-1580	38	Female	10	1.05589323	1.50155603	1	NA	NA	NA	NA	NA	NA	NA
MD-1582	27	Male	4	1.02536166	-0.0660992	NA	NA	NA	NA	NA	NA	1	NA
MD-1590	18	Female	4	-0.0328625	-0.7002131	1	NA	NA	NA	NA	NA	NA	NA
MD-1595	16	Male	7	1.37035101	-0.9873942	NA	1	NA	NA	NA	NA	NA	NA

MD-1598	18	Female	8	0.94056078	0.6567604	1	NA	NA	NA	NA	NA	NA	NA
MD-1599	36	Female	3	4.50367837	1.78411295	NA	NA	NA	1	NA	NA	NA	NA
MD-1602	40	Male	12	2.12709135	1.69244181	1	NA	NA	NA	NA	NA	NA	NA
MD-1632	19	Female	30	2.4378046	1.67126446	1	NA	NA	NA	NA	NA	NA	NA
MD-1664	23	Female	21	0.34510238	-0.3446884	1	NA	NA	NA	NA	NA	NA	NA
MD-1706	16	Female	14	1.2404258	0.13557942	1	NA	NA	NA	NA	NA	NA	NA
MD-1707	24	Male	6	1.67443513	1.53289473	1	NA	NA	NA	NA	NA	NA	NA
MD-1726	23	Male	7	1.58417347	-0.1799893	NA	NA	NA	NA	NA	NA	1	NA
MD-1749	55	Female	7	0.38462493	-0.2736644	1	NA	NA	NA	NA	NA	NA	NA
MD-1776	25	Female	3	3.75764928	2.10848077	NA	NA	NA	NA	NA	NA	NA	1
MD-1778	26	Female	4	-0.2023802	-0.2051605	NA	1	NA	NA	NA	NA	NA	NA
MD-1823	54	Female	5	-0.7735589	-2.0301234	NA	1	NA	NA	NA	NA	NA	NA
MD-1868	60	Male	20	3.44449292	2.58501982	1	NA	NA	NA	NA	NA	NA	NA
MD-1917	49	Female	10	0.58729605	0.94206906	1	NA	NA	NA	NA	NA	NA	NA
MD-1921	19	Female	4	1.18060535	0.65060506	NA	NA	NA	NA	NA	NA	1	NA
MD-1923	27	Female	5	0.93376907	0.44376873	NA	NA	NA	NA	NA	NA	1	NA
MD-1989	20	Female	3	2.07521157	0.92470646	NA	NA	NA	1	NA	NA	NA	NA
MD-2017	45	Male	6	0.83799984	-0.362129	1	NA	NA	NA	NA	NA	NA	NA
MD-2041	35	Male	5	1.21211683	0.46209027	NA	NA	NA	NA	NA	NA	1	NA
MD-2047	24	Male	3	2.93995688	0.44849113	NA	NA	NA	NA	NA	NA	NA	1
MD-2121	37	Male	30	7.76582532	3.89314657	NA	NA	NA	NA	NA	NA	NA	1
MD-2137	60	Female	20	0.44018579	-0.8305989	NA	1	NA	NA	NA	NA	NA	NA
MD-2143	16	Male	7	2.16213705	1.4993639	NA	NA	NA	NA	NA	NA	1	NA
MD-2145	19	Male	7	2.26570034	0.67953089	NA	NA	NA	NA	NA	NA	1	NA
MD-2146	19	Female	5	1.79734891	1.23153263	NA	NA	NA	NA	NA	NA	1	NA
MD-2176	26	Female	7	1.72699161	1.32709247	1	NA	NA	NA	NA	NA	NA	NA
MD-2228	20	Male	3	1.91414899	0.39995173	NA	NA	NA	NA	NA	NA	1	NA
MD-2255	19	Male	4	1.20404435	0.53145327	NA	NA	NA	NA	NA	NA	1	NA
MD-2260	21	Male	4	1.49500072	0.31078718	NA	NA	NA	NA	NA	NA	1	NA
MD-2278	18	Male	15	1.23282067	0.20881076	NA	NA	NA	NA	NA	NA	1	NA
MD-2300	18	Male	3	1.93279754	0.70837782	NA	NA	NA	NA	NA	NA	1	NA
MD-2329	38	Male	4	0.85112483	-0.2129888	NA	1	NA	NA	NA	NA	NA	NA
MD-2351	16	Male	6	0.07368202	-1.3396312	1	NA	NA	NA	NA	NA	NA	NA
MD-2357	24	Female	3	-1.7243634	-4.0165012	NA	NA	NA	NA	NA	NA	NA	1
MD-2370	54	Male	14	5.04274998	3.16628307	NA	NA	NA	NA	NA	NA	NA	1
MD-2411	43	Male	4	2.62780069	0.17091187	NA	NA	NA	NA	NA	NA	NA	1
MD-2443	24	Male	4	2.23109872	0.00340875	NA	NA	NA	NA	NA	NA	1	NA
MD-2459	18	Female	3	2.50289207	1.29214793	NA	NA	NA	NA	NA	NA	1	NA
MD-2460	19	Female	3	2.73541704	1.95672423	NA	NA	NA	NA	NA	NA	1	NA

MD-2520	49	Female	7	5.80562441	4.44124376	NA	NA	NA	NA	NA	NA	NA	1
MD-2543	17	Male	7	1.67827799	1.15773326	NA	NA	NA	NA	NA	NA	1	NA
MD-2547	20	Male	4	1.16470352	0.7258623	NA	NA	NA	NA	NA	NA	1	NA
MD-2555	40	Female	3	5.03905619	3.10703922	NA	NA	NA	NA	NA	NA	NA	1
MD-2571	41	Male	4	1.07023112	1.09766139	NA	NA	NA	NA	NA	NA	1	NA
MD-2611	27	Female	7	0.81632679	-1.8816253	NA	1	NA	NA	NA	NA	NA	NA
MD-2639	35	Female	5	1.43169438	0.17998754	1	NA	NA	NA	NA	NA	NA	NA
MD-2665	45	Female	4	1.68675982	0.44044194	NA	NA	NA	NA	NA	NA	1	NA
MD-2668	50	Female	3	5.1932359	3.21385732	NA	NA	NA	NA	NA	NA	NA	1
MD-2684	70	Female	5	6.01072102	3.01442941	NA	NA	NA	NA	NA	NA	NA	1
MD-2689	41	Female	3	1.85510265	1.59876459	1	NA	NA	NA	NA	NA	NA	NA
MD-2697	17	Female	4	1.62488167	0.79288649	NA	NA	NA	NA	NA	NA	1	NA
MD-2723	60	Male	8	1.10866383	0.78099576	1	NA	NA	NA	NA	NA	NA	NA
PD-423	9	Male	10	1.07687789	0.20797101	NA	NA	NA	NA	NA	NA	NA	1
PD-473	13	Male	5	1.62335746	0.87489941	1	NA	NA	NA	NA	NA	NA	NA
PD-474	11	Female	6	0.93176607	-0.5208058	1	NA	NA	NA	NA	NA	NA	NA
PD-490	13	Female	9	1.44773646	-0.1022349	1	NA	NA	NA	NA	NA	NA	NA
PD-560	5	Male	3	2.77162574	0.26226301	NA	1	NA	NA	NA	NA	NA	NA
PD-592	10	Female	13	1.78033118	-0.3250347	NA	NA	1	NA	NA	NA	NA	NA
PD-619	2	Male	8	-0.6428226	-1.8703919	NA	NA	1	NA	NA	NA	NA	NA
PD-634	5	Male	3	-1.6908361	-2.7067134	NA	1	NA	NA	NA	NA	NA	NA
PD-642	7	Male	6	-2.6197061	-3.3044409	NA	1	NA	NA	NA	NA	NA	NA
PD-653	15	Female	3	-0.6218849	-2.705731	NA	NA	NA	NA	NA	NA	1	NA
PD-676	15	Male	3	0.92313135	0.78402417	NA	NA	NA	NA	NA	NA	1	NA
PD-702	5	Female	7	-1.1616615	-2.0989911	NA	NA	NA	NA	NA	1	NA	NA
PD-709	6	Female	10	1.3972632	-0.8962867	NA	1	NA	NA	NA	NA	NA	NA
PD-714	2	Female	5	-0.8689453	-1.1010541	NA	NA	1	NA	NA	NA	NA	NA
PD-723	14	Female	5	0.96163498	0.96361867	NA	NA	NA	NA	NA	NA	1	NA
PD-743	4	Male	3	-0.2185531	-2.5971428	NA	NA	NA	NA	NA	1	NA	NA
PD-746	11	Male	4	1.27318504	0.52120528	NA	NA	NA	NA	NA	NA	1	NA
PD-748	9	Male	3	1.1562908	-1.1388686	NA	NA	NA	NA	NA	1	NA	NA
PD-750	6	Female	4	-1.6687056	-3.5335519	NA	NA	NA	NA	NA	1	NA	NA
PD-753	6	Male	7	0.59552809	-0.4418712	NA	1	NA	NA	NA	NA	NA	NA
PD-755	2	Male	6	-1.1908377	-2.5323262	NA	1	NA	NA	NA	NA	NA	NA
PD-762	4	Male	6	-0.5776425	-1.1508679	NA	NA	NA	NA	NA	1	NA	NA
PD-783	4	Male	4	-1.8371084	-4.0605512	NA	NA	NA	NA	NA	1	NA	NA
PD-799	5	Male	4	0.48332088	-1.2126503	NA	1	NA	NA	NA	NA	NA	NA
PD-863	11	Female	4	0.72803281	-0.1961533	NA	NA	NA	NA	NA	NA	1	NA

Table S12: TaqMan assays used in Fluidigm validation, related to Figure 5 and Figure S11.
Assays were normalized using the geometric mean of three housekeeping genes: 18S, ACTB, and KPNA6.

Gene Symbol	Gene Name	Alias	RefSeq	Thermofisher ID	fullname	Amplicon Length	Best Coverage	3' Most	Signature	in Laos validation set	in Nepal validation set
18S	Eukaryotic 18S rRNA			Hs99999901_s1	18S_Hs99999901_s1	187	No	No	control	y	y
ACTB	actin beta	BRWS1; PS1TP5BP1	NM_001101.3	Hs01060665_g1	ACTB_Hs01060665_g1	63	Yes	No	control	y	y
FARP1	FERM; ARH/RhoGEF and pleckstrin domain protein 1	CDEP; FARP1-IT1; PLEKHC2; PPP1R75	NM_005766.3;XM_011521046.2;XM_017020312.1;XM_017020313.1;NM_001286839.1	Hs00195010_m1	FARP1_Hs00195010_m1	65	Yes	No	control	y	n
FPGS	folylpolyglutamate synthase		XM_005251864.3;NM_001018078.2;NR_110170.1;XM_011518439.2;NM_001288803.1;NM_004957.5;XM_017014565.1;XM_017014566.1	Hs00191956_m1	FPGS_Hs00191956_m1	116	Yes	Yes	control	y	n
GAPDH	glyceraldehyde-3-phosphate dehydrogenase	G3PD; GAPD; HEL-S-162eP	NM_002046.5;NM_001256799.2;NM_001289745.1;NM_001289746.1	Hs02786624_g1	GAPDH_Hs02786624_g1	157	Yes	No	control	y	n
KPNA6	karyopherin subunit alpha 6	IPOA7; KPNA7	NM_012316.4;XM_005270711.3	Hs00202389_m1	KPNA6_Hs00202389_m1	76	Yes	No	control	y	y
PEX16	peroxisomal biogenesis factor 16	PBD8A; PBD8B	NM_057174.2;XM_011520474.1;NM_004813.2	Hs00191337_m1	PEX16_Hs00191337_m1	75	Yes	No	control	y	n
RPS4Y1	ribosomal protein S4; Y-linked 1	RPS4Y; S4	NM_001008.3;XM_017030061.1	Hs00606158_m1	RPS4Y1_Hs00606158_m1	105	Yes	Yes	control (distinguishes male/female)	y	n
EBI3	Epstein-Barr virus induced 3	IL-27B; IL27B	NM_005755.2;XM_011527619.2	Hs01057148_m1	EBI3_Hs01057148_m1	64	Yes	No	BoVI signature	y	y
FCER1A	Fc fragment of IgE receptor Ia	FCE1A; FcERI	NM_002001.3	Hs00758600_m1	FCER1A_Hs00758600_m1	105	Yes	Yes	BoVI signature	y	y
HESX1	HESX homeobox 1	ANF; CPHD5; RPX	XM_011534205.2;NM_003865.2;XM_005265526.4;XM_017007421.1;XM_011534204.2;XM_006713379.3	Hs00172696_m1	HESX1_Hs00172696_m1	110	Yes	No	BoVI signature	y	y

ICAM1	intercellular adhesion molecule 1	BB2; CD54; P3.58	NM_000201.2	Hs00164932_m1	ICAM1_Hs00164932_m1	87	Yes	No	BoVI signature	y	y
IFI27	interferon alpha inducible protein 27	FAM14D; ISG12; ISG12A; P27	NM_005532.4;NM_001130080.2	Hs01086370_m1	IFI27_Hs01086370_m1	71	No	No	BoVI signature*	y	y
IFI27	interferon alpha inducible protein 27	FAM14D; ISG12; ISG12A; P27	NM_001288952.1;NM_001288954.1;NM_005532.4;NM_001130080.2;NM_001288995.1;NM_001288956.1;NM_001288958.1;NM_001288957.1	Hs00271467_m1	IFI27_Hs00271467_m1	63	No	No	BoVI signature*	y	y
IFI27	interferon alpha inducible protein 27	FAM14D; ISG12; ISG12A; P27	NM_005532.4;NM_001288960.1;NM_001288959.1;NM_001288956.1;NM_001288958.1;NM_001288957.1;NM_001288952.1;NM_001288954.1;NM_001130080.2;NM_001288995.1	Hs01086373_g1	IFI27_Hs01086373_g1	68	Yes	Yes	BoVI signature*	y	y
JUP	junction plakoglobin	ARVD12; CTNNG; DP3; DPIII; PDGB; PKGB	NM_021991.2;XM_017024588.1;XM_011524753.2;XM_017024589.1;XM_017024590.1;XM_011524755.1;XM_011524756.1;XM_011524757.2;XM_006721878.1;XM_011524758.1;NM_002230.2;XM_006721875.1;XM_006721874.2;XM_006721873.2	Hs00158408_m1	JUP_Hs00158408_m1	66	Yes	No	BoVI signature	y	y
SMARCD3	SWI/SNF related; matrix associated; actin dependent regulator of chromatin; subfamily d; member 3	BAF60C; CRACD3; Rsc6p	NM_001003801.1;XM_017012556.1;XM_011516521.2;NM_003078.3;NM_001003802.1	Hs00162003_m1	SMARCD3_Hs00162003_m1	109	Yes	No	BoVI signature	y	y
SUCLG2	succinate-CoA ligase GDP-forming beta subunit	GBETA	XM_017007417.1;XM_017007416.1;XM_017007419.1;XM_017007418.1;XM_017007420.1;NM_001177599.1;NM_003848.3;XM_017007415.1	Hs00896917_g1	SUCLG2_Hs00896917_g1	107	Yes	Yes	BoVI signature	y	y

* averaged Ct for all IFI27 assays; similar results obtained using each individual assay (see Methods)
