# Supplementary material for: A robust host-response-based signature distinguishes bacterial and viral infections across diverse global populations
Source: Cell Rep Med. 2022 Dec 20;3(12):100842. doi: 10.1016/j.xcrm.2022.100842 (PMC9797950; doi:10.1016/j.xcrm.2022.100842)
Supplement: Document S2. Article plus supplemental information [file mmc2.pdf]

Cell Reports Medicine

Article

A robust host-response-based signature distinguishes bacterial and viral infections across diverse global populations

Graphical abstract

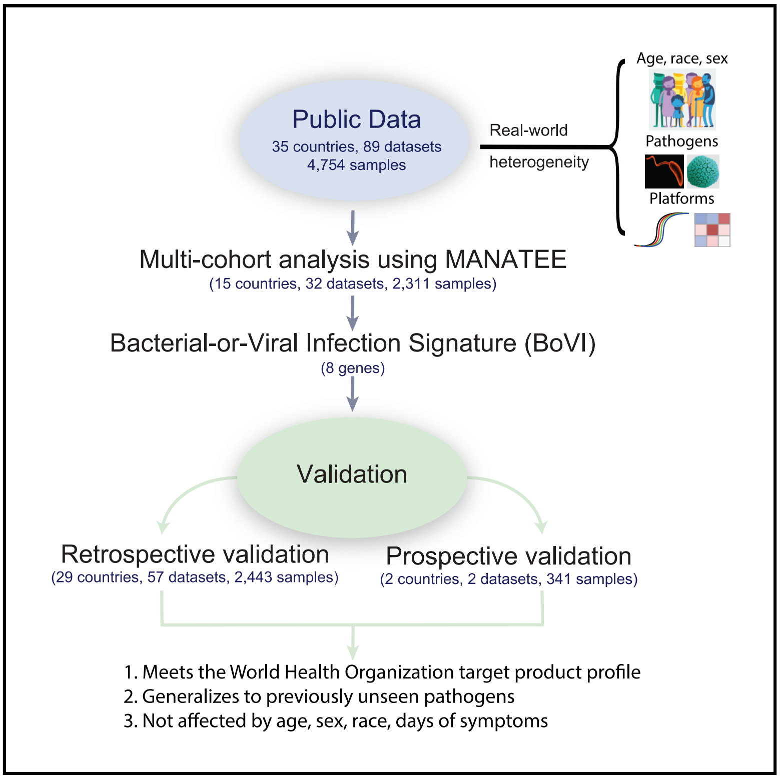

Highlights

- Intracellular and extracellular bacterial infection prevalence differ across countries
- Host-response-based signatures have low accuracy for intracellular bacterial infections
- 8-gene signature distinguishes bacterial versus viral infection despite broad heterogeneity
- 8 genes diagnose intra- and extracellular bacterial infection with similar accuracy

Authors

Aditya M. Rao, Stephen J. Popper, Sanjana Gupta, ..., Jason R. Andrews, David A. Relman, Purvesh Khatri

Correspondence

pkhatri@stanford.edu

In brief

Current host-response-based diagnostics have lower accuracy for distinguishing intracellular bacterial infections from viral infections. Rao et al. identified an 8-gene signature to address this challenge. Using multiple validation cohorts, they show that the 8-gene signature generalizes to global patient population with similar accuracy for extra- and intracellular bacterial infections.

Rao et al., 2022, Cell Reports Medicine *3*, 100842
December 20, 2022 © 2022 The Authors.
https://doi.org/10.1016/j.xcrm.2022.100842

CellPress

Cell Reports Medicine

CellPress
OPEN ACCESS

Article

A robust host-response-based signature distinguishes bacterial and viral infections across diverse global populations

Aditya M. Rao,[1,2,14] Stephen J. Popper,[3,14] Sanjana Gupta,[1,4] Viengmon Davong,[5] Krista Vaidya,[6] Anisone Chanthongthip,[5] Sabine Dittrich,[5,7] Matthew T. Robinson,[5,7] Manivanh Vongsouvath,[5] Mayfong Mayxay,[5,7,8] Pruksa Nawtaisong,[5] Biraj Karmacharya,[6] Simone A. Thair,[1,4] Isaac Bogoch,[10] Timothy E. Sweeney,[9] Paul N. Newton,[5,7] Jason R. Andrews,[3] David A. Relman,[1,3,11,12,15] and Purvesh Khatri[1,4,13,15,16,*]

[1]Institute for Immunity, Transplantation, and Infection, Stanford University School of Medicine, 240 Pasteur Dr., Biomedical Innovation Building, Room 1553, Stanford, CA, USA
[2]Immunology Graduate Program, Department of Medicine, Stanford University, Stanford, CA, USA
[3]Division of Infectious Diseases and Geographic Medicine, Department of Medicine, Stanford University, Stanford, CA, USA
[4]Center for Biomedical Informatics Research, Department of Medicine, Stanford University, Stanford, CA, USA
[5]Lao-Oxford-Mahosot Hospital-Wellcome Trust Research Unit, Microbiology Laboratory, Mahosot Hospital, Vientiane, Lao PDR
[6]Dhulikhel Hospital, Kathmandu University Hospital, Kavrepalanchok, Nepal
[7]Centre for Tropical Medicine and Global Health, Nuffield Department of Medicine, University of Oxford, Oxford, UK
[8]Institute of Research and Education Development (IRED), University of Health Sciences, Ministry of Health, Vientiane, Lao PDR
[9]Inflammatix Inc., Sunnyvale, CA, USA
[10]Department of Medicine, University of Toronto, Toronto, ON, Canada
[11]Department of Microbiology and Immunology, Stanford University, Stanford, CA, USA
[12]Infectious Diseases Section, Veterans Affairs Palo Alto Health Care System, Palo Alto, CA, USA
[13]Present address: Inflammatix Inc., Sunnyvale, CA, USA
[14]These authors contributed equally
[15]These authors contributed equally
[16]Lead contact
*Correspondence: pkhatri@stanford.edu
https://doi.org/10.1016/j.xcrm.2022.100842

SUMMARY

Limited sensitivity and specificity of current diagnostics lead to the erroneous prescription of antibiotics. Host-response-based diagnostics could address these challenges. However, using 4,200 samples across 69 blood transcriptome datasets from 20 countries from patients with bacterial or viral infections representing a broad spectrum of biological, clinical, and technical heterogeneity, we show current host-response-based gene signatures have lower accuracy to distinguish intracellular bacterial infections from viral infections than extracellular bacterial infections. Using these 69 datasets, we identify an 8-gene signature to distinguish intracellular or extracellular bacterial infections from viral infections with an area under the receiver operating characteristic curve (AUROC) > 0.91 (85.9% specificity and 90.2% sensitivity). In prospective cohorts from Nepal and Laos, the 8-gene classifier distinguished bacterial infections from viral infections with an AUROC of 0.94 (87.9% specificity and 91% sensitivity). The 8-gene signature meets the target product profile proposed by the World Health Organization and others for distinguishing bacterial and viral infections.

INTRODUCTION

Early and accurate diagnosis of acute infection has important consequences for clinical outcome, antibiotic stewardship, and allocation of health care resources. However, the majority of patients with acute infection never receive a positive microbiological diagnosis.[1,2] Acute systemic infections typically present with non-specific signs and symptoms, and yet decisions about clinical management and treatment are often made on the basis of clinical presentation, local epidemiology, and local clinical standards of care, leading to the erroneous prescription of empiric antibiotics in 30%–75% of patients with viral infections in the US, Canada, or the UK.[3,4] This number is even higher in low- and middle-income countries (LMICs), with up to 95% of patients with non-bacterial infections inappropriately prescribed antibiotics.[5,6] With resistance to antimicrobial treatment rising, the health and economic burden of these misdiagnoses is, and will continue to be, very large.[7,8] There is a critical unmet need to develop diagnostics that can safely rule out bacterial infection.

To address this challenge the World Health Organization (WHO) and the Foundation for Innovative New Diagnostics (FIND), together with several others, have proposed diagnostic performance requirements of >90% sensitivity and >80% specificity for distinguishing bacterial from non-bacterial infections.[9] None

This is an open access article under the CC BY license (http://creativecommons.org/licenses/by/4.0/).

of the current pathogen-detecting diagnostic solutions meet this target product profile (TPP). Despite the recent popularity of metagenomic sequencing, the sensitivity and interpretability of this approach remain in question.[10,11] In fact, there have been calls for establishing next-generation sequencing (NGS) stewardship boards to restrict its use in intensive care units (ICUs) to only patients who truly need it.[12] In contrast, a host-response-based diagnostic test for infectious diseases can be sensitive, more easily interpreted, and cheaper with faster turnaround time (TAT). However, host-response-based single-biomarker tests such as C-reactive protein (CRP), pro-calcitonin (PCT), and lactate are often non-specific for the type of infection and can be elevated in the inflammatory response to non-infection settings such as trauma.[9,10] The information gained from these tests has limited clinical utility, as negative results have poor predictive value and often are not sufficient to rule out infection.[13]

Recently, several studies have demonstrated the ability of host-response-based gene signatures to diagnose the presence and type of infection even when the pathogen itself is not detected.[14–16] However, these signatures were identified and validated exclusively in populations from Western Europe and North America, whereas the greatest burden of systemic infection exists elsewhere.[17,18] In fact, a recent study suggested that the vast majority of sepsis cases occur in countries with low socio-demographic indices.[19] Further, the epidemiology of febrile illness in these countries includes a distinct group of bacterial and viral pathogens. For instance, important infectious causes of fever in LMICs, particularly in Asia, are either not endemic or more rarely acquired in Europe or North America, including typhoid, dengue, and scrub typhus (*Orientia tsutsugamushi*).[20–22] In addition, bacterial pathogens that are common in LMICs are often obligate or facultative intracellular pathogens that, similar to viruses, generate an interferon-driven host response that leads to different modulations of host cell metabolism and gene expression than what is observed in response to extracellular bacterial infections.[23,24] Note that this dichotomous classification of bacterial pathogens as intracellular or extracellular pathogens is simplistic because certain pathogens (e.g., *Staphylococcus aureus*) can occupy both niches. The qualifier terms, intracellular and extracellular, are sometimes used to indicate bacterial infections that are differentially enriched in studies from LMIC and non-LIMC countries, respectively.

These differences in both demographics and causative infectious agents have underscored the importance of accounting for real-world biological, clinical, and technical heterogeneity in developing diagnostics generalizable to global populations. For example, a host-response-based signature for tuberculosis (TB) identified using samples from one country failed to replicate in samples from other countries on the same continent.[25] On the other hand, a host-response-based diagnostic signature for TB that was discovered using multiple independent cohorts across several countries has been independently validated in several follow-up prospective studies and translated into a point-of-care test,[26,27] and has also been validated in additional prospective cohorts.[28] Similar results have also been observed in host-response-based diagnostic signatures for sepsis where an 11-gene signature derived using multiple independent cohorts has outperformed those derived using a single cohort.[29,30]

Here, we hypothesized that recently described host-response-based mRNA signatures for distinguishing bacterial and viral infections do not account for biological heterogeneity in the immune response to intracellular bacterial infections, and therefore will not distinguish intracellular infections from viral infections with clinically useful accuracy. Our comparison of 4 host-response-based gene signatures, ranging from 2 to 120 genes, demonstrated, as hypothesized, their lower accuracy in distinguishing intracellular bacterial infections from viral infections. Next, we developed a multi-cohort analysis framework to enable integration of a large number of independent heterogeneous datasets with bacterial infections (extracellular or intracellular) and viral infections, and used it to derive an 8-gene host-response-based signature that is able to distinguish extracellular and intracellular bacterial infections from viral infections with comparable accuracy. Finally, we validated the 8-gene signature and observed increased accuracy in multiple independent retrospective datasets and two prospective cohorts from Nepal and the Lao People's Democratic Republic (Laos).

RESULTS

Current host-response-based gene signatures have lower accuracy for distinguishing intracellular bacterial infections from viral infections

We hypothesized that a host-response-based gene signature identified using only blood samples from patients with viral or extracellular bacterial infections would have lower accuracy in distinguishing intracellular bacterial infections from viral infections. To test this hypothesis, we performed a search of public data repositories to identify blood transcriptome profiles from febrile patients with extracellular bacterial, intracellular bacterial, or viral infections.[31,32] We identified 64 independent datasets including 55 from whole-blood (WB) samples and nine from peripheral blood mononuclear cell (PBMC) samples that met our inclusion and exclusion criteria (STAR Methods; Tables S1 and S2).[14–16,23,24,30,33–73] These 64 datasets included transcriptome profiles of 3,708 blood samples from 1,186 healthy controls and 2,522 patients with a microbiologically confirmed infection (728 extracellular bacterial infections, 301 intracellular bacterial infections, 191 infections with an unspecified bacterial pathogen, 1,302 viral infections) collected from 20 countries across six continents, with similar representation of both sexes and a wide range of ages (Table S3). These samples also include a wide range of extracellular bacteria (e.g., *S. aureus*, *Escherichia coli*, *Streptococcus pneumoniae*, *Leptospira* spp., *Streptococcus pyogenes*, *Klebsiella pneumoniae*), intracellular bacteria (e.g., *Burkholderia pseudomallei*, *Salmonella enterica* Typhi, *Rickettsia typhi*, *Orientia tsutsugamushi*, *Brucella* spp.), and viruses (e.g., influenza, dengue, cytomegalovirus [CMV], parainfluenza, adenovirus, human rhinovirus, respiratory syncytial virus) (Table S4). Finally, the patients were enrolled in diverse clinical settings, including outpatient clinics, emergency departments, general wards, and intensive care units (Tables S1 and S2).

After co-normalizing the 64 datasets using Combat Co-normalization Using Controls (COCONUT) (STAR Methods), we compared four previously described host-response-based

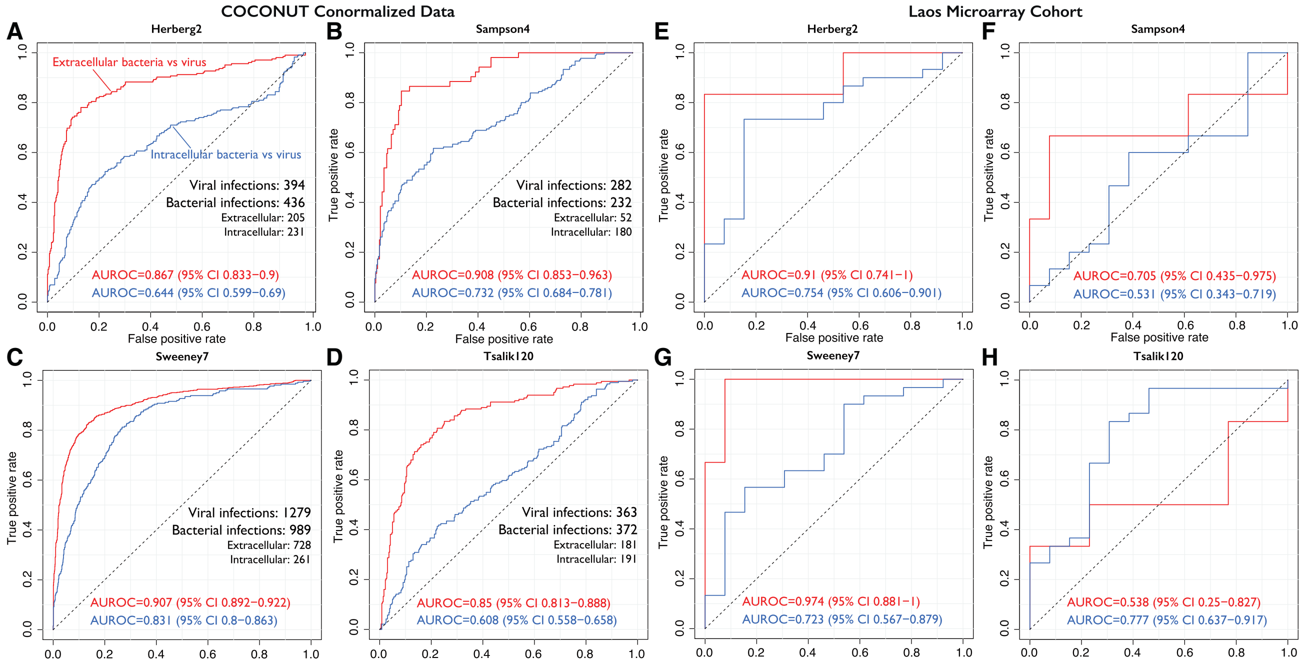

Figure 1. Existing signatures derived by comparing only extracellular bacterial infections and viral infections have lower accuracy in distinguishing intracellular bacterial infections from viral infections

ROC curves comparing the performance of 4 published signatures at separating extracellular bacterial and viral infections versus separating intracellular bacterial and viral infections.

(A–D) Receiver operating characteristic (ROC) curves from testing the signatures in up to 64 COCONUT co-normalized datasets.

(E–H) ROC curves from testing the signatures in the Laos Microarray cohort. See also Figures S1 and S2, and Tables S1–S5 and S7.

gene signatures for distinguishing bacterial and viral infections.[74] The number of genes in these signatures ranged from two to 120, and they were each derived using between 240 and 599 samples. The Sampson4[75] and Sweeney7[74] signatures were derived using multiple independent cohorts, whereas the Herberg2[14] and Tsalik120[76] signatures were each derived from a single cohort. Importantly, none of these signatures were derived using samples from patients with intracellular bacterial infections.

Each of the four signatures had high accuracy in distinguishing extracellular bacterial infections from viral infections in the 64 co-normalized datasets, with the AUROC ranging from 0.85 for Tsalik120 to 0.91 for Sampson4 and Sweeney7 (Figures 1A–1D). However, each signature had significantly lower accuracy in distinguishing intracellular bacterial from viral infections, with AUROCs ranging from 0.61 for Tsalik120 to 0.83 for Sweeney7 (Figures 1A–1D). The difference in AUROCs for distinguishing extracellular or intracellular bacterial infections from viral infections ranged from 7.6% (Sweeney7) to 24.2% (Tsalik120) (Figures 1A–1D). To ensure that the higher accuracy for extracellular bacterial infection was not an artifact of COCONUT co-normalization, we also profiled each of the four signatures in an independent cohort of 61 WB samples from Laos, including healthy controls (n = 12) and patients with extracellular bacterial (n = 6), intracellular bacterial (n = 30), and viral infections (n = 13) using a custom cDNA array (Tables S1 and S5). In the Laos Microarray cohort, three of the four signatures had higher accuracy for extracellular bacterial infections than intracellular bacterial infections (difference in AUROC >15%), and no signature demonstrated clinically useful accuracy in distinguishing intracellular bacterial from viral infections, with AUROCs ranging from 0.531 to 0.777 (Figures 1E–1H).

Arguably, these estimates may be confounded as we could not use all datasets to evaluate every signature, since not all genes for each of the four signatures were measured across every dataset. Therefore, we performed an extensive random sampling of our datasets and analyzed each signature's performance across 1,396,677 unique combinations (STAR Methods). Irrespective of the number of samples and datasets used, each signature had consistently higher accuracy for extracellular bacterial infections. The difference between the AUROC for distinguishing extracellular bacterial infections and that for distinguishing intracellular bacterial infections ranged from approximately 10% for Sweeney7 to just under 20% for Herberg2 (Figure S1). Interestingly, the difference in AUROCs had higher variability for the Sampson4 and Tsalik120 signatures than the Herberg2 and Sweeney7 signatures. One explanation for this is that some datasets did not measure all of the genes in Sampson4 and Tsalik120. However, we found that, even when fewer genes were missing, the Tsalik120 signature had higher AUROC for distinguishing extracellular bacterial infections from viral infections than for distinguishing intracellular bacterial infections from viral infections (Figure S2).

Collectively, these results strongly supported our hypothesis that a host-response-based gene signature derived using only samples from patients with extracellular bacterial or viral

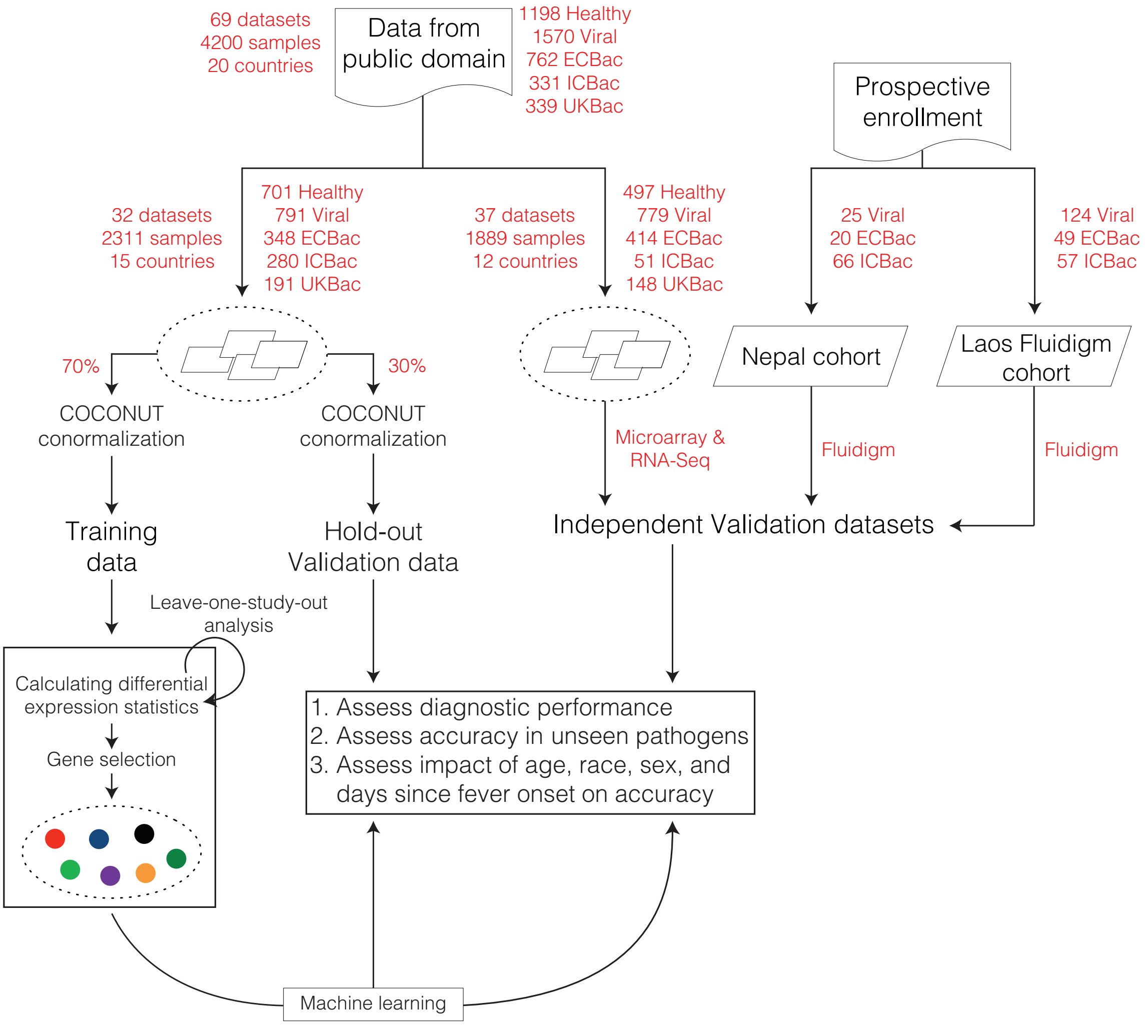

Figure 2. MANATEE framework diagram

Schematic of the multi-cohort analysis work flow that was used for training and validation of the BoVI signature. The Laos Microarray cohort was combined with 31 datasets from the public domain, for a total of 32 datasets used in signature discovery. ECBac, extracellular bacterial infection; ICBac, intracellular bacterial infection; UKB, unknown or unspecified bacterial infection. See also Figure S3 and Tables S3 and S4.

infections will have lower discriminatory power for distinguishing intracellular bacterial infections from viral infections, resulting in severely restricted generalizability in the global fight against antimicrobial resistance. They were also in concordance with previous observations that signatures developed by leveraging heterogeneity across multiple datasets are more robust than those developed using single cohorts.[25,29,77,78] Importantly, these results demonstrated that virtually none of the host-response-based diagnostic signatures described to date are generalizable to LMICs where the incidence of intracellular bacterial infections is as high or higher than extracellular bacterial infections.

Deriving an 8-gene signature for distinguishing bacterial and viral infections with MANATEE

We set out to identify a globally generalizable host-response-based gene signature for distinguishing viral infections from either extracellular or intracellular bacterial infections with comparable accuracy. However, despite the large amount of blood transcriptome data available from patients with either extracellular bacterial, intracellular bacterial, or viral infections, only 3 datasets included transcriptome profiles from all 3 classes of infections. Therefore, we devised a machine learning framework, referred to as Multi-cohort Analysis of Aggregated Gene Expression (MANATEE), to aggregate and analyze data from a large number of independent studies while reducing the risk of overfitting (Figure 2 and STAR Methods).

Prior to applying MANATEE, we aggregated the 64 datasets with the Laos Microarray dataset and 5 additional publicly available datasets (3 WB and 2 PBMC) composed of 431 samples.[14,73,76] These five datasets did not include healthy subjects and thus could not be co-normalized using COCONUT, but, because each dataset included both patients with bacterial and viral infections, they could still be used to validate any

CellPress
OPEN ACCESS

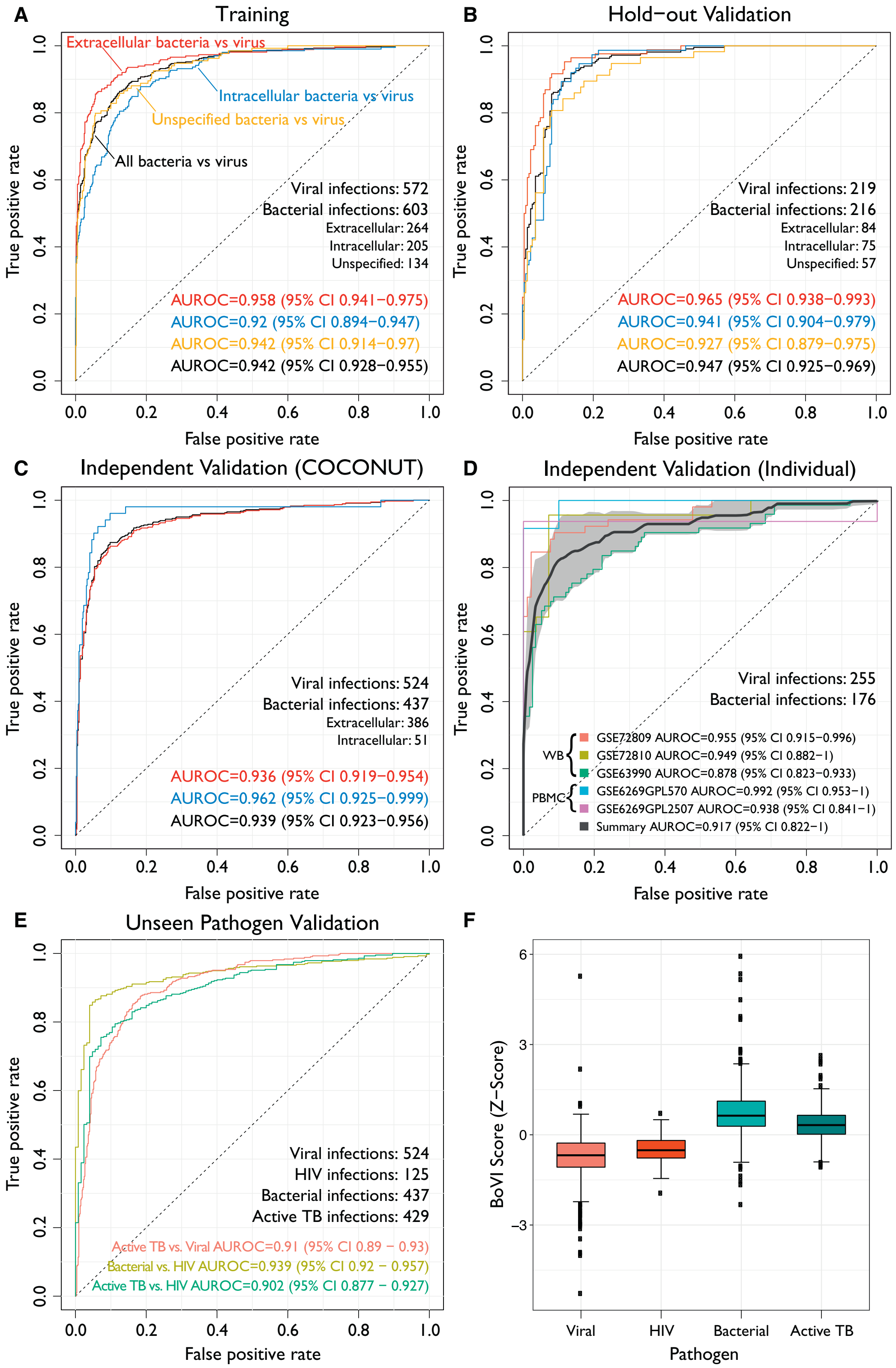

(legend on next page)

signatures we discovered. Overall, we identified 69 datasets composed of 4,200 samples (Figure 2). We divided these 69 datasets into 3 groups: training, hold-out validation, and independent validation (Tables S1–S3 and S6). In order to ensure that no samples from the datasets used in independent validation were included in either training or hold-out validation, we chose 32 WB datasets (2,311 samples) and *a priori* randomly divided them into the training set (70%) and the hold-out validation set (30%). We used the remaining 37 WB or PBMC datasets (1,889 samples) as an independent validation set to ensure the generalizability of a signature derived using the training and hold-out validation sets.

We selected three sets of genes using stringent selection criteria in the training set: (1) 417 genes with FDR $\leq$ 1% and absolute effect size (|ES|) $\geq$ 0.6, (2) 76 genes with FDR $\leq$ 1% and |ES| $\geq$ 0.8, and (3) 100 genes with the highest scores from a significance analysis of microarrays (SAM) analysis (Table S7). We further applied different strategies to identify a parsimonious set of genes used for distinguishing extracellular and intracellular bacterial infections from viral infections (STAR Methods). There were two signatures with fewer than 10 genes, making them suitable for clinical translation. Of these, an 8-gene signature had consistently higher AUROCs for distinguishing viral infection from bacterial infection in both training and hold-out validation sets (Table S7). Therefore, we chose the 8-gene signature for further investigation.

The 8-gene signature included three genes over-expressed in bacterial infections (*SMARCD3*, *ICAM1*, *EBI3*; “bacterial genes”) and five over-expressed in viral infections (*JUP*, *SUCLG2*, *IFI27*, *FCER1A*, *HESX1*; “viral genes”) (Figures S3A and S3B). We defined a bacterial or viral infection (BoVI) score for each sample as the difference between the geometric mean of the expression of the three bacterial genes and the geometric mean of the five viral genes (STAR Methods). The BoVI score distinguished viral infections from extracellular and intracellular bacterial infections with high accuracy in the training (AUROC = 0.942; 95% confidence interval [CI], 0.928–0.955) and hold-out validation (AUROC = 0.947; 95% CI, 0.925–0.969) sets (Figures 3A and 3B). Importantly, the high accuracy of the BoVI score in training and hold-out validation was not dependent on any single pathogen (Figures S4A and S4B). At >90% sensitivity, the BoVI score had specificities of 84.4% and 84% in the training and the hold-out validation sets (Table 1), respectively, which met the diagnostic TPP for distinguishing bacterial infections from viral infections.[9] These high accuracies translated to positive predictive values (PPVs) >85%, negative predictive values (NPVs) $\geq$89%, and diagnostic odds ratios (DORs) >49 at 51.3% prevalence in the training set and 49.7% prevalence in the hold-out validation set, respectively (Table 1).

Validation of the BoVI score in independent retrospective datasets

We further investigated the robustness and generalizability of the BoVI score in an additional 32 independent validation datasets (23 WB and 9 PBMC) consisting of 1,889 blood samples, none of which were used in either the training or hold-out validation sets (Figure 2). In these 32 COCONUT co-normalized datasets, each of the eight genes were significantly differentially expressed (Figure S3C). The BoVI score distinguished viral infections from extracellular and intracellular bacterial infections with AUROCs ranging from 0.936 to 0.962 (Figure 3C) and was similarly accurate across a variety of pathogens (Figure S4C). The BoVI score had equal accuracy in distinguishing extracellular or intracellular bacterial infections from viral infections irrespective of which and how many datasets and samples were used (Figure S5). At >90% sensitivity, the BoVI score had 85.1% specificity (Table 1), which again met the proposed diagnostic TPP. At 45.5% prevalence of bacterial infections in the 32 COCONUT-co-normalized validation datasets, the BoVI score had 83.5% PPV and 91.2% NPV (Table 1).

Next, we validated the BoVI score in five additional datasets composed of 431 samples that profiled patients with bacterial or viral infection but did not include any healthy controls (Table S6). In these five datasets, each of the 8 genes was significantly differentially expressed (Figure S3D). Across the datasets, the BoVI score distinguished bacterial and viral infections with high accuracy (summary AUROC = 0.917; 95% CI, 0.822–1.0; range, 0.878–0.955) (Figure 3D). At >90% sensitivity, the BoVI score had 87% specificity (Table 1).[79] At 40.8% prevalence of bacterial infections in these five datasets, the BoVI score had 82.7% PPV and 92.9% NPV (Table 1).

In addition, to assess the ability of the BoVI score to generalize to pathogens that were not included in the discovery process, we tested the BoVI score in 14 datasets containing 429 samples with active TB (ATB) infection and 6 datasets containing 125 samples with HIV infection. Distinguishing TB from viral infections has been challenging to date. At 90% sensitivity, the BoVI score distinguished patients with ATB from other viral infections with ~85% specificity (Figure 3E) but did not differentiate it from other bacterial infections (Figure 3F). The BoVI score distinguished these “unseen” pathogens from each other and from the other bacterial and viral pathogens with AUROCs ranging

Figure 3. Performance of the BoVI signature in training, hold-out validation, and independent validation data

ROC curves were generated for training, hold-out validation, and both independent validation sets. The number of samples in each infection category is shown for each stage.

(A–C) Across the co-normalized data, performance was assessed for four comparisons: extracellular bacterial infections versus viral infections (red), intracellular bacterial infections versus viral infections (blue), bacterial infections with an unknown or unspecified pathogen versus viral infections (yellow), and all bacterial infections versus viral infections (black).

(D) For individual datasets, performance was assessed by pooling the bacterial versus viral AUROCs for each dataset in order to generate a summary ROC curve (black) (STAR Methods). The weighted SD for the summary ROC curve is shown in gray. Each dataset was designated as containing whole blood (WB) or peripheral blood mononuclear cell (PBMC) samples. GSE6269 was measured on two non-overlapping platforms (GPL570 and GPL2507), so each platform was assessed separately.

(E) Performance was assessed for HIV and active TB infections.

(F) Boxplots of the BoVI score across unseen pathogens. The BoVI score was centered and scaled. See also Figures S4–S6, and Tables S6 and S8.

Table 1. Diagnostic statistics for the BoVI signature across all analysis stages

Dataset group	Dataset	Prevalence of bacterial infection (%)	AUROC	Sensitivity (%)	Specificity (%)	PPV (%)	NPV (%)	LR+	LR−	DOR
Discovery datasets	training	51.3	0.94 (0.93–0.96)	90.0	84.4	85.9	89.0	5.79	0.118	49.1
	hold-out validation	49.7	0.95 (0.93–0.97)	91.7	84.0	85.0	91.1	5.74	0.099	58
Retrospective validation datasets	independent validation (COCONUT)	45.5	0.94 (0.92–0.96)	90.2	85.1	83.5	91.2	6.06	0.116	52.4
	independent validation (individual)	40.8	0.92 (0.82–1)	90.3 (85.1–93.9)	87 (71.1–94.8)	82.7 (67–92.6)	92.9 (87.4–95.8)	7.38 (2.8–19.4)	0.123 (0.078–0.192)	62 (19.9–193.6)
summary (retrospective datasets)		44.0	0.93 (0.88–0.99)	90.1 (87.5–92.3)	85.9 (74.3–92.8)	83.4 (72.8–91)	91.7 (88.3–93.9)	6.33 (3.46–11.6)	0.118 (0.093–0.151)	51.3 (26.1–100.9)
Unseen pathogen datasets	active TB versus viral independent validation	45.0	0.91 (0.89–0.93)	90.2	74	74	90.2	3.48	0.13	26.3
	bacterial versus HIV independent validation	77.8	0.94 (0.92–0.96)	90.4	85.6	95.6	71.8	6.28	0.11	55.9
	active TB versus HIV independent validation	77.4	0.9 (0.88–0.93)	90.2	64.8	89.8	65.9	2.56	0.15	17
Prospective validation datasets	Laos Fluidigm Cohort	46.1	0.96 (0.93–0.99)	96.2	87.9	87.2	96.5	7.95	0.043	184.9
	Nepal Cohort	77.5	0.91 (0.86–0.97)	90.7	76.0	92.9	70.4	3.78	0.122	31
summary (prospective datasets)		56.3	0.94 (0.89–1)	91.6 (69.4–98.1)	87.9 (81.7–92.3)	90.7 (83–94.3)	89 (67.5–97.4)	7.78 (5.04–12)	0.095 (0.02-0.4)	87 (18.3–413.4)

The AUROC and prevalence of bacterial infections were computed for each dataset, and other diagnostic statistics were computed for two different BoVI score cutoffs in each dataset. The cutoff was selected by eliminating all cutoffs with a sensitivity below 90% and then choosing the cutoff that maximized the sum of sensitivity and specificity. For independent validation (individual), summary (retrospective datasets), and summary (prospective datasets), the summary AUROC was calculated by pooling the bacterial versus viral AUROCs for each dataset (STAR Methods), the summary sensitivity, specificity, likelihood ratios, and DOR were calculated using the R package *mada*, the summary prevalence was calculated as the proportion of samples with bacterial infection across all the constituent datasets, and the NPV and PPV were calculated using the summary sensitivity/specificity and the summary prevalence. When combining statistics with *mada*, each individual cutoff was selected to maximize the sum of summary sensitivity and summary specificity. The 95% CI is included in parentheses for all relevant statistics. AUROC, area under the receiver operating characteristics curve; PPV, positive predictive value; NPV, negative predictive value; LR+, positive likelihood ratio; LR−, negative likelihood ratio; DOR, diagnostic odds ratio.

from 0.902 to 0.939 (Figures 3E and 3F; Table S8). Importantly, when we applied the 8-gene signature to only healthy controls from the studies that included patients with either TB or viral infection, it did not distinguish control cases (AUROC = 0.51; 95% CI, 0.453–0.568; Figure S6), demonstrating that, after COCONUT normalization, the discovery of the 8-gene signature is not affected by batch effects. These results suggest that the 8-gene signature could be integrated with a host-response signature for diagnosis of TB (e.g., a 3-gene signature for diagnosis of TB[26] that has been translated and validated as a point-of-care test[80–83]) to further improve diagnosis of ATB.

Overall, across the 37 independent validation datasets composed of 2,320 samples, the BoVI score had a summary AUROC of 0.932 (95% CI, 0.875–0.989). At >90% sensitivity, the BoVI score had 85.9% specificity, a DOR of 51.3, and 83.4% PPV and 91.7% NPV at 44% prevalence of bacterial infections (Table 1). Hence, the BoVI score accurately distinguished patients with viral infection from those with bacterial infection, irrespective of extracellular or intracellular bacterial infection, and met the proposed diagnostic TPP.

The BoVI score is robust to differences in age, sex, race, and days of fever

Several datasets provided detailed demographic information for individual patients including race, sex, age (or age range), and days since fever onset. We note that these datasets reported sex, not environmentally defined gender. In datasets where sex was not explicitly recorded, we imputed it using expression of a set of genes located on the Y chromosome and a set of known X-escape genes.[84,85] Overall, we had data on age, race, sex, and days since fever onset for 1,787, 1,376, 2,762, and 1,053 samples, respectively, across the 69 datasets used in our analysis. Across a wide range of ages, from less than 6 months to more than 80 years, the BoVI score was consistently higher in patients with bacterial infection than in those with viral infections and distinguished them with high accuracy (AUROC range: 0.844–0.956 in training/hold-out validation, 0.878–0.97 in independent validation; Figures 4A and S7). The BoVI score also diagnosed whether a patient had a bacterial or viral infection with high accuracy irrespective of race (AUROC range: 0.911–0.970 in training/hold-out validation, 0.954–0.963 in independent validation; Figures 4B and S8) and sex (AUROC range: 0.927–0.947 in training/hold-out validation, 0.943–0.955 in independent validation; Figures 4C and S9). Finally, the BoVI score maintained high discriminative power irrespective of the number of days since fever onset (AUROC range: 0.872–1 in training/hold-out validation, 0.88–0.992 in independent validation; Figures 4D and S10). Across 1,053 samples for which age, race, and sex information was available, a multivariable logistic regression found that the BoVI score, age, and black patients had significantly positive associations with bacterial infection (Table S9).

Validation of the BoVI score in two independent prospective cohorts using Fluidigm RT-PCR

Finally, we validated the BoVI score in two cohorts of febrile patients in two countries, Laos (Table S10) and Nepal (Table S11), using Fluidigm RT-PCR. The main intracellular bacterial pathogens in these two cohorts included *O. tsutsugamushi*, *R. typhi*, *B. pseudomallei*, and *S.* Typhi. The Laos Fluidigm cohort consisted of 230 WB samples (49 extracellular bacterial infections, 57 intracellular bacterial infections, 124 viral infections) from febrile inpatients over the age of 15 years. We excluded any patients that had been enrolled in the Laos Microarray cohort. The BoVI score had an overall AUROC of 0.96 (95% CI, 0.933–0.987) for distinguishing bacterial and viral infections with virtually identical accuracy for distinguishing extracellular bacterial (AUROC = 0.957; 95% CI, 0.917–0.998) and intracellular bacterial (AUROC = 0.962; 95% CI, 0.926–0.997) infections from viral infections (Figure 5A), irrespective of the pathogen (Figure S11A). At 96.2% sensitivity, the BoVI score had 87.9% specificity and a DOR of 184.9. At 46.1% prevalence of bacterial infections in the Laos Fluidigm cohort, the BoVI score had 87.2% PPV and 96.5% NPV (Table 1).

The Nepal cohort consisted of 111 WB samples (15 extracellular bacterial, 71 intracellular bacterial, 25 viral) from individuals >12 months of age presenting to the hospital with a self-reported >72-h history of fever. The BoVI score distinguished bacterial and viral infections with an overall AUROC of 0.914 (95% CI, 0.862–0.966) with almost the same accuracy for extracellular bacterial (AUROC = 0.91; 95% CI, 0.816–1.0) and intracellular bacterial (AUROC = 0.915; 95% CI, 0.859–1.0) infections (Figure 5B), irrespective of the pathogen (Figure S11B). At >90% sensitivity, the BoVI score had 76% specificity and a DOR of 31 (Table 1). At 77.5% prevalence of bacterial infections in the Nepal cohort, the BoVI score had 92.9% PPV and 70.4% NPV (Table 1).

Together, across 341 samples in two prospective cohorts from two countries, the BoVI score had 91.6% specificity at 87.9% sensitivity and a DOR of 87, which met the diagnostic TPP. At 56.3% prevalence of bacterial infections in both cohorts combined, the BoVI score had 90.7% PPV and 89% NPV (Table 1).

DISCUSSION

Overuse and misuse of antimicrobial agents continues to drive antimicrobial resistance on a global scale. Antimicrobial agents are overused or misused for several reasons, including inappropriate broad coverage due to an incomplete understanding of local epidemiology and inadequate diagnostics. Our comparison of four host-response gene signatures for distinguishing bacterial and viral infections found that current signatures have significantly lower accuracy for distinguishing intracellular bacterial infections from viral infections, which limits their clinical utility to settings where these infections are not prevalent.[86]

Arguably, we could have grouped our datasets as from LMICs and non-LMICs to investigate generalizability of the four previously described host response-based signatures. However, datasets from non-LMICs had an over-representation of mild viral infections (e.g., rhinovirus), whereas datasets from LMICs had severe viral infections over-represented. In other words, grouping datasets by LMIC and non-LMIC settings was confounded by disease severity.

We applied MANATEE, a multi-cohort analysis framework, to address this challenge, and performed the largest multi-cohort analysis to date of blood transcriptome profiles from patients with bacterial or viral infections. Our analysis of 4,200

CellPress
OPEN ACCESS

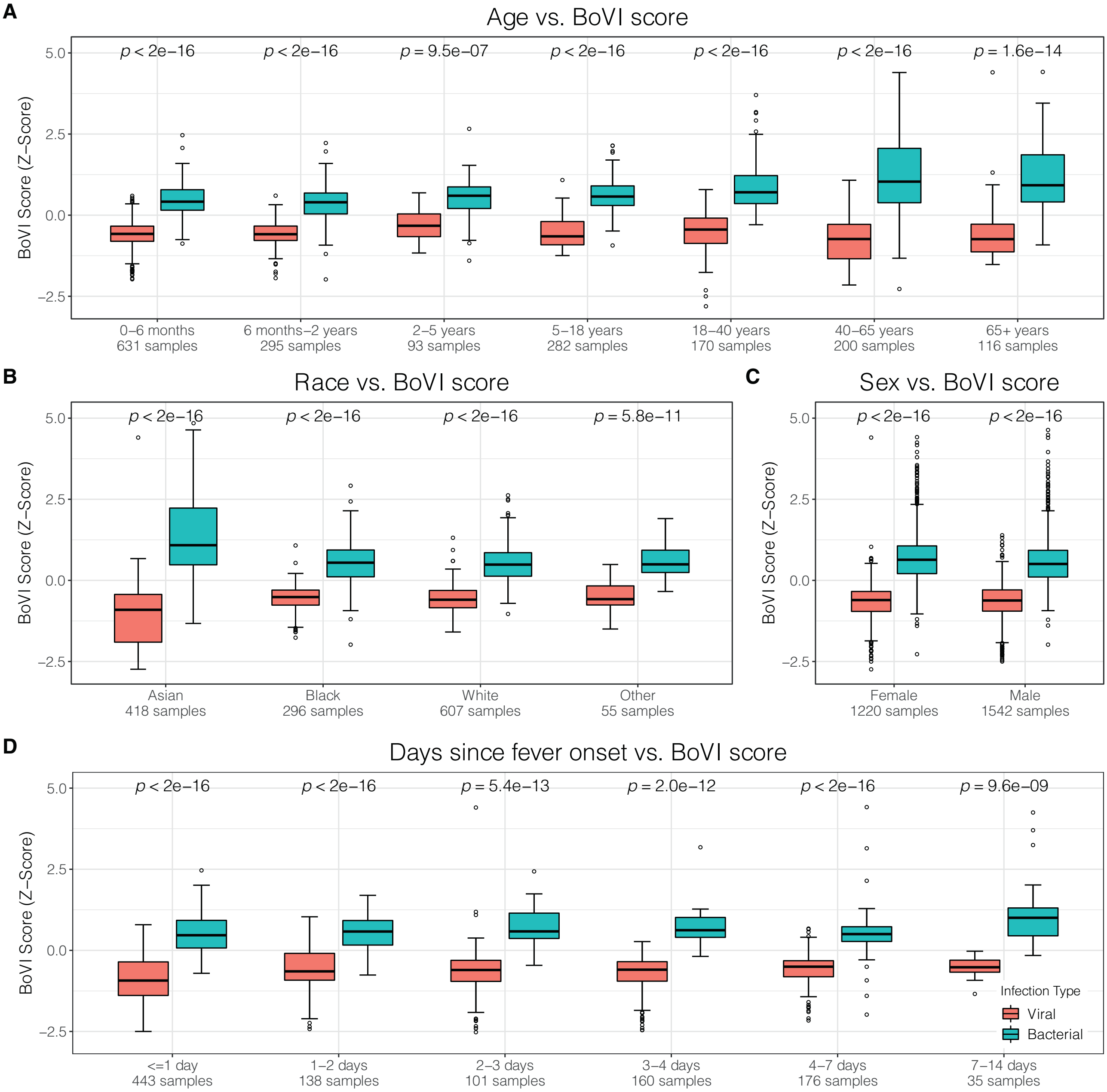

Figure 4. The BoVI signature maintains high accuracy regardless of age, race, sex, or days since fever onset

Performance of the BoVI signature was calculated for each category across training, hold-out validation, and COCONUT co-normalized independent validation samples.

Scores were centered at 0 and then the *Z* score was calculated.

Centering and scaling was done separately for training/hold-out validation and for independent validation.

The p values were calculated using the Mann-Whitney U test.

(A) Boxplots of the BoVI score across each age category. Samples were used only if (1) the exact age was listed, (2) a range of ages was provided that fit completely into a single age window, or (3) the mean and SD of the ages was provided and all ages that were within one SD of the mean fit into a single age window. Overall, 1,787 samples had usable age information.

(B) Boxplots of the BoVI score across each race category. Overall, there were 1,376 samples with information on race.

(C) Boxplots of the BoVI score for either female or male patients. Overall, there were 2,762 samples with sex listed or with sex imputed using a set of Y-linked genes and a set of known X-escape genes, as described in Bongen et al.[84]

(D) Boxplots of the BoVI score across each day since fever onset category. Overall, there were 1,053 samples with information on day since fever onset. See also Figures S7–S10 and Table S9.

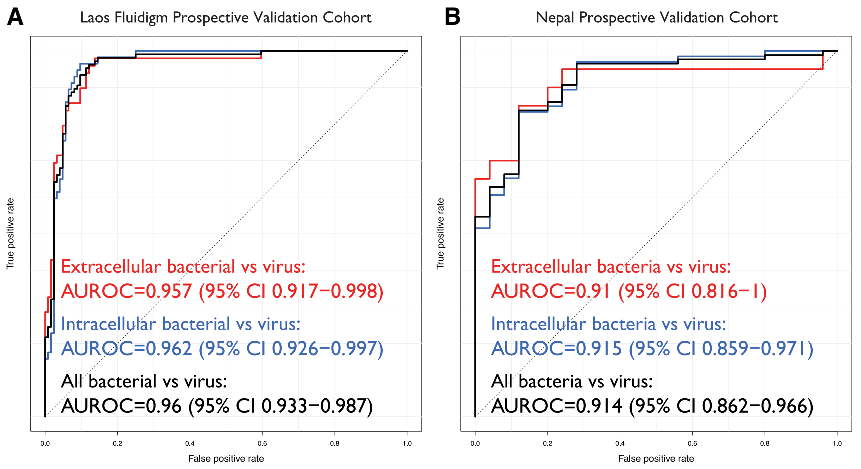

Figure 5. Performance of the BoVI signature in the Laos Fluidigm and Nepal prospective validation cohorts

(A) AUROC using the BoVI signature in the Laos cohort.

(B) AUROC using the BoVI signature in the Nepal cohort.

Performance was assessed for three comparisons: extracellular bacterial infections versus viral infections (red), intracellular bacterial infections versus viral infections (blue), and all bacterial infections versus viral infections (black). See also Figure S11 and Tables S10–S12.

transcriptome profiles represented biological, clinical, and technical heterogeneity observed in the worldwide population by including substantial diversity of pathogens, host genetics, ethnic and racial backgrounds, and environmental exposures from different geographic regions, ages, sex, and clinical severity. We identified and extensively validated an 8-gene signature that distinguished both extracellular and intracellular bacterial infections from viral infections with high accuracy and met the proposed diagnostic TPP, despite broad heterogeneity in age, sex, race, days since fever onset, and severity of infection, demonstrating its robustness across heterogeneous clinical populations. The 8-gene signature had slightly lower accuracy in the Nepal cohort, which could be due to the large proportion of identified human herpesvirus 6 (HHV6)- and CMV-infected patients, viruses for which testing was not performed in the Lao cohorts. These viruses may be detected and have disease attributed to them even when they are not the causative agent of acute illness.[87,88]

Across 37 retrospective and 2 prospective cohorts used for validation, the BoVI score has low negative likelihood ratios (<0.12), high positive likelihood ratios ($\geq$6.33), and high PPV and NPV ($\geq$83%). These test characteristics are substantially higher than other host-response-based diagnostics,[89] which suggest that it would be potentially effective as a rule-out and a rule-in test. Moreover, these test characteristics assume no knowledge of the patient and thus are only estimates of the real-world clinical utility of such a test, since patient history, physical examination, vital signs, and laboratory values would all assist in a diagnosis as well. Ultimately, only interventional trials will be able to establish the cost-effectiveness and clinical utility of a diagnostic test.

We validated the BoVI score in two prospective cohorts using Fluidigm RT-PCR, demonstrating that the score is robust. The parsimony of our 8-gene signature makes it an ideal candidate for translation to a point-of-care test as several commercial platforms capable of measuring multiple mRNAs in a short time are available.[90] For example, the GeneXpert platform from Cepheid has successfully translated a 3-gene signature for diagnosis of active tuberculosis[26] into a point-of-care diagnostic cartridge with high accuracy in a prospective cohort.[27,28]

The identity of the genes in our signature is consistent with the known biology of the response to infection. *ICAM1* is a well-studied cell surface glycoprotein that is expressed by many immune cells and is upregulated in response to lipopolysaccharide (LPS) exposure.[91] *SMARCD3* and *EBI3*, two genes over-expressed in bacterial infection, are associated with tuberculosis infection and bacterial burden.[92,93] Of the viral genes, *IFI27* and *JUP* are known to be upregulated in response to viral infection.[42,94] Furthermore, *FCER1A* encodes a subunit of the immunoglobulin (Ig) E receptor and is thought to be involved in the immune response to viral infections.[95] Hence, selection of these transcripts as biomarkers for distinguishing bacterial and viral infections makes sense biologically based on known features of host immune response to the type of pathogens.

Limitations of the study

Our study has a few limitations. First, COCONUT co-normalization may have removed some biological variation between the immune responses measured in different patient groups. However, our results demonstrate that the effects of acute bacterial or viral infection on the host immune response are robustly detectable across cohorts, even in datasets that were separately co-normalized or datasets that were not co-normalized. Second, we validated the 8-gene signature in two independent cohorts that used only microbiologically confirmed patients in case-control design and did not establish accuracy of the 8-gene signature against current biomarkers of bacterial infection such as CRP and PCT. The performance characteristics of the 8-gene signature will differ in patient cohorts that rely on non-microbiological adjudication methods, which are known to suffer from high inter-rater variations, leading to a variable standard. We were also not able to assess the positive predictive value for subsequent microbiological confirmation of either bacterial or viral infections. The 8-gene signature should be validated in additional prospective cohorts from other populations and where microbiological diagnosis is not available. Third, although we demonstrated that the 8-gene signature can distinguish TB from viral infections, it was dependent on merging datasets that included either viral or TB cases. The 8-gene signature should be validated in future studies that include both classes within the same study. Fourth, there were no TB, malaria, or other protozoal/fungal infection cases in the two prospective

cohorts, although we did not systematically exclude them. However, these infections can present as an acute febrile illness. Hence, the 8-gene signature should be validated in the future studies that include these infections.

In summary, by leveraging the large amount of heterogeneity present across tens of independent datasets composed of thousands of blood samples, we ensured that our data were representative of real-world global patient populations. In turn, we utilized biological, clinical, and technical heterogeneity in these cohorts to identify a parsimonious and robust host-response-based 8-gene signature for distinguishing patients with bacterial infections from those with viral infections that generalizes to global populations. The signature consistently met the TPP for distinguishing bacterial and viral infections in both retrospective and prospective cohorts. Its parsimony makes it an ideal candidate for translation to a point-of-care test, which in turn has the potential to improve targeted patient therapy and to help reduce the overuse of antimicrobial drugs.

STAR★METHODS

Detailed methods are provided in the online version of this paper and include the following:

- KEY RESOURCES TABLE
- RESOURCE AVAILABILITY
  - Lead contact
  - Materials availability
  - Data and code availability
- EXPERIMENTAL MODEL AND SUBJECT DETAILS
  - Search for gene expression datasets
  - Laos cohorts
  - Nepal cohort
- METHOD DETAILS
- QUANTIFICATION AND STATISTICAL ANALYSIS
  - COCONUT co-normalization
  - Validation of published signatures
  - Derivation of the 8-gene signature with MANATEE
  - Calculation of the BoVI score
  - Summary ROC curves
  - Selecting optimal cutoffs with the Youden Index
  - Whole genome transcript profiling
  - Fluidigm RT-PCR validation

SUPPLEMENTAL INFORMATION

Supplemental information can be found online at https://doi.org/10.1016/j.xcrm.2022.100842.

ACKNOWLEDGMENTS

P.K. is funded in part by the Bill and Melinda Gates Foundation (OPP1113682); the National Institute of Allergy and Infectious Diseases (NIAID) grants 1U19AI167903-01, 5U01AI165527-02, 1U19AI109662, U19AI057229, and 5R01AI125197; Department of Defense contracts W81XWH-18-1-0253 and W81XWH1910235; and the Ralph & Marian Falk Medical Research Trust. D.A.R. is supported by NIH/NIAID U19 AI109761 and the Thomas C. and Joan M. Merigan Endowment at Stanford University. Lao-Oxford-Mahosot Hospital-Wellcome Trust Research Unit is funded by the Wellcome Trust of Great Britain. A.M.R. is funded by the National Science Foundation Graduate Research Fellowship and the Stanford Graduate Fellowship. This research was funded in whole, or in part, by the Wellcome Trust (grant number 220211). For the purpose of Open Access, the author has applied a CC BY public copyright license to any Author Accepted Manuscript version arising from this submission.

We are very grateful to the late Dr. Rattanaphone Phetsouvanh, Dr. Vilada Chansamouth, Dr. Sayaphet Rattanavong, Dr. Koukeo Phommasone, the patients, and to Assoc. Prof. Bounthaphany Bounxouei, ex-Director of Mahosot Hospital, and the staff of Mahosot Hospital and the Microbiology Laboratory for their technical help and support. We thank Assoc. Prof. Bounnack Saysanasongkham, the ex-Director of Department of Health Care, Ministry of Health; Assoc. Prof. Bounkong Syhavong, the former Minister of Health, Lao PDR; and Dr. Catrin Moore for their very kind help and support. We thank Stuart Blacksell, Audrey Dubot-Pérès, and Audrey Rachlin for laboratory support. We also thank Veda Khadka for technical assistance.

AUTHOR CONTRIBUTIONS

A.M.R., S.J.P., and P.K. conceived the project. A.M.R. collected and curated datasets and performed the primary analysis. S.J.P. and S.G. assisted with analysis. A.M.R. and S.J.P. had full access to the data and verified the data. Patient recruitment, sample collection, and sample processing was done by S.J.P., V.D., K.V., A.C., M.V., M.M., P.N., S.A.T., S.D., M.T.R., B.K., and I.B., and overseen by P.N.N. and J.R.A. S.J.P., A.M.R., and S.A.T. curated patient data. A.M.R. and P.K. wrote and revised the manuscript with input from all authors, who reviewed and approved the final submitted paper. P.K. and D.A.R. supervised the project and were responsible for the decision to submit the paper.

DECLARATION OF INTERESTS

P.N. is also an honorary professor at National University of Lao, London School of Hygiene and Tropical Medicine, and Boston University. He receives no salary or other financial support from these institutions. D.R. is a shareholder of Blue Willow Biologics, Cantata Bio, Evelo Biosciences, Karius, Proderm IQ, and Second Genome. D.R. is an advisor to Cantata Bio and Visby Medical. A.M.R., S.J.P., T.E.S., D.A.R., and P.K. are named as inventors on a patent application describing the 8-gene set, which has been licensed to Inflammatix. T.E.S. and P.K. are employees of Inflammatix and are shareholders in Inflammatix. Inflammatix had no role in the design, funding, or reporting of this work. S.D. is currently employed by FIND.

Received: March 11, 2022
Revised: July 12, 2022
Accepted: November 9, 2022
Published: December 20, 2022

REFERENCES

1. Crump, J.A., Morrissey, A.B., Nicholson, W.L., Massung, R.F., Stoddard, R.A., Galloway, R.L., Ooi, E.E., Maro, V.P., Saganda, W., Kinabo, G.D., et al. (2013). Etiology of severe non-malaria febrile illness in Northern Tanzania: a prospective cohort study. PLoS Negl. Trop. Dis. *7*, e2324.
2. Mayxay, M., Castonguay-Vanier, J., Chansamouth, V., Dubot-Pérès, A., Paris, D.H., Phetsouvanh, R., Tangkhabuanbutra, J., Douangdala, P., Inthalath, S., Souvannasing, P., et al. (2013). Causes of non-malarial fever in Laos: a prospective study. Lancet. Glob. Health *1*, e46–e54.
3. Fleming-Dutra, K.E., Hersh, A.L., Shapiro, D.J., Bartoces, M., Enns, E.A., File, T.M., Jr., Finkelstein, J.A., Gerber, J.S., Hyun, D.Y., Linder, J.A., et al. (2016). Prevalence of inappropriate antibiotic prescriptions among US Ambulatory care Visits, 2010-2011. JAMA *315*, 1864–1873.
4. Silverman, M., Povitz, M., Sontrop, J.M., and Shariff, S.Z. (2017). Antibiotic prescribing for Nonbacterial acute Upper respiratory infections in Elderly Persons. Ann. Intern. Med. *167*, 758–759.
5. Andrews, J.R., Vaidya, K., Bern, C., Tamrakar, D., Wen, S., Madhup, S., Shrestha, R., Karmacharya, B., Amatya, B., Koju, R., et al. (2018). High

rates of enteric fever diagnosis and lower burden of culture-confirmed disease in Peri-urban and Rural Nepal. J. Infect. Dis. *218*, S214–S221.

6. Gwimile, J.J., Shekalaghe, S.A., Kapanda, G.N., and Kisanga, E.R. (2012). Antibiotic prescribing practice in management of cough and/or diarrhoea in Moshi Municipality, Northern Tanzania: cross-sectional descriptive study. Pan Afr. Med. J. *12*, 103.

7. Laxminarayan, R., Duse, A., Wattal, C., Zaidi, A.K.M., Wertheim, H.F.L., Sumpradit, N., Vlieghe, E., Hara, G.L., Gould, I.M., Goossens, H., et al. (2013). Antibiotic resistance-the need for global solutions. Lancet Infect. Dis. *13*, 1057–1098.

8. Andrews, J.R., Qamar, F.N., Charles, R.C., and Ryan, E.T. (2018). Extensively Drug-Resistant typhoid — are Conjugate Vaccines Arriving just in time? N. Engl. J. Med. *379*, 1493–1495.

9. Dittrich, S., Tadesse, B.T., Moussy, F., Chua, A., Zorzet, A., Tängdén, T., Dolinger, D.L., Page, A.L., Crump, J.A., D'Acremont, V., et al. (2016). Target product profile for a diagnostic assay to differentiate between bacterial and non-bacterial infections and reduce antimicrobial overuse in Resource-limited settings: an Expert Consensus. PLoS One *11*, e0161721.

10. Hogan, C.A., Yang, S., Garner, O.B., Green, D.A., Gomez, C.A., Dien Bard, J., Pinsky, B.A., and Banaei, N. (2021). Clinical Impact of metagenomic next-generation sequencing of plasma cell-Free DNA for the diagnosis of infectious diseases: a Multicenter retrospective cohort study. Clin. Infect. Dis. *72*, 239–245. https://doi.org/10.1093/cid/ciaa035.

11. Wilson, M.R., Naccache, S.N., Samayoa, E., Biagtan, M., Bashir, H., Yu, G., Salamat, S.M., Somasekar, S., Federman, S., Miller, S., et al. (2014). Actionable diagnosis of neuroleptospirosis by next-generation sequencing. N. Engl. J. Med. *370*, 2408–2417.

12. Babady, N.E. (2021). Clinical metagenomics for Bloodstream infections: is the Juice Worth the Squeeze? Clin. Infect. Dis. *72*, 246–248. https://doi.org/10.1093/cid/ciaa041.

13. Watson, J., Salisbury, C., Whiting, P., Banks, J., Pyne, Y., and Hamilton, W. (2019). Added value and cascade effects of inflammatory marker tests in UK primary care: a cohort study from the Clinical Practice Research Datalink. Br. J. Gen. Pract. *69*, e470–e478.

14. Herberg, J.A., Kaforou, M., Wright, V.J., Shailes, H., Eleftherohorinou, H., Hoggart, C.J., Cebey-López, M., Carter, M.J., Janes, V.A., Gormley, S., et al. (2016). Diagnostic test accuracy of a 2-transcript host RNA signature for discriminating bacterial vs viral infection in febrile children. JAMA *316*, 835–845.

15. Suarez, N.M., Bunsow, E., Falsey, A.R., Walsh, E.E., Mejias, A., and Ramilo, O. (2015). Superiority of transcriptional profiling over procalcitonin for distinguishing bacterial from viral lower respiratory tract infections in hospitalized adults. JID (J. Infect. Dis.) *212*, 213–222. Oxford University Press.

16. Zaas, A.K., Chen, M., Varkey, J., Veldman, T., Hero, A.O., 3rd, Lucas, J., Huang, Y., Turner, R., Gilbert, A., Lambkin-Williams, R., et al. (2009). Gene expression signatures diagnose influenza and other symptomatic respiratory viral infections in humans. Cell Host Microbe *6*, 207–217.

17. Lubell, Y., Althaus, T., Blacksell, S.D., Paris, D.H., Mayxay, M., Pan-Ngum, W., White, L.J., Day, N.P.J., and Newton, P.N. (2016). Modelling the Impact and cost-effectiveness of biomarker tests as compared with pathogen-specific diagnostics in the management of Undifferentiated fever in Remote Tropical settings. PLoS One *11*, e0152420.

18. Prasad, N., Murdoch, D.R., Reyburn, H., and Crump, J.A. (2015). Etiology of severe febrile illness in low- and middle-income countries: a systematic review. PLoS One *10*, e0127962.

19. Rudd, K.E., Johnson, S.C., Agesa, K.M., Shackelford, K.A., Tsoi, D., Kievlan, D.R., Colombara, D.V., Ikuta, K.S., Kissoon, N., Finfer, S., et al. (2020). Global, regional, and national sepsis incidence and mortality, 1990-2017: analysis for the Global Burden of Disease Study. Lancet *395*, 200–211.

20. GBD 2017 Typhoid and Paratyphoid Collaborators (2019). The global burden of typhoid and paratyphoid fevers: a systematic analysis for the Global Burden of Disease Study 2017. Lancet Infect. Dis. *19*, 369–381.

21. Bhatt, S., Gething, P.W., Brady, O.J., Messina, J.P., Farlow, A.W., Moyes, C.L., Drake, J.M., Brownstein, J.S., Hoen, A.G., Sankoh, O., et al. (2013). The global distribution and burden of dengue. Nature *496*, 504–507.

22. Bonell, A., Lubell, Y., Newton, P.N., Crump, J.A., and Paris, D.H. (2017). Estimating the burden of scrub typhus: a systematic review. PLoS Negl. Trop. Dis. *11*, e0005838.

23. Blohmke, C.J., Darton, T.C., Jones, C., Suarez, N.M., Waddington, C.S., Angus, B., Zhou, L., Hill, J., Clare, S., Kane, L., et al. (2016). Interferon-driven alterations of the host's amino acid metabolism in the pathogenesis of typhoid fever. J. Exp. Med. *213*, 1061–1077.

24. Thompson, L.J., Dunstan, S.J., Dolecek, C., Perkins, T., House, D., Dougan, G., Nguyen, T.H., Tran, T.P.L., Doan, C.D., Le, T.P., et al. (2009). Transcriptional response in the peripheral blood of patients infected with Salmonella enterica serovar Typhi. Proc. Natl. Acad. Sci. USA *106*, 22433–22438.

25. Suliman, S., Thompson, E.G., Sutherland, J., Weiner, J., 3rd, Ota, M.O.C., Shankar, S., Penn-Nicholson, A., Thiel, B., Erasmus, M., Maertzdorf, J., et al. (2018). Four-gene pan-African blood signature predicts progression to tuberculosis. Am. J. Respir. Crit. Care Med. *197*, 1198–1208.

26. Sweeney, T.E., Braviak, L., Tato, C.M., and Khatri, P. (2016). Genome-wide expression for diagnosis of pulmonary tuberculosis: a multicohort analysis. Lancet Respir. Med. *4*, 213–224.

27. Södersten, E., Ongarello, S., Mantsoki, A., Wyss, R., Persing, D.H., Banderby, S., et al. (2020). Diagnostic accuracy study of a novel blood-based assay for identification of TB in people living with HIV. J. Clin. Microbiol. *59*.

28. Sutherland, J.S., van der Spuy, G., Gindeh, A., Thuong, N.T.T., Namuganga, A., Owolabi, O., Mayanja-Kizza, H., Nsereko, M., Thwaites, G., Winter, J., et al. (2022). Diagnostic accuracy of the Cepheid 3-gene host response fingerstick blood test in a prospective, multi-site study: interim results. Clin. Infect. Dis. *74*, 2136–2141. https://doi.org/10.1093/CID/CIAB839.

29. Maslove, D.M., Shapira, T., Tyryshkin, K., Veldhoen, R.A., Marshall, J.C., and Muscedere, J. (2019). Validation of diagnostic gene sets to identify critically ill patients with sepsis. J. Crit. Care *49*, 92–98.

30. Thair, S., Mewes, C., Hinz, J., Bergmann, I., Büttner, B., Sehmisch, S., Meissner, K., Quintel, M., Sweeney, T.E., Khatri, P., and Mansur, A. (2021). Gene expression–based diagnosis of infections in critically ill patients—prospective validation of the SepsisMetaScore in a Longitudinal severe Trauma cohort. Crit. Care Med. *49*, e751–e760. https://doi.org/10.1097/CCM.0000000000005027.

31. Edgar, R., Domrachev, M., and Lash, A.E. (2002). Gene Expression Omnibus: NCBI gene expression and hybridization array data repository. Nucleic Acids Res. *30*, 207–210.

32. Kolesnikov, N., Hastings, E., Keays, M., Melnichuk, O., Tang, Y.A., Williams, E., Dylag, M., Kurbatova, N., Brandizi, M., Burdett, T., et al. (2015). ArrayExpress update-simplifying data submissions. Nucleic Acids Res. *43*, D1113–D1116.

33. Popper, S.J., Gordon, A., Liu, M., Balmaseda, A., Harris, E., and Relman, D.A. (2012). Temporal dynamics of the transcriptional response to dengue virus infection in Nicaraguan children. PLoS Negl. Trop. Dis. *6*, e1966.

34. Koh, G.C.K.W., Schreiber, M.F., Bautista, R., Maude, R.R., Dunachie, S., Limmathurotsakul, D., Day, N.P.J., Dougan, G., and Peacock, S.J. (2013). Host responses to melioidosis and tuberculosis are both dominated by interferon-mediated signaling. PLoS One *8*, e54961.

35. Ardura, M.I., Banchereau, R., Mejias, A., Di Pucchio, T., Glaser, C., Allantaz, F., Pascual, V., Banchereau, J., Chaussabel, D., and Ramilo, O.

CellPress
OPEN ACCESS

(2009). Enhanced monocyte response and decreased central memory T cells in children with invasive Staphylococcus aureus infections. PLoS One *4*, e5446.

36. Parnell, G.P., McLean, A.S., Booth, D.R., Armstrong, N.J., Nalos, M., Huang, S.J., Manak, J., Tang, W., Tam, O.Y., Chan, S., and Tang, B.M. (2012). A distinct influenza infection signature in the blood transcriptome of patients with severe community-acquired pneumonia. Crit. Care *16*, R157.

37. Banchereau, R., Jordan-Villegas, A., Ardura, M., Mejias, A., Baldwin, N., Xu, H., Saye, E., Rossello-Urgell, J., Nguyen, P., Blankenship, D., et al. (2012). Host immune transcriptional profiles reflect the variability in clinical disease manifestations in patients with staphylococcus aureus infections. PLoS One *7*, e34390.

38. Berdal, J.E., Mollnes, T.E., Wæhre, T., Olstad, O.K., Halvorsen, B., Ueland, T., Laake, J.H., Furuseth, M.T., Maagaard, A., Kjekshus, H., et al. (2011). Excessive innate immune response and mutant D222G/N in severe A (H1N1) pandemic influenza. J. Infect. *63*, 308–316.

39. de Steenhuijsen Piters, W.A.A., Heinonen, S., Hasrat, R., Bunsow, E., Smith, B., Suarez-Arrabal, M.C., Chaussabel, D., Cohen, D.M., Sanders, E.A.M., Ramilo, O., et al. (2016). Nasopharyngeal Microbiota, host transcriptome, and disease severity in children with respiratory syncytial virus infection. Am. J. Respir. Crit. Care Med. *194*, 1104–1115.

40. Heinonen, S., Jartti, T., Garcia, C., Oliva, S., Smitherman, C., Anguiano, E., de Steenhuijsen Piters, W.A.A., Vuorinen, T., Ruuskanen, O., Dimo, B., et al. (2016). Rhinovirus detection in symptomatic and asymptomatic children value of host transcriptome analysis. Am. J. Respir. Crit. Care Med. *193*, 772–782.

41. Herberg, J.A., Kaforou, M., Gormley, S., Sumner, E.R., Patel, S., Jones, K.D.J., Paulus, S., Fink, C., Martinon-Torres, F., Montana, G., et al. (2013). Transcriptomic profiling in childhood H1N1/09 influenza reveals reduced expression of protein synthesis genes. J. Infect. Dis. *208*, 1664–1668.

42. Ioannidis, I., McNally, B., Willette, M., Peeples, M.E., Chaussabel, D., Durbin, J.E., Ramilo, O., Mejias, A., and Flaño, E. (2012). Plasticity and virus specificity of the airway epithelial cell immune response during respiratory virus infection. J. Virol. *86*, 5422–5436.

43. Jong, V.L., Ahout, I.M.L., van den Ham, H.J., Jans, J., Zaaraoui-Boutahar, F., Zomer, A., Simonetti, E., Bijl, M.A., Brand, H.K., van IJcken, W.F.J., et al. (2016). Transcriptome assists prognosis of disease severity in respiratory syncytial virus infected infants. Sci. Rep. *6*, 36603–36612.

44. Lindow, J.C., Wunder, E.A., Jr., Popper, S.J., Min, J.N., Mannam, P., Srivastava, A., Yao, Y., Hacker, K.P., Raddassi, K., Lee, P.J., et al. (2016). Cathelicidin Insufficiency in patients with Fatal leptospirosis. PLoS Pathog. *12*, e1005943.

45. Liu, T.Y., Burke, T., Park, L.P., Woods, C.W., Zaas, A.K., Ginsburg, G.S., and Hero, A.O. (2016). An individualized predictor of health and disease using paired reference and target samples. BMC Bioinf. *17*, 47.

46. Mejias, A., Dimo, B., Suarez, N.M., Garcia, C., Suarez-Arrabal, M.C., Jartti, T., Blankenship, D., Jordan-Villegas, A., Ardura, M.I., Xu, Z., et al. (2013). Whole blood gene expression profiles to assess pathogenesis and disease severity in infants with respiratory syncytial virus infection. PLoS Med. *10*, e1001549.

47. Rodriguez-Fernandez, R., Tapia, L.I., Yang, C.F., Torres, J.P., Chavez-Bueno, S., Garcia, C., Jaramillo, L.M., Moore-Clingenpeel, M., Jafri, H.S., Peeples, M.E., et al. (2017). Respiratory syncytial virus Genotypes, host immune profiles, and disease severity in young children hospitalized with Bronchiolitis. J. Infect. Dis. *217*, 24–34.

48. Tang, B.M., Shojaei, M., Parnell, G.P., Huang, S., Nalos, M., Teoh, S., O'Connor, K., Schibeci, S., Phu, A.L., Kumar, A., et al. (2017). A novel immune biomarker IFI27 discriminates between influenza and bacteria in patients with suspected respiratory infection. Eur. Respir. J. *49*, 1602098.

49. Ahn, S.H., Tsalik, E.L., Cyr, D.D., Zhang, Y., van Velkinburgh, J.C., Langley, R.J., Glickman, S.W., Cairns, C.B., Zaas, A.K., Rivers, E.P., et al. (2013). Gene expression-based classifiers identify Staphylococcus aureus infection in mice and humans. PLoS One *8*, e48979.

50. Bermejo-Martin, J.F., Martin-Loeches, I., Rello, J., Antón, A., Almansa, R., Xu, L., Lopez-Campos, G., Pumarola, T., Ran, L., Ramirez, P., et al. (2010). Host adaptive immunity deficiency in severe pandemic influenza. Crit. Care *14*, R167.

51. Berry, M.P.R., Graham, C.M., McNab, F.W., Xu, Z., Bloch, S.A.A., Oni, T., Wilkinson, K.A., Banchereau, R., Skinner, J., Wilkinson, R.J., et al. (2010). An interferon-inducible neutrophil-driven blood transcriptional signature in human tuberculosis. Nature *466*, 973–977.

52. Chaussabel, D., Quinn, C., Shen, J., Patel, P., Glaser, C., Baldwin, N., Stichweh, D., Blankenship, D., Li, L., Munagala, I., et al. (2008). A modular analysis framework for blood genomics studies: application to systemic lupus erythematosus. Immunity *29*, 150–164.

53. Cvijanovich, N., Shanley, T.P., Lin, R., Allen, G.L., Thomas, N.J., Checchia, P., Anas, N., Freishtat, R.J., Monaco, M., Odoms, K., et al. (2008). Validating the genomic signature of pediatric septic shock. Physiol. Genomics *34*, 127–134.

54. Hu, X., Yu, J., Crosby, S.D., and Storch, G.A. (2013). Gene expression profiles in febrile children with defined viral and bacterial infection. Proc. Natl. Acad. Sci. USA *110*, 12792–12797.

55. Irwin, A.D., Marriage, F., Mankhambo, L.A., IPD Group Study; Jeffers, G., Kolamunnage-Dona, R., Guiver, M., Denis, B., Molyneux, E.M., Molyneux, M.E., et al. (2012). Novel biomarker combination improves the diagnosis of serious bacterial infections in Malawian children. BMC Med. Genomics *5*, 13.

56. Jaggi, P., Mejias, A., Xu, Z., Yin, H., Moore-Clingenpeel, M., Smith, B., Burns, J.C., Tremoulet, A.H., Jordan-Villegas, A., Chaussabel, D., et al. (2018). Whole blood transcriptional profiles as a prognostic tool in complete and incomplete Kawasaki Disease. PLoS One *13*, e0197858.

57. Kulohoma, B.W., Marriage, F., Vasieva, O., Mankhambo, L., Nguyen, K., Molyneux, M.E., Molyneux, E.M., Day, P.J.R., and Carrol, E.D. (2017). Peripheral blood RNA gene expression in children with pneumococcal meningitis: a prospective case-control study. BMJ Paediatr. Open *1*, e000092.

58. Kwissa, M., Nakaya, H.I., Onlamoon, N., Wrammert, J., Villinger, F., Perng, G.C., Yoksan, S., Pattanapanyasat, K., Chokephaibulkit, K., Ahmed, R., and Pulendran, B. (2014). Dengue virus infection induces expansion of a CD14(+)CD16(+) monocyte population that stimulates plasmablast differentiation. Cell Host Microbe *16*, 115–127.

59. Lill, M., Kõks, S., Soomets, U., Schalkwyk, L.C., Fernandes, C., Lutsar, I., and Taba, P. (2013). Peripheral blood RNA gene expression profiling in patients with bacterial meningitis. Front. Neurosci. *7*, 33.

60. Nascimento, E.J.M., Braga-Neto, U., Calzavara-Silva, C.E., Gomes, A.L.V., Abath, F.G.C., Brito, C.A.A., Cordeiro, M.T., Silva, A.M., Magalhães, C., Andrade, R., et al. (2009). Gene expression profiling during early acute febrile stage of dengue infection can predict the disease outcome. PLoS One *4*, e7892.

61. Pankla, R., Buddhisa, S., Berry, M., Blankenship, D.M., Bancroft, G.J., Banchereau, J., Lertmemongkolchai, G., and Chaussabel, D. (2009). Genomic transcriptional profiling identifies a candidate blood biomarker signature for the diagnosis of septicemic melioidosis. Genome Biol. *10*, R127.

62. Popper, S.J., Strouts, F.R., Lindow, J.C., Cheng, H.K., Montoya, M., Balmaseda, A., Durbin, A.P., Whitehead, S.S., Harris, E., Kirkpatrick, B.D., and Relman, D.A. (2018). Early transcriptional responses after dengue Vaccination Mirror the response to natural infection and predict Neutralizing Antibody Titers. J. Infect. Dis. *218*, 1911–1921.

63. Simmons, C.P., Popper, S., Dolocek, C., Chau, T.N.B., Griffiths, M., Dung, N.T.P., Long, T.H., Hoang, D.M., Chau, N.V., Thao, L.T.T., et al. (2007). Patterns of host genome-wide gene transcript abundance in the peripheral blood of patients with acute dengue hemorrhagic fever. J. Infect. Dis. *195*, 1097–1107.

64. Smith, C.L., Dickinson, P., Forster, T., Craigon, M., Ross, A., Khondoker, M.R., France, R., Ivens, A., Lynn, D.J., Orme, J., et al. (2014). Identification of a human neonatal immune-metabolic network associated with bacterial infection. Nat. Commun. *5*, 4649.

65. Sutherland, A., Thomas, M., Brandon, R.A., Brandon, R.B., Lipman, J., Tang, B., McLean, A., Pascoe, R., Price, G., Nguyen, T., et al. (2011). Development and validation of a novel molecular biomarker diagnostic test for the early detection of sepsis. Crit. Care *15*, R149.

66. Tantibhedhyangkul, W., Prachason, T., Waywa, D., El Filali, A., Ghigo, E., Thongnoppakhun, W., Raoult, D., Suputtamongkol, Y., Capo, C., Limwongse, C., and Mege, J.L. (2011). Orientia tsutsugamushi stimulates an original gene expression program in monocytes: relationship with gene expression in patients with scrub typhus. PLoS Negl. Trop. Dis. *5*, e1028.

67. Thuny, F., Textoris, J., Amara, A.B., Filali, A.E., Capo, C., Habib, G., Raoult, D., and Mege, J.L. (2012). The gene expression analysis of blood reveals S100A11 and AQP9 as potential biomarkers of infective endocarditis. PLoS One *7*, e31490.

68. van de Weg, C.A.M., van den Ham, H.J., Bijl, M.A., Anfasa, F., Zaaraoui-Boutahar, F., Dewi, B.E., Nainggolan, L., van IJcken, W.F.J., Osterhaus, A.D.M.E., Martina, B.E.E., et al. (2015). Time since onset of disease and individual clinical markers associate with transcriptional changes in uncomplicated dengue. PLoS Negl. Trop. Dis. *9*, e0003522.

69. Wong, H.R., Shanley, T.P., Sakthivel, B., Cvijanovich, N., Lin, R., Allen, G.L., Thomas, N.J., Doctor, A., Kalyanaraman, M., Tofil, N.M., et al. (2007). Genome-level expression profiles in pediatric septic shock indicate a role for altered zinc homeostasis in poor outcome. Physiol. Genomics *30*, 146–155.

70. Zhai, Y., Franco, L.M., Atmar, R.L., Quarles, J.M., Arden, N., Bucasas, K.L., Wells, J.M., Niño, D., Wang, X., Zapata, G.E., et al. (2015). Host transcriptional response to influenza and other acute respiratory viral infections – a prospective cohort study. PLoS Pathog. *11*, e1004869.

71. Mahajan, P., Kuppermann, N., Mejias, A., Suarez, N., Chaussabel, D., Casper, T.C., Smith, B., Alpern, E.R., Anders, J., Atabaki, S.M., et al. (2016). Association of RNA Biosignatures with bacterial infections in febrile infants aged 60 Days or younger. JAMA *316*, 846–857.

72. Parnell, G., McLean, A., Booth, D., Huang, S., Nalos, M., and Tang, B. (2011). Aberrant cell cycle and apoptotic changes characterise severe influenza a infection - a meta-analysis of genomic signatures in circulating leukocytes. PLoS One *6*, e17186.

73. Ramilo, O., Allman, W., Chung, W., Mejias, A., Ardura, M., Glaser, C., Wittkowski, K.M., Piqueras, B., Banchereau, J., Palucka, A.K., and Chaussabel, D. (2007). Gene expression patterns in blood leukocytes discriminate patients with acute infections. Blood *109*, 2066–2077.

74. Sweeney, T.E., Wong, H.R., and Khatri, P. (2016). Robust classification of bacterial and viral infections via integrated host gene expression diagnostics. Sci. Transl. Med. *8*, 346ra91.

75. Sampson, D.L., Fox, B.A., Yager, T.D., Bhide, S., Cermelli, S., McHugh, L.C., Seldon, T.A., Brandon, R.A., Sullivan, E., Zimmerman, J.J., et al. (2017). A four-biomarker blood signature discriminates systemic inflammation due to viral infection versus other Etiologies. Sci. Rep. *7*, 2914.

76. Tsalik, E.L., Henao, R., Nichols, M., Burke, T., Ko, E.R., McClain, M.T., Hudson, L.L., Mazur, A., Freeman, D.H., Veldman, T., et al. (2016). Host gene expression classifiers diagnose acute respiratory illness etiology. Sci. Transl. Med. *8*, 322ra11.

77. Warsinske, H., Vashisht, R., and Khatri, P. (2019). Host-response-based gene signatures for tuberculosis diagnosis: a systematic comparison of 16 signatures. PLoS Med. *16*, e1002786.

78. Warsinske, H.C., Rao, A.M., Moreira, F.M.F., Santos, P.C.P., Liu, A.B., Scott, M., Malherbe, S.T., Ronacher, K., Walzl, G., Winter, J., et al. (2018). Assessment of Validity of a blood-based 3-gene signature score for progression and diagnosis of tuberculosis, disease severity, and treatment response. JAMA Netw. Open *1*, e183779.

79. Philipp Doebler, H.H., Holling, H., and Sousa-Pinto, B. (2013). Meta-Analysis of Diagnostic Accuracy with Mada.

80. Södersten, E., Ongarello, S., Mantsoki, A., Wyss, R., Persing, D.H., Banderby, S., et al. (2020). Diagnostic accuracy study of a novel blood-based assay for identification of tuberculosis in people living with HIV. J. Clin. Microbiol. *59*.

81. Zimmer, A.J., Schumacher, S.G., Södersten, E., Mantsoki, A., Wyss, R., Persing, D.H., Banderby, S., Strömqvist Meuzelaar, L., Prieto, J., Gnanashanmugam, D., et al. (2021). A novel blood-based assay for treatment monitoring of tuberculosis. BMC Res. Notes *14*, 247.

82. Moreira, F.M.F., Verma, R., Pereira Dos Santos, P.C., Leite, A., da Silva Santos, A., de Araujo, R.C.P., da Silva, B.O., de Sá Queiroz, J.H.F., Persing, D.H., Södersten, E., et al. (2021). Blood-based host biomarker diagnostics in active case finding for pulmonary tuberculosis: a diagnostic case-control study. EClinicalMedicine *33*, 100776.

83. Sutherland, J.S., Spuy, G van der, Gindeh, A., Thuong, N.T., Namuganga, A.R., Owolabi, O., et al. (2021). Diagnostic accuracy of the Cepheid 3-gene host response fingerstick blood test in a prospective, multi-site study: interim results. Clin. Infect. Dis.. ciab839-.

84. Bongen, E., Lucian, H., Khatri, A., Fragiadakis, G.K., Bjornson, Z.B., Nolan, G.P., Utz, P.J., and Khatri, P. (2019). Sex differences in the blood transcriptome identify robust changes in immune cell proportions with aging and influenza infection. Cell Rep. *29*, 1961–1973.e4.

85. Tukiainen, T., Villani, A.C., Yen, A., Rivas, M.A., Marshall, J.L., Satija, R., Aguirre, M., Gauthier, L., Fleharty, M., Kirby, A., et al. (2017). Landscape of X chromosome inactivation across human tissues. Nature *550*, 244–248.

86. GBD 2019 Diseases and Injuries Collaborators (2020). Global burden of 369 diseases and injuries in 204 countries and territories, 1990–2019: a systematic analysis for the Global Burden of Disease Study 2019. Lancet *396*, 1204–1222.

87. Plosa, E.J., Esbenshade, J.C., Fuller, M.P., and Weitkamp, J.H. (2012). Cytomegalovirus infection. Pediatr. Rev. *33*, 156–163. quiz 163.

88. Zhou, W., Lin, F., Teng, L., Li, H., Hou, J., Tong, R., Zheng, C., Lou, Y., and Tan, W. (2013). Prevalence of herpes and respiratory viruses in induced sputum among hospitalized children with non typical bacterial community-acquired pneumonia. PLoS One *8*, 79477.

89. Wacker, C., Prkno, A., Brunkhorst, F.M., and Schlattmann, P. (2013). Procalcitonin as a diagnostic marker for sepsis: a systematic review and meta-analysis. Lancet Infect. Dis. *13*, 426–435.

90. Ducharme, J., Self, W.H., Osborn, T.M., Ledeboer, N.A., Romanowsky, J., Sweeney, T.E., Liesenfeld, O., and Rothman, R.E. (2020). A multi-mRNA host-response molecular blood test for the diagnosis and prognosis of acute infections and sepsis: Proceedings from a clinical advisory Panel. J. Pers. Med. *10*, 266.

91. Suzuki, K., Ohkuma, M., and Nagaoka, I. (2019). Bacterial lipopolysaccharide and antimicrobial LL-37 enhance ICAM-1 expression and NF-kappaB p65 phosphorylation in senescent endothelial cells. Int. J. Mol. Med. *44*, 1187–1196.

92. Sambarey, A., Devaprasad, A., Mohan, A., Ahmed, A., Nayak, S., Swaminathan, S., D'Souza, G., Jesuraj, A., Dhar, C., Babu, S., et al. (2017). Unbiased identification of blood-based biomarkers for pulmonary tuberculosis by modeling and mining molecular Interaction networks. EBioMedicine *15*, 112–126.

93. Zheng, R., Liu, H., Song, P., Feng, Y., Qin, L., Huang, X., Chen, J., Yang, H., Liu, Z., Cui, Z., et al. (2015). Epstein-Barr virus-induced gene 3 (EBI3) polymorphisms and expression are associated with susceptibility to pulmonary tuberculosis. Tuberculosis *95*, 497–504.

94. Tolfvenstam, T., Lindblom, A., Schreiber, M.J., Ling, L., Chow, A., Ooi, E.E., and Hibberd, M.L. (2011). Characterization of early host responses in adults with dengue disease. BMC Infect. Dis. *11*, 209.

95. Li, L., Ni, Y.A., Song, Z., Yi, Z., and Wang, F. (2021). Identification of pathogenic genes and transcription factors in respiratory syncytial virus. BMC Pediatr. *21*, 27.
96. Turner, C.T., et al. (2020). Blood transcriptional profiling in a prospective observational cohort of South African adults presenting for investigation of possible pulmonary tuberculosis. BioStudies. E-MTAB-8290.
97. Eckold, C., Kumar, V., Weiner, J., Alisjahbana, B., Riza, A.L., Ronacher, K., Coronel, J., Kerry-Barnard, S., Malherbe, S.T., Kleynhans, L., et al. (2021). Impact of Intermediate Hyperglycemia and Diabetes on immune Dysfunction in tuberculosis. Clin. Infect. Dis. *72*, 69–78.
98. Singhania, A., Verma, R., Graham, C.M., Lee, J., Tran, T., Richardson, M., Lecine, P., Leissner, P., Berry, M.P.R., Wilkinson, R.J., et al. (2018). A modular transcriptional signature identifies phenotypic heterogeneity of human tuberculosis infection. Nat. Commun. *9*, 2308.
99. Pollard, A., and Blohmke, C. (2015). Whole blood transcriptome in humans experimentally infected with Salmonella Typhi. BioStudies. E-MTAB-3423.
100. Schreiber, F. (2010). Transcription profiling of diabetic vs non-diabetic human blood cells infected with Burkholderia pseudomallei. BioStudies. E-TABM-852.
101. Sweeney, T.E., Shidham, A., Wong, H.R., and Khatri, P. (2015). A comprehensive time-course-based multicohort analysis of sepsis and sterile inflammation reveals a robust diagnostic gene set. Sci. Transl. Med. *7*, 287ra71.
102. van den Ham, H.J. (2015). Time since onset of disease and individual clinical markers associate with transcriptional changes in uncomplicated dengue. BioStudies. E-MTAB-3162.
103. Andeweg, A. (2016). Blood mRNA profiling to support prognosis of disease severity in respiratory syncytial virus infected infants. BioStudies. E-MTAB-5195.
104. Liu, K., Chen, L., Kaur, R., and Pichichero, M. (2012). Transcriptome signature in young children with acute otitis media due to Streptococcus pneumoniae. Microbes Infect. *14*, 600–609.
105. Bloom, C.I., Graham, C.M., Berry, M.P.R., Rozakeas, F., Redford, P.S., Wang, Y., Xu, Z., Wilkinson, K.A., Wilkinson, R.J., Kendrick, Y., et al. (2013). Transcriptional blood signatures distinguish pulmonary tuberculosis, pulmonary sarcoidosis, pneumonias and lung cancers. PLoS One *8*, e70630.
106. Maertzdorf, J., Ota, M., Repsilber, D., Mollenkopf, H.J., Weiner, J., Hill, P.C., and Kaufmann, S.H.E. (2011). Functional correlations of pathogenesis-driven gene expression signatures in tuberculosis. PLoS One *6*, e26938.
107. Maertzdorf, J., Weiner, J., 3rd, Mollenkopf, H.J., TBornotTB Network; Bauer, T., Prasse, A., Müller-Quernheim, J., and Kaufmann, S.H.E. (2012). Common patterns and disease-related signatures in tuberculosis and sarcoidosis. Proc. Natl. Acad. Sci. USA *109*, 7853–7858.
108. Verhagen, L.M., Zomer, A., Maes, M., Villalba, J.A., Del Nogal, B., Eleveld, M., van Hijum, S.A., de Waard, J.H., and Hermans, P.W. (2013). A predictive signature gene set for discriminating active from latent tuberculosis in Warao Amerindian children. BMC Genom. *14*, 74.
109. Blankley, S., Graham, C.M., Turner, J., Berry, M.P.R., Bloom, C.I., Xu, Z., Pascual, V., Banchereau, J., Chaussabel, D., Breen, R., et al. (2016). The transcriptional signature of active tuberculosis reflects symptom Status in extra-pulmonary and pulmonary tuberculosis. PLoS One *11*, e0162220.
110. Maertzdorf, J., Repsilber, D., Walzl, G., Mollenkopf, H., and Kaufmann, S. (2010). Tuberculosis patients, latent and uninfected controls; whole blood. BioStudies. E-GEOD-25534.
111. Noursadeghi, M. (2016). Whole blood transcriptional profiling of healthy volunteers and patients with active tuberculosis. BioStudies. E-MTAB-3260.
112. Rinchai, D., Altman, M.C., Konza, O., Hässler, S., Martina, F., Toufiq, M., Garand, M., Kabeer, B.S.A., Palucka, K., Mejias, A., et al. (2020). Definition of erythroid cell-positive blood transcriptome phenotypes associated with severe respiratory syncytial virus infection. Clin. Transl. Med. *10*, e244.
113. Park, J., Munagala, I., Xu, H., Blankenship, D., Maffucci, P., Chaussabel, D., Banchereau, J., Pascual, V., and Cunningham-Rundles, C. (2013). Interferon signature in the blood in inflammatory common variable immune deficiency. PLoS One *8*, e74893.
114. Montano, M., Rarick, M., Sebastiani, P., Brinkmann, P., Russell, M., Navis, A., Wester, C., Thior, I., and Essex, M. (2006). Gene-expression profiling of HIV-1 infection and perinatal transmission in Botswana. Genes Immun. *7*, 298–309.
115. Singh, S., Toor, J.S., Sharma, A., and Arora, S.K. (2020). Signature genes associated with immunological non-responsiveness to anti-retroviral therapy in HIV-1 subtype-c infection. PLoS One *15*, e0234270.
116. R Core Team (2020).
117. Wu, J., and Irizarry, R. (2020). Gcrma: Background Adjustment Using Sequence Information. (Bioconductor).
118. Love, M.I., Huber, W., and Anders, S. (2014). Moderated estimation of fold change and dispersion for RNA-seq data with DESeq2. Genome Biol. *15*, 550–621.
119. Haynes, W.A., Vallania, F., Liu, C., Bongen, E., Tomczak, A., Andres-Terrè, M., Lofgren, S., Tam, A., Deisseroth, C.A., Li, M.D., et al. (2017). Empowering multi-cohort gene expression analysis to increase reproducibility. Pac. Symp. Biocomput. *22*, 144–153.
120. Sing, T., Sander, O., Beerenwinkel, N., and Lengauer, T. (2005). ROCR: visualizing classifier performance in R. Bioinformatics *21*, 3940–3941.
121. Lumley, T. (2003). Rmeta Version 3.0. R Package.
122. López-Ratón, M., Rodríguez-Álvarez, M.X., Suárez, C.C., and Sampedro, F.G. (2014). OptimalCutpoints: an R package for selecting optimal Cutpoints in diagnostic tests. J. Stat. Software *61*, 1–36.
123. Friedman, J., Hastie, T., and Tibshirani, R. (2010). Regularization Paths for generalized linear models via Coordinate descent. J. Stat. Softw. *33*, 1–22.
124. Tusher, V.G., Tibshirani, R., and Chu, G. (2001). Significance analysis of microarrays applied to the ionizing radiation response. Proc. Natl. Acad. Sci. USA *98*, 5116–5121.
125. Phetsouvanh, R., Phongmany, S., Soukaloun, D., Rasachak, B., Soukhaseum, V., Soukhaseum, S., Frichithavong, K., Khounnorath, S., Pengdee, B., Phiasakha, K., et al. (2006). Causes of community-acquired bacteremia and patterns of antimicrobial resistance in Vientiane, Laos. Am. J. Trop. Med. Hyg. *75*, 978–985.
126. Nielsen, A.C.Y., Böttiger, B., Midgley, S.E., and Nielsen, L.P. (2013). A novel enterovirus and parechovirus multiplex one-step real-time PCR-validation and clinical experience. J. Virol. Methods *193*, 359–363.
127. Jia, J., Ma, Y., Zhao, X., Guo, Y., Huangfu, C., Fang, C., Fan, R., Lv, M., Yin, H., and Zhang, J. (2015). Prevalence of human parvovirus B19 in Chinese plasma pools for manufacturing plasma derivatives. Virol. J. *12*, 162.
128. Binnicker, M.J., and Espy, M.E. (2013). Comparison of six real-time PCR assays for qualitative detection of cytomegalovirus in clinical specimens. J. Clin. Microbiol. *51*, 3749–3752.
129. Riediger, I.N., Stoddard, R.A., Ribeiro, G.S., Nakatani, S.M., Moreira, S.D.R., Skraba, I., Biondo, A.W., Reis, M.G., Hoffmaster, A.R., Vinetz, J.M., et al. (2017). Rapid, actionable diagnosis of urban epidemic leptospirosis using a pathogenic Leptospira lipL32-based real-time PCR assay. PLoS Negl. Trop. Dis. *11*, e0005940.
130. Henry, K.M., Jiang, J., Rozmajzl, P.J., Azad, A.F., Macaluso, K.R., and Richards, A.L. (2007). Development of quantitative real-time PCR assays to detect Rickettsia typhi and Rickettsia felis, the causative agents of murine typhus and flea-borne spotted fever. Mol. Cell. Probes *21*, 17–23.
131. Jiang, J., Chan, T.C., Temenak, J.J., Dasch, G.A., Ching, W.M., and Richards, A.L. (2004). Development of a quantitative real-time polymerase

chain reaction assay specific for Orientia tsutsugamushi. Am. J. Trop. Med. Hyg. *70*, 351–356.

132. Locatelli, G., Santoro, F., Veglia, F., Gobbi, A., Lusso, P., and Malnati, M.S. (2000). Real-time quantitative PCR for human herpesvirus 6 DNA. J. Clin. Microbiol. *38*, 4042–4048.
133. Johnson, W.E., Fau, L.C., and Rabinovic, A. (2006). Adjusting batch effects in microarray expression data using empirical Bayes methods. Biostat. TA - Biostat.
134. Bodkin, N., Ross, M., McClain, M.T., Ko, E.R., Woods, C.W., Ginsburg, G.S., Henao, R., and Tsalik, E.L. (2022). Systematic comparison of published host gene expression signatures for bacterial/viral discrimination. Genome Med. *14*, 18.
135. Mayhew, M.B., Buturovic, L., Luethy, R., Midic, U., Moore, A.R., Roque, J.A., Shaller, B.D., Asuni, T., Rawling, D., Remmel, M., et al. (2020). A generalizable 29-mRNA neural-network classifier for acute bacterial and viral infections. Nat. Commun. *11*, 1177.
136. Kaforou, M., Wright, V.J., Oni, T., French, N., Anderson, S.T., Bangani, N., Banwell, C.M., Brent, A.J., Crampin, A.C., Dockrell, H.M., et al. (2013). Detection of tuberculosis in HIV-infected and -uninfected African adults using whole blood RNA expression signatures: a case-control study. PLoS Med. *10*, e1001538.
137. Tibshirani, R. (1996). Regression shrinkage and selection via the Lasso. J. Roy. Stat. Soc. B *58*, 267–288.
138. Benjamini, Y., and Hochberg, Y. (1995). Controlling the false discovery rate: a practical and powerful approach to multiple testing. J. Roy. Stat. Soc. B *57*, 289–300.
139. Doncaster, C.P., and Spake, R. (2018). Correction for bias in meta-analysis of little-replicated studies. Methods Ecol. Evol. *9*, 634–644.
140. Hedges, L.V. (1982). Estimation of effect size from a series of independent experiments. Psychol. Bull. *92*, 490–499.
141. Hedges, L.V. (1983). A random effects model for effect sizes. Psychol. Bull. *93*, 388–395.
142. Arlot, S., and Celisse, A. (2010). A survey of cross-validation procedures for model selection. Stat. Surv. *4*, 40–79.
143. Stone, M. (1974). Cross-validatory Choice and assessment of statistical Predictions. J. Roy. Stat. Soc. B *36*, 111–133.
144. Kester, A.D., and Buntinx, F. (2000). Meta-analysis of ROC curves. Med. Decis. Making *20*, 430–439.
145. Shapiro, D.E. (1999). The interpretation of diagnostic tests. Stat. Methods Med. Res. *8*, 113–134.
146. Youden, W.J. (1950). Index for rating diagnostic tests. Cancer *3*, 32–35.
147. Edwards, D. (2003). Non-linear normalization and background correction in one-channel cDNA microarray studies. Bioinformatics *19*, 825–833.

CellPress
OPEN ACCESS

STAR★METHODS

KEY RESOURCES TABLE

REAGENT or RESOURCE	SOURCE	IDENTIFIER
Deposited data		
RNA-seq dataset	Turner et al.[96]	ArrayExpress: E-MTAB-8290
RNA-seq dataset	Eckold et al.[97]	GEO: GSE114192
RNA-seq dataset	Sambarey et al.[92]	GEO: GSE122485
RNA-seq dataset	Singhania et al.[98]	GEO: GSE107995
RNA-seq dataset	N/A (no associated paper)	GEO: GSE69597
Microarray dataset	N/A (no associated paper)	GEO: GSE206829
Microarray dataset	Mahajan et al.[71]	GEO: GSE64456
Microarray dataset	Lindow et al.[44]	GEO: GSE72946
Microarray dataset	Pollard and Blohmke[99]	GEO: E-MTAB-3423
Microarray dataset	Schreiber[100]	ArrayExpress: E-MTAB-852
Microarray dataset	Simmons et al.[63]	GEO: GSE40628
Microarray dataset	de Steenhuijsen Piters et al.[39]	GEO: GSE77087
Microarray dataset	Wong et al.[69]	GEO: GSE4607
Microarray dataset	Hu et al.[54]	GEO: GSE40396
Microarray dataset	Herberg et al.[41]	GEO: GSE42026
Microarray dataset	Bermejo-Martin et al.[50]	GEO: GSE21802
Microarray dataset	Zhai et al.[70]	GEO: GSE68310
Microarray dataset	Parnell et al.[72]	GEO: GSE20346
Microarray dataset	Parnell et al.[36]	GEO: GSE40012
Microarray dataset	Sweeney et al.[101]	GEO: GSE66099
Microarray dataset	Suarez et al.[15]	GEO: GSE60244
Microarray dataset	N/A (no associated paper)	GEO: GSE11755
Microarray dataset	Sutherland et al.[65]	GEO: GSE28750
Microarray dataset	Thuny et al.[67]	GEO: GSE29161
Microarray dataset	Ahn et al.[49]	GEO: GSE33341
Microarray dataset	Lill et al.[59]	GEO: GSE40586
Microarray dataset	Pankla et al.[61]	GEO: GSE69528
Microarray dataset	van den Ham[102]	ArrayExpress: E-MTAB-3162
Microarray dataset	Smith et al.[64]	GEO: GSE25504
Microarray dataset	Pankla et al.[61]	GEO: GSE13015
Microarray dataset	Mejias et al.[46]	GEO: GSE38900
Microarray dataset	Heinonen et al.[40]	GEO: GSE67059
Microarray dataset	Thompson et al.[24]	GEO: GSE7000
Microarray dataset	Kwissa et al.[58]	GEO: GSE51808
Microarray dataset	Irwin et al.[55]	ArrayExpress: E-MEXP-3567
Microarray dataset	Zaas et al.[16]	GEO: GSE17156
Microarray dataset	Rodriguez-Fernandez et al.[47]	GEO: GSE103842
Microarray dataset	Banchereau et al.[37]	GEO: GSE30119
Microarray dataset	Popper et al.[62]	GEO: GSE96656
Microarray dataset	Kulohoma et al.[57]	GEO: GSE47172
Microarray dataset	Herberg et al.[14]	GEO: GSE80496
Microarray dataset	Tang et al.[48]	GEO: GSE82050
Microarray dataset	Andeweg[103]	ArrayExpress: E-MTAB-5195

(*Continued on next page*)

Continued		
REAGENT or RESOURCE	SOURCE	IDENTIFIER
Microarray dataset	Liu et al.[45]	GEO: GSE73072
Microarray dataset	Cvijanovich et al.[53]	GEO: GSE9692
Microarray dataset	Berry et al.[51]	GEO: GSE19491
Microarray dataset	Jaggi et al.[56]	GEO: GSE68004
Microarray dataset	Berdal et al.[38]	GEO: GSE27131
Microarray dataset	Popper et al.[33]	GEO: GSE38246
Microarray dataset	Nascimento et al.[60]	GEO: GSE18090
Microarray dataset	Ramilo et al.[73]	GEO: GSE6269
Microarray dataset	Ioannidis et al.[42]	GEO: GSE34205
Microarray dataset	Liu et al.[104]	GEO: GSE23140
Microarray dataset	Ardura et al.[35]	GEO: GSE16129
Microarray dataset	Tantibhedhyangkul et al.[66]	GEO: GSE16463
Microarray dataset	Chaussabel et al.[52]	GEO: GSE11907
Microarray dataset	Herberg et al.[14]	GEO: GSE72809
Microarray dataset	Herberg et al.[14]	GEO: GSE72810
Microarray dataset	Tsalik et al.[76]	GEO: GSE63990
Microarray dataset	Bloom et al.[105]	GEO: GSE42834
Microarray dataset	Maertzdorf et al.[106]	GEO: GSE28623
Microarray dataset	Maertzdorf et al.[107]	GEO: GSE34608
Microarray dataset	Verhagen et al.[108]	GEO: GSE41055
Microarray dataset	Blankley et al.[109]	GEO: GSE83456
Microarray dataset	N/A (no associated paper)	GEO: GSE81746
Microarray dataset	Maertzdorf et al.[110]	ArrayExpress: E-GEOD-25534
Microarray dataset	Noursadeghi et al.[111]	ArrayExpress: E-MTAB-3260
Microarray dataset	Rinchai et al.[112]	GEO: GSE100150
Microarray dataset	N/A (no associated paper)	GEO: GSE29429
Microarray dataset	Park et al.[113]	GEO: GSE29536
Microarray dataset	Montano et al.[114]	GEO: GSE4124
Microarray dataset	Singh et al.[115]	GEO: GSE77939
Oligonucleotides		
Taqman gene expression assays	See Table S12	See Table S12
Software and algorithms		
R	R Core Team[116]	https://www.r-project.org/
gcRMA	Wu and Irizarry[117]	https://www.bioconductor.org/packages/release/bioc/html/gcrma.html
DESeq2	Love et al.[118]	https://bioconductor.org/packages/release/bioc/html/DESeq2.html
MetaIntegrator	Haynes et al.[119]	https://cran.r-project.org/web/packages/MetaIntegrator/index.html
COCONUT	Sweeney et al.[74]	https://cran.r-project.org/web/packages/COCONUT/index.html
ROCR	Sing et al.[120]	https://cran.r-project.org/web/packages/ROCR/index.html
rmeta	Lumley[121]	https://cran.r-project.org/web/packages/rmeta/index.html
OptimalCutpoints	López-Ratón et al.[122]	https://cran.r-project.org/web/packages/OptimalCutpoints/index.html
glmnet	Friedman et al.[123]	https://cran.r-project.org/web/packages/glmnet/index.html
samr	Tusher et al.[124]	https://cran.r-project.org/web/packages/samr/index.html

CellPress
OPEN ACCESS

RESOURCE AVAILABILITY

Lead contact

Further information and requests for resources, software, and data should be directed to and will be fulfilled by the lead contact, Purvesh Khatri (pkhatri@stanford.edu).

Materials availability

This study did not generate new unique reagents.

Data and code availability

- All datasets, software, and algorithms used in this study are publicly available and listed in the Key resources table.
- This paper did not generate any unique datasets or code.
- Any additional information required to reanalyze the data reported in this paper is available from the lead contact upon request.

EXPERIMENTAL MODEL AND SUBJECT DETAILS

Search for gene expression datasets

We performed a search in NIH Gene Expression Omnibus (GEO) and European Bioinformatics Institute (EBI) ArrayExpress for public human microarray genome-wide expression studies of bacterial or viral infections.[31,32] We excluded datasets that (i) were nonclinical, (ii) were profiled using tissues other than WB or PBMCs, (iii) did not have samples from at least 3 healthy controls, or (iv) did not provide information to identify whether a patient had bacterial or viral infection.

We renormalized Affymetrix arrays using GC robust multiarray average (gcRMA) (on arrays with mismatch probes) or RMA. We renormalized Illumina, Agilent, GE, and other commercial arrays using normal-exponential background correction followed by quantile normalization. We did not renormalize the custom arrays. After $\log_2$-transformation of normalized expression data, we used a fixed-effect model to summarize probes to genes within each study. Within each study, cohorts assayed with different microarray types were treated as independent datasets.

Laos cohorts

Patients were recruited at Mahosot Hospital, Vientiane, Laos between July 2007 and March 2009 as part of a study of the etiology of infectious febrile illness.[125] All adults (>15 years of age) with tympanic temperature >37.5°C and suspected septicemia were enrolled if they provided written informed consent. Ethical clearance was granted by the former Faculty of Medical Sciences Ethical Review Committee (now University of Health Sciences Ethics Committee), National University of Laos, the Oxford University Tropical Ethics Committee, and the Stanford University Institutional Review Board.

Venous blood samples were drawn for blood culture and for other diagnostic tests. Positive blood cultures were identified as described in Phetsouvanh et al.[125] Targeted PCR tests were performed for specific pathogens known to be of importance in this hospital population, including *O. tsutsugamushi, Rickettsia* spp., *Leptospira* spp., *dengue virus*, and *Japanese encephalitis virus* (Mayxay et al.[2]). A positive case was defined as CT below 40 (single sample) with appropriate results for controls. Patients without any positive diagnosis were excluded from the analysis. Whole blood for RNA was collected into PAXgene tubes, stored at room temperature for 2 h and then stored at 4°C for up to four days. RNA was extracted using Qiagen PAXgene Blood RNA kit according to manufacturer's direction, stored at −80°C, and shipped to Stanford University on dry ice.

Nepal cohort

We recruited all individuals >12 months of age presenting to Dhulikhel Hospital, Kathmandu University Hospital, Kavrepalanchok, Nepal with a self-reported >72-h history of fever. Participants were recruited from outpatient departments, emergency department, and inpatient wards. After obtaining informed consent, we administered a standardized questionnaire to each participant or adult guardian to ascertain demographic and clinical characteristics. Ethical clearance was granted by the Nepal Health Research Council and the Stanford University Institutional Review Board. A venous blood specimen was collected from each participant and transferred into PAXgene RNA tubes, EDTA tubes, and Bactec blood culture bottles. Blood culture was performed using a Bactec automated culture system, and bacterial identification was performed using biochemical testing.[5]

We performed real-time PCR for Rickettsia, Leptospira, HHV6, CMV, Parvovirus B19, and enteroviruses using previously published protocols.[126–132] Patients without any positive diagnosis were excluded from analysis.

METHOD DETAILS

Methods for signature discovery and validation, including co-normalization using COCONUT, identifying 8-gene signature using a multi-cohort analysis framework, and a classifier are described below.

QUANTIFICATION AND STATISTICAL ANALYSIS

COCONUT co-normalization

Despite availability of 64 independent datasets that profiled respiratory infections, there were only three cohorts that contained intracellular bacterial, extracellular bacterial, and viral infections. Furthermore, two of these datasets had ≤ 2 intracellular bacterial infections. This made it virtually impossible to conduct within-dataset analyses, so we instead decided to perform an analysis across all the datasets. However, because of the difference in background measurements for each dataset (owing to the use of different platforms), we were concerned about skewed results due to the batch effects. In order to make use of these data, we used Combat CO-Normalization Using conTrols or COCONUT.[74] ComBat only assumes that the data have been normalized and expression values have been estimated for all genes and samples. Prior to running COCONUT, we ensured that all datasets were normalized as described above in the subsection search for gene expression datasets. COCONUT allows for co-normalization of expression data without changing the distribution of genes between studies and without any bias towards sample diagnosis. It applies a modified version of the ComBat empirical Bayes normalization method[133] that assumes an equal distribution only between control samples. Briefly, the healthy controls from each cohort undergo ComBat co-normalization without covariates, and the ComBat estimated parameters are acquired for each dataset's healthy samples. These parameters are then applied to the disease samples in each dataset, which causes all samples to assume the same background distribution while still retaining within-dataset differences between healthy and disease samples in each dataset. We have previously shown that when COCONUT co-normalization is applied, housekeeping genes remain invariant across diseases and cohorts, and each gene retains the same distribution between diseases and controls within each dataset.[74]

When validating published signatures (see below), we co-normalized all 64 datasets and performed analyses across all datasets. However, for signature discovery the data were randomly assigned to one of three groups (training, hold-out validation, or independent validation; described below) and each group was independently batch-corrected using COCONUT co-normalization. Additional statistical details can be found in Figure 2.

Validation of published signatures

We chose 4 signatures to investigate whether they generalize to global patient population or not based on a recently described systematic comparison of 28 host gene expression signatures, following PRISMA guidelines, for discrimination of bacterial and viral infections using 51 datasets by Bodkin et al.[134] Only 7 out of the 51 datasets used by Bodkin et al. were from low-and-middle income countries (LMICs), 3 of which included only patients with viral infections. In other words, Bodkin et al. did not have sufficient datasets (only 4 out of 51 datasets from LMICs) to investigate whether these 28 signatures are generalizable to global population or not.

Given the comprehensive analysis by Bodkin et al., we chose signatures that had high accuracy for diagnosis of bacterial infections according to Bodkin et al. We reasoned that if a signature with higher accuracy for bacterial infection in Western Europe and North America (non-LMICs) does not generalize to LMICs, a signature with lower accuracy will not generalize either (because even if it has higher accuracy in LMICs, it has lower accuracy in non-LMICs). Bodkin et al. further found that signatures with smaller number of genes had lower accuracy than those with higher number of genes. Therefore, we selected a set of signatures with a varying number of genes with high accuracy to test our hypothesis.

Finally, we prioritized signatures that are under active development for translation to clinical practice to demonstrate that none of them will address the global health challenge of discriminating bacterial vs viral infections. To the best of our knowledge, the signatures under active development are Herberg2, Sweeney7, Mayhew29[135] and Tsalik120. Among these, Mayhew29 includes all genes from Sweeney7 and had only 1% higher AUROC.

We also excluded signatures from comparison if (i) the underlying model could not be recreated, either because the training data were inaccessible or because not enough details about the training procedure were available; (ii) the model was unable to be applied to a new dataset without re-training (e.g., *kNN*-based classifiers); or (iii) the model did not produce a continuous score. For Sweeney7, we used a previously described bacterial/viral metascore to perform classification (Equation 1). For Herberg2 and Sampson4, we used the described Disease Risk Score[136] to perform classification (Equation 2). For Tsalik120, we recreated their Least Absolute Shrinkage and Selection Operator (LASSO) models[137] using the provided coefficients and then calculated the score as the ratio of the linear outputs of the bacterial and viral LASSOs (Equation 3). We removed two predictors from the Tsalik120 model because they had no existing mapping to a gene symbol and thus could not be used in any dataset that was not run with the original microarray platform. Since COCONUT requires genes to be measured in all datasets, for each signature we removed any datasets that would cause a significant number of signature genes to be excluded. In the COCONUT co-normalized data, no signatures were missing any genes other than Tsalik120, which was missing 17/120 genes. In the Laos Microarray cohort, Tsalik120 was missing two genes and Sampson4 was missing one gene.

To ensure that the decreased accuracy for intracellular bacterial infections that we observed for all four published signatures was not simply due to the specific subset of datasets that we chose for each signature, we also analyzed the difference in performance for different subsets of 64 datasets. Because there were too many combinations for an exhaustive analysis, we randomly sampled 1,396,677 combinations from all possible combinations. To reduce noise, we only included combinations where each infection group had at least five samples, at least one of the two AUROCs was greater than 0.6, and where at least half of the signature genes were present. Additionally, to ensure that this phenomenon was not driven by the number of genes present in Tsalik120, we also computed the difference in performance compared to the number of missing genes. Additional statistical details can be found in Figure 1.

$$S_i = \left(\prod_{gene \in pos} x_i(gene)\right)^{\frac{1}{|pos|}} - \left(\prod_{gene \in neg} x_i(gene)\right)^{\frac{1}{|neg|}} \cdot \frac{|neg|}{|pos|} \quad \text{(Equation 1)}$$

$$S_i = \left(\sum_{gene \in pos} x_i(gene)\right) - \left(\sum_{gene \in neg} x_i(gene)\right) \quad \text{(Equation 2)}$$

$$S_i = \frac{lasso_{bac}(i)}{lasso_{vir}(i)} \quad \text{(Equation 3)}$$

Derivation of the 8-gene signature with MANATEE

We developed Multi-cohort ANalysis of AggregaTed gEne Expression (MANATEE) to include biological, clinical, and technical heterogeneity representative of the global patient population across heterogeneous independent datasets. After collecting and curating relevant datasets from public repositories through a search, we selected a subset of those datasets for the discovery of a gene signature, referred to as "training data", and the rest were set aside for validation of the gene signature, referred to as "independent validation datasets." We randomly divided the training data such that 70% of the data were used for discovery of the gene signature and the remaining 30% were used as hold-out validation data. Both the training and hold-out data were independently batch-corrected using COCONUT.

We calculated four measures of differential expression between cases and controls for each gene using the training data: (1) the Significant Analysis of Microarray (SAM) score from SAM,[124] (2) the corresponding SAM local FDR, (3) the Benjamini-Hochberg FDR corrected p value from a t-test,[138] and (4) Hedge's adjusted g that accounts for small sample bias[139–141] as the effect size (ES).

To ensure that resulting signatures are generalizable and not affected by unknown confounders that may be present in individual datasets, we performed a leave-one-study-out (LOSO) analysis, wherein we iteratively removed each study that accounted for at least 5% of the training samples from the training set and re-calculated each of the four differential expression statistics for each iteration of the training set. This LOSO iterative analysis ensured that any gene selected to be a part of the signature met the selection criteria irrespective of which study was removed. This strategy prevented a single study from exerting too strong of an effect on the selection of genes.[142,143] We have recently showed that classification signatures identified using LOSO tend to be less overfit than signatures identified using k-fold cross validation.[135]

Next, we applied three different differential expression thresholds: 1) ES $\geq$ 0.6 & FDR $\leq$1%; 2) ES $\geq$ 0.8 & FDR $\leq$1%; and 3) top 100 SAM scores. For the SAM score threshold, all genes with a local SAM FDR $\leq$1% were ranked by their SAM score, and the top 100 genes from that list were selected. After applying the differential expression filters, several feature selection methods could be used to further narrow down which genes were selected. Here, we used three methods: 1) greedy forward search; 2) greedy backward search; and 3) abridged best subset selection (abridged BSS; described below). We selected two signatures from each abridged BSS: (1) the signature with the maximum AUROC in the training data, and (2) the signature with the fewest number of genes but with AUROC within the 95% confidence interval of the maximum AUROC signature. Additional statistical details can be found in Figure 2.

Abridged Best Subset Selection method combines a greedy backward search with an exhaustive search to perform as exhaustive a search as possible within a reasonable computational time. Performing a greedy search alone would be computationally feasible, but because of the nature of the greedy algorithm it does not ensure that one finds the best possible combination of genes for diagnostic purposes. In contrast, best subset selection is an exhaustive search that will always select the optimal combination of genes; however, the computational cost of best subset selection increases exponentially with the number of genes, which makes it infeasible to execute on more than ~20 genes. The Abridged Best Subset Selection (Abridged BSS) combines the strengths of both methods.

First, we ran a greedy backward search on the initial gene list. Briefly, we took the starting gene set and calculated the AUROC after individually removing each of the genes. We identified the gene whose removal leads to the largest increase in AUROC, and then permanently remove that gene from the set. We then applied this same strategy to the new gene set, once again removing the gene whose exclusion resulted in the largest increase in AUROC. In a normal greedy backward search, we would continue repeating this step until we reach a point such that removing any gene resulted in a loss of AUROC that is greater than some pre-defined threshold. However, in this case, the greedy backward search was simply run until 20 genes remained.

Once we had a smaller set of genes, best subset selection could be run on the abridged gene list. Briefly, we assessed the diagnostic power of every possible combination of the genes by calculating the signature scores for each combination and reporting the corresponding AUROC. Next, for every unique number of total genes, the subset of genes that produced the best AUROC was reported. This resulted in a list of the best signatures for each number of total genes, from which the final gene signature was selected.

We tested each signature in the hold-out validation data. While the samples in the hold-out validation data came from the same datasets as the training data, the samples themselves were different and were co-normalized independently. This allowed us to acquire a semi-independent assessment of each signature's diagnostic accuracy. In addition, the drop-off in performance of each signature between training and hold-out validation could be used as a proxy for estimating the degree to which each signature is overfit.

We selected an 8-gene signature that was derived by filtering out the top 100 genes from SAM, running an abridged BSS, and then selecting the cutoff with the minimum number of genes while still being within the 95% confidence interval of the max AUROC. We then assessed the accuracy of this 8-gene signature in the 37 independent validation datasets, which were set aside at the beginning of the analysis. Here, out of the 37 datasets, 32 were COCONUT co-normalized; the remaining 5 datasets, which did not contain healthy samples, were not COCONUT co-normalized. The validation datasets were completely independent from those included in the training and hold-out validation sets, which allowed assessment of a signature in more heterogeneous and independent cohorts to ensure that it was not confounded by and is generalizable across a broad spectrum of patient populations, even when applied to samples containing previously unseen clinical, biological, and technical heterogeneity. Finally, we validated the 8-gene signature in two prospective cohorts from two countries (Nepal and Laos; described below) to directly assess its real-world diagnostic accuracy. Additional statistical details can be found in Table S7.

Calculation of the BoVI score

In order to perform disease classification, we defined a BoVI score (S_i) for a sample based on our previously described signature score.[26,74] The BoVI score is defined as the geometric mean of the genes over-expressed in bacterial infection minus the geometric mean of the genes over-expressed in viral infection (Equation 4).

$$S_i = \left(\prod_{gene \in pos} x_i(gene)\right)^{\frac{1}{|pos|}} - \left(\prod_{gene \in neg} x_i(gene)\right)^{\frac{1}{|neg|}} \qquad \text{(Equation 4)}$$

Summary ROC curves

Summary ROC curves represent a weighted average of multiple independent ROC curves. When fewer than 5 curves were present, we generated unsmoothed summary ROC curves as follows. We used linear interpolation to approximate the true positive rate (TPR) values for each curve, so that TPR values could be aligned without changing the corresponding false positive rate (FPR) values. For each curve, we assigned weights based on the number of samples used to create the corresponding curve and calculated a summary ROC curve by taking the weighted mean of the corresponding TPR values. In addition, we calculated the weighted SD for each TPR, which was represented by a gray area on the plot. Finally, we calculated the summary AUROC using the trapezoid rule and the 95% confidence interval using the pooled SE of the individual curves. When greater than 5 curves were present, we generated a smoothed summary ROC curve using a modified version of the method of Kester and Buntinx[144] as previously described.[74] Summary ROC curve is used in Figure 3D.

Selecting optimal cutoffs with the Youden Index

When a discrete signature score cutoff needed to be selected (e.g., for calculating sensitivity/specificity), the Youden Index was sometimes used to identify an optimal cutoff. The Youden Index is defined as the cutoff which maximizes the sum of sensitivity and specificity.[145,146]

Whole genome transcript profiling

We performed human whole genome transcript profiling in the Laos Microarray cohort. We amplified total RNA using the TargetAmp Aminoallyl aRNA amplification kit (Epicentre, Madison, CA). We labeled eight ug of amplified RNA and 5 ug of amplified Universal Human Reference RNA (Agilent, Santa Clara, CA) using Cy5 and Cy3 dyes, respectively, and hybridized to Human Exonic Evidence Based Oligonucleotide (HEEBO) microarrays. Details of the protocol have been described previously.[33] HEEBO microarrays, containing 44,544 probes, were printed by the Stanford Functional Genomics Core Facility.

We filtered data to exclude control probes, calculated background measurements using the Edwards correction, and then normalized data using a 2D spatial loess estimation.[147] For replicate probes, we averaged the expression values.

Fluidigm RT-PCR validation

We validated the 8-gene signature Fluidigm RT-PCR using samples from the prospective cohorts from Laos and Nepal. There was no overlap between the patients enrolled in the Laos Microarray and Laos Fluidigm cohorts. AROS Applied Biotechnology (Aarhus, Denmark) carried out cDNA synthesis and qPCR using the Fluidigm platform. TaqMan assays and the corresponding genes are listed in Table S12. TaqMan assays were normalized using the geometric mean of three housekeeping genes: *18S, ACTB*, and *KPNA6*. We calculated the BoVI score using the geometric mean of the Ct values, as described above.

Cell Reports Medicine, Volume *3*

Supplemental information

A robust host-response-based signature distinguishes bacterial and viral infections across diverse global populations

Aditya M. Rao, Stephen J. Popper, Sanjana Gupta, Viengmon Davong, Krista Vaidya, Anisone Chanthongthip, Sabine Dittrich, Matthew T. Robinson, Manivanh Vongsouvath, Mayfong Mayxay, Pruksa Nawtaisong, Biraj Karmacharya, Simone A. Thair, Isaac Bogoch, Timothy E. Sweeney, Paul N. Newton, Jason R. Andrews, David A. Relman, and Purvesh Khatri

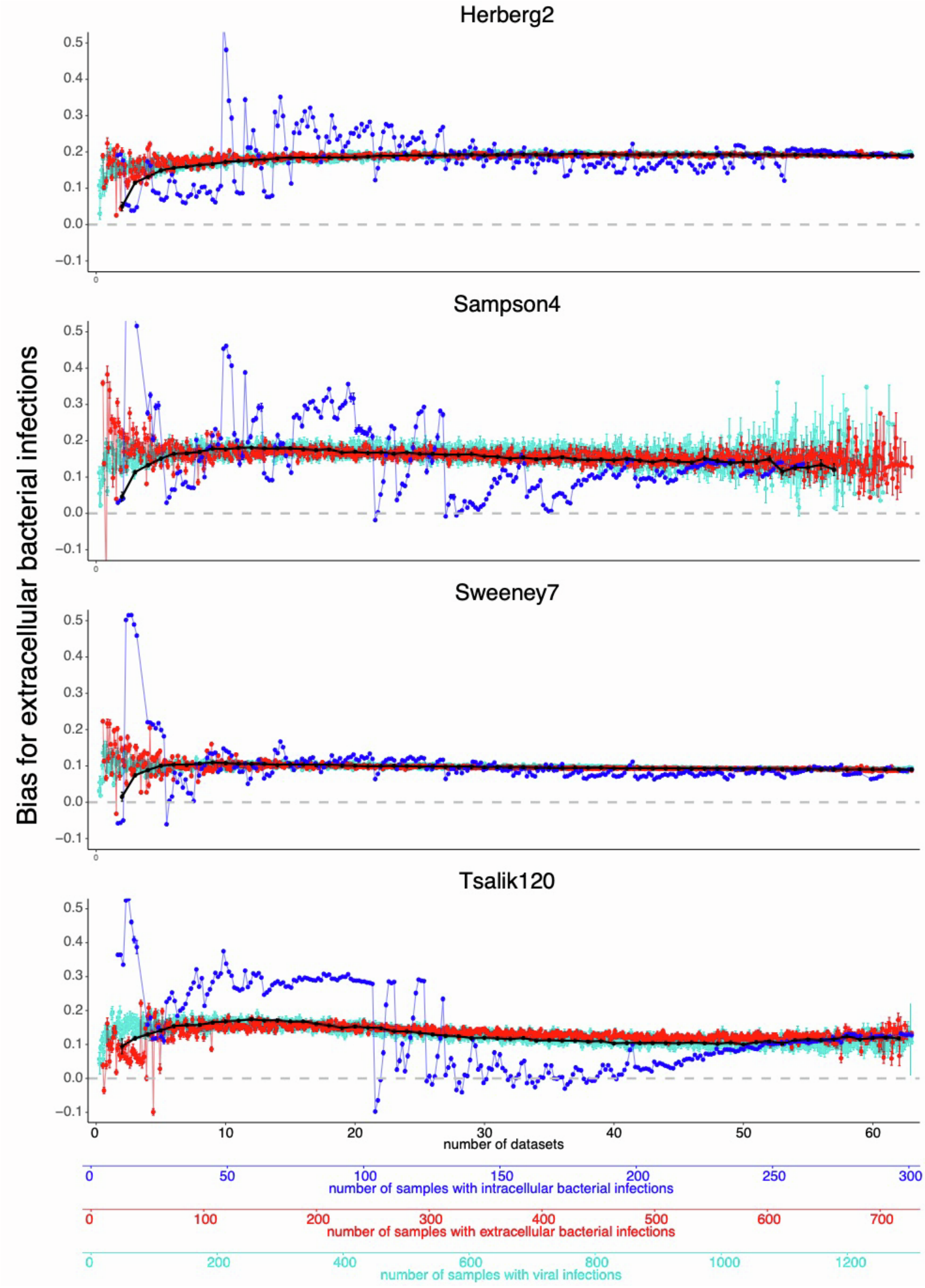

Herberg2
Sampson4
Sweeney7
Tsalik120
Bias for extracellular bacterial infections
0.5
0.4
0.3
0.2
0.1
0.0
−0.1
0
10
20
30
40
50
60
number of datasets
0
50
100
150
200
250
300
number of samples with intracellular bacterial infections
0
100
200
300
400
500
600
700
number of samples with extracellular bacterial infections
0
200
400
600
800
1000
1200
number of samples with viral infections

Figure S1. **Lower accuracy in intracellular bacterial infections is not dependent on the number of datasets or samples used.** Summary of the difference in performance between extracellular and intracellular bacterial infections for 1,396,677 unique, randomly sampled subsets of our 64 datasets. Bias for extracellular bacterial infections was calculated by taking the extracellular bacterial vs. viral infection AUROC and subtracting the intracellular bacterial vs. viral infection AUROC. The dotted line at 0 indicates no bias, and anything above the dotted line indicates positive bias (i.e., higher performance for extracellular vs. viral infection). The bias was averaged for each unique number of datasets (black) or samples in each of the three categories (intracellular bacterial infection = blue; extracellular bacterial infections = red; viral infections = cyan). The x-axes were scaled to match each other and then curves were overlaid. The error bar indicates the standard error of each measurement. **Related to Figure 1**.

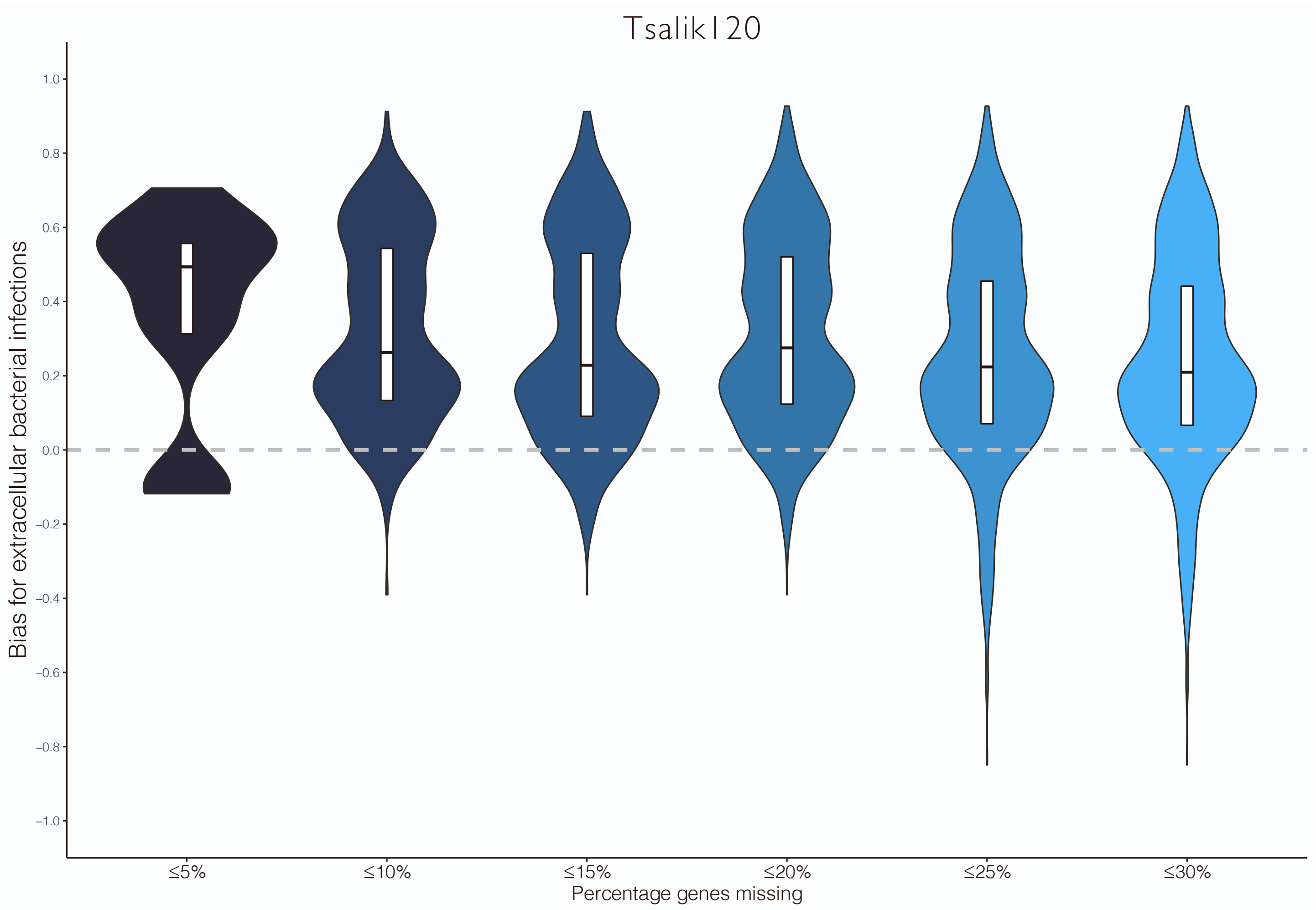

Figure S2. **The AUROC differential in the Tsalik120 signature is less biased when fewer genes are missing.** Summary of the difference in performance between extracellular and intracellular bacterial infections for 1,396,677 unique, randomly sampled subsets of our 64 datasets. The difference in performance was averaged for each unique number of missing genes in the Tsalik120 signature. The violin plots represent the distribution of AUROCs for all combinations where at most the given percentage of genes are missing. The horizontal grey dotted line at 0 indicates no difference between extracellular vs. viral and intracellular vs. viral performance, and anything above the dotted line indicates higher performance for extracellular vs. viral performance. **Related to Figure 1**.

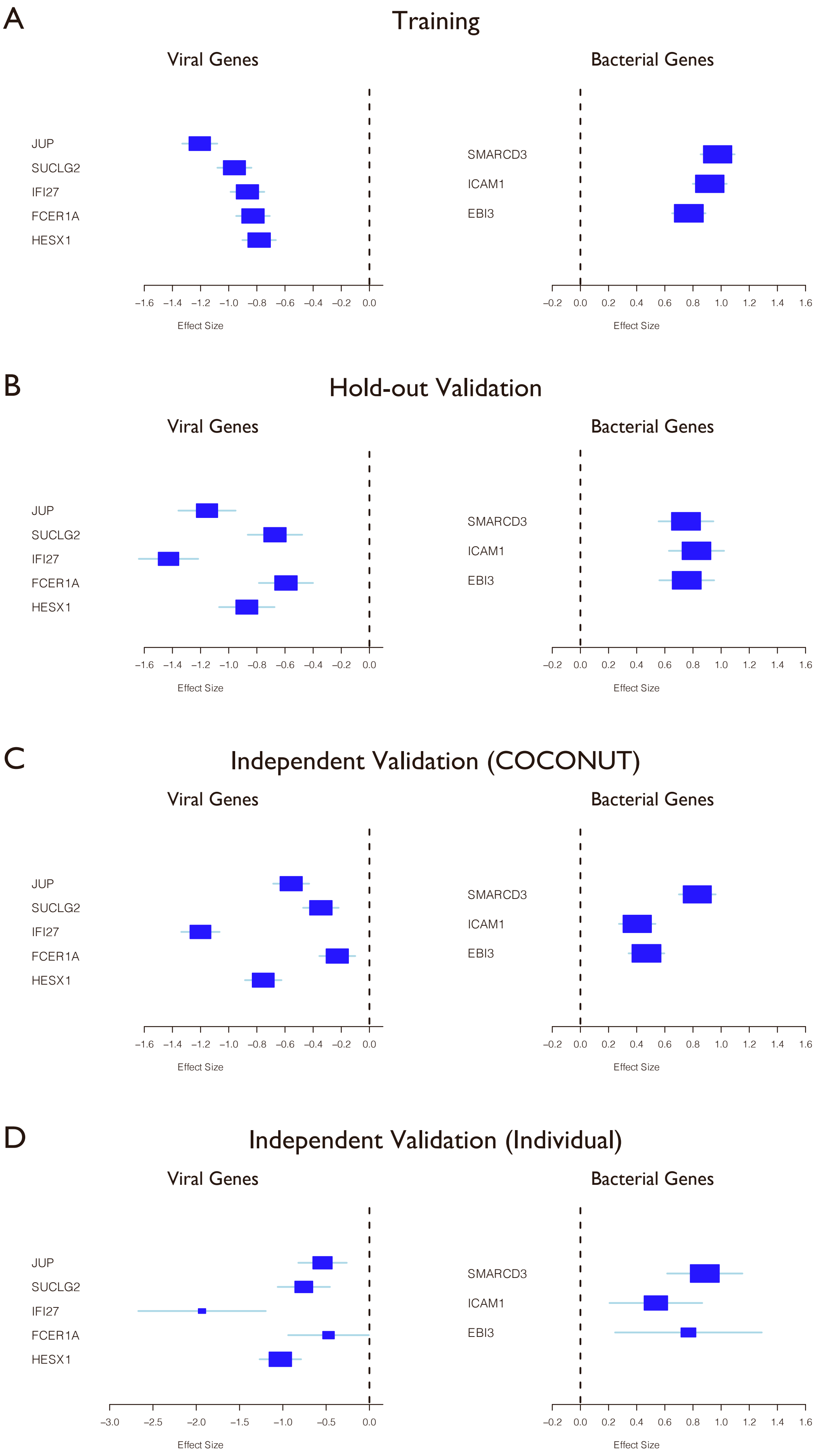

A
Training
Viral Genes
JUP
SUCLG2
IFI27
FCER1A
HESX1
−1.6 −1.4 −1.2 −1.0 −0.8 −0.6 −0.4 −0.2 0.0
Effect Size
Bacterial Genes
SMARCD3
ICAM1
EBI3
−0.2 0.0 0.2 0.4 0.6 0.8 1.0 1.2 1.4 1.6
Effect Size
B
Hold-out Validation
Viral Genes
JUP
SUCLG2
IFI27
FCER1A
HESX1
−1.6 −1.4 −1.2 −1.0 −0.8 −0.6 −0.4 −0.2 0.0
Effect Size
Bacterial Genes
SMARCD3
ICAM1
EBI3
−0.2 0.0 0.2 0.4 0.6 0.8 1.0 1.2 1.4 1.6
Effect Size
C
Independent Validation (COCONUT)
Viral Genes
JUP
SUCLG2
IFI27
FCER1A
HESX1
−1.6 −1.4 −1.2 −1.0 −0.8 −0.6 −0.4 −0.2 0.0
Effect Size
Bacterial Genes
SMARCD3
ICAM1
EBI3
−0.2 0.0 0.2 0.4 0.6 0.8 1.0 1.2 1.4 1.6
Effect Size
D
Independent Validation (Individual)
Viral Genes
JUP
SUCLG2
IFI27
FCER1A
HESX1
−3.0 −2.5 −2.0 −1.5 −1.0 −0.5 0.0
Effect Size
Bacterial Genes
SMARCD3
ICAM1
EBI3
−0.2 0.0 0.2 0.4 0.6 0.8 1.0 1.2 1.4 1.6
Effect Size

Figure S3. **The 8 signature genes exhibit consistent differential expression patterns across all analysis stages.** Forest plots of the 8 signature genes in the BoVI signature, with bacterial samples as the positive class and viral samples as the negative class. These forest plots display the effect sizes for each signature gene, computed as Hedges' *g*. The size of the blue rectangles is proportional to the standard error of the effect size. Whiskers represent the 95% confidence interval. **(A-C)** Effect sizes were computed across all samples. **(D)** Summary effect sizes were computed for each gene by performing a meta-analysis across all 5 datasets. Meta-analysis was done using the R package *MetaIntegrator*. **Related to Figure 2**.

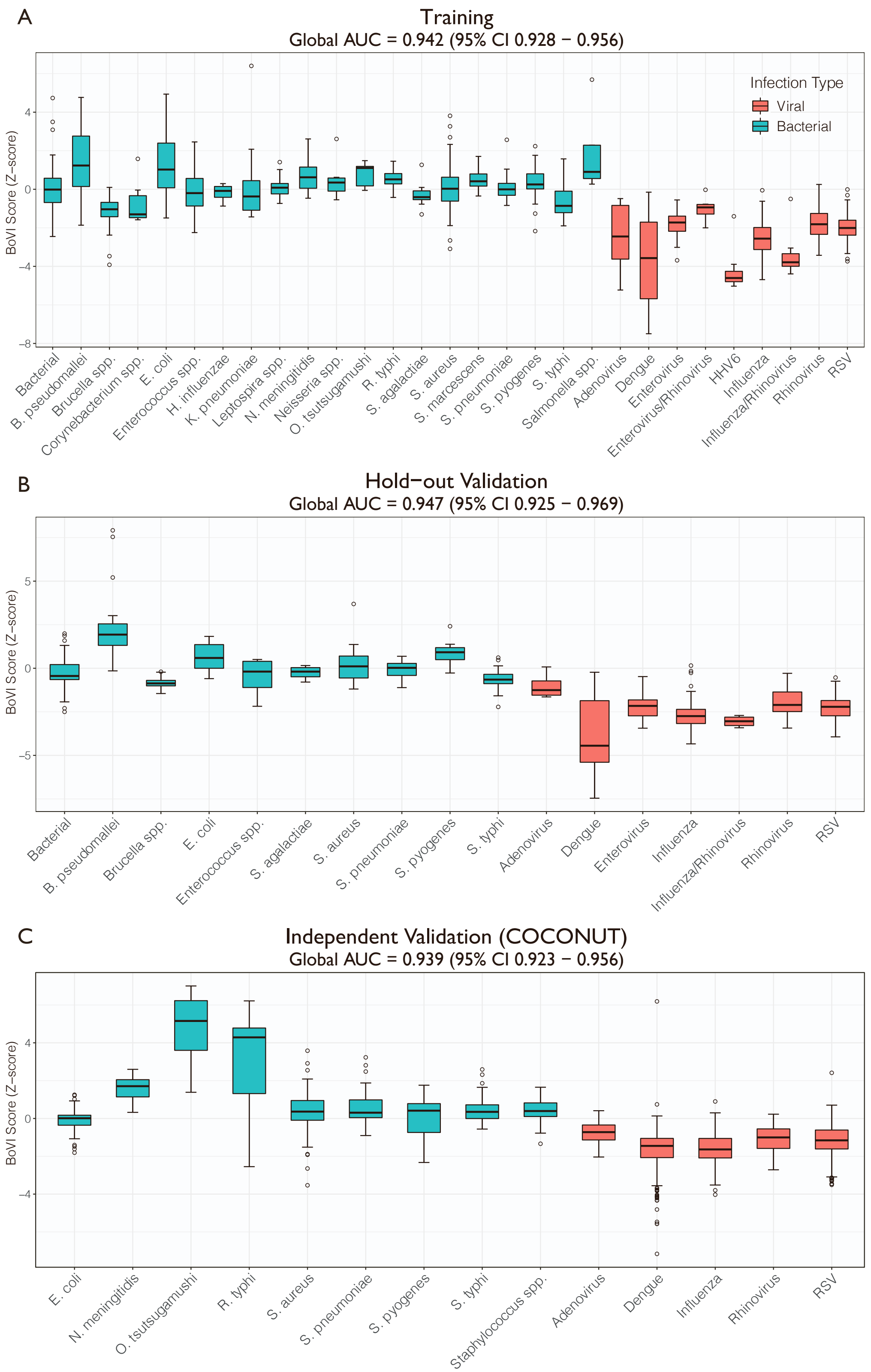

Figure S4. **The BoVI signature works across a wide variety of pathogens.** Boxplots of the BoVI score across different pathogens, for each in-silico analysis stage. Scores were centered at 0 and then the Z-score was calculated. Any bacterial infection with a non-specific diagnosis was listed as "Bacterial". Only infecting pathogens that were present in at least 4 samples were displayed. **Related to Figure 3**.

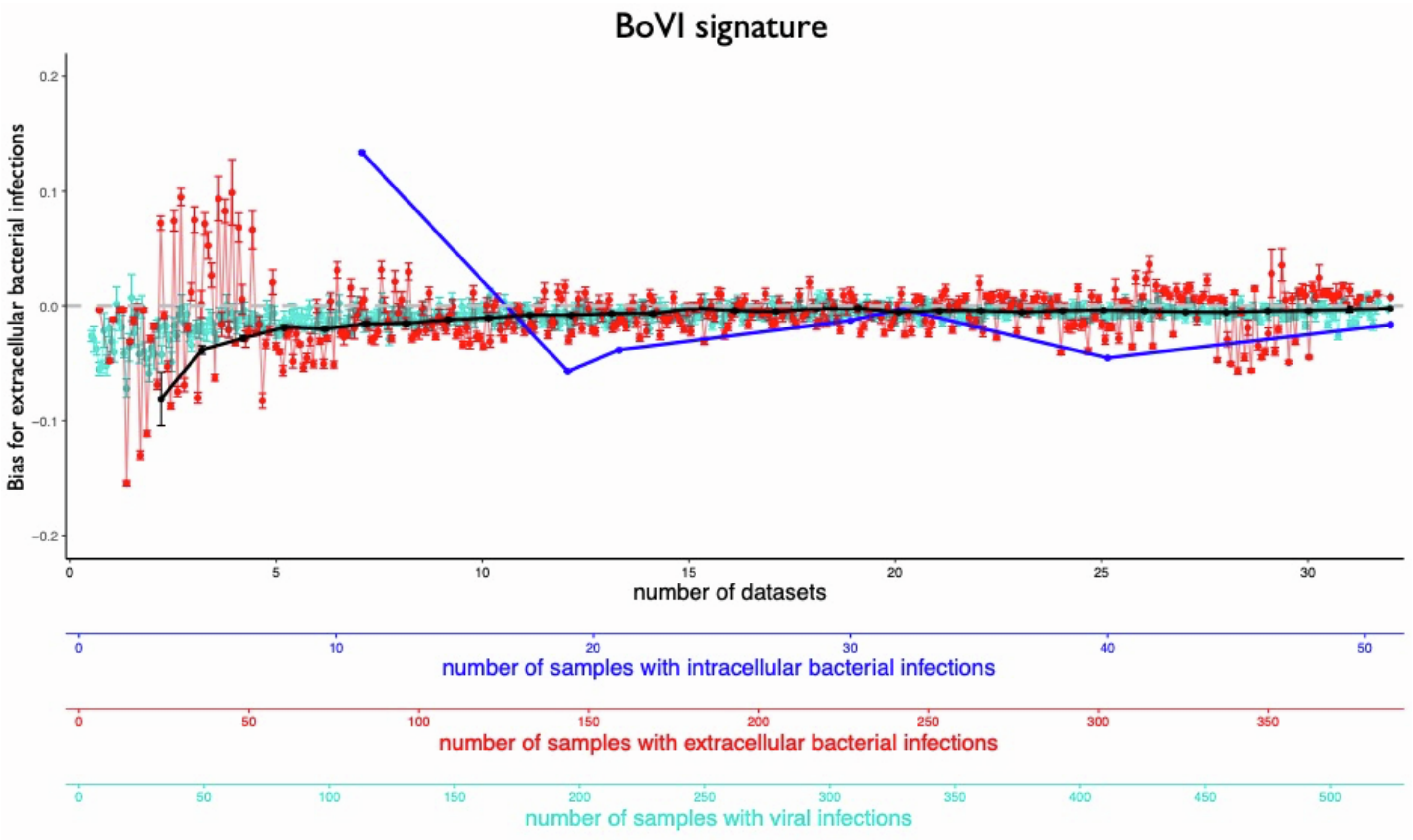

Figure S5. **The BoVI score is not biased toward extracellular bacterial infections, regardless of the number of datasets or samples used.** Summary of the difference in performance between extracellular and intracellular bacterial infections for 435,902 unique, randomly sampled subsets of our 32 independent validation datasets. Bias for extracellular bacterial infections was calculated by taking the extracellular bacterial vs. viral infection AUROC and subtracting the intracellular bacterial vs. viral infection AUROC. Bias was averaged for each unique number of datasets (black) or samples in each of the three categories (intracellular bacterial infection = blue; extracellular bacterial infections = red; viral infections = cyan). The dotted line at 0 indicates no bias, and anything above the dotted line indicates positive bias (i.e., higher performance for extracellular vs. viral infection). The x-axes were scaled to match each other and then curves were overlaid. The error bar indicates the standard error of each measurement. **Related to Figure 3**.

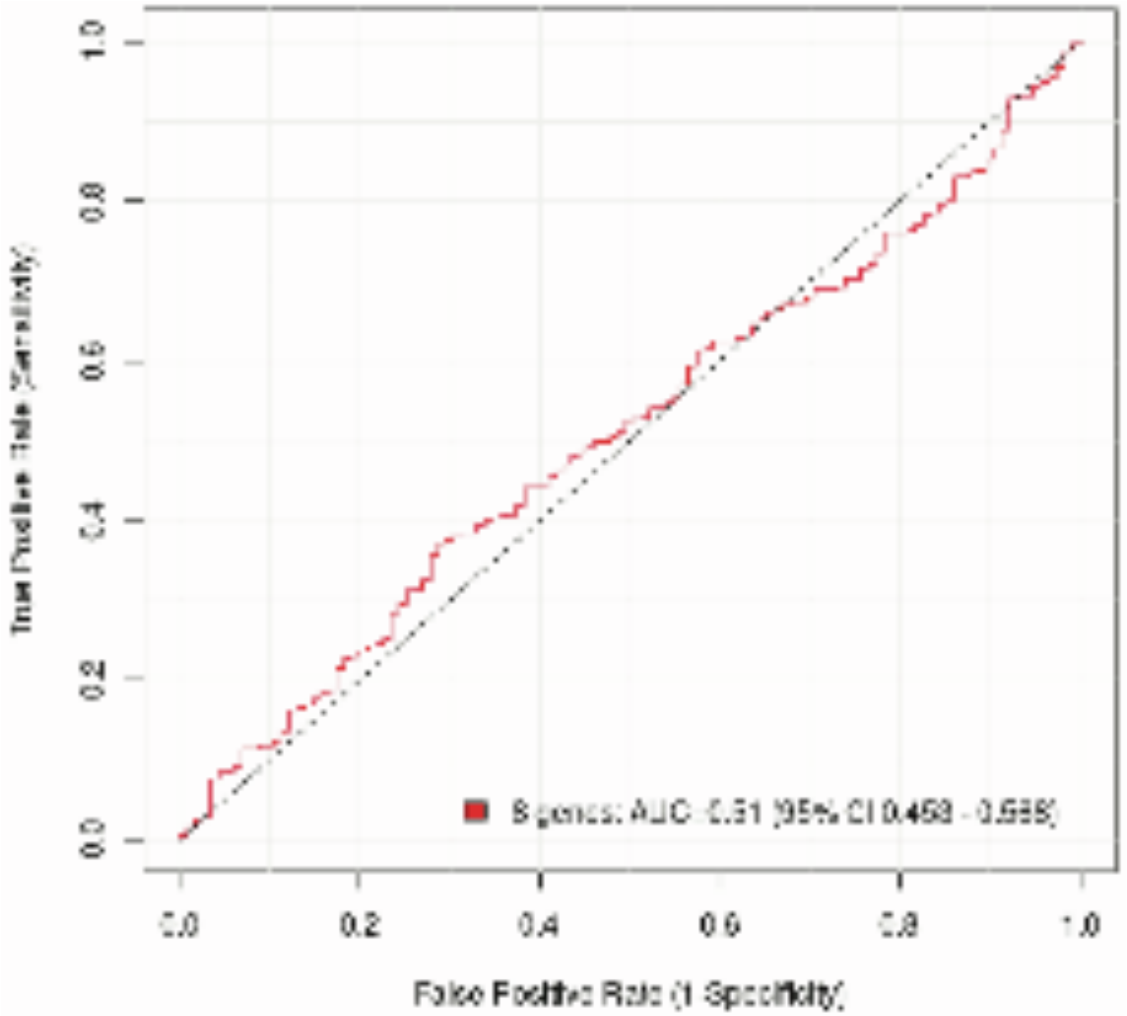

Figure S6. The 8-gene signature does not distinguish healthy controls from studies that profiled either patients with TB or with HIV, demonstrating it is not affected by batch effects between these studies. Related to Figure 3.

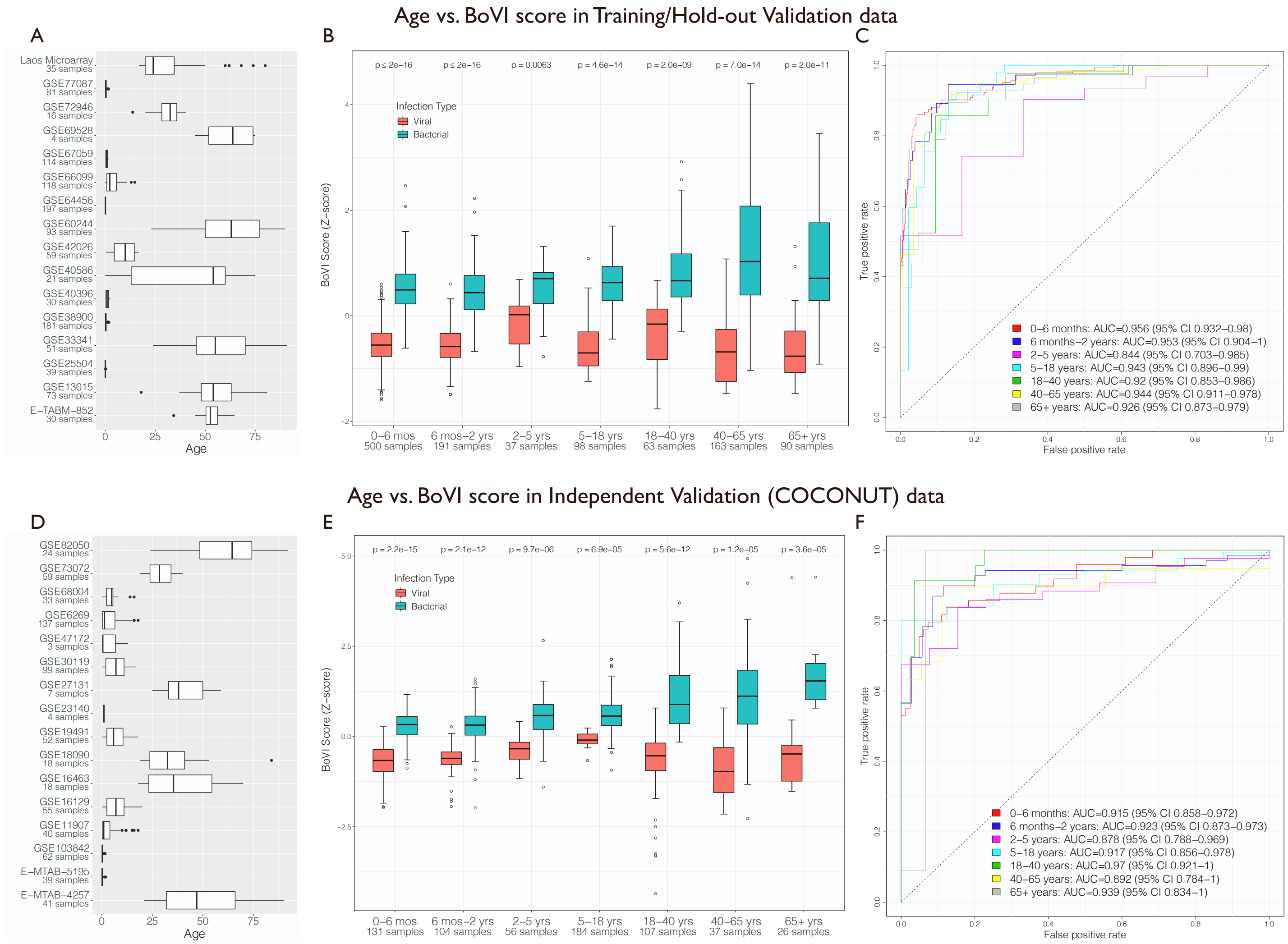

Figure S7. **The BoVI signature maintains high accuracy across all ages.** Performance of the BoVI signature was calculated for different age windows across training, hold-out validation, and independent validation samples. Samples were used only if (i) the exact age was listed, (ii) a range of ages was provided that fit completely into a single age window, or (iii) the mean and standard deviation of the ages was provided and all ages that were within one standard deviation of the mean fit into a single age window. In training/hold-out validation, 1,035 samples had the exact age listed and 107 samples had a usable age range. In independent validation, 600 samples had the exact age listed and 45 samples had a usable age range. Overall, there were 1,142 samples in training/hold-out validation and 645 samples in independent validation that were used. **(A,D)** All samples with usable age information were plotted to show the distribution of ages within each dataset. Ages were randomly generated for the 107 samples in training/hold-out validation and 45 samples in independent validation which only had age range info available. For samples with an exact range provided, ages were randomly sampled from a uniform distribution. For samples with the mean and standard deviation provided, ages were randomly sampled from a normal distribution. **(B,E)** Scores were centered at 0 and then the Z-score was calculated. P-values were calculated using the Mann-Whitney U test. **(C,F)** ROC curves comparing the performance of the BoVI signature across each age window. mos, months; yrs, years. **Related to Figure 4**.

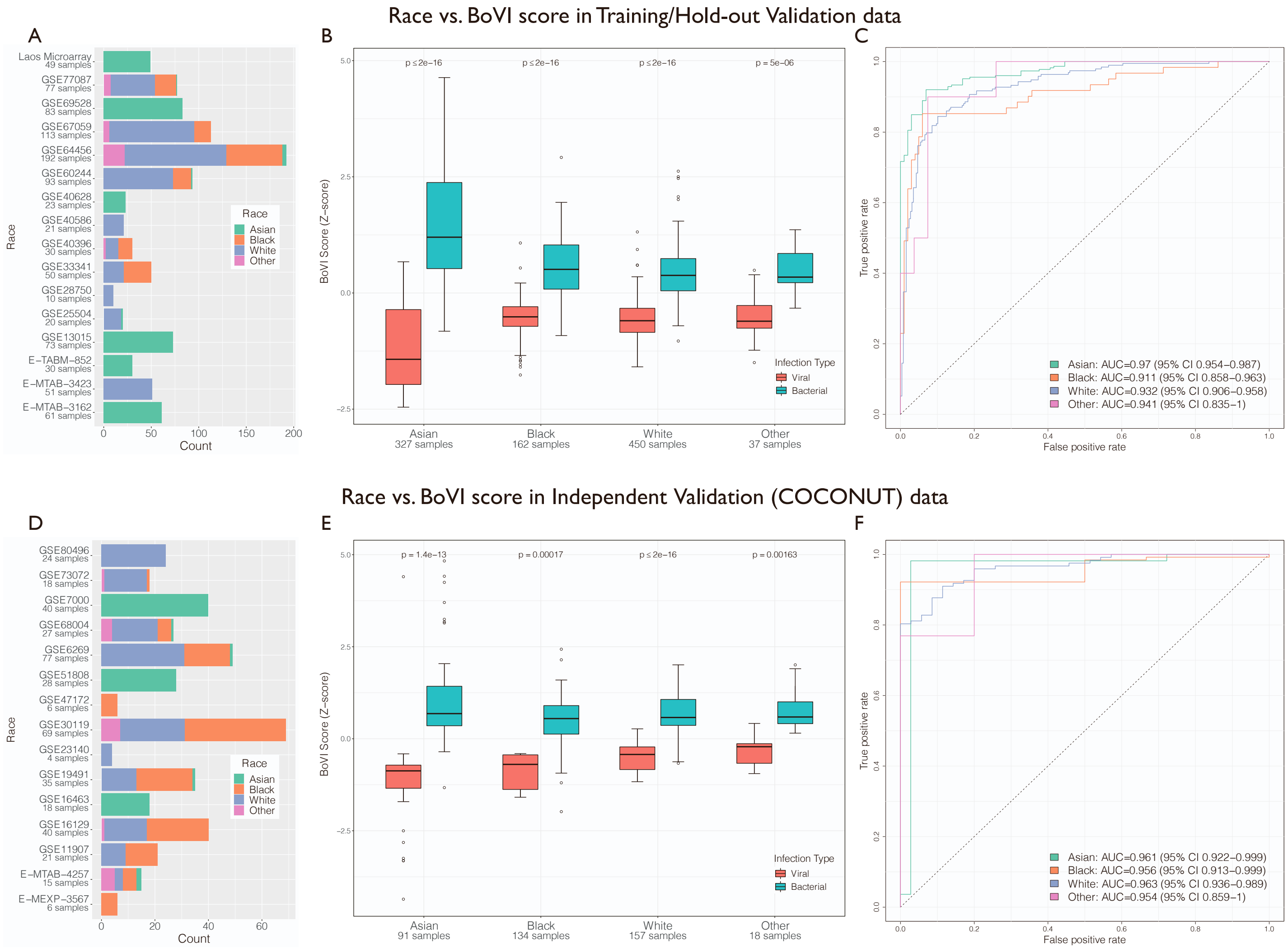

Figure S8. **The BoVI signature retains high accuracy regardless of race.** Performance of the BoVI signature was calculated for each race category across training, hold-out validation, and independent validation samples. Overall, there were 976 samples in training/hold-out validation and 400 samples in independent validation that were used. **(A,D)** All samples with information on race were plotted to show the distribution within each dataset. **(B,E)** Scores were centered at 0 and then the Z-score was calculated. P-values were calculated using the Mann-Whitney U test. **(C,F)** ROC curves comparing the performance of the BoVI signature across each race category. **Related to Figure 4**.

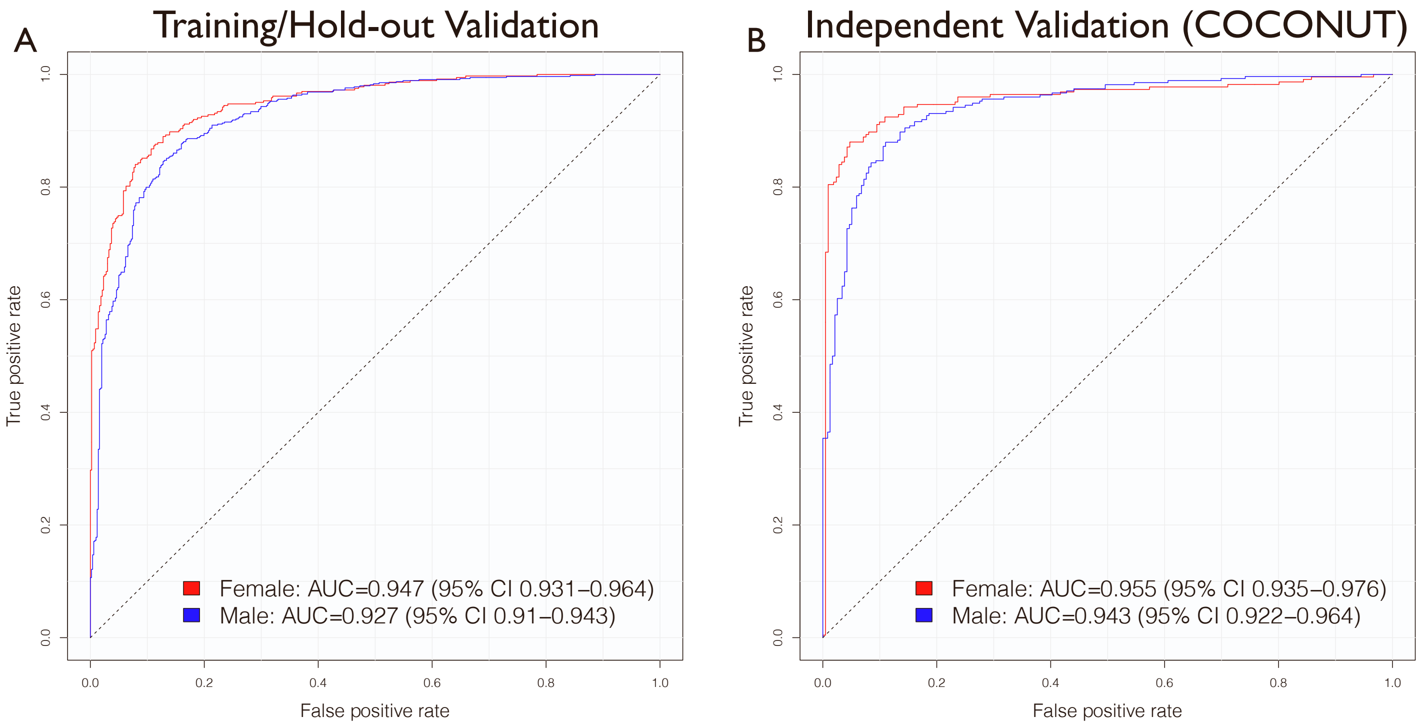

Figure S9. **The BoVI signature's performance is unaffected by sex.** ROC curves comparing the performance of the BoVI signature for all male or all female samples. Overall, there were 1,839 samples in training/hold-out validation and 923 samples in independent validation that were used. **(A)** Performance across training and hold-out validation samples. **(B)** Performance across independent validation samples. **Related to Figure 4**.

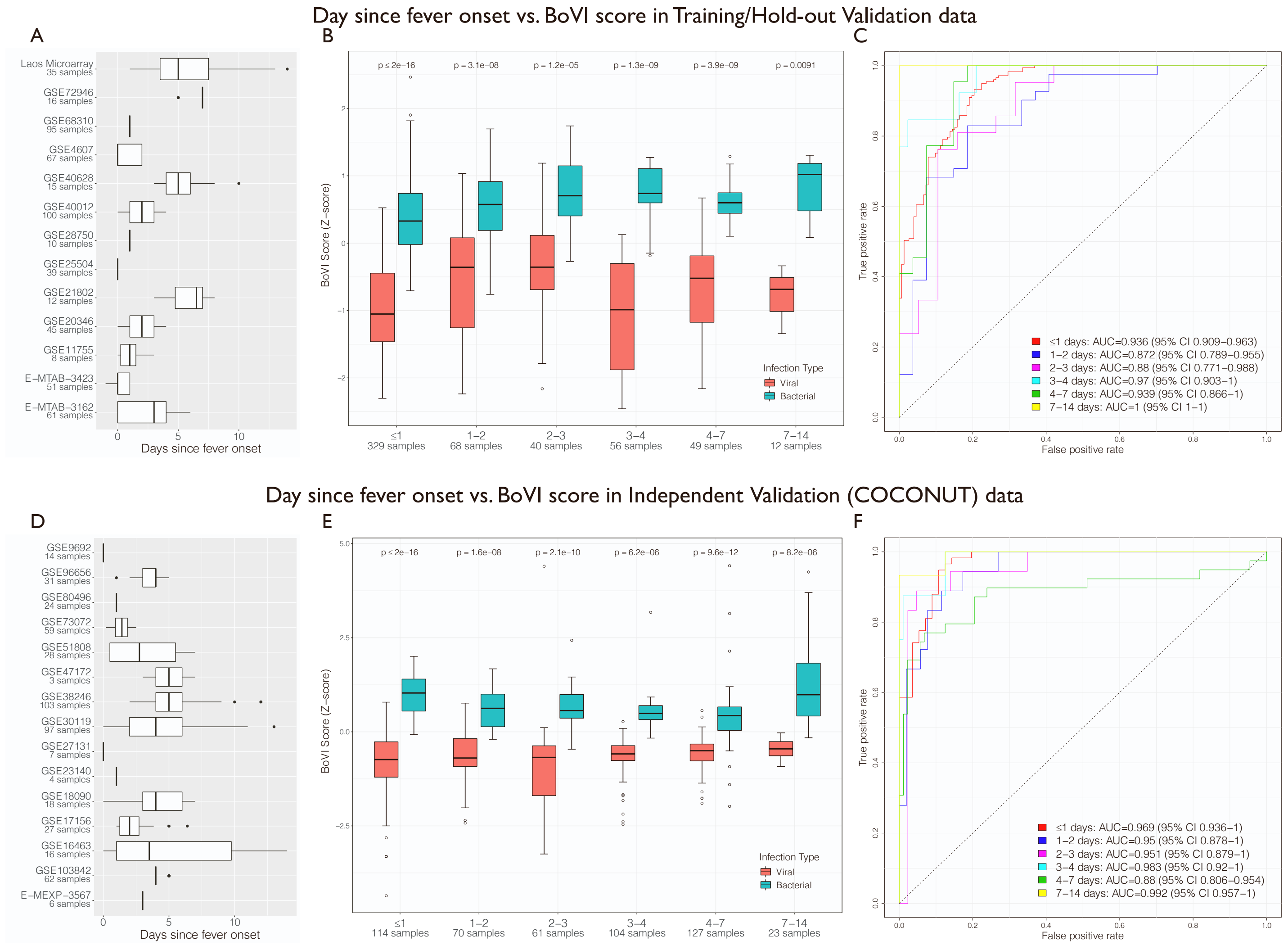

Figure S10. The BoVI signature effectively distinguishes between bacterial and viral infection up to two weeks after the first incidence of febrile symptoms. Performance of the BoVI signature was calculated for different windows of the number of days since fever onset across training, hold-out validation, and independent validation samples. Only samples that were taken within 14 days of fever onset were used. Overall, there were 554 samples in training/hold-out validation and 499 samples in independent validation that were used. **(A,D)** All samples with information on the number of days since fever onset were plotted to show the distribution within each dataset. **(B,E)** Scores were centered at 0 and then the Z-score was calculated. P-values were calculated using the Mann-Whitney U test. (**C,F**) ROC curves comparing the performance of the BoVI signature across each window of days since fever onset. **Related to Figure 4**.

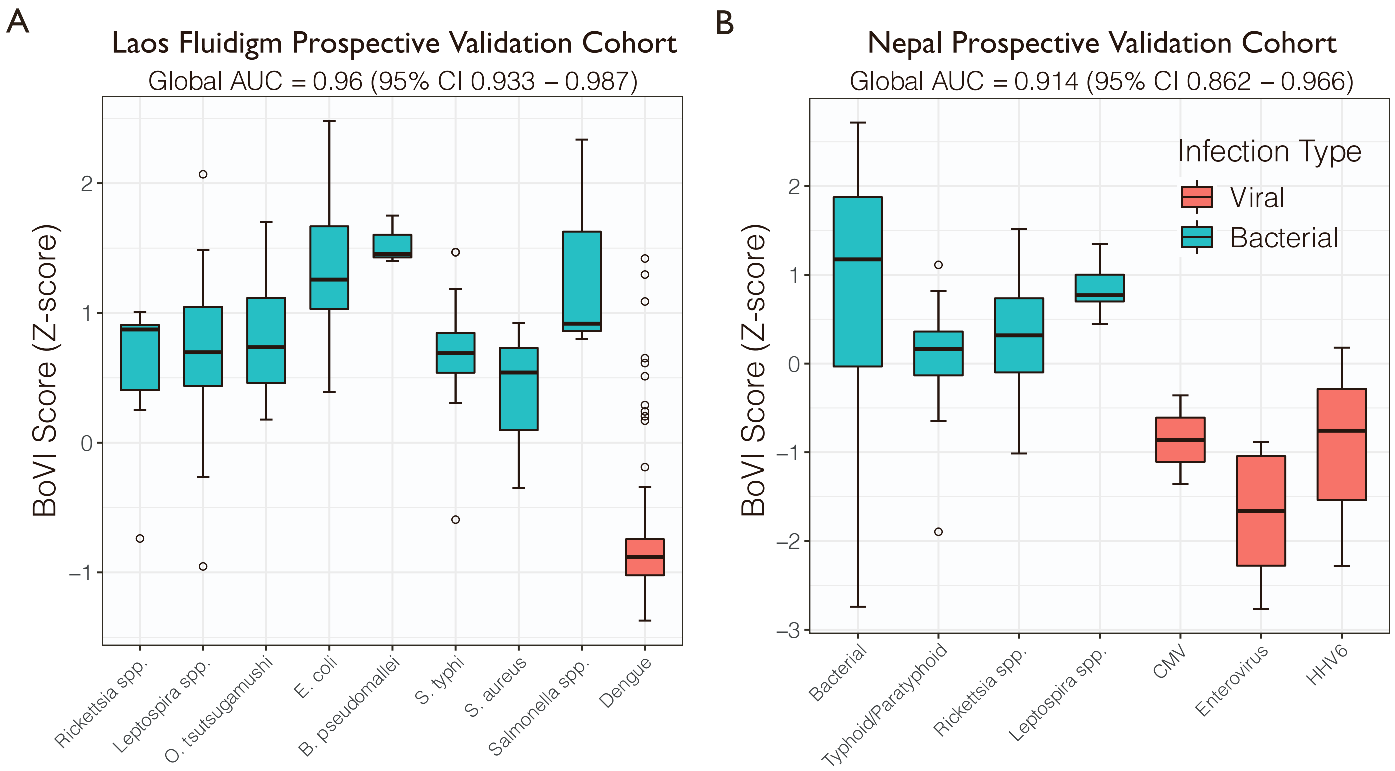

Figure S11. **Performance of the BoVI signature in the prospective validation cohorts.** Scores were centered at 0 and then the Z-score was calculated. Any bacterial infection with a non-specific diagnosis was listed as "Bacterial". Only infecting pathogens that were present in at least 3 samples were displayed. **Related to Figure 5.**

Table S1: Datasets used in the training and hold-out validation cohorts, related to Figure 1 and 2.

The Laos Microarray cohort was used separately for the analysis of existing bacterial vs. viral signatures, but was combined with the other datasets during signature discovery. WB, whole blood; PBMC, peripheral blood mononuclear cells; HRV, human rhinovirus; RSV, respiratory syncytial virus; HSV, herpes simplex virus; CMV, cytomegalovirus; HHV6, human herpesvirus 6; HMPV, human metapneumovirus; HPIV, human parainfluenza virus; BKV, BK virus; PICU, pediatric intensive care unit; ICU, intensive care unit; NICU; neonatal intensive care unit; ARI, acute respiratory infection; CAP, community acquired pneumonia; LRTI, lower respiratory tract infection.

Accession	Author	Platform	Tissue	Location	Demographic	Clinical setting(s)	Bacteria	Viruses	# of healthy samples	# of extracellular bacterial samples	# of intracellular bacterial samples	# of unknown bacterial	# of viral samples
Laos Microarray	Popper	GPL20858	WB	Laos	Adults with systemic infections	Inpatient	*S. typhi*, *R. typhi*, *O. tsutsugamushi*, *E. coli*	Dengue	12	6	30	0	13
GSE64456	Mahajan	GPL10558	WB	USA	Febrile infants ≤ 60 days old	Emergency department	Unknown	Influenza, Enterovirus, HRV, RSV	19	0	0	89	108
GSE72946	Lindow	GPL17077	WB	Brazil	Adults with acute leptospirosis	Inpatient, ICU	*Leptospira* spp.		4	16	0	0	0
E-MTAB-3423	Blohmke	GPL10558	WB	UK	Adults challenged with infective dose of S. typhi	Outpatient	*S. typhi*		40	0	47	0	0
E-TABM-852	Koh	GPL6884	WB	Thailand	Adults with melioidosis	Inpatient	*B. pseudomallei*		10	0	30	0	0
GSE40628	Simmons	GPL16021	WB	Vietnam	Dengue patients at hospital admission	Inpatient		Dengue	4	0	0	0	23
GSE69597	Khaenam	GPL16791	WB	Macedonia, Spain	Acute brucellosis patients	Unknown	*Brucella* spp.		40	0	103	0	0
GSE77087	de Steenhuijsen Piters	GPL10558	WB	USA	Young children with mild and severe RSV disease	Outpatient, Pediatric ward, PICU		RSV	23	0	0	0	81
GSE4607	Wong	GPL570	WB	USA	Septic children in the PICU	PICU	Multiple	Influenza, Rotavirus,	15	48	2	4	13
GSE40396	Hu	GPL10558	WB	USA	Febrile children in the emergency department	Emergency department	*S. aureus*, *E. coli*, *Salmonella* spp.	Adenovirus, Enterovirus, HHV6	22	6	1	0	22
GSE42026	Herberg	GPL6947	WB	UK	Children admitted with febrile infections	Inpatient, PICU	Gram-positive	Influenza, RSV	33	0	0	18	41
GSE21802	Bermejo-Martin	GPL6102	WB	Spain	Adults with septic influenza	ICU		Influenza	4	0	0	0	12

GSE68310	Zhai	GPL10558	WB	USA	Adults with ARIs	Outpatient		Influenza, HRV, RSV, Enterovirus, Coronavirus	98	0	0	0	95
GSE20346	Parnell	GPL6947	WB	Australia	Adults with CAP	ICU	Unknown	Influenza	18	0	0	6	4
GSE40012	Parnell	GPL6947	WB	Australia, Hong Kong	Adults with CAP	ICU	Unknown	Influenza	18	0	0	16	8
GSE66099	Sweeney	GPL570	WB	USA	Septic children in the PICU	PICU	Multiple	Influenza, HSV, HMPV, HPIV, BKV, Adenovirus, CMV	47	103	0	4	11
GSE60244	Suarez	GPL10558	WB	USA	Adults hospitalized with LRTI	Inpatient	Unknown		40	0	0	22	0
GSE11755	Emonts	GPL570	WB	Netherlands	Children in PICU with meningococcal sepsis	PICU	Unknown		3	0	0	6	0
GSE28750	Sutherland	GPL570	WB	Australia	Adults with community-acquired bacterial sepsis	ICU	Unknown		20	0	0	10	0
GSE29161	Thuny	GPL6480	WB	France	Adults with native valve-infected endocarditis	Inpatient	Unknown		5	0	0	5	0
GSE33341	Ahn	GPL571	WB	USA	Adults with septic bloodstream infections	Inpatient	*E. coli* , *S. aureus*		43	51	0	0	0
GSE40586	Lill	GPL6244	WB	Estonia	Bacterial meningitis patients	Inpatient	Multiple		18	19	1	1	0
GSE69528	Khaenam	GPL10558	WB	Thailand	Adults with bacterial sepsis	Unknown	Multiple		28	45	32	5	0
E-MTAB-3162	van de Weg	GPL570	WB	Indonesia	Indonesian patients > 14 years old with uncomplicated and severe dengue	Outpatient, Inpatient		Dengue	15	0	0	0	61
GSE25504 GPL13667	Dickinson	GPL13667	WB	UK	Septic neonates	Neonatal ward, NICU	Multiple	HRV, CMV	6	7	0	2	3
GSE25504 GPL6947	Dickinson	GPL6947	WB	UK	Septic neonates	Neonatal ward, NICU	Multiple	CMV	32	22	0	2	1
GSE13015 GPL6106	Pankla	GPL6106	WB	Thailand	Adults with bacterial sepsis	Unknown	Multiple		3	18	26	1	0
GSE13015 GPL6947	Pankla	GPL6947	WB	Thailand	Adults with bacterial sepsis	Unknown	Multiple		5	7	8	0	0

GSE38900 GPL10558	Mejias	GPL10558	WB	USA	Children with acute LRTI	Outpatient, Inpatient	RSV	8	0	0	0	28
GSE38900 GPL6884	Mejias	GPL6884	WB	USA, Finland	Children with acute LRTI	Outpatient, Inpatient	Influenza, HRV, RSV	31	0	0	0	153
GSE67059 GPL6947	Heinonen	GPL6947	WB	USA, Spain, Finland	Previously healthy children with asymptomatic or symptomatic HRV	Outpatient, Inpatient	HRV	21	0	0	0	82
GSE67059 GPL10558	Heinonen	GPL10558	WB	USA, Spain, Finland	Previously healthy children with asymptomatic or symptomatic HRV	Outpatient, Inpatient	HRV	16	0	0	0	32

Table S2: COCONUT co-normalized datasets used for independent validation of the BoVI signature, related to Figure 1 and 2.

WB, whole blood; PBMC, peripheral blood mononuclear cells; HRV, human rhinovirus; RSV, respiratory syncytial virus; ARI, acute respiratory infection; ICU, intensive care unit; ED, emergency department; PICU, pediatric intensive care unit.

Accession	Author	Platform	Tissue	Location	Demographic	Clinical setting(s)	Bacteria	Viruses	# of healthy samples	# of extracellular bacterial samples	# of intracellular bacterial samples	# of unknown bacterial	# of viral samples
GSE7000 GPL4857	Thompson	GPL4857	WB	Vietnam	Patients with typhoid fever	Inpatient	*S. typhi* , *S. enterica*		15	0	21	0	0
GSE7000 GPL4858	Thompson	GPL4858	WB	Vietnam	Patients with typhoid fever	Inpatient	*S. typhi* , *S. enterica*		3	0	19	0	0
GSE51808	Kwissa	GPL13158	WB	Thailand	Patients with dengue fever or dengue hemorrhagic fever	Inpatient		Dengue	9	0	0	0	28
E-MEXP-3567	Irwin	GPL96	WB	Malawi	Children with bacterial meningitis or pneumonia	ED	*S. pneumoniae*		3	6	0	0	0
GSE17156	Zaas	GPL571	WB	USA, UK	Adults with respiratory viral infection	Outpatient		Influenza, HRV, RSV	56	0	0	0	27
GSE103842	Rodriguez-Fernandez	GPL10558	WB	USA	Young children hospitalized with bronchiolitis	Inpatient		RSV	12	0	0	0	62
GSE30119	Banchereau	GPL6947	WB	USA	Children with community acquired Staph infection	Inpatient, PICU	*S. aureus*		44	99	0	0	0
GSE96656	Popper	GPL20858	WB	Nicaragua	Dengue patients	Inpatient, ICU		Dengue	9	0	0	0	31
GSE47172	Kulohoma	GPL96	WB	Malawi	Children with pneumococcal meningitis	Inpatient	*S. pneumoniae*		3	6	0	0	0
GSE80496	Herberg	GPL6883	WB	UK, Spain, Netherland, USA	Children < 17 years old with meningococcal disease	Inpatient, PICU	*N. meningitidis*		21	24	0	0	0

GSE82050	Tang	GPL21185	WB	Germany	Adults with influenza	Inpatient, ICU		Influenza	15	0	0	0	24
E-MTAB-5195	Jong	GPL570	WB	Netherlands	Infants with RSV	Inpatient, ICU		RSV	4	0	0	0	39
GSE73072 (RSV DEE1)	Liu	GPL14604	WB	USA	Patients in the acute phase of a viral challenge study	Outpatient		RSV	20	0	0	0	9
GSE73072 (H3N2 DEE2)	Liu	GPL14604	WB	USA	Patients in the acute phase of a viral challenge study	Outpatient		Influenza	17	0	0	0	9
GSE73072 (H1N1 DEE3)	Liu	GPL14604	WB	USA	Patients in the acute phase of a viral challenge study	Outpatient		Influenza	22	0	0	0	9
GSE73072 (H1N1 DEE4)	Liu	GPL14604	WB	USA	Patients in the acute phase of a viral challenge study	Outpatient		Influenza	19	0	0	0	5
GSE73072 (H3N2 DEE5)	Liu	GPL14604	WB	USA	Patients in the acute phase of a viral challenge study	Outpatient		Influenza	21	0	0	0	8
GSE73072 (HRV UVA)	Liu	GPL14604	WB	USA	Patients in the acute phase of a viral challenge study	Outpatient		HRV	20	0	0	0	8
GSE73072 (HRV DUKE)	Liu	GPL14604	WB	USA	Patients in the acute phase of a viral challenge study	Outpatient		HRV	26	0	0	0	11
GSE9692	Cvijanovich	GPL570	WB	USA	Children with septic shock	PICU	*S. aureus*, *Streptococcus* spp., *Neisseria* spp.	Adenovirus	15	13	0	0	1
GSE19491	Berry	GPL6947	WB	UK, South Africa	Patients with febrile bacterial infection	Unknown	*S. pyogenes*, *Staphylococcus* spp.		18	52	0	0	0
GSE68004	Jaggi	GPL10558	WB	USA	Children with bacterial or viral infection	Inpatient	*S. pyogenes*	Adenovirus	37	14	0	0	19
GSE27131	Berdal	GPL6244	WB	Norway	Adults with influenza	ICU		Influenza	7	0	0	0	7

GSE38246	Popper	GPL15615	PBMC	Nicaragua	Children hospitalized with dengue infection	Inpatient		Dengue	8	0	0	0	105
GSE18090	Nascimento	GPL570	PBMC	Brazil	Adults with early acute febrile dengue infection	Inpatient		Dengue	8	0	0	0	18
GSE6269 GPL96	Ramilo	GPL96	PBMC	USA	Children with bacterial or viral sepsis	ED	*E. coli* , *S. aureus* , *S. pneumoniae*	Influenza	6	73	0	0	18
GSE34205	Ioannidis	GPL570	PBMC	USA	Children with ARIs	Inpatient		Influenza, RSV	22	0	0	0	79
GSE23140	Liu	GPL6254	PBMC	USA	Children with acute otitis media	Inpatient	*S. pneumoniae*		4	4	0	0	0
GSE16129 GPL6106	Ardura	GPL6106	PBMC	USA	Children with invasive staph infections	Inpatient	*S. aureus*		9	9	0	0	0
GSE16129 GPL96	Ardura	GPL96	PBMC	USA	Children with invasive staph infections	Inpatient	*S. aureus*		10	46	0	0	0
GSE16463	Tantibhedhy angkul	GPL6102	PBMC	Thailand	Patients with rickettsial or dengue infection	Inpatient	*R. typhi* , *O. tsutsugamushi*	Dengue	2	0	11	0	7
GSE11907 GPL96	Chaussabel	GPL96	PBMC	USA	Patients with bacterial infection	Unknown	*E. coli* , *S. aureus*		12	40	0	0	0

Table S3: Demographic characteristics of the subjects used for signature training, hold-out validation, and independent validation, related to Figure 1 and 2.

Age and sex characteristics across datasets, for each analysis phase. The first table summarizes both healthy samples and samples with infection, and the second table summarizes just the samples with infection. WB, whole blood; PBMC, peripheral blood mononuclear cells.

All samples (includes healthy controls)		Training	Hold-out Validation	Independent Validation (COCONUT)	Independent Validation (Individual)	Total
	Number of Datasets	28	22	24	4	
	Number of Samples	1678	633	1458	431	4200
	≤5 years old	684	271	332	30	1317
	>5 and ≤18 years old	39	14	202	16	271
	>18 and ≤65 years old	164	45	100	0	309
	>65 years old	48	12	15	0	75
	Unknown age	743	291	809	385	2228
	Female	699	274	675	214	1862
	Male	979	359	783	217	2338

Only samples with infection		Training	Hold-out Validation	Independent Validation (COCONUT)	Independent Validation (Individual)	Total
	Number of Datasets	28	22	24	4	
	Number of Samples	1175	435	961	431	3002
	≤5 years old	530	213	284	30	1057
	>5 and ≤18 years old	32	8	154	16	210
	>18 and ≤65 years old	107	25	53	0	185
	>65 years old	39	8	14	0	61
	Unknown age	467	181	456	385	1489
	Female	490	176	444	214	1324
	Male	685	259	517	217	1678

Table S4: Infecting pathogen reported for the subjects used in training, hold-out validation, and independent validation, related to Figure 2.

RSV, respiratory syncytial virus; CoNS, coagulase-negative staphylococci; HHV6, human herpesvirus 6; CMV, cytomegalovirus; HSV, herpes simplex virus; BKV, BK virus; HMPV, human

Pathogen	Pathogen Category	Training	Hold-out Validation	Independent Validation (COCONUT)	Independent Validation (Individual)
B. pseudomallei	Intracellular bacteria	74	17		
***Brucella* spp.**	Intracellular bacteria	72	31		
S. typhi	Intracellular bacteria	35	19	38	
O. tsutsugamushi	Intracellular bacteria	9	3	4	
R. typhi	Intracellular bacteria	9	2	7	
***Salmonella* spp.**	Intracellular bacteria	4	2		
L. monocytogenes	Intracellular bacteria	1			
***Rickettsia* spp.**	Intracellular bacteria	1	1		
S. enterica	Intracellular bacteria			2	
S. aureus	Extracellular bacteria	53	23	208	19
E. coli	Extracellular bacteria	37	10	51	
S. pneumoniae	Extracellular bacteria	23	12	31	9
**CoNS**	Extracellular bacteria	17	2		
***Leptospira* spp.**	Extracellular bacteria	16			
S. pyogenes	Extracellular bacteria	15	7	28	
K. pneumoniae	Extracellular bacteria	14	3		
S. agalactiae	Extracellular bacteria	13	5	2	
***Enterococcus* spp.**	Extracellular bacteria	11	4		
N. meningitidis	Extracellular bacteria	9	2	24	
**Viridans Streptococci**	Extracellular bacteria	8			
***Corynebacterium* spp.**	Extracellular bacteria	6	1		
**Enterovirus/Rhinovirus**	Extracellular bacteria	6	2		

***Neisseria* spp.**	Extracellular bacteria	6	1	2	
H. Influenzae	Extracellular bacteria	4	2		
S. marcescens	Extracellular bacteria	4			
A. baumannii	Extracellular bacteria	3	1		
P. aeruginosa	Extracellular bacteria	3			
S. epidermidis	Extracellular bacteria	3			
***Aeromonas* spp.**	Extracellular bacteria	2			
E. faecalis	Extracellular bacteria	2			
E. faecium	Extracellular bacteria	2			
***Micrococcus* spp.**	Extracellular bacteria	2	2		
A. hydrophila	Extracellular bacteria	1			
A. lwoffii	Extracellular bacteria	1	1		
***Acinetobacter* spp.**	Extracellular bacteria	1			
C. difficile	Extracellular bacteria	1	1		
C. freundii	Extracellular bacteria	1			
E. corrodens	Extracellular bacteria	1	1		
***Enterobacter* spp.**	Extracellular bacteria	1	2		
M. catarrhalis	Extracellular bacteria	1			
***Pseudomonas* spp.**	Extracellular bacteria	1	1		
S. anginosus	Extracellular bacteria	1			
S. suis	Extracellular bacteria	1			
E. cloacae	Extracellular bacteria		1		
K. oxytoca	Extracellular bacteria		1		
***Sphingomonas* spp.**	Extracellular bacteria		1		
***Staphylococcus* spp.**	Extracellular bacteria			40	
**Unspecified bacteria**	Unspecified bacteria	116	49		148
**Gram Positive Bacteria**	Unspecified bacteria	14	4		
**Gram Negative Bacteria**	Unspecified bacteria	4	4		
**RSV**	Virus	178	63	170	
**Rhinovirus**	Virus	118	53	29	
**Influenza**	Virus	115	46	116	18

**Dengue**	Virus	74	23	189	
**Enterovirus**	Virus	39	19		
**Influenza/Rhinovirus**	Virus	15	4		
**HHV6**	Virus	8			
**Adenovirus**	Virus	5	4	20	
**CMV**	Virus	3	2		
**HSV**	Virus	3	2		
**Rotavirus**	Virus	2			
**Varicella**	Virus	2			
**BKV**	Virus	1			
**Coronavirus**	Virus	1	1		
**HMPV**	Virus	1			
**Parainfluenza**	Virus	1			
**Unspecified virus**	Virus				237

Table S5: Demographic and clinical characteristics of the subjects from the Laos Microarray cohort, related to Figure 1.

Slide	ID	Diagnosis	Age	Sex	Days since fever onset
HOAD086	10205	Dengue	20	Male	5
HOAD119	10262	Dengue	24	Male	5
HOAD159	10307	Dengue	32	Female	3
HOAD167	10371	Dengue	19	Female	5
HOAD100	10424	Dengue	17	Male	6
HOAD093	10528	Dengue	20	Male	8
HOAD124	10607	Dengue	20	Female	4
HOAD084	10608	Dengue	21	Male	6
HOAD162	10637	Dengue	23	Female	5
HOAD117	10639	Dengue	19	Male	4
HOAD082	10663	Dengue	24	Female	5
HOAD128	10669	Dengue	23	Female	6
HOAD127	10672	Dengue	23	Female	7
HOAD135	10122	*E. coli*	74	Female	4
HOAD090	10240	*E. coli*	80	Female	2
HOAD160	10374	*E. coli*	68	Female	4
HOAD165	10437	*E. coli*	62	Female	6
HOAD122	11066	*E. coli*	20	Female	4
HOAD106	11474	*E. coli*	25	Female	4
HOAD140	BDC22	Healthy	NA	NA	NA
HOAD169	BDC23	Healthy	NA	NA	NA
HOAD110	BDC26A	Healthy	NA	NA	NA
HOAD112	BDC27	Healthy	NA	NA	NA
HOAD144	BDC29	Healthy	NA	NA	NA
HOAD173	BDC31	Healthy	NA	NA	NA
HOAD147	BDC33	Healthy	NA	NA	NA
HOAD107	BDC35	Healthy	NA	NA	NA
HOAD101	BDC37	Healthy	NA	NA	NA

HOAD172	BDC38	Healthy	NA	NA	NA
HOAD109	BDC39A	Healthy	NA	NA	NA
HOAD168	BDC40	Healthy	NA	NA	NA
HOAD102	10039	*Orientia tsutsugamushi*	60	Female	5
HOAD095	10375	*Orientia tsutsugamushi*	28	Male	7
HOAD158	10532	*Orientia tsutsugamushi*	30	Male	10
HOAD149	10584	*Orientia tsutsugamushi*	27	Male	12
HOAD096	10653	*Orientia tsutsugamushi*	18	Female	11
HOAD089	11106	*Orientia tsutsugamushi*	50	Female	14
HOAD164	11114	*Orientia tsutsugamushi*	31	Female	11
HOAD091	10654A	*Orientia tsutsugamushi*	23	Female	9
HOAD138	N058	*Orientia tsutsugamushi*	NA	NA	NA
HOAD103	N248	*Orientia tsutsugamushi*	NA	NA	NA
HOAD145	N250	*Orientia tsutsugamushi*	NA	NA	NA
HOAD139	N363	*Orientia tsutsugamushi*	NA	NA	NA
HOAD161	10191	*Rickettsia typhi*	44	Female	7
HOAD130	10551	*Rickettsia typhi*	46	Male	6
HOAD081	11184	*Rickettsia typhi*	25	Male	12
HOAD132	11357	*Rickettsia typhi*	18	Female	6
HOAD136	11452	*Rickettsia typhi*	22	Female	9
HOAD088	11379A	*Rickettsia typhi*	30	Female	4
HOAD171	N120	*Rickettsia typhi*	NA	NA	NA
HOAD137	N240	*Rickettsia typhi*	NA	NA	NA
HOAD143	N298	*Rickettsia typhi*	NA	NA	NA
HOAD166	N303	*Rickettsia typhi*	NA	NA	NA
HOAD108	N397	*Rickettsia typhi*	NA	NA	NA
HOAD129	9774	*S. typhi*	20	Male	6
HOAD131	10259	*S. typhi*	37	Male	15
HOAD142	N102	*S. typhi*	NA	NA	NA
HOAD146	N259	*S. typhi*	NA	NA	NA
HOAD163	N367	*S. typhi*	NA	NA	NA
HOAD111	N372	*S. typhi*	NA	NA	NA
HOAD104	N387	*S. typhi*	NA	NA	NA

Table S6: Individual datasets used for independent validation of the BoVI signature, related to Figure 3.

WB, whole blood; PBMC, peripheral blood mononuclear cells; ARI, acute respiratory infection; PICU, pediatric intensive care unit; ED, emergency department

Accession	Author	Platform	Tissue	Location	Demographic	Clinical setting(s)	Bacteria	Viruses	# of healthy samples	# of extracellular bacterial samples	# of intracellular bacterial samples	# of unknown bacterial	# of viral samples
GSE72809	Herberg	GPL10558	WB	UK, Spain, Netherland, USA	Children <17 years old with bacterial or viral infections	Inpatient, PICU	Unknown	Unknown	0	0	0	52	92
GSE72810	Herberg	GPL6947	WB	UK, Spain, Netherland, USA	Children <17 years old with bacterial or viral infections	Inpatient, PICU	Unknown	Unknown	0	0	0	23	28
GSE63990	Tsalik	GPL571	WB	USA	Patients with ARIs	ED, Inpatient	Unknown	Unknown	0	0	0	73	117
GSE6269 GPL570	Ramilo	GPL570	PBMC	USA	Children with bacterial or viral sepsis	ED	*S. aureus, S. pneumoniae*	Influenza	0	12	0	0	10
GSE6269 GPL2507	Ramilo	GPL2507	PBMC	USA	Children with bacterial or viral sepsis	ED	*S. aureus, S. pneumoniae*	Influenza	0	16	0	0	8

Table S7: Assessment of promising signatures in the training and hold-out validation sets, related to Figure 2.
Performance of the differential expression filters and corresponding feature selection methods that were used to identify potential signatures. The signature that was chosen for independent validation is highlighted in green. AUROC, area under the receiving operator characteristics curve; ES, effect size; FDR, false discovery rate; abridged BSS, Abridged Best Subset Selection; min.1ci, minimum AUROC within the confidence interval of the maximum AUROC.

**Differential expression thresholds**	**Feature selection method**	**Number of genes**	**Training**			**Hold-out Validation**		
			**All bacterial vs. viral**	**EC bacterial vs. viral**	**IC bacterial vs. viral**	**All bacterial vs. viral**	**EC bacterial vs. viral**	**IC bacterial vs. viral**
ES ≥ 0.6; FDR ≤ 1%	N/A	417	0.864 (0.843-0.885)	0.945 (0.926-0.965)	0.723 (0.679-0.766)	0.845 (0.808-0.882)	0.937 (0.9-0.974)	0.725 (0.654-0.796)
	Forward Search	20	0.962 (0.951-0.973)	0.974 (0.96-0.988)	0.953 (0.933-0.974)	0.94 (0.917-0.963)	0.964 (0.936-0.993)	0.937 (0.898-0.976)
	Backward Search	44	0.968 (0.958-0.978)	0.979 (0.966-0.991)	0.96 (0.941-0.979)	0.953 (0.932-0.973)	0.975 (0.952-0.999)	0.947 (0.911-0.983)
	Backward Search + abridged BSS (max AUROC)	38	0.968 (0.958-0.978)	0.978 (0.966-0.991)	0.961 (0.943-0.98)	0.953 (0.933-0.974)	0.975 (0.952-0.999)	0.952 (0.918-0.986)
	Backward Search + abridged BSS (min.1ci AUROC)	15	0.959 (0.947-0.97)	0.964 (0.948-0.98)	0.956 (0.935-0.976)	0.95 (0.928-0.971)	0.96 (0.93-0.99)	0.957 (0.925-0.99)
ES ≥ 0.8; FDR ≤ 1%	N/A	76	0.89 (0.871-0.909)	0.953 (0.935-0.971)	0.773 (0.733-0.814)	0.904 (0.874-0.933)	0.957 (0.927-0.988)	0.83 (0.769-0.89)
	Forward Search	14	0.942 (0.928-0.955)	0.96 (0.943-0.977)	0.918 (0.891-0.944)	0.93 (0.905-0.955)	0.953 (0.921-0.986)	0.924 (0.882-0.967)
	Backward Search	19	0.943 (0.93-0.957)	0.962 (0.945-0.978)	0.913 (0.886-0.94)	0.941 (0.918-0.964)	0.959 (0.921-0.989)	0.942 (0.904-0.979)
	Backward Search + abridged BSS (max AUROC)	18	0.943 (0.929-0.957)	0.96 (0.943-0.977)	0.913 (0.886-0.943)	0.94 (0.917-0.964)	0.956 (0.925-0.988)	0.942 (0.905-0.98)
	Backward Search + abridged BSS (min.1ci AUROC)	6	0.934 (0.919-0.949)	0.951 (0.932-0.969)	0.914 (0.886-0.941)	0.928 (0.903-0.954)	0.956 (0.925-0.987)	0.919 (0.875-0.963)
Top 100 SAM scores	N/A	100	0.874 (0.854-0.894)	0.944 (0.924-0.964)	0.742 (0.699-0.784)	0.877 (0.844-0.911)	0.948 (0.914-0.982)	0.773 (0.706-0.841)
	Forward Search	15	0.951 (0.938-0.963)	0.968 (0.952-0.983)	0.925 (0.9-0.951)	0.947 (0.925-0.969)	0.965 (0.937-0.993)	0.944 (0.907-0.981)
	Backward Search	16	0.951 (0.939-0.964)	0.966 (0.95-0.981)	0.932 (0.908-0.957)	0.946 (0.924-0.968)	0.967 (0.939-0.994)	0.94 (0.902-0.978)
	Backward Search + abridged BSS (max AUROC)	15	0.951 (0.939-0.964)	0.967 (0.952-0.983)	0.928 (0.903-0.953)	0.948 (0.926-0.969)	0.967 (0.939-0.994)	0.944 (0.907-0.981)
	**Backward Search + abridged BSS (min.1ci AUROC)**	8	**0.942 (0.928-0.955)**	**0.958 (0.941-0.975)**	**0.92 (0.894-0.947)**	**0.947 (0.925-0.969)**	**0.965 (0.938-0.993)**	**0.941 (0.904-0.979)**

Table S8: Active TB and HIV datasets used for validation of the BoVI signature, related to Figure 3.
Active TB samples were excluded if they exhibited an atypical presentation of TB (e.g. extrapulmonary TB), were HIV positive or had an ambiguous HIV status, had any other significant comorbidities, or had been successfully treated for TB. HIV samples were excluded if they were undergoing HIV treatment. Any datasets that did not contain all 8 genes in the BoVI signature were excluded. TB, tuberculosis; HIV, human immunodeficiency virus; WB, whole blood; PBMC, peripheral blood mononuclear cells.

Accession	Author	Platform	Tissue	Location	Demographic	Clinical setting(s)	Pathogen	# of healthy samples	# of bacterial samples	# of viral samples
GSE42834	Bloom	GPL10558	WB	UK, France, South Africa	Adults with TB,	Inpatient	TB	118	40	0
GSE19491	Berry	GPL6947	WB	UK, South Africa	Patients with TB or other febrile conditions	Inpatient, outpatient	TB	18	61	0
GSE28623	Maertzdorf	GPL4133	WB	The Gambia	Adults with TB	Outpatient	TB	37	46	0
GSE34608	Maertzdorf	GPL6480	WB	Germany	Adults with TB	Outpatient	TB	18	8	0
GSE41055	Verhagen	GPL5175	WB	Venezuela	Children with ATB or LTBI	Outpatient	TB	9	2	0
GSE83456	Blankley	GPL10558	WB	UK	Adults with TB	Inpatient	TB	61	45	0
GSE81746	Sambarey	GPL17077	WB	India	Adults with TB	Unknown	TB	2	4	0
E-GEOD-25534	Maertzdorf	GPL1708	WB	South Africa	Patients with TB	Inpatient, outpatient	TB	13	44	0
E-MTAB-3260	Noursadeghi	GPL14550	WB	Unknown	Patients with ATB	Inpatient, outpatient	TB	11	16	0
E-MTAB-8290	Turner	GPL18573	WB	South Africa	Adults presenting with possible pulmonary TB	Outpatient	TB	99	37	0

GSE100150	Altman	GPL6884	WB	USA, UK, Finland, Spain	Patients with a variety of clinical conditions	Unknown	TB	11	23	0
GSE114192	Eckold	GPL18573	WB	South Africa, Romania, Indonesia, Peru	TB patients with diabetes comorbidity	Outpatient	TB	36	46	0
GSE122485	Sambarey	GPL15520	WB	India	Adults with TB	Outpatient	TB	3	4	0
GSE107995	Singhania	GPL20301	WB	India, UK, Kenya, Somalia, Afghanistan, Uganda, Sudan, Tanzania, Pakistan, Poland, etc.	Individuals with ATB and LTBI	Inpatient	TB	119	53	0
GSE100150	Altman	GPL6884	WB	USA	Adult patients diagnosed with HIV infection	Unknown	HIV	35	0	28
GSE29429 GPL10558	Chaussabel	GPL10558	WB	Africa	Adult patients diagnosed with acute HIV infection	Unknown	HIV	17	0	30
GSE29429 GPL6947	Chaussabel	GPL6947	WB	USA, Africa	Adult patients diagnosed with acute HIV infection	Unknown	HIV	38	0	28
GSE29536	Banchereau	GPL6102	WB	USA	Patients diagnosed with acute HIV infection	HIV testing sites	HIV	36	0	9
GSE4124	Montano	GPL571	PBMC	Botswana	HIV+ mothers	Outpatient	HIV	20	0	25

GSE77939	Singh	GPL15207	WB	India	Patients diagnosed with HIV infection	Outpatient	HIV	4	0	5

Table S9: Multivariable logistic regression of BoVI score, age, sex, and race, related to Figure 4 and Figure S7-S9.

*, **, *** indicates significance at p<0.05, p<0.01, and p<0.001, respectively.

	Estimate	Standard error	Z-score	P value
**Intercept**	-0.485	0.414	-1.17	0.242
**BoVI score**	3.703	0.231	16.006	<2e-16***
**Age**	0.011	0.005	2.344	0.019*
**Sex (Male)**	0.067	0.197	0.342	0.732
**Race (Asian)**	0.892	0.552	1.615	0.106
**Race (Black)**	1.344	0.448	3.002	0.003**
**Race (White)**	0.313	0.431	0.728	0.466

Table S10: Demographic and clinical characteristics of the subjects from the Laos Fluidigm prospective validation cohort, related to Figure 5 and Figure S11.
JEV; Japanese encephalitis virus.

ID	Sponsor ID	Diagnosis	Age	Sex	Days since fever onset	Sweeney7 Score	BoVI Score
A3927_047	9718	*Rickettsia* sp.	49	female	9	-0.2842763	1.36086713
A3927_048	9787	Dengue	16	female	3	-4.5055377	-4.135445
A3927_049	9788	Dengue	26	male	3	-3.8083701	-3.633376
A3927_050	9806	JEV	19	male	8	-0.0015691	-0.1778702
A3927_051	9812	Dengue	20	female	3	-5.8282189	-4.462533
A3927_052	9819	Dengue	19	male	4	-4.1586179	-2.7141281
A3927_053	9831	Dengue	21	female	11	-0.6100248	-0.4813855
A3927_054	9850	Dengue	21	female	4	-4.7359659	-3.7685813
A3927_055	9875	Dengue	33	male	4	-4.4376922	-3.3014833
A3927_056	9878	Dengue	20	female	4	-3.7528465	-2.518329
A3927_057	9882	*Leptospira* sp.	32	male	30	0.89918285	1.75503654
A3927_058	9883	Dengue	16	male	14	-4.3279038	-3.7107889
A3927_059	9887	*Leptospira* sp.	75	female	1	0.77921931	0.45155862
A3927_060	9904	*O. tsutsugamushi*	20	male	1	-0.8374049	0.18251192
A3927_061	9905	Dengue	17	female	13	-5.5880167	-3.5077974
A3927_062	9910	Dengue	23	female	5	-3.6568906	-2.9977703
A3927_063	9917	*Leptospira* sp.	26	male	7	-0.7509469	-0.2045696
A3927_064	9922	Dengue	16	male	5	-4.1960872	-3.500152
A3927_065	9923	Dengue	50	female	3	-5.1648068	-4.2582222
A3927_066	9948	*E. coli*	28	female	5	2.5847888	4.02212649
A3927_067	9949	Dengue	19	female	7	-4.1496766	-3.3656053
A3927_068	9954	Dengue	25	male	5	-3.8874533	-2.6515083
A3927_069	9967	Dengue	19	female	2	-4.4789349	-4.2006006
A3927_070	9980	Dengue	19	male	6	-3.5164916	-2.7146446
A3927_071	9989	*B. pseudomallei*	68	male	14	2.57218257	3.53341337
A3927_072	9997	Dengue	36	male	6	-4.9263644	-4.5716664
A3927_073	10040	*O. tsutsugamushi*	65	female	5	-2.0295993	0.07732783
A3927_075	10045	Dengue	22	male	3	-4.2149842	-3.0465148
A3927_076	10046	Dengue	17	female	8	-4.4877463	-2.8077175
A3927_077	10052	Dengue	23	male	6	-1.1849604	0.68127555
A3927_078	10067	Dengue	29	female	4	-5.5081609	-3.983312
A3927_080	10084	Dengue	16	female	5	-4.2470734	-3.5888749
A3927_081	10086	Dengue	31	male	4	-4.0065337	-2.971861
A3927_082	10090	*Leptospira* sp.	30	female	5	-2.0361731	-1.6176782

A3927_083	10092	Dengue	17	female	6	-4.6525075	-3.6791776
A3927_084	10095	Dengue	16	male	6	-4.300987	-2.9086558
A3927_085	10113	Dengue	21	female	4	-4.6290395	-3.3002791
A3927_086	10135	Dengue	19	female	5	-6.301492	-2.8401764
A3927_087	10141	Dengue	22	male	5	-4.0997869	-3.4965484
A3927_088	10151	Dengue	23	female	3	-6.1992554	-4.493835
A3927_089	10153	Dengue	16	male	6	-4.1479984	-3.478402
A3927_090	10179	Dengue	18	female	3	-4.055814	-3.6643872
A3927_091	10183	Dengue	46	female	2	-4.3256663	-4.1770774
A3927_092	10184	Dengue	26	female	7	-4.3524515	-3.1708999
A3927_093	10186	Dengue	35	male	4	-4.0783841	-2.8054071
A3927_094	10195	Dengue	22	male	4	-3.7983135	-3.0740389
A3927_095	10203	Dengue	17	female	8	-4.0067396	-4.1001964
A3927_096	10217	Dengue	56	female	3	-1.4357707	-0.2570482
A3927_097	10227	Dengue	19	female	3	-4.5347737	-4.3002903
A3927_098	10231	Dengue	21	male	6	-3.9751261	-3.0535746
A3927_099	10232	Dengue	19	female	7	-4.1229678	-3.7857059
A3927_100	10233	Dengue	32	male	5	-3.8024054	-2.9473052
A3927_101	10237	Dengue	17	female	6	-4.7077025	-2.9432092
A3927_102	10239	Dengue	16	male	5	-3.6883384	-3.6664561
A3927_103	10241	Group B Streptococcus	65	male	5	1.13771676	3.01747692
A3927_104	10248	Dengue	30	male	4	-5.4214761	-3.480253
A3927_105	10249	Dengue	22	female	5	-4.7045711	-3.8841779
A3927_106	10251	Dengue	18	male	4	-4.1115379	-3.5632562
A3927_107	10273	Dengue	21	male	8	-3.1020946	-2.9902377
A3927_108	10274	Dengue	22	female	4	-4.7045008	-3.7467694
A3927_109	10276	Dengue	26	female	4	-5.5829015	-3.8923438
A3927_110	10279	Dengue	33	male	4	-3.5809608	-2.9977932
A3927_111	10288	Dengue	25	female	1	-4.2751769	-2.9325343
A3927_112	10293	*Leptospira* sp.	25	male	4	-0.1836041	1.1624809
A3927_113	10320	Dengue	23	male	4	-3.0891238	-3.226352
A3927_114	10321	Dengue	24	female	4	-4.4235776	-3.456484
A3927_115	10328	Dengue	17	female	3	-4.1718115	-3.0192062
A3927_116	10329	Dengue	23	male	5	-5.4867136	-4.1919911
A3927_118	10342	Dengue	20	male	4	-4.1309704	-3.2435833
A3927_119	10344	Dengue	23	female	5	-4.7699874	-3.6302574
A3927_120	10347	Dengue	30	male	5	-4.0124122	-3.691171
A3927_121	10348	Dengue	17	male	3	-4.6413426	-4.1914877
A3927_122	10378	Dengue	19	female	4	-4.2840491	-3.1607955
A3927_123	10379	Dengue	18	female	7	-4.0139017	-3.9830861

A3927_124	10394	Dengue	16	female	5	-4.5486072	-3.2067688
A3927_125	10410	Dengue	37	male	0	-4.614831	-3.80356
A3927_126	10423	Dengue	24	female	4	-5.716688	-3.7623149
A3927_127	10426	Dengue	32	male	4	-3.824037	-3.3528182
A3927_128	10438	Dengue	24	male	4	-4.1957231	-3.5803347
A3927_129	10439	Dengue	26	female	5	-3.3921763	-3.0867067
A3927_130	10441	Dengue	19	male	3	-4.3955831	-3.8022082
A3927_131	10444	Dengue	50	male	12	0.49239044	-0.5705381
A3927_132	10447	*Leptospira* sp.	35	female	17	1.19056242	0.79741472
A3927_133	10451	Dengue	21	male	5	-3.1875275	-3.5418447
A3927_134	10465	*Leptospira* sp.	16	female	14	-4.2672681	-3.4863745
A3927_135	10476	Dengue	24	male	4	-3.4447348	-2.2719929
A3927_136	10478	Dengue	17	male	4	-3.7322377	-3.7170191
A3927_137	10479	Dengue	20	male	4	-3.9731906	-3.627635
A3927_138	10480	Dengue	26	female	12	-2.65517	-1.4991074
A3927_139	10481	Dengue	21	male	6	-3.5780821	-2.7416055
A3927_140	10489	Dengue	29	male	9	0.29060053	2.35418291
A3927_141	10503	Dengue	23	male	5	-4.9412412	-3.1984048
A3927_142	10509	Dengue	16	male	6	-5.2735293	-3.1895555
A3927_143	10510	Dengue	24	male	7	-4.063949	-2.8904876
A3927_144	10511	Flavivirus	67	female	5	-2.9067966	-0.9588956
A3927_145	10513	Dengue	28	male	6	0.52094256	2.67524425
A3927_146	10514	Dengue	18	female	6	-4.8199451	-3.7011998
A3927_147	10515	Dengue	19	female	1	-4.3733874	-3.8601736
A3927_148	10516	Dengue	30	female	6	-5.300171	-3.5965722
A3927_149	10526	Dengue	17	female	5	-4.4309173	-3.1514213
A3927_150	10527	Dengue	33	female	5	-2.4505906	-1.9033776
A3927_151	10529	Dengue	27	female	5	-4.4133988	-2.6392044
A3927_152	10536	Dengue	24	male	6	-4.0224486	-2.5519584
A3927_153	10537	Dengue	24	male	3	-4.6853943	-3.6493829
A3927_154	10542	Dengue	16	male	5	-4.3440396	-3.6762824
A3927_155	10543	Dengue	20	female	4	-4.9890436	-4.0315129
A3927_156	10549	Dengue	24	male	5	-4.4501951	-3.5212294
A3927_157	10550	Dengue	21	male	6	-4.3613183	-3.1998903
A3927_158	10557	Dengue	19	female	3	-4.9256874	-3.4687698
A3927_159	10570	Dengue	21	female	5	-4.4409013	-3.6485339
A3927_160	10573	Dengue	22	male	6	-4.7983169	-3.4013833
A3927_161	10581	Dengue	23	female	2	-5.0993293	-3.1842701
A3927_162	10590	Dengue	24	male	5	-3.7984137	-3.5880525
A3927_163	10591	Salmonella Group B	75	female	1	0.7402411	1.68932446

A3927_164	10609	Dengue	17	male	3	-4.8228601	-3.1758097
A3927_165	10630	*E. coli*	67	female	9	0.85883914	1.64054443
A3927_166	10656	Dengue	22	female	7	-5.3498392	-3.6301059
A3927_167	10670	Dengue	18	male	4	-4.4679868	-3.1880643
A3927_169	10950	Dengue	27	male	5	-4.7657594	-3.4352387
A3927_170	10955	Dengue	20	female	3	-5.2901359	-3.5818298
A3927_171	10962	Dengue	30	female	5	-3.9687157	-3.1281607
A3927_172	10967	Dengue	27	female	4	-1.2336534	0.31947886
A3927_173	10979	Dengue	18	male	5	-4.5916658	-3.515949
A3927_174	10984	Dengue	27	male	4	-4.6327415	-2.6331445
A3927_175	11031	Dengue	18	male	4	-4.0892495	-2.9318089
A3927_176	11041	Dengue	23	male	4	-4.7437689	-2.9202957
A3927_177	11051	Dengue	18	male	5	-5.1329932	-3.0226404
A3927_178	11068	Dengue	31	female	4	-2.1190547	-0.3841236
A3927_180	11074	*Leptospira* sp.	21	female	4	0.35185772	1.66150753
A3927_181	11075	Dengue	20	male	6	-4.2496686	-3.0954013
A3927_182	11076	*Leptospira* sp.	68	female	5	0.67988926	1.27938729
A3927_183	11083	Dengue	16	male	3	-4.8285382	-3.270098
A3927_184	11084	Dengue	38	female	5	-4.9039075	-3.3484396
A3927_185	11087	Dengue	24	male	5	-4.856111	-3.1017652
A3927_186	11104	Dengue	26	male	5	-4.1983643	-3.3501246
A3927_187	11119	Dengue	25	male	4	-5.1314106	-3.9308003
A3927_188	11142	*S. typhi*	18	male	15	-1.0659978	0.8381991
A3927_189	11145	*S. aureus*	20	female	1	0.53365817	1.38131038
A3927_190	11147	Dengue	25	female	7	-3.822148	-2.2039227
A3927_191	11212	Dengue	30	female	2	1.92460644	0.58289779
A3927_192	11240	*Leptospira* sp.	52	female	7	-0.9575956	0.54029979
A3927_193	11243	JEV	20	male	7	-1.5907234	0.62872443
A3927_194	11246	*Leptospira* sp.	40	male	7	2.20900389	2.84546327
A3927_195	11304	Dengue	28	male	4	-4.9883793	-3.2050754
A3927_196	11343	*S. aureus*	18	male	4	-0.2085599	0.39270775
A3927_197	11362	Dengue	64	female	2	0.53261846	1.81559899
A3927_198	11368	*Rickettsia* sp.	30	female	10	-0.7027827	1.60631459
A3927_199	11441	*Leptospira* sp.	26	female	2	-3.0345107	-1.69988
A3927_200	11467	*S. typhi*	24	female	1	-1.1531873	0.62502306
A3927_201	11564	*E. coli*	70	female	5	1.12967343	2.28822574
A3927_202	11567	*S. typhi*	33	female	13	-1.0072912	0.92565394
A3927_203	11579	*S. typhi*	24	female	3	-1.6375075	0.21287683
A3927_204	11582	*S. typhi*	36	female	5	-1.2200625	1.92501691
A3927_205	11584	*S. typhi*	27	male	6	-1.5057356	1.20901186

A3927_206	11614	*Salmonella* sp.	26	female	6	-1.3025954	1.37032192
A3927_207	11620	*E. coli*	61	female	3	2.28093658	3.42002081
A3927_208	11633	*E. coli*	76	female	3	-1.6263572	0.75229182
A3927_209	11655	*S. typhi*	26	male	14	-0.0725324	2.06721521
A3927_210	11659	*S. typhi*	38	male	7	-1.4125439	0.44762989
A3927_211	11666	*S. pneumoniae*	24	male	80	-1.3889904	-0.7265846
A3927_212	11670	*S. typhi*	18	male	3	-3.7272969	-2.5497606
A3927_214	11705	*S. typhi*	19	male	5	-1.135101	0.11199716
A3927_215	11712	*S. typhi*	28	female	7	-1.633431	1.48055402
A3927_216	11744	*E. coli*	26	female	3	-0.5867071	0.63865616
A3927_217	11759	*E. coli*	45	male	0	0.8471719	2.12593253
A3927_218	11770	*K. pneumoniae*	77	male	4	3.00157554	3.75193157
A3927_219	12108	*B. pseudomallei*	56	female	3	2.17134576	2.7679059
A3927_222	12123	*S. typhi*	42	male	15	-1.2246822	-0.2164049
A3927_223	12124	*O. tsutsugamushi*	23	male	15	-0.8035127	0.89797054
A3927_224	12136	*Salmonella* sp.	63	female	7	3.51267616	5.05413188
A3927_225	12171	*E. coli*	16	male	5	0.63284021	1.22290046
A3927_226	12173	*O. tsutsugamushi*	50	female	6	-1.382469	-0.2449159
A3927_227	12179	*Moraxella* sp.	42	male	7	-1.4765506	0.43249978
A3927_229	12183	*O. tsutsugamushi*	46	male	4	-1.7997418	-0.4794779
A3927_230	12233	*O. tsutsugamushi*	42	male	7	-1.0591502	0.59564239
A3927_231	12268	*Leptospira* sp.	20	male	2	2.47822385	2.26163316
A3927_232	12283	*S. typhi*	43	male	14	0.30259321	2.803426
A3927_233	12288	Group G Streptococcus	22	male	34	-3.666012	-2.3129063
A3927_234	12291	*W. paucula*	17	male	5	-2.6244924	-0.8999069
A3927_235	12299	*S. typhi*	30	female	10	-1.0689172	0.89164264
A3927_236	12342	*Leptospira* sp.	38	male	2	3.90388738	4.36180575
A3927_237	12353	*E. coli*	55	female	2	3.1884651	2.84828033
A3927_238	12354	*E. coli*	35	female	2	4.3666286	5.42320768
A3927_239	12407	*O. tsutsugamushi*	25	female	6	-1.7210431	1.00187452
A3927_240	12441	*O. tsutsugamushi*	31	male	8	-0.0524469	2.83037616
A3927_241	12443	*O. tsutsugamushi*	25	male	4	-0.393036	2.95342342
A3927_242	12456	*S. typhi*	36	female	4	-1.3189474	0.12037628
A3927_243	12468	*Rickettsia* sp.	20	female	2	-3.6344279	-2.9278346
A3927_244	12646	*E. coli*	61	male	2	2.42024431	4.97150091
A3927_246	12657	*B. pseudomallei*	46	female	5	0.15965792	2.62399401
A3927_247	12674	*K. pneumoniae*	76	male	4	3.06772443	4.39873173
A3927_248	12697	*S. typhi*	19	female	8	-1.2814548	0.83892971
A3927_250	12822	*Salmonella* sp.	42	female	3	-1.1160205	1.06652316
A3927_251	12828	*O. tsutsugamushi*	30	male	7	-0.8884574	0.9742752

A3927_252	12852	*S. typhi*	16	male	13	-1.2056229	1.79651309
A3927_253	12881	*S. typhi*	25	male	9	-1.5602695	0.66348972
A3927_254	12931	*E. coli*	30	male	4	4.11754219	4.49562445
A3927_255	12986	*O. tsutsugamushi*	30	male	11	-0.9617764	3.40731278
A3927_256	12996	*E. coli*	60	female	7	1.86391068	2.41566424
A3927_258	13037	*Rickettsia* sp.	18	female	7	-2.0307133	-0.3530427
A3927_259	13043	*E. coli*	73	male	6	0.39648477	2.45709271
A3927_260	13077	*E. coli*	40	female	7	2.50582929	3.01299696
A3927_261	13095	*O. tsutsugamushi*	18	male	7	-1.5149969	0.41330791
A3927_262	13138	*O. tsutsugamushi*	35	male	7	-1.0789415	0.58250695
A3927_263	13141	*Rickettsia* sp.	53	male	7	-1.3965248	1.2138481
A3927_264	13165	*A. baumanii*	54	male	6	-1.9451865	-0.639857
A3927_265	13168	*E. coli*	21	female	3	-0.3258542	1.86913276
A3927_266	13205	*O. tsutsugamushi*	52	female	7	-0.5715867	1.88856915
A3927_267	13209	*O. tsutsugamushi*	58	female	7	-2.0228829	-0.549114
A3927_268	13210	*S. typhi*	18	male	7	-1.439958	0.134612
A3927_269	13268	*S. typhi*	30	female	8	-1.1609706	1.12376916
A3927_270	13294	*E. coli*	54	female	6	-1.2726039	0.26146321
A3927_271	13306	*E. coli*	53	female	3	0.4312623	1.7334704
A3927_272	13337	*S. oralis*	15	female	5	-1.8670762	0.86444044
A3927_273	13354	*S. aureus*	40	male	10	-3.2123484	-1.9186084
A3927_274	13398	*E. coli*	17	female	4	1.87153828	2.216926
A3927_275	13441	*O. tsutsugamushi*	30	female	6	-0.151075	2.90558887
A3927_276	13457	*O. tsutsugamushi*	25	female	6	-1.5792693	1.56722631
A3927_277	13489	*Rickettsia* sp.	33	female	8	-1.4823442	1.29393368
A3927_278	13573	*S. typhi*	32	male	14	-2.0661619	0.90438807
A3927_279	13578	*E. coli*	62	female	4	0.28045882	1.76966989
A3927_280	13599	*S. typhi*	42	female	17	-2.4143316	0.4527292
A3927_281	13655	*S. typhi*	17	male	18	-1.9979776	0.36928285
A3927_282	13697	*S. typhi*	25	male	4	-1.0771982	0.71887155
A3927_283	13698	*E. coli*	50	female	3	-11.666667	0
A3927_284	13781	*S. typhi*	25	male	7	-1.6000734	0.52132874
A3927_285	13795	*S. pneumoniae*	26	male	5	1.59725183	3.06282355
A3927_286	13803	*E. coli*	78	male	30	3.16054764	3.60446798
A3927_287	13823	*S. typhi*	27	female	30	0.67581219	2.79915108
A3927_288	13836	*Leptospira* sp.	32	female	90	-1.7390053	0.78712755

Table S11: Demographic and clinical characteristics of the subjects from the Nepal prospective validation cohort, related to Figure 5 and Figur

HHV6, Human Herpesvirus 6; CMV, Cytomegalovirus

ID	Age	Sex	Days since fever onset	Sweeney7 Score	BoVI Score	Rickettsia	HHV6	CMV	Lepto-spirosis	Parvovirus B19	Enterovirus	Typhoid/ Paratyphoi	Other Bacteremia
MD-1271	23	Male	5	4.35671117	2.3223715	NA	NA	NA	1	NA	NA	NA	NA
MD-1291	27	Male	4	0.73432697	-0.7710399	NA	NA	NA	NA	NA	NA	1	NA
MD-1305	25	Female	3	1.52998749	0.02202964	NA	NA	NA	NA	NA	NA	1	NA
MD-1307	26	Male	30	1.00187608	-0.5777194	NA	NA	NA	NA	NA	NA	1	NA
MD-1311	18	Male	3	2.42445032	0.98989613	NA	NA	NA	NA	NA	NA	1	NA
MD-1316	25	Male	13	2.08013424	0.50908894	NA	1	NA	NA	NA	NA	NA	NA
MD-1318	20	Female	4	1.0635897	-0.6678065	NA	NA	NA	NA	NA	NA	1	NA
MD-1342	35	Female	4	1.66562992	0.440424	1	NA	NA	NA	NA	NA	NA	NA
MD-1343	19	Male	3	1.30865015	0.15870642	NA	NA	NA	NA	NA	NA	1	NA
MD-1368	31	Female	3	1.58971076	0.50186351	NA	NA	NA	NA	NA	NA	1	NA
MD-1380	55	Female	6	2.34527005	0.89877188	1	NA	NA	NA	NA	NA	NA	NA
MD-1389	15	Female	9	0.5619972	0.18905936	NA	NA	NA	NA	NA	NA	NA	1
MD-1398	20	Female	5	0.88372359	0.28834722	1	NA	NA	NA	NA	NA	NA	NA
MD-1400	36	Male	7	1.20414882	0.03104493	NA	NA	NA	NA	NA	NA	1	NA
MD-1420	31	Male	5	5.00541109	2.05086348	NA	NA	NA	NA	NA	NA	NA	1
MD-1421	65	Female	3	1.74478929	-0.264004	NA	NA	NA	NA	NA	NA	NA	1
MD-1430	19	Male	3	0.54188317	-0.3656634	NA	NA	NA	NA	NA	NA	1	NA
MD-1436	85	Female	4	2.22611925	-0.3602213	NA	NA	NA	NA	NA	NA	NA	1
MD-1447	47	Female	3	2.68513843	1.42418338	NA	NA	NA	1	NA	NA	NA	NA
MD-1459	54	Male	4	1.84798906	0.30013943	1	NA	NA	NA	NA	NA	NA	NA
MD-1461	19	Female	5	0.87076873	0.68479991	1	NA	NA	NA	NA	NA	NA	NA
MD-1498	23	Female	3	2.01938907	0.51344586	NA	NA	NA	NA	NA	NA	1	NA
MD-1501	23	Male	3	-0.0325583	-2.9283768	NA	1	NA	NA	NA	NA	NA	NA
MD-1517	56	Male	10	1.92912896	2.23939672	1	NA	NA	NA	NA	NA	NA	NA
MD-1522	19	Female	4	2.30401256	1.153923	NA	NA	NA	NA	NA	NA	1	NA
MD-1525	72	Male	10	1.40643515	1.11042719	1	NA	NA	NA	NA	NA	NA	NA
MD-1526	27	Male	7	1.39362338	0.12945331	NA	NA	NA	NA	NA	NA	1	NA
MD-1568	55	Female	4	2.49605219	1.31565851	NA	NA	NA	1	NA	NA	NA	NA
MD-1572	28	Female	6	3.76007714	0.35243434	NA	1	NA	NA	NA	NA	NA	NA
MD-1577	22	Female	6	1.42916007	0.76210368	1	NA	NA	NA	NA	NA	NA	NA
MD-1580	38	Female	10	1.05589323	1.50155603	1	NA	NA	NA	NA	NA	NA	NA
MD-1582	27	Male	4	1.02536166	-0.0660992	NA	NA	NA	NA	NA	NA	1	NA
MD-1590	18	Female	4	-0.0328625	-0.7002131	1	NA	NA	NA	NA	NA	NA	NA
MD-1595	16	Male	7	1.37035101	-0.9873942	NA	1	NA	NA	NA	NA	NA	NA

MD-1598	18	Female	8	0.94056078	0.6567604	1	NA	NA	NA	NA	NA	NA	NA
MD-1599	36	Female	3	4.50367837	1.78411295	NA	NA	NA	1	NA	NA	NA	NA
MD-1602	40	Male	12	2.12709135	1.69244181	1	NA	NA	NA	NA	NA	NA	NA
MD-1632	19	Female	30	2.4378046	1.67126446	1	NA	NA	NA	NA	NA	NA	NA
MD-1664	23	Female	21	0.34510238	-0.3446884	1	NA	NA	NA	NA	NA	NA	NA
MD-1706	16	Female	14	1.2404258	0.13557942	1	NA	NA	NA	NA	NA	NA	NA
MD-1707	24	Male	6	1.67443513	1.53289473	1	NA	NA	NA	NA	NA	NA	NA
MD-1726	23	Male	7	1.58417347	-0.1799893	NA	NA	NA	NA	NA	NA	1	NA
MD-1749	55	Female	7	0.38462493	-0.2736644	1	NA	NA	NA	NA	NA	NA	NA
MD-1776	25	Female	3	3.75764928	2.10848077	NA	NA	NA	NA	NA	NA	NA	1
MD-1778	26	Female	4	-0.2023802	-0.2051605	NA	1	NA	NA	NA	NA	NA	NA
MD-1823	54	Female	5	-0.7735589	-2.0301234	NA	1	NA	NA	NA	NA	NA	NA
MD-1868	60	Male	20	3.44449292	2.58501982	1	NA	NA	NA	NA	NA	NA	NA
MD-1917	49	Female	10	0.58729605	0.94206906	1	NA	NA	NA	NA	NA	NA	NA
MD-1921	19	Female	4	1.18060535	0.65060506	NA	NA	NA	NA	NA	NA	1	NA
MD-1923	27	Female	5	0.93376907	0.44376873	NA	NA	NA	NA	NA	NA	1	NA
MD-1989	20	Female	3	2.07521157	0.92470646	NA	NA	NA	1	NA	NA	NA	NA
MD-2017	45	Male	6	0.83799984	-0.362129	1	NA	NA	NA	NA	NA	NA	NA
MD-2041	35	Male	5	1.21211683	0.46209027	NA	NA	NA	NA	NA	NA	1	NA
MD-2047	24	Male	3	2.93995688	0.44849113	NA	NA	NA	NA	NA	NA	NA	1
MD-2121	37	Male	30	7.76582532	3.89314657	NA	NA	NA	NA	NA	NA	NA	1
MD-2137	60	Female	20	0.44018579	-0.8305989	NA	1	NA	NA	NA	NA	NA	NA
MD-2143	16	Male	7	2.16213705	1.4993639	NA	NA	NA	NA	NA	NA	1	NA
MD-2145	19	Male	7	2.26570034	0.67953089	NA	NA	NA	NA	NA	NA	1	NA
MD-2146	19	Female	5	1.79734891	1.23153263	NA	NA	NA	NA	NA	NA	1	NA
MD-2176	26	Female	7	1.72699161	1.32709247	1	NA	NA	NA	NA	NA	NA	NA
MD-2228	20	Male	3	1.91414899	0.39995173	NA	NA	NA	NA	NA	NA	1	NA
MD-2255	19	Male	4	1.20404435	0.53145327	NA	NA	NA	NA	NA	NA	1	NA
MD-2260	21	Male	4	1.49500072	0.31078718	NA	NA	NA	NA	NA	NA	1	NA
MD-2278	18	Male	15	1.23282067	0.20881076	NA	NA	NA	NA	NA	NA	1	NA
MD-2300	18	Male	3	1.93279754	0.70837782	NA	NA	NA	NA	NA	NA	1	NA
MD-2329	38	Male	4	0.85112483	-0.2129888	NA	1	NA	NA	NA	NA	NA	NA
MD-2351	16	Male	6	0.07368202	-1.3396312	1	NA	NA	NA	NA	NA	NA	NA
MD-2357	24	Female	3	-1.7243634	-4.0165012	NA	NA	NA	NA	NA	NA	NA	1
MD-2370	54	Male	14	5.04274998	3.16628307	NA	NA	NA	NA	NA	NA	NA	1
MD-2411	43	Male	4	2.62780069	0.17091187	NA	NA	NA	NA	NA	NA	NA	1
MD-2443	24	Male	4	2.23109872	0.00340875	NA	NA	NA	NA	NA	NA	1	NA
MD-2459	18	Female	3	2.50289207	1.29214793	NA	NA	NA	NA	NA	NA	1	NA
MD-2460	19	Female	3	2.73541704	1.95672423	NA	NA	NA	NA	NA	NA	1	NA

MD-2520	49	Female	7	5.80562441	4.44124376	NA	NA	NA	NA	NA	NA	NA	1
MD-2543	17	Male	7	1.67827799	1.15773326	NA	NA	NA	NA	NA	NA	1	NA
MD-2547	20	Male	4	1.16470352	0.7258623	NA	NA	NA	NA	NA	NA	1	NA
MD-2555	40	Female	3	5.03905619	3.10703922	NA	NA	NA	NA	NA	NA	NA	1
MD-2571	41	Male	4	1.07023112	1.09766139	NA	NA	NA	NA	NA	NA	1	NA
MD-2611	27	Female	7	0.81632679	-1.8816253	NA	1	NA	NA	NA	NA	NA	NA
MD-2639	35	Female	5	1.43169438	0.17998754	1	NA	NA	NA	NA	NA	NA	NA
MD-2665	45	Female	4	1.68675982	0.44044194	NA	NA	NA	NA	NA	NA	1	NA
MD-2668	50	Female	3	5.1932359	3.21385732	NA	NA	NA	NA	NA	NA	NA	1
MD-2684	70	Female	5	6.01072102	3.01442941	NA	NA	NA	NA	NA	NA	NA	1
MD-2689	41	Female	3	1.85510265	1.59876459	1	NA	NA	NA	NA	NA	NA	NA
MD-2697	17	Female	4	1.62488167	0.79288649	NA	NA	NA	NA	NA	NA	1	NA
MD-2723	60	Male	8	1.10866383	0.78099576	1	NA	NA	NA	NA	NA	NA	NA
PD-423	9	Male	10	1.07687789	0.20797101	NA	NA	NA	NA	NA	NA	NA	1
PD-473	13	Male	5	1.62335746	0.87489941	1	NA	NA	NA	NA	NA	NA	NA
PD-474	11	Female	6	0.93176607	-0.5208058	1	NA	NA	NA	NA	NA	NA	NA
PD-490	13	Female	9	1.44773646	-0.1022349	1	NA	NA	NA	NA	NA	NA	NA
PD-560	5	Male	3	2.77162574	0.26226301	NA	1	NA	NA	NA	NA	NA	NA
PD-592	10	Female	13	1.78033118	-0.3250347	NA	NA	1	NA	NA	NA	NA	NA
PD-619	2	Male	8	-0.6428226	-1.8703919	NA	NA	1	NA	NA	NA	NA	NA
PD-634	5	Male	3	-1.6908361	-2.7067134	NA	1	NA	NA	NA	NA	NA	NA
PD-642	7	Male	6	-2.6197061	-3.3044409	NA	1	NA	NA	NA	NA	NA	NA
PD-653	15	Female	3	-0.6218849	-2.705731	NA	NA	NA	NA	NA	NA	1	NA
PD-676	15	Male	3	0.92313135	0.78402417	NA	NA	NA	NA	NA	NA	1	NA
PD-702	5	Female	7	-1.1616615	-2.0989911	NA	NA	NA	NA	NA	1	NA	NA
PD-709	6	Female	10	1.3972632	-0.8962867	NA	1	NA	NA	NA	NA	NA	NA
PD-714	2	Female	5	-0.8689453	-1.1010541	NA	NA	1	NA	NA	NA	NA	NA
PD-723	14	Female	5	0.96163498	0.96361867	NA	NA	NA	NA	NA	NA	1	NA
PD-743	4	Male	3	-0.2185531	-2.5971428	NA	NA	NA	NA	NA	1	NA	NA
PD-746	11	Male	4	1.27318504	0.52120528	NA	NA	NA	NA	NA	NA	1	NA
PD-748	9	Male	3	1.1562908	-1.1388686	NA	NA	NA	NA	NA	1	NA	NA
PD-750	6	Female	4	-1.6687056	-3.5335519	NA	NA	NA	NA	NA	1	NA	NA
PD-753	6	Male	7	0.59552809	-0.4418712	NA	1	NA	NA	NA	NA	NA	NA
PD-755	2	Male	6	-1.1908377	-2.5323262	NA	1	NA	NA	NA	NA	NA	NA
PD-762	4	Male	6	-0.5776425	-1.1508679	NA	NA	NA	NA	NA	1	NA	NA
PD-783	4	Male	4	-1.8371084	-4.0605512	NA	NA	NA	NA	NA	1	NA	NA
PD-799	5	Male	4	0.48332088	-1.2126503	NA	1	NA	NA	NA	NA	NA	NA
PD-863	11	Female	4	0.72803281	-0.1961533	NA	NA	NA	NA	NA	NA	1	NA

Table S12: TaqMan assays used in Fluidigm validation, related to Figure 5 and Figure S11.
Assays were normalized using the geometric mean of three housekeeping genes: 18S, ACTB, and KPNA6.

Gene Symbol	Gene Name	Alias	RefSeq	Thermofisher ID	fullname	Amplicon Length	Best Coverage	3' Most	Signature	in Laos validation set	in Nepal validation set
18S	Eukaryotic 18S rRNA			Hs99999901_s1	18S_Hs99999901_s1	187	No	No	control	y	y
ACTB	actin beta	BRWS1; PS1TP5BP1	NM_001101.3	Hs01060665_g1	ACTB_Hs01060665_g1	63	Yes	No	control	y	y
FARP1	FERM; ARH/RhoGEF and pleckstrin domain protein 1	CDEP; FARP1-IT1; PLEKHC2; PPP1R75	NM_005766.3;XM_011521046.2;XM_017020312.1;XM_017020313.1;NM_001286839.1	Hs00195010_m1	FARP1_Hs00195010_m1	65	Yes	No	control	y	n
FPGS	folylpolyglutamate synthase		XM_005251864.3;NM_001018078.2;NR_110170.1;XM_011518439.2;NM_001288803.1;NM_004957.5;XM_017014565.1;XM_017014566.1	Hs00191956_m1	FPGS_Hs00191956_m1	116	Yes	Yes	control	y	n
GAPDH	glyceraldehyde-3-phosphate dehydrogenase	G3PD; GAPD; HEL-S-162eP	NM_002046.5;NM_001256799.2;NM_001289745.1;NM_001289746.1	Hs02786624_g1	GAPDH_Hs02786624_g1	157	Yes	No	control	y	n
KPNA6	karyopherin subunit alpha 6	IPOA7; KPNA7	NM_012316.4;XM_005270711.3	Hs00202389_m1	KPNA6_Hs00202389_m1	76	Yes	No	control	y	y
PEX16	peroxisomal biogenesis factor 16	PBD8A; PBD8B	NM_057174.2;XM_011520474.1;NM_004813.2	Hs00191337_m1	PEX16_Hs00191337_m1	75	Yes	No	control	y	n
RPS4Y1	ribosomal protein S4; Y-linked 1	RPS4Y; S4	NM_001008.3;XM_017030061.1	Hs00606158_m1	RPS4Y1_Hs00606158_m1	105	Yes	Yes	control (distinguishes male/female)	y	n
EBI3	Epstein-Barr virus induced 3	IL-27B; IL27B	NM_005755.2;XM_011527619.2	Hs01057148_m1	EBI3_Hs01057148_m1	64	Yes	No	BoVI signature	y	y
FCER1A	Fc fragment of IgE receptor Ia	FCE1A; FcERI	NM_002001.3	Hs00758600_m1	FCER1A_Hs00758600_m1	105	Yes	Yes	BoVI signature	y	y
HESX1	HESX homeobox 1	ANF; CPHD5; RPX	XM_011534205.2;NM_003865.2;XM_005265526.4;XM_017007421.1;XM_011534204.2;XM_006713379.3	Hs00172696_m1	HESX1_Hs00172696_m1	110	Yes	No	BoVI signature	y	y

ICAM1	intercellular adhesion molecule 1	BB2; CD54; P3.58	NM_000201.2	Hs00164932_m1	ICAM1_Hs00164932_m1	87	Yes	No	BoVI signature	y	y
IFI27	interferon alpha inducible protein 27	FAM14D; ISG12; ISG12A; P27	NM_005532.4;NM_001130080.2	Hs01086370_m1	IFI27_Hs01086370_m1	71	No	No	BoVI signature*	y	y
IFI27	interferon alpha inducible protein 27	FAM14D; ISG12; ISG12A; P27	NM_001288952.1;NM_001288954.1;NM_005532.4;NM_001130080.2;NM_001288995.1;NM_001288956.1;NM_001288958.1;NM_001288957.1	Hs00271467_m1	IFI27_Hs00271467_m1	63	No	No	BoVI signature*	y	y
IFI27	interferon alpha inducible protein 27	FAM14D; ISG12; ISG12A; P27	NM_005532.4;NM_001288960.1;NM_001288959.1;NM_001288956.1;NM_001288958.1;NM_001288957.1;NM_001288952.1;NM_001288954.1;NM_001130080.2;NM_001288995.1	Hs01086373_g1	IFI27_Hs01086373_g1	68	Yes	Yes	BoVI signature*	y	y
JUP	junction plakoglobin	ARVD12; CTNNG; DP3; DPIII; PDGB; PKGB	NM_021991.2;XM_017024588.1;XM_011524753.2;XM_017024589.1;XM_017024590.1;XM_011524755.1;XM_011524756.1;XM_011524757.2;XM_006721878.1;XM_011524758.1;NM_002230.2;XM_006721875.1;XM_006721874.2;XM_006721873.2	Hs00158408_m1	JUP_Hs00158408_m1	66	Yes	No	BoVI signature	y	y
SMARCD3	SWI/SNF related; matrix associated; actin dependent regulator of chromatin; subfamily d; member 3	BAF60C; CRACD3; Rsc6p	NM_001003801.1;XM_017012556.1;XM_011516521.2;NM_003078.3;NM_001003802.1	Hs00162003_m1	SMARCD3_Hs00162003_m1	109	Yes	No	BoVI signature	y	y
SUCLG2	succinate-CoA ligase GDP-forming beta subunit	GBETA	XM_017007417.1;XM_017007416.1;XM_017007419.1;XM_017007418.1;XM_017007420.1;NM_001177599.1;NM_003848.3;XM_017007415.1	Hs00896917_g1	SUCLG2_Hs00896917_g1	107	Yes	Yes	BoVI signature	y	y

* averaged Ct for all IFI27 assays; similar results obtained using each individual assay (see Methods)
